# Identification of quantitative trait loci associated with upper temperature tolerance in turbot, *Scophthalmus maximus*

**DOI:** 10.1038/s41598-021-01062-3

**Published:** 2021-11-09

**Authors:** Aijun Ma, Zhihui Huang, Xin-an Wang, Yuhui Xu, Xiaoli Guo

**Affiliations:** 1 Shandong Key Laboratory of Marine Fisheries Biotechnology and Genetic Breeding; Qingdao Key Laboratory for Marine Fish Breeding and Biotechnology, Yellow Sea Fisheries Research Institute, Chinese Academy of Fishery Sciences, No.106 Nanjing Road, Qingdao, 266071 Shandong China; 2 Laboratory for Marine Biology and Biotechnology, Pilot National Laboratory for Marine Science and Technology (Qingdao), Qingdao, 266237 China; 3 Biomarker Technologies Corporation, Beijing, 101300 China

**Keywords:** Genetic techniques, Molecular biology, Genetics, Genetic linkage study, Inbreeding

## Abstract

Temperature tolerance is an important trait from both an economic and evolutionary perspective in fish. Because of difficulties with measurements, genome-wide selection using quantitative trait loci (QTLs) affecting Upper temperature tolerance may be an alternative for genetic improvement. Turbot *Scophthalmus maximus* (L.) is a cold-water marine fish with high economic value in Europe and Asia. The genetic bases of upper temperature tolerance (UTTs) traits have been rarely studied. In this study, we constructed a genetic linkage map of turbot using simple sequence repeats (SSRs) and single nucleotide polymorphism (SNP) markers. A total of 190 SSR and 8,123 SNP were assigned to 22 linkage groups (LGs) of a consensus map, which spanned 3,648.29 cM of the turbot genome, with an average interval of 0.44 cM. Moreover, we re-anchored genome sequences, allowing 93.8% physical sequences to be clustered into 22 turbot pseudo-chromosomes. A high synteny was observed between two assemblies from the literature. QTL mapping and validation analysis identified thirteen QLTs which are major effect QTLs, of these, 206 linked SNP loci, and two linked SSR loci were considered to have significant QTL effects. Association analysis for UTTs with 129 QTL markers was performed for different families, results showed that eight SNP loci were significantly correlated with UTT, which markers could be helpful in selecting thermal tolerant breeds of turbot. 1,363 gene sequences were genomically annotated, and 26 QTL markers were annotated. We believe these genes could be valuable candidates affecting high temperatures, providing valuable genomic resources for the study of genetic mechanisms regulating thermal stress. Similarly, they may be used in marker-assisted selection (MAS) programs to improve turbot performance.

## Introduction

Turbot *Scophthalmus maximus* (L.) is a cold-water marine fish, which was introduced to China in 1992, and is an important economic species of wild fisheries. In China, turbot culture represents a large proportion of land-based tank-cultured fish, in particular in recirculating aquaculture systems, and the annual production reached 49.5 kilo tons, which is accounted for more than 80% of total global aquaculture output over the last decade^1^. Turbot culture has become the country’s largest marine industry on the north coast of China and has driven the development of other marine fish culture industries^2^. In recent years, undesirable phenomena in the turbot industry have emerged from time to time, damaging the development of this industry in China^3,4^. Increasing growth rate, controlling sex ratios, enhancing disease resistance, and enhancing environment tolerance, currently constitute the main goals of genetic breeding programs for this species.

From an economic and evolutionary point of view, temperature tolerance is an important trait of fish, especially for cool- and cold-water turbot. However, turbot is difficult to culture in Northern China during the summer, where natural seawater temperatures often exceed 26 °C^5^. Such high temperatures can cause stress responses in these fishes, resulting in increased mortality, decreased growth rate and disease resistance^6^. Therefore, genetically improving the temperature tolerance of turbot, and breeding new high-temperature-tolerant varieties with excellent growth performance, has become an urgent task for energy conservation, environmental protection, improvement of breeding efficiency and healthy breeding level.

Traditional genetic improvement methods have relied on family and individual selection, based on phenotype and pedigree information^7^. However, thermal tolerance is a multi-genetic trait, which is controlled by multi-loci across the genome, in combination with environmental influences^8^. Genetic characteristics are difficult to determine, i.e. associating phenotypes with genotype. According to estimations of genetic parameters for upper thermal tolerance (UTTs) traits, the heritability is low, however, according to the classification levels of Xu et al. (2015), UTTs is relative to moderate heritability^9,10^. Thus, the construction of high-density genetic linkage maps, and the analysis of quantitative trait loci (QTLs) for marker-assisted selection (MAS) is of significant value, as it accelerates breeding processes, reduces population sizes and breeding costs of bringing progeny to maturity^11,12^. To date, QTLs have been mapped for UTTs in rainbow trout, Arctic Charr, Chinook salmon^13–15^.

QTLs for molecular breeding operations not only require molecular markers to be closely linked to target genes, but they also require that the quantitative trait is practical and reproducible, and is detectable in large numbers of individuals. However, only a few study have been validated across families and different genetic backgrounds, a factor essential for implementation in MAS programs^12,16^. Therefore, before QTLs are used for marker-assisted selection, several issues must be addressed: Are genetic effects identified by these QTLs stable? Which markers are shared among different families? Are there specific markers? Answers to these questions are crucial for MAS programs.

Several studies have demonstrated the genetic basis of growth and sex determination in turbot, thereby contributing to marker-assisted turbot breeding^17–19^. However, genetic and genomic studies are rare for UTTs traits in this fish. After the *S. maximus* genome sequence was published^20^, SNP markers were integrated to build genetic maps for locating QTLs. Specific length amplified fragment sequencing (SLAF-seq) is a recently reported method for SNP discovery and genotyping, based on next generation sequencing (NGS) technologies^21^. In contrast to conventional methods of molecular marker development and genotyping, SLAF-seq is more cost-effective and flexible^22^. Several high-density genetic maps based on SLAF-seq have been recently built in a variety of species, including sesame, kiwifruit, carp and shrimp^22–25^.

In this study, we performed linkage analysis to identify genomic regions involved in the expression of UTTs trait in turbot. Our study focused on the following elements: (1) High-density linkage map construction using two types of molecular markers, i.e. SSRs and SNPs. We used SLAF-seq analysis in F1 mapping population derived from tolerant and sensitive turbot strains. (2) The identification of genomic regions involved in heat tolerance using QTL mapping, and the validation of UTTs-QTL markers in different families, and (3) identifying potential candidate genes in UTTs-QTL intervals. Our study provides a genetic analysis of turbot upper thermal tolerance traits, and valuable genomic resources for molecular breeding programs in this commercially vital fish.

## Materials and methods

All methods were carried out in accordance with Yellow Sea Fisheries Research Institute guidelines and regulations. The study was carried out in compliance with the ARRIVE (Animal Research: Reporting of In Vivo Experiments) guidelines. All experimental protocol were approved by the institutional ethic committee of Yellow Sea Fisheries Research Institute.

### Mapping family preparation

A selective breeding program for thermotolerance in turbot (*Scophthalmus maximus* L.) was carried out at the Yellow Sea Fisheries Research Institute, Chinese Academy of Fishery Sciences in 2007^10^. High temperature tolerance strains and sensitive strains were produced by our team, through a series of selection experiments which used survival data and UTT (calculated as cumulative thermal exposure in degree hours) obtained from a thermal challenge^10,26^. Following matings, selection for high temperature tolerance strains and sensitive strains were conducted for three successive generations. An F2 family derived from a cross of high temperature tolerant strains and from sensitive strains. Finally, with 140 progenies was obtained and used for map construction and QTL detection. The mapping family used in this study was established on May 28, 2014 via artificial insemination in Tianyuan Fisheries Co. Ltd. hatchery plant (Yantai city, Shandong Province, P. R. China).

### Trait measurement and DNA extraction

Offspring of family (approximately 140 individuals), when the family reached 10 months of age and 90–120 g were acclimatized at a normal factor (14 °C, salinity 30‰) for two weeks. Fish were subject to chronic thermal shock, similarly to the experimental scheme of Diegane Ndong^27^. Temperature was programmed for a 3 °C increment every 48 h, and then held at 29 °C until the end of the experiment. Fish were considered to have died when they lost activity and could not level themselves; at this point they were euthanized with an overdose of clove oil, placed on ice, and tagged individually to indicate time of death. The experiment continued until all fish had succumbed to the thermal challenge. Fins were sampled, and frozen at − 20 °C until genetic analyses could be performed.

In many aquaculture breeding programs, the selection trait for temperature resistance is generally measured as survival or upper thermal tolerance (UTT; calculated as cumulative thermal exposure in degree hours) under a controlled thermal challenge^28^.

UTT = ∑_*j*_(T_*j*_-T_a_) where T_*j*_ is the experimental temperature at each hour, T_a_ is the acclimation temperature (14 °C), and *j* represents each hour up to LOA (loss of activity) for each individual fish. This technique has been reported previously^28^.

DNA of fins was extracted using the TIANGEN marine animal DNA extraction kit (TIANGEN, Beijing, China). Gel electrophoresis and an ND-1000 spectrophotometer (NanoDrop, Wilmington, DE, USA) were used to assess DNA quality and quantity, respectively. A minimum 2 μg DNA was used for SLAF-seq.

### SLAF library construction and genotyping of the mapped population

A total of 121 SLAF libraries were prepared: two parent libraries and 119 progeny libraries. SLAF libraries were prepared according to Sun with minor modifications^29^. The genome of turbot (https://www.ebi.ac.uk/ena/browser/text-search?query=PRJEB11743)^20^was chosen as the reference genome to design the SLAF scheme. For the population, *Hae III* and *Hpy 166II* (NEB, Ipswich, MA, USA) enzyme combinations were applied to digest genomic DNA. Afterwards, dual-index sequencing adapters were ligated to the fragments by T4 ligase (NEB, Ipswich, MA, USA). Subsequently, SLAFs were amplified by PCR, screened, and used to construct SLAF libraries. After samples were gel purified, DNA fragments with indices and adaptors (SLAFs) of 314–414 bp were excised and diluted for pair-end sequencing on an Illumina High-seq 2500 sequencing platform was performed at Beijing Biomarker Technologies Co. Ltd. in Beijing.

SLAF marker identification and genotyping were performed using procedures described by Sun et al.^29^ Briefly, raw reads were separated by barcodes. The sequence quality of each sample reads was evaluated by the GC content and the Q30 (Q = –10*log^e^_10_; indicating a 0.1% chance of an error and thus 99.9% confidence) quality score. According to the similarity between sequences, cluster analysis was used to detect all read sequences. Reads from different sample were classified into one set named SLAF tag. Therefore, SLAF markers differ in sample from other sample as defined by polymorphism. All SLAF marks had been filtered and quality assessed many times by the method described by Sun et al.^29^ (1) All polymorphism SLAFs loci were genotyped with consistency in the offspring and parental SNP loci. (2) SLAFs that had less than three SNPs, and average depths of each sample above three, were considered as high quality SLAFs. (3) These high quality SLAFs with two, three, or four tags were identified as polymorphic SLAFs and considered to be potential markers. (4) Polymorphic markers were classified into eight segregation patterns (ab × cd, ef × eg, hk × hk, lm × ll, nn × np, aa × bb, ab × cc and cc × ab). The marker codes of the polymorphic SLAFs were analyzed according to the population type CP, which removed one segregation type (aa × bb). (5) In a final filtering step, SLAF markers with average sequence depths of more than tenfold in parents, and with integrity of more than 75% in mapping population individuals, were selected for use in genetic mapping.

### SSR genotyping

Sequence information on SSR primers were obtained from the literature, but some were designed in our laboratory (Supplementary Table S1)^30–33^. In our previous study, 294 markers were confirmed as adhering to mapping standards^32^, and these markers were used for constructing an integrated map. PCR products were detected on an 8% denaturing polyacrylamide gel in a tris-base EDTA buffer and silver stained.

### Linkage map construction

Genetic maps were constructed using HighMap software^34^. The modified logarithm of odds (MLOD) scores between markers were calculated to further confirm the robustness of markers for each linkage group (LG). Then, the SMOOTH error correction strategy was conducted according to the parental contribution of genotypes^35^, and a k-nearest neighbor algorithm was applied to impute missing genotypes^36^. Skewed markers were then added to this map, by applying a multipoint maximum likelihood method. Map distances were estimated using the Kosambi mapping function^37^.

### Re-anchoring scaffolds and synteny analysis

The high-density sex-average genetic map with SLAF marker position files and the scaffold sequences^20^ were used as input data. ALLMAPS software^38^ was used to ordering and orientation scaffolds onto the high-density genetic map, and the un-anchored scaffolds were discarded intactly. Co-linearity was used to evaluate the between this newly assembly and the genetic map with default settings (https://github.com/tanghaibao/jcvi/wiki/ALLMAPS). To further investigate the synteny between our re-anchored genome and Maroso’s et al. genome (ASM318616v1)^39^, we adopted MUMmer (Version 3.23) to identify similar regions under default parameters^40^. The synteny circle diagram output was plotted using an R script (in house) based on the physical positions on the same chromosome pairs.

### Analyses of QTL linked to UTT

Normality of the UTT phenotypes was evaluated graphically through normality probability plots using the Proc Univariate Normal command in SAS9.2 (SAS Inst). QTL analysis for UTT was performed, using MapQTL5.0. QTL region detection^41^, the percentage of the phenotypic variance explained, and the genotypic information coefficient were calculated using the interval QTL mapping model^42^. In the QTL mapping step, the LOD threshold for testing the significance of the QTL peaks was calculated using 1, 000 permutations for each of the trait data sets, and a genome-wide significant level of 5%. For interval distances > 1.0 cM, significant thresholds were estimated every 1.0 cM. Considering that the present QTL mapping was based on a single mapping family, these QTLs should also be tested for significance in other mapping families or populations, QTLs with an LOD ≥ 3.0 were used in the present study.

### Analysis and validation of UTT-QTL markers in different families

A total of 129 SNPs were selected from five LGs, using the following criteria: (1) The markers under UTT-QTL peaks and flanking regions were used; (2) Major QTLs explaining phenotypic variance > 20% and (3) LGs containing QTLs were evaluated with at least fifteen markers; (4) Primers targeting these variable regions were designed to amplify fragments around 100 bp and primers melting temperature (Tm) > 60 °C^43^. All 129 markers were genotyped to validation for their relation in mapping family and other eight family (The eight family were random chosen, all the families were included in the experiment were listed in the Supplementary Table S2). And trait measurement as “Trait measurement and DNA extraction” section.

Small amplicon genotyping is a simple yet powerful genotyping technique; primers are designed to amplify fragments of approximately 120 bp using primer 5.0 (http://www.premierbiosoft.com/). The amplification reactions were conducted in a Mastercycler ep gradient S (Eppendorf, Germany). Each PCR reaction contained 40 ng template DNA, 10 μM of each forward and reverse primer, 4.5 μl 2 × ES Taq Master Mix (Takara, China), in a total volume of 10 μl. PCR conditions were defined as: an initial denaturation of 95 °C for 2 min; followed by 35 cycles of 94 °C for 30 s and the annealing temperature (depending on the specific primer Tm) for 30 s, 72 °C for 30 s and a final extension for 7 min at 72 °C. This program also allowed a step for heteroduplex formation by adding the DNA dye, LC Green (Idaho Technology, USA), and heating to 95 °C for 5 min. To avoid evaporation, the 96-well plate containing reactions was overlaid with 15 μl mineral oil. After amplification, the plate was transferred to a Light Scanner (Idaho Technology, USA) where High Resolution Melting Analysis (HRMA) were performed. HRMA is a fast technology for sequence variants detection^44^. Samples were melted at 60–95 °C, in 0.5 °C increments. Melting curve analyses were performed using Light Scanner software, with Call-IT version 2.0 (Idaho Technology, USA).

According to manufacturer’s instructions, in using a curve shape-matching algorithm, samples are automatically clustered into groups, and melting curve and difference plots are inspected. Significant differences in fluorescence in all subsets which indicate different genotypes. The frequency of distribution of genotypes with different SNP loci in resistant and sensitive populations, were analyzed by the chi-square test, using SPSS. A Pearson correlation was performed to analyze correlations between the SNP mutation site, and temperature tolerance traits of turbot.

Genotypes of SNP loci of all individuals in eight families were collected and analyzed. A General Linear Model (GLM) using SPSS 19.0 was used to analyze correlations between genotype and UTT, using the model Y = μ + G_i_ + e_ij_, where Y is UTT; μ is the overall average; G_i_ is the effect of SNP and e_ij_ is random error. SHESIS (http://analysis.bio-x.cn) was used to perform linkage disequilibrium and haplotype analysis for significantly correlated loci^45^.

### Gene annotation of UTT-QTL intervals

The whole genomic sequence of turbot was used to predict the candidate genes that are responsible for the yield^39^. Gene annotation was carried out in the interval of QTL of the genetic map. For Gene Ontology enrichment analysis, AgriGO toolkits^46^ were used with the vertebrate database. Furthermore, we used R package clusterProfilter v3.16 to conduct Kyoto Encyclopedia of Genes and Genomes (KEGG) pathways enrichment analysis^47^. KO terms of the annotated genes within the QTL were used to identify statistically enriched related pathways in the respective genome region, with default cut-offs and default hypergeometric statistical test and false discovery rate (FDR) correction, using a whole genome set as background.

## Results

### Analysis of SLAF-seq data and markers genotyping

Illumina HiSeq 2500 platform generated 418.26 million reads for parents and for 116 progenies. After eliminating the index sequences at both ends, each read was approximately 100 bp. Among them, 86.80% bases were of high-quality, with quality scores of at least 30. A total of 781,520 SLAFs were detected, of which 364,990 were polymorphic (Table 1). Among these polymorphic markers, 232,282 were successfully genotyped in both parents and offspring. The average read depth of genotyped markers was 8.20 in the offspring. In the male and female parents, the average read depth was 35.01 and19.21, respectively (Table 2).

**Table 1:** SLAF marker mining results.

Type	Polymorphic SLAF	Non-polymorphic SLAF	Total SLAF
Number	364,990	416,530	781,520
Percentage	32.40%	67.39%	100%

**Table 2:** Summary of marker depths.

Sample ID	SLAF number	Total depth	Average depth
♂	313,661	6,025,060	19.21
♀	439,186	15,377,774	35.01
Offspring	321,738	2,656,651	8.20

### The genetic map

**Figure 1:**
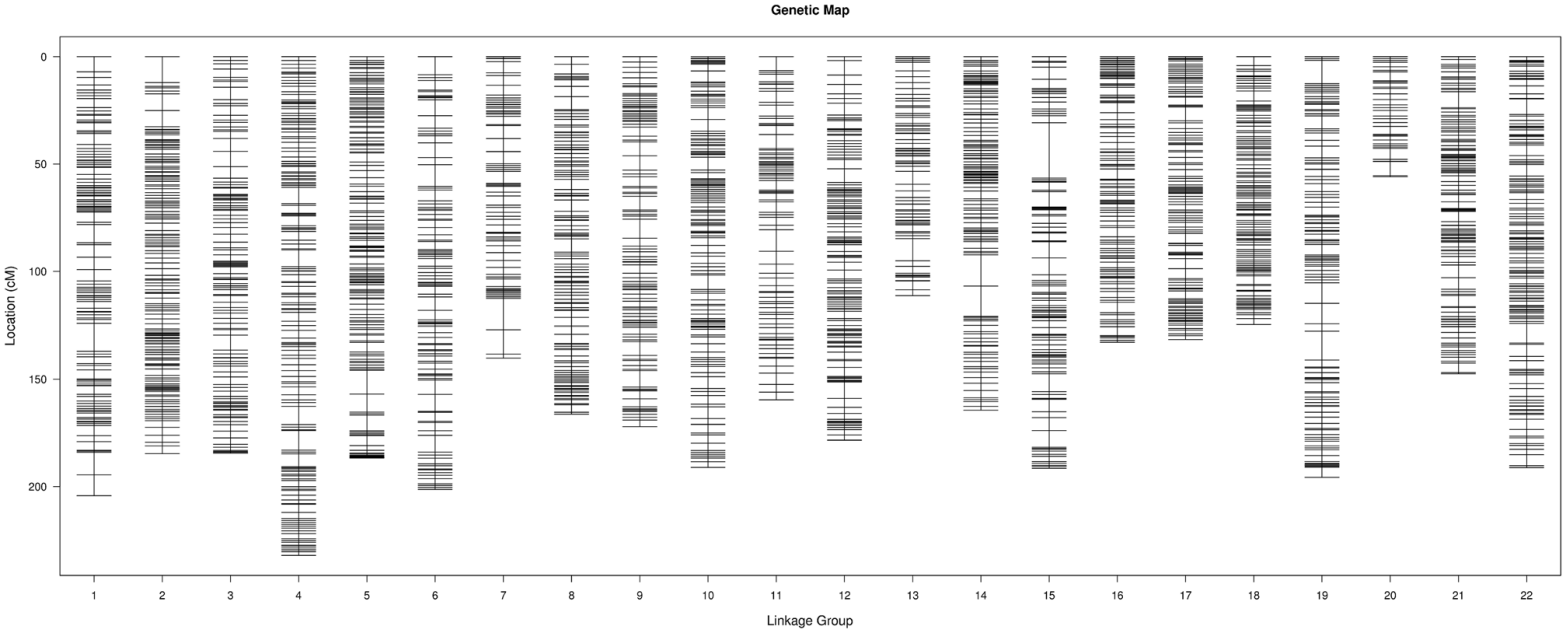
Turbot high-density genetic map.

The group LOD value ranged from 3 to 5, depending on the linkage group. After linkage analysis, 8,123 SNP and 190 SSR markers, including 4,936 markers in the male map, and 5,061 markers in the female map, were mapped onto the sex-averaged genetic map, with 22 LGs (Fig. 1). The total map distances for the three maps were 3,176.68 cM (male map), 4,053.06 cM (female map), and 3,648.29 cM (sex-averaged map). The mean distance between two markers was 0.65 cM (male map), 0.80 cM (female map), and 0.44 cM (sex-averaged map), indicating a high-resolution genetic map (Supplementary Table S3).

### High-density genetic map-assisted scaffolds anchoring and synteny analysis

**Figure 2:**
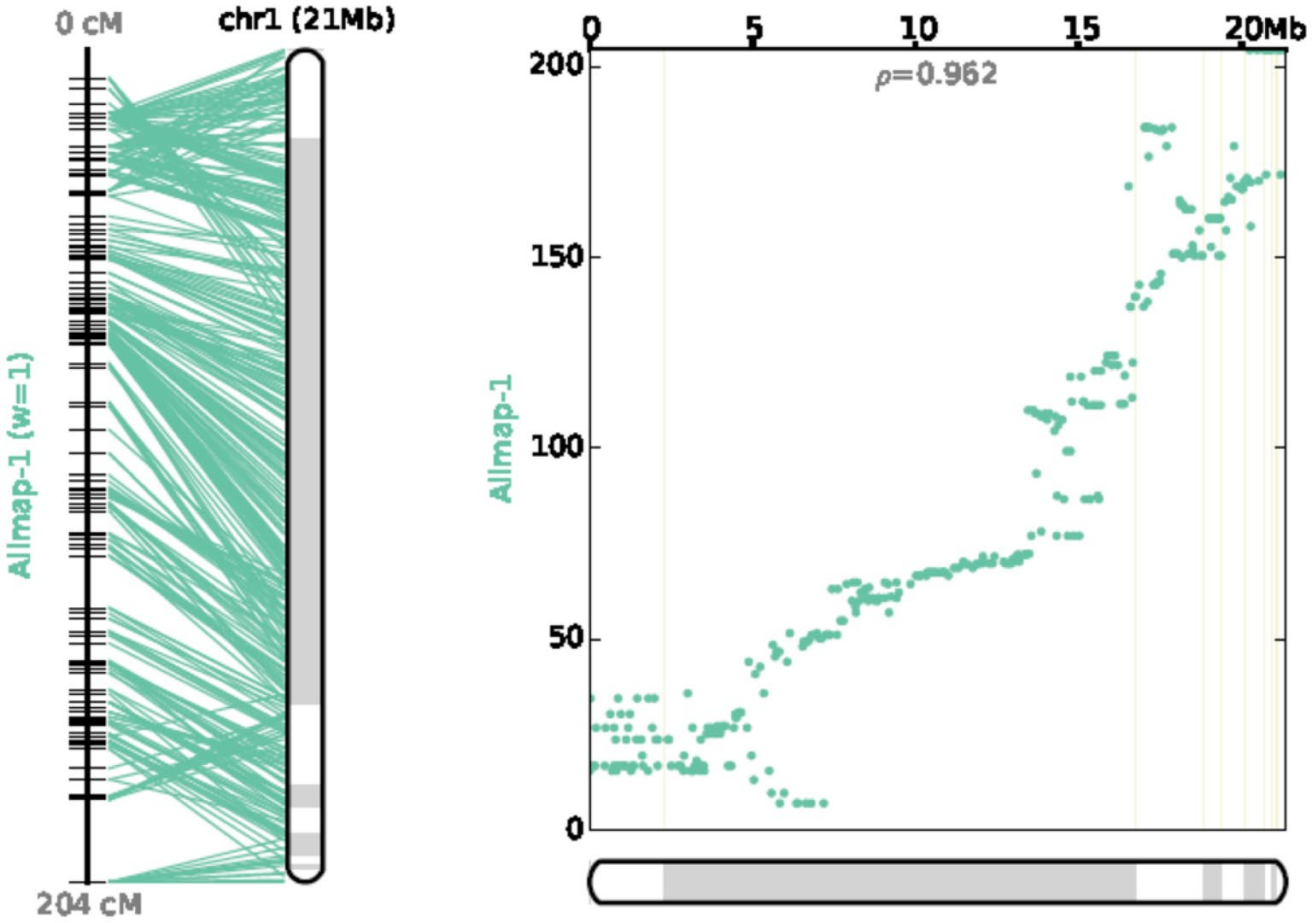
Chromosome 1 co-linearity between the SLAF-based high-density genetic map and the corresponding chromosome assembly by ALLMAP (https://github.com/tanghaibao/jcvi/wiki/ALLMAPS)^49^.

**Figure 3:**
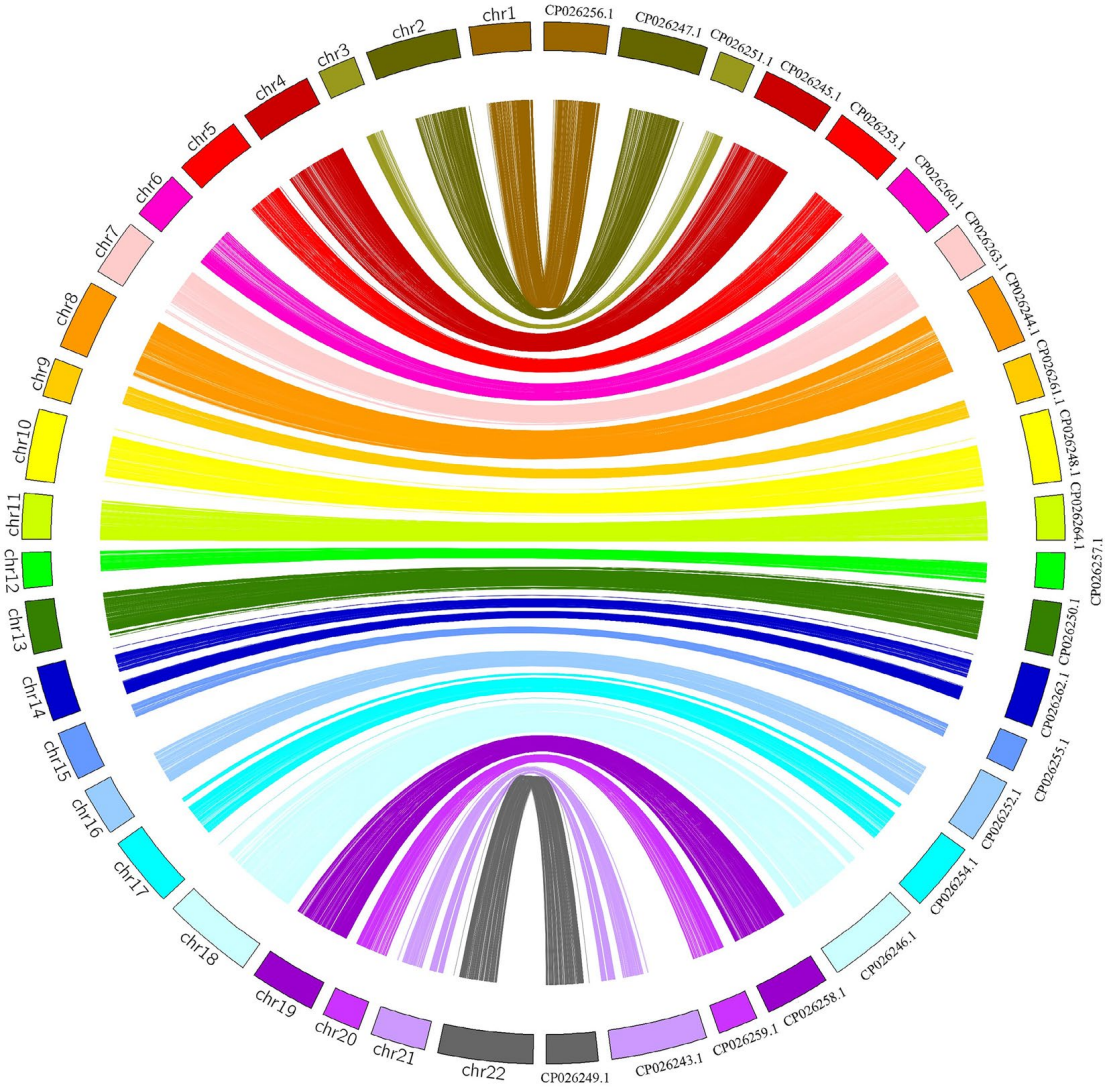
Comparison analysis of the syntenic relationship between the re-anchored genome, and Maroso’s et al. genome (ASM318616v1).

A chromosome-level draft genome of turbot was released in 2016 using a genetic map with 492 available markers^20^. To determine more scaffolds and contigs onto 22 pseudo-chromosomes, we used this high density-genetic map to re-anchor the physical map. Using 8029 uniquely aligned SLAF markers, 317 scaffolds with 510 Mb genome assembly were anchored to turbot’s 22 pseudo-chromosomes, accounting for 93.8% physical sequences. Of the 317 anchored scaffolds, 205 anchored more than two SLAF markers (Table 3). Compared with Figueras’s et al. assembly with 79.7% anchoring rate^20^, we mapped additional 75.62 Mb on this reference genome (Supplementary Table S4). The co-linearity between the genetic map and Figueras’s et al. reference genome (https://www.ncbi.nlm.nih.gov/genome/?term=turbot) was also investigated and high co-linearity was observed (Fig. 2, Supplementary Fig. S1). To further assess this assembly, we analyzed the synteny between our re-anchored genome and Maroso’s et al. genome (ASM318616v1)^39^. Similarly, there were widespread synteny between the two genomes (Fig. 3, Supplementary Table S4). These assessments indicated that a consensus and much higher integrated reference genome was generated.

**Table 3:** Re-anchoring summary information using SLAF-based high density genetic map.

Index	Anchored	Oriented	Unplaced
Unique mapped makers	8029	7453	94
Makers per Mb	15.7	16.1	2.8
Scaffolds	317	205	35
Scaffolds with 1 anchored marker	38	0	21
Scaffolds with 2 anchored markers	34	19	4
Scaffolds with 3 anchored markers	23	8	5
Scaffolds with ≥ 4 anchored markers	222	178	5
Total bases (bp)	510,714,894	463,314,252	33,523,908
Mapping rate	93.80%	85.10%	6.20%

### QTL mapping

Based on the high-density genetic map, QTL mapping of UTT was performed. A total of thirteen putative QTLs were identified, and distributed across five different chromosomes (LG8, LG10, LG13, LG16 and LG22). (Table 4 and Supplementary Fig. S2). Among these QTL linkage groups, qUTT16-4 had the highest LOD (4.97), explaining approximately 28.4% of the phenotypic variation, whereas qUTT16-4 had the lowest LOD (3.02), explaining 21.6% of the phenotypic variation. Of these, 206 linked SNP loci, and two linked SSR loci were considered to have significant QTL effects. Most UTT related QTLs were located in LG16.

**Table 4:** Location of UTT QTLs in turbot.

QTL name	Linkage group (LG)	95% confidence interval (cM)	Flanking markers	Flanking marker interval (cM)	Marker number	Phenotypic variance explained %
qUTT8-1	LG8	104.498–104.498	Marker135981	0	1	22.5
qUTT8-2	LG8	105.368–105.368	Marker135941–Marker136210	0	2	22.5
qUTT10	LG10	6.694–6.694	Marker165156–Marker53684	0	13	22.5
qUTT13-1	LG13	0.0–23.748	Marker227760–Marker237214	23.75	34	26.8
qUTT13-2	LG13	26.478–28.232	Marker93382–Marker93528	1.75	11	24.9
qUTT16-1	LG16	17.941–26.1	Marker177839–Marker187309	8.16	15	28.4
qUTT16-2	LG16	50.154–61.047	Marker186568–Marker79135	10.89	22	26.8
qUTT16-3	LG16	64.559–70.465	Marker79783–Marker79204	5.91	15	23
qUTT16-4	LG16	83.846–85.514	Marker186661–Marker77912	1.67	11	21.6
qUTT16-5	LG16	89.346–101.171	Marker79251–Marker77550	11.83	42	24.4
qUTT16-6	LG16	103.136–109.339	Marker77501–Marker77219	6.2	24	22.2
qUTT22-1	LG22	121.346–121.346	Marker6914–Marker5019	0	2	22.5
qUTT22-2	LG22	168.668–177.33	Marker211746–Marker211686	8.66	16	25.4

### Validation of UTT-QTL markers in different families

HRM analysis is a quick and closed-tube method that detects different genotypes in small PCR fragments. Association analysis for UTT with 129 QTL markers was performed for different families (Supplementary Table S1).

We used GLM to analyze correlations between genotype and UTT of SNP loci. Our results showed that three SNP loci were highly significantly correlated with UTT (p < 0.01) and five were significantly correlated with UTT (*p * < 0.05) (Supplementary Table S5 and Supplementary Fig. S2 Seven out of eight SNP loci that were significantly correlated to UTT were located in the same linkage group (LG16). Linkage disequilibrium analysis (Table 5) showed that three pairs of SNP loci were completely disequilibrated (D' = 1); seven other pairs of loci were closely linked (D' > 0.9). Simultaneously, haplotype analysis of the seven loci showed they constituted 17 haplotypes, among which, eight had frequencies > 0.03, wherein GTCCGCC had the highest frequency, reaching 26.8% (Supplementary Table S6).

**Table 5:** Pair-loci D’ linkage disequilibrium values.

	M78928	M177839	M77078	M76817	M79433	M78790
M79971	0.993	0.644	0.787	0.375	0.983	0.473
M78928	–	0.999	1.000	0.998	0.928	0.457
M177839	–	–	0.632	1.000	0.721	0.292
M77078	–	–	–	0.778	1.000	0.833
M76817	–	–	–	–	0.991	0.986
M79433	–	–	–	–	–	0.777

### Identification and confirmation of candidate genes underlying QTLs for UTT

To identify candidate genes linked to UTT, 1,363 gene sequences were genomically annotated, and 26 QTL markers were annotated (Supplementary Table S7). 388 gene were categorized by GO analysis which classified genes into three main categories (cellular component, molecular function and biological process). Each gene was assigned to one or more GO term. Based on pathway analysis, approximately 882 genes with known functions were bioinformatically identified at QTL regions. Corresponding pathways were identified on the KEGG website (http://www.kegg.jp/). The enrichment analysis showed that 10 KEGG pathways were significantly enriched (using p-adjust values cut-offs (0.05/0.01) for significances), including “ubiquitin proteasome”, “FoxO signaling”, “MAPK signaling”, “JAK-STAT signaling”, “Fatty acid metabolism”, “GnRH signaling”, “Adrenergic signaling in cardiomyocytes” “p53 signaling”, “peroxisome signaling” and “calcium signaling” (Supplementary Table S8, Supplementary Fig. S3). Most pathways were involved in defense responses, in agreement with GO enrichment analyses. Out of 1,363 genes, 34 related with stress responses and with significantly ten pathways were selected as candidate genes for turbot UTTs traits (Supplementary Table S9).

## Discussion

### Construction of a high-density linkage map in turbot using SSRs and SNPs

High-density linkage maps are exceptionally valuable tools in many genetic and genomic applications, such as fine-scale QTL mapping, characterization of recombination hotspots, comparative genome analysis and genome scaffolding^49^. Effective use of this information is necessary for turbot aquaculture. To date, several genetic linkage maps have been constructed for turbot, using SSRs and SNPs^17,18^. Due to the limited number of markers, these maps only contain a few hundred loci. More recently, a high-density map was reported by Wang et al*.*^19^, but few studies have reported an integrated high-density map constructed with SNP and SSR markers. However, the quality and quantity of markers directly affect the construction of high-quality genetic maps. In this study, 190 SSR markers and 8,123 SNP markers were used to construct a high-density map.

SNPs are suitable for automated large-scale genotyping, which can be genotype a large number of effective markers to construct a high-density genetic map^50^. In our research, SLAF-seq was successfully employed for large-scale SNP discovery and genotyping in turbot. In total, 781,520 SLAFs were developed, and 364,990 of these were polymorphic. For this map, we identified 8,123 high-quality SNP markers.

SSR has high polymorphism with codominantly inherited, and are widely distributed throughout the genome^51^. Even if SNP is used for the construction of linkage groups, SSR are still essential for integrating different molecular markers maps, assisting genome assembly, and comparative genome research^52,53^. For this map, we used 294 SSR markers for mapping, of which 190 were anchored. Approximately 5–13 SSR markers were distributed to each LG.

In this study, the total length of the genome was 3,648.29 cM, and the mean distance between two markers was 0.44 cM (sex-averaged map), indicating a high-resolution genetic map. This high-density genetic map will be a useful platform for locating goal traits, identifying QTLs, assisting scaffold anchoring, and identifying candidate genes related to economically important turbot characteristics.

### High-density genetic map-assisted scaffold anchoring

Universally, high-density genetic map-assisted genome anchoring is a traditional strategy for chromosome-level reference genome generating in many species before Hi-C technology appearance^54–56^. The contig/scaffold anchoring rate by a high-density genetic map was associated with the base length of contig N50, and the marker numbers on the genetic map. Here, we used 8,029 unique SLAF makers to re-anchor genome sequences; 93.8% physical sequences were clustered into 22 turbot pseudo-chromosomes (Table 3). Moreover, when compared with Figueras’s et al. assembly^20^, more than 75.62 Mb sequences were arranged, and a high synteny was observed between these two assemblies (Fig. 2), indicating an improved genome assembly of turbot with high consistency with previous assembly^39^. In combining our genetic map with Maroso’s et al. map, this will help us to generate a consensus genetic map to consolidate the reference genome of the turbot^39^. Nowadays, single molecular sequencing technology such as PacBio or Nanopore can greatly increase the contig N50 of genome de novo assembly, and thus will greatly benefit the anchoring efficiency of genetic map-assisted chromosome-level genome anchoring^57,58^.

### QTLs correlate with UTT

The UTTs trait of fish has long been studied, thanks to its importance in aquaculture. However, most contemporary studies have focused on high-density genetic linkage map, and identified less QTLs related to UTTs in fish, but these displayed small effects^13–15,59^. Currently, no QTL analyses for UTTs traits in turbot have been reported, meaning that turbot QTL genetics has lagged behind other species. Therefore, this linkage map and QTL analyses of UTTs traits provides a foundation for future turbot breeding approaches, and identifies genes associated with UTT.

We used a hybrid turbot system, with a high temperature tolerance strain and a sensitive strain. This system provided an ideal analytical mechanism for UTTs. Segregation of chromosomes and F2 trait analysis provided a good system to identify UTTs genes and QTLs. 13 QTLs were associated with UTT, all of which are major effect QTLs (phenotypic variation > 20%). The high phenotypic variation at these loci suggests major genes controlling thermal tolerance in turbot. QTL regions were concentrated on certain LGs (i.e. LG16 and LG22, qUTT 16–5 were located on LG16, including 41 SNP and 1 SSR markers), suggesting that genes from different chromosomes may contribute to the same trait.

### Association analyses

We used QTL mapping to identify 129 candidate SNP loci, which were further analyzed using association analyses, and verified in multiple families. Other studies have shown that linkage maps and association analyses are irreplaceable for the identification of QTL; the two methods are complementary in terms of accuracy and location, the information generated, and statistical analysis methods^60^. We used GLM to perform association analyses, revealing that eight SNP loci were significantly correlated to UTT, of which seven were located with LG16, which was consistent with our UTT QTL mapping data. These results imply that LG16 may be closely related to upper thermal tolerance in turbot.

Linkage disequilibrium between SNP loci is important for population genetics, precise gene mapping and correlation analyses. We performed linkage disequilibrium analysis on seven significantly correlated loci on LG16. Three SNP loci were completely linkage disequilibrium (D' = 1); seven SNP loci were significant linkage disequilibrium (D' > 0.9). We also performed haplotype analysis on seven significantly correlated SNPs located at LG16, which constituted 17 haplotypes. Together with our linkage disequilibrium analysis, one fragment of LG16 was closely correlated to UTT, which was a linkage disequilibrium region. This result was consistent with our association analysis.

### Signal pathways and candidate genes related to UTT

A key objective of this study was to identify genes involved in UTT. It could be argued that further studies should also identify candidate genes belonging to specific pathway, which localized to QTL regions. Some pathways that are over-represented among genes with UTT, and that also localize to QTL regions. Some pathways were significantly over-represented (Supplementary Table S8, Supplementary Fig. S3). These data strongly suggest that apoptosis, oxidative stress, energy metabolism and stress signal transduction play significant roles during thermal stress. Moreover, these results were consistent with our previous findings^61^. Some studies have reported that high temperature challenges increase endogenous ROS in aquatic organisms^62^. ROS overproduction damages important biomolecules, such as DNA, proteins and lipids, and initiates events that impair cellular function^63^. Cells have a well-developed antioxidant defense system to protect against such oxidative stresses^64^. Our results indicated that a number of genes were enriched in peroxisome signaling, the ubiquitin–proteasome pathway and the p53 signaling pathway, and were related to antioxidant defenses and repair mechanisms (Supplementary Table S9).

We identified some genes with putative peroxisome activity, located in two different QTL regions (qUTT16-1 and qUTT16-6). Peroxisomes are ubiquitous and multifunctional organelle with important roles in cellular lipid metabolism and the regulation of oxidative stress-related signaling pathways^65^. Many peroxisomal enzymes catalyze redox reactions in ROS and lipid metabolism, where they execute distinct metabolic functions^66^. An intriguing question for future studies is how do peroxisomes contribute to heat stress responses and metabolic pathways in the turbot.

The ubiquitin–proteasome pathway (UPP) selectively degrades damaged and faulty proteins^67^. Our previous studies have shown that levels of ubiquitin-protein complexes increase under heat shock^61^. In this study, by sequencing and BLAST analysis, six ubiquitin related genes responding to heat stress were identified. These data indicated that UPP may important play roles in heat resistance in turbot^68^.

A number of p53 target genes were located at two QTL regions. P53 target genes (e.g., Waf-1/p21, Gadd45, thrombospondin and cyclin) (Supplementary Table S9) regulate genome stability, cellular responses to DNA damage and progression through the cell cycle^69^. These proteins contain consensus sequences and are regulated by p53. If oxidative stress is detected, p53 increases oxidative stress levels to promote apoptosis, to ensure genetic stability^70^. For example, temperature-induced oxidative stress may cause DNA damage or apoptosis, to cooperatively stimulate p53 expression^71^. P53 can also reduce intracellular ROS by regulating cellular metabolism. The major function of p53 tumor suppressors is to restrict abnormal cells before DNA is damaged or altered causing an inheritable mutation^72^.

**Figure 4:**
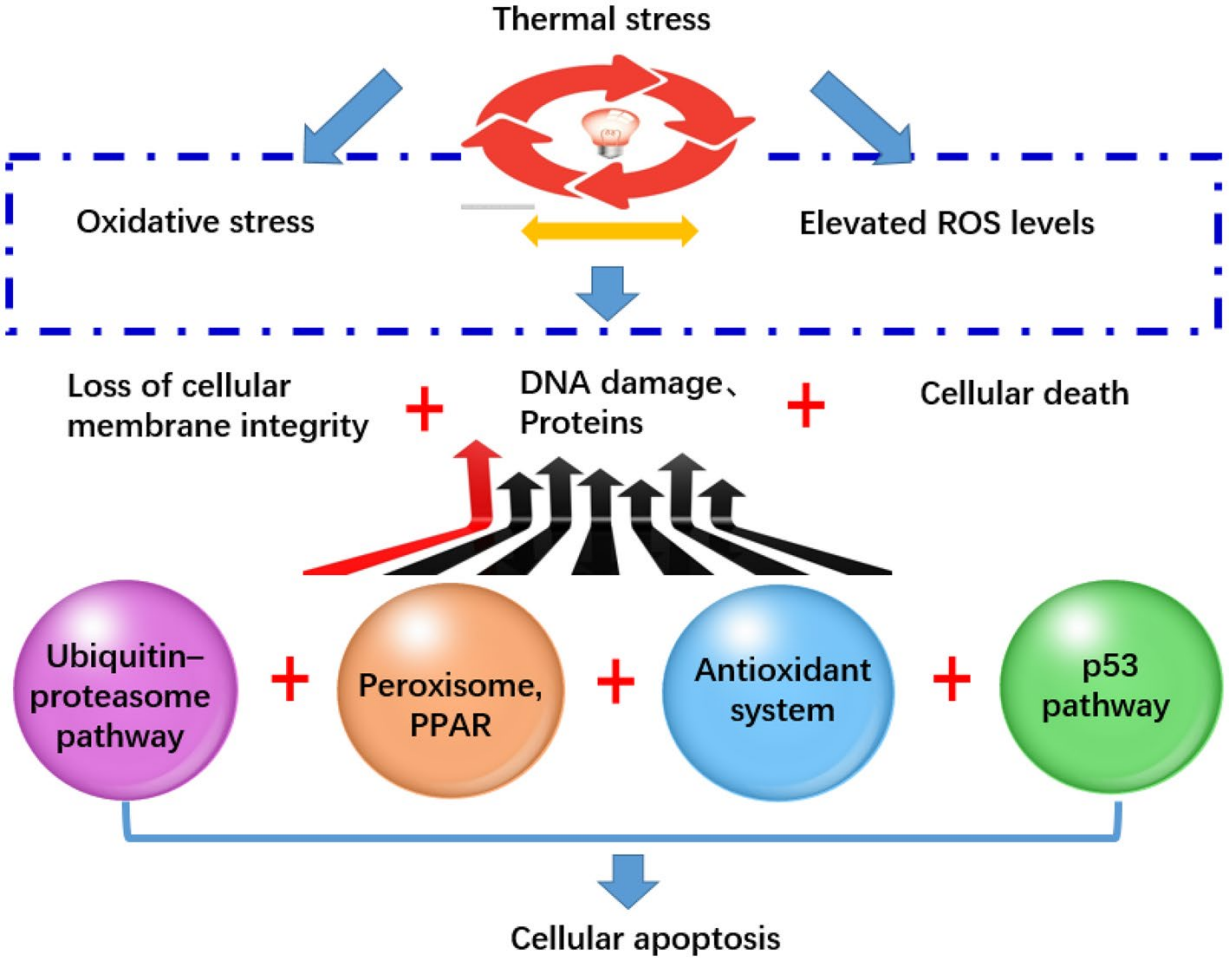
Thermal stress increases ROS generation. When the physiological antioxidant system cannot counteract elevated ROS levels, oxidative stress is induced, resulting in loss of cellular membrane integrity, extensive DNA damage and cell death. Fish have evolved a number of strategies to counteract unfavorable chemical exposures, including antioxidant defenses and cellular apoptosis mechanisms.

We have proposed an efficient approach to identify candidate genes linked to UTT in turbot, exploiting numerous molecular and bioinformatics reagents including a high-density genetic map and a reference genome^73^. Previous studies have confirmed that thermal stress causes systemic hypoxemia and the interaction of temperature and thermally induced hypoxemia will thereby shape acclimation responses at various molecular to whole organism levels^74–76^. Studies of temperature-dependent oxygen supply, mode of metabolism, and associated mechanisms of thermal adaptation in fishes suggested a role of oxygen supply in thermal limitation. Some studies suggested that high temperature induced apoptosis and oxidative stress^77^. However, the precise mechanism and the pathways that are activated in fish are still unclear. In the present study, we demonstrated that fish have evolved a number of strategies to counteract unfavorable chemical exposures, including “ubiquitin proteasome”, “FoxO signaling”, “JAK-STAT signaling”, “Fatty acid metabolism”, “p53 signaling”, “peroxisome signaling” and “calcium signaling”. Among them, antioxidant defenses and cellular apoptosis mechanisms are the most important balancing strategy. To reflect this, we constructed an interacting paradigm based on these pathways (Fig. 4). Thus, our data provides new insights into thermal stress mechanisms induced by oxidative damage, DNA damage and apoptosis in fish.

In this study, we are currently working on QTL validation, and UTT-QTLs associated markers were detected and confirmed across a large set of families, an essential previous step for MAS implementation. 34 candidate genes for UTTs traits were identified; these genes could potentially be utilized for turbot breeding, to improve our understanding of underlying molecular mechanisms of thermal tolerance in this species.

## Supplementary Information

Supplementary Information.

## Data Availability

The HiSeq reads of SLAF-seqwere under BioProject in the NCBI SRA database under accession PRJNA607371.
