# Supplementary material for: Identification of quantitative trait loci associated with upper temperature tolerance in turbot, Scophthalmus maximus
Source: Sci Rep. 2021 Nov 9;11:21920. doi: 10.1038/s41598-021-01062-3 (PMC8578632; doi:10.1038/s41598-021-01062-3)
Supplement: Supplementary file 1 — Supplementary Information. [file 41598_2021_1062_MOESM1_ESM.pdf]

Table S1 The primary sequence of some microsatellite loci (SSR) in Turbot

locus	repeat motif sequence	primer sequence	Tm	GeneBank accession no.
L12001	$(GT)_{16}$	R：CTGGCACTTGGATTCTGACC<br>F：GGTGCGGCAAGATGGAGT	60	
L12002	$(TG)_{19}$	R：CAAAGGCAGATGACAATCAAAC<br>F：ACTCGTAGCCGCTTTCGTC	60	
L12003	$(GT)_{36}$	R：ACACGGACGAAAGTGAAAAG<br>F：CCCGGACATTACAACCAAGA	59	
L12004	$(CAA)_{14}$	R：TTGCGTTCTCAAATCCAGTAG<br>F：GTATGGAGCGAATGAATGTTG	56	
L12005	$(CA)_5$	R：GCGAATACTGAAACAAGCG<br>F：CCCTACAGCAATCTCCCTC	55	
L12006	$(TG)_{26}$	R：GAAGAGTGGGGTGTATTGGTG<br>F：CGCTCTTGAGTTTGGCTTTTAG	57	
L12007	$(CA)_9$	R：CATACTGCTTTCAGGGGTGC<br>F：CGTGGTGATGACTGGTGGA	56	
L12008	$(AC)_{17}$	R：GACTGGCGATTTGATAAGGC<br>F：GAGTAATCACTTTTCGCTCTGC	56	
L12009	$(TG)_{18}$	R：GAGGTGAGGGACACTTGCG<br>F：CCAGTTGGCATCATGTATGG	56	
L12010	$(AC)_{17}$	R：GCTTACCGATTGTATTTTGGC<br>F：CATCGTTTTATCATTTTGAAGGT	57	
L12011	$(TG)_{17}$	R：GGGCCAAAGGAGGTCAAG<br>F：AGTCGTGCGGGAGGATG	60	
L12012	$(CA)_{24}$	R：GGGGATGAACAGGGAGATG<br>F：GGAGAACAGAGGTGAGGAGGC	61	
L12013	$(CA)_{16}$	R：ACACGTGTATGCACATTGTCC<br>F：GGAATGCGAAACTAACCTC	53	
L12014	$(GT)_{40}$	R：GGTCACATGATCTTTATCAGCAG<br>F：TGATGAGTCCTGAGTAAACAAC	53	
L12016	$(CT)_{12}CA(CT)_7$	R：AGCCCCGTTGGAAAATAAT<br>F：GGCGGCAGAAACTCAAGAT	55	
L12017	$(CA)_{22}CG(CA)_9$	R：GATACAGCAACCTCACGGAA<br>F：CCACCTTGTATGAGCGTTTG	59	
L12018	$(ATCTC)_6$	R：CCTGTGCCTCCCCGTGAT<br>F：CATCTACTGGACAAATGTGG	56	
L12019	$(GC)_4(AC)_{18}$	R：TGGGATTATTACTTGGGAGGTT<br>F：CAGGCTTTACACTTTATGCTTCC	57	
L12144	(TC)5	F:CGATAATTCAACAAGGGG<br>R:GGTTTTACAGTATCTGGCTG	51	

L12151	(GT)18	R: AGCGTCTGCTTGTTTCTG<br>F: GCTCTGCTCTACGCCTCA	51	
FF0916	(GT)14	F: TCTCTGTTCTACGGTGGGTT<br>R:AGATCAACACTCCTCTCACTAAAG	58	GU433244
FF0928	(CT)6	F:CTGTCTGACATGAAGGGATACC<br>R:ACAACGGTCATAATGCAAGTC	60	GU433255
FF0947	(ACA)5	F: ATTGTTGGAGCGGACTAAAA<br>R: AACTCGCAGGCAGGTATGT	60	GU433273
Sma-USC38	(TG)17	F: CAGTGTGCGTATGGAGAGTG<br>R: TGTGGGCAGTGAGAGTCAAG	54	DQ810838
Sma-USC81	(GT)78	F: CCAGAAGACAACAGGCAGA<br>R: TGAACTCAGGACACAAGAACG	57	DQ810880
Sma-USC85	(CA)28	F: ATCCGTCATTACTCCCAGCA<br>R: TCCGAGTCACTTCTCCCTCT	56	DQ810885
Sma-USC86	(GT)18	F: CACCAACAACGAAGTCCTCA<br>R: AGTCCTCCAAATCACATCCA	54	DQ810886
Sma-USC89	(TC)11	F: CCAGCTTGTAGCACATCAGC<br>R: GAAACGAACGGAAATGAACC	56	DQ810889
Sma-USC91	(GT)28	F: CCACTTCTTGGCTGGTTGTA<br>R: TCTTACAGCACCGAGCACCT	60	DQ810891
Sma-USC95	(GT)18	F: GTTCTGTGAGGGTGGCAAG<br>R: CCCTGTTTGGGATAATGTGG	55	DQ810895
Sma-USC96	(TG)13	F: GTGGGCAAATGTCAGGAACT<br>R: GCATTAAACACGGACCCATA	55	DQ810896
Sma-USC97	(CA)17	F: AACGCGCTCTATTCAACGAC<br>R: CAAACCAGTCACACCACCAC	58	DQ810897
Sma-USC98	(TG)21(GTGC)4	F: CCCACCCTCTTGACTTTCAG<br>R: CCAACCATGCAAACGTACAG	55	DQ810898
Sma-USC100	(TGA)7	F: ACTCGGTTTCCTCCGTCATA<br>R: ATTCGGCACTCAATTTACGC	55	DQ810900
Sma-USC102	(TGTA)10	F: ATGACCACACGCACCAGT<br>R: CTGACAAGCAATAGGAAACAG	52	DQ810902
Sma-USC108	(CATA)14	F: TAACCCTGAGGAGGAGCAT<br>R: TTACACATAATGAGGCTGAATG	55	DQ810908
Sma-USC109	(CTAT)7	F: CCGTCATTGGTAGAATCGT<br>R: GGCATCATTGGGTCTTTAT	55	DQ810909

Sma-USC113	(GATA)10	F: AAGGTTGCAGGACTCCCAAT<br>R: CCCACCAGGTCTCTCTGTCT	55	DQ810913
Sma-USC117	(GT)21	F: GACTAACACTGAGCGGACAT<br>R: TTGTCGTTCTGCCCATCT	55	DQ810917
Sma-USC279	(ACACT)9	F: TGTGTTTCTATGTCTCTCCTGT<br>R: CTGGATGAATATGGGCATCG	50	DQ811079
Sma-USC281	(AC)35	F: GCACGCACGCTCAACTTT<br>R: GCTATTCTTCTGAACACTGCAA	55	DQ811081
Sma-USC284	(GT)23	F: GTTTCTTTTCCAGTTTTTCAAT<br>R: TCTTTTCACCTCTCTTTTTCTC	52	DQ811084
Sma1-125INRA	(TAGA)11-(TG)4	F: CACACCTGACAAAGCTCAAC<br>R: GCTGAACATTTTCATGTTGATAG	55	No data
Sma3-129INRA	(GT)29	F: GCACTGCCTTTTCATTGG<br>R: CAGCTCTAGATTGTTTATCCC	55	No data
Sma5-111INRA	(TG)13	F: TCTACACTGCAGGTTGGG<br>R: CTGATTATGGGCTGGACG	52	No data
1/4AC18	(AC)18	F: AGAAAGGCTCGACCAGCTCC<br>R: TGATGGCTCATAGTGGCTAC	62	AF182088
3/9CA15	(CA)15	F: AGAGTGAAGAACGTACCTGC<br>R: CAATGGAGAGGCAGTATCGG	60	AF182091
4/4CA4/13	(AC)4G(CA)13	F: GCACTCATGCTGACATACAG<br>R: GACTCAGTGACTGATGAATC	64	AF182094
4/5CA22/6/2	(CA)22CT(CA)6 CT(CA)2	F: ACTCTGAGGGCAGAGGAAC<br>R: GCTGCTGGTAATCAGAAAGC	60	AF182095
H LJDLP23	(GT) 20	F: CTGGAACCTGAGCAGTGACA<br>R: CTGAATCACACACCGACTGG	56	FJ015047
H LJDLP33	(AC) 30	F: CGAGTCTCTGACCGGTTCTC<br>R: GACGTCTGAAATCCTCAGCAG	48	FJ015049
H LJDLP50	(GT) 35	F: GACGATCAGTTTGGGTCCAT<br>R: TGCTCAATTTTGACCCAGTG	51	FJ015051
Sma2432	(GTGC) 7	F: GGGTGTTTCCTGTGCAGATTGA<br>R: CTTCCCTCCGTCTACGCTCC	56	DQ454101

Table S2 Mean UTT and hour to loss of activity for each family in the thermal tolerance challenge

Family name	Number of hour to LOA		average of UTT	Sample size	Mean weight(g)
	The first	The last			
Mapping family	48	169	6.07×10^{3}	87	129.371
family1	62	185	7.76×10^{3}	89	137.846
family4	41	157	5.61×10^{3}	90	115.94
family5	47	183	7.92×10^{3}	90	125.782
family7	51	176	7.17×10^{3}	88	143.045
family10	38	182	7.13×10^{3}	87	139.428
family15	60	164	6.58×10^{3}	89	119.848
family18	58	179	7.72×10^{3}	90	126.94
family19	43	161	6.77×10^{3}	90	131.573

UTT=$\sum$j(Tj-Ta) where Tj is the experimental temperature at each hour, Ta is the acclimation temperature (14 ℃), and j represents each hour up to LOA (loss of activity) for each individual fish.

Table S3-1 The total map distances for the male maps

Linkage Group ID	Total Marker	Total Distance(cM)	AverageDistance(cM)	Max Gap
LG1	235	111.86	0.48	6.43
LG2	302	192.48	0.64	14.96
LG3	205	200.37	0.98	13.81
LG4	276	206.5	0.75	17.34
LG5	257	188.96	0.74	13.81
LG6	198	135.91	0.69	19.84
LG7	170	137.44	0.81	34.66
LG8	172	107.83	0.63	9.46
LG9	192	114.81	0.6	10.52
LG10	293	177.43	0.61	10.51
LG11	151	139.23	0.92	11.59
LG12	173	146.2	0.85	30.18
LG13	136	47.75	0.35	4.51
LG14	239	176.46	0.74	28.2
LG15	241	198.09	0.82	44.12
LG16	266	88.3	0.33	5.46
LG17	274	132.28	0.48	8.43
LG18	322	113.22	0.35	9.46
LG19	216	188.34	0.87	34.66
LG20	47	59.29	1.26	18.58
LG21	291	139.28	0.48	14.96
LG22	280	174.65	0.62	9.46
Total	4936	3176.68	0.65	44.12

Table S3-2 The total map distances for the female maps

Linkage Group ID	Total Marker	Total Distance(cM)	AverageDistance(cM)	Max Gap
LG1	210	296.73	1.41	21.14
LG2	318	161.3	0.51	6.43
LG3	183	166.86	0.91	12.69
LG4	290	257.7	0.89	13.81
LG5	300	182.75	0.61	13.81
LG6	198	258.39	1.3	19.84
LG7	141	142.97	1.01	11.59
LG8	248	216.65	0.87	13.81
LG9	204	229.51	1.13	12.69
LG10	245	201.28	0.82	10.52
LG11	130	168.42	1.3	13.81
LG12	251	208.84	0.83	10.52
LG13	162	173.13	1.07	18.58
LG14	184	149.86	0.81	8.43
LG15	241	184.85	0.77	22.48
LG16	269	176.47	0.66	9.46
LG17	247	128.27	0.52	8.43
LG18	321	133.45	0.42	5.46
LG19	248	201.33	0.81	19.84
LG20	71	52.6	0.74	12.69
LG21	351	155.72	0.44	9.46
LG22	249	205.98	0.83	13.81
Total	5061	4053.06	0.8	22.48

Table S3-3 The total map distances for the sex-averaged maps

Linkage Group ID	Total Marker	Total Distance(cM)	AverageDistance(cM)	Max Gap
LG1	367	204.3	0.56	12.83
LG2	511	184.71	0.36	12.06
LG3	321	184.49	0.57	7.16
LG4	463	232.1	0.5	9.07
LG5	476	186.74	0.39	11.08
LG6	345	201.37	0.58	10.11
LG7	267	140.2	0.53	14.45
LG8	359	166.28	0.46	6.22
LG9	333	172.16	0.52	8.81
LG10	453	191.11	0.42	6.67
LG11	228	159.69	0.7	10.02
LG12	364	178.42	0.49	7.64
LG13	279	111.31	0.4	10.36
LG14	363	164.48	0.45	14.53
LG15	408	191.47	0.47	25.79
LG16	432	132.82	0.31	4.93
LG17	383	131.61	0.34	6.48
LG18	542	124.69	0.23	4.73
LG19	367	195.71	0.53	13.44
LG20	96	55.94	0.58	6.73
LG21	514	147.5	0.29	7.48
LG22	442	191.19	0.43	9.03
Total	8313	3648.29	0.44	25.79

Table S4 The synteny pairs of Maroso's genome and the re-anchored genome.

synteny pairs	Chromosome ID of Maroso's genome	Position 1	Position 2	Chromosome ID of the re-anchored genome	Position 3	Position 4
1	CP026256.1	3686217	3958585	chr1	2306205	2578573
2	CP026256.1	4279029	4529485	chr1	2899017	3149473
3	CP026256.1	4700531	4765859	chr1	3320519	3385847
4	CP026256.1	4807756	4835262	chr1	3427744	3455250
5	CP026256.1	4849027	4852942	chr1	3469015	3472930
6	CP026256.1	4947863	5093533	chr1	3567851	3713521
7	CP026256.1	5229051	5352351	chr1	3849039	3972339
8	CP026256.1	5352449	5359154	chr1	3972437	3979142
9	CP026256.1	5463027	5532421	chr1	4083015	4152409
10	CP026256.1	5567291	5679158	chr1	4187279	4299146
11	CP026256.1	5694816	5701645	chr1	4314804	4321633
12	CP026256.1	5722335	5735105	chr1	4342323	4355093
13	CP026256.1	5801921	5805485	chr1	4421909	4425473
14	CP026256.1	5805665	5822074	chr1	4425653	4442062
15	CP026256.1	5822563	5839130	chr1	4442551	4459118
16	CP026256.1	5839922	5860593	chr1	4459910	4480581
17	CP026256.1	5860700	5909132	chr1	4480688	4529120
18	CP026256.1	5910005	5921784	chr1	4529993	4541772
19	CP026256.1	5924219	6015941	chr1	4544207	4635929
20	CP026256.1	6016294	6084348	chr1	4636282	4704336
21	CP026256.1	6084883	6134805	chr1	4704871	4754793
22	CP026256.1	6135859	6210909	chr1	4755847	4830897
23	CP026256.1	6211027	6343925	chr1	4831015	4963913
24	CP026256.1	6344035	6345471	chr1	4964023	4965459
25	CP026256.1	6345980	6373263	chr1	4965968	4993251

26	CP026256.1	6373634	6462369	chr1	4993622	5082357
27	CP026256.1	6462644	6474391	chr1	5082632	5094379
28	CP026256.1	6474651	6543908	chr1	5094639	5163896
29	CP026256.1	6544117	6568608	chr1	5164105	5188596
30	CP026256.1	6568887	6634724	chr1	5188875	5254712
31	CP026256.1	6634885	6684829	chr1	5254873	5304817
32	CP026256.1	6685167	6703926	chr1	5305155	5323914
33	CP026256.1	6704802	6743765	chr1	5324790	5363753
34	CP026256.1	6743863	6772551	chr1	5363851	5392539
35	CP026256.1	6772776	6814797	chr1	5392764	5434785
36	CP026256.1	6814885	6821024	chr1	5434873	5441012
37	CP026256.1	6821204	6848249	chr1	5441192	5468237
38	CP026256.1	6848361	6927375	chr1	5468349	5547363
39	CP026256.1	6927650	7000165	chr1	5547638	5620153
40	CP026256.1	7000844	7028305	chr1	5620832	5648293
41	CP026256.1	7028470	7031630	chr1	5648458	5651618
42	CP026256.1	7031760	7099524	chr1	5651748	5719512
43	CP026256.1	7099621	7115086	chr1	5719609	5735074
44	CP026256.1	7115173	7126489	chr1	5735161	5746477
45	CP026256.1	7126692	7280615	chr1	5746680	5900603
46	CP026256.1	7280809	7551058	chr1	5900797	6171046
47	CP026256.1	7551185	7591359	chr1	6171173	6211347
48	CP026256.1	7591451	7810796	chr1	6211439	6430784
49	CP026256.1	7810943	7852599	chr1	6430931	6472587
50	CP026256.1	7852757	8060483	chr1	6472745	6680471
51	CP026256.1	8061135	8069834	chr1	6681123	6689822
52	CP026256.1	8072671	8183638	chr1	6692659	6803626
53	CP026256.1	8183762	8189842	chr1	6803750	6809830
54	CP026256.1	8190007	8224845	chr1	6809995	6844833

55	CP026256.1	8225188	8279795	chr1	6845176	6899783
56	CP026256.1	8280053	8314693	chr1	6900041	6934681
57	CP026256.1	8314937	8330960	chr1	6934925	6950948
58	CP026256.1	8333178	8382631	chr1	6953166	7002619
59	CP026256.1	8382970	8679696	chr1	7002958	7299684
60	CP026256.1	8679828	8692377	chr1	7299816	7312365
61	CP026256.1	8692540	8714195	chr1	7312528	7334183
62	CP026256.1	8714590	8780071	chr1	7334578	7400059
63	CP026256.1	8780218	8783265	chr1	7400206	7403253
64	CP026256.1	8783390	8788408	chr1	7403378	7408396
65	CP026256.1	8788629	8810858	chr1	7408617	7430846
66	CP026256.1	8810977	9032572	chr1	7430965	7652560
67	CP026256.1	9032942	9058392	chr1	7652930	7678380
68	CP026256.1	9060041	9078116	chr1	7680029	7698104
69	CP026256.1	9078336	9159186	chr1	7698324	7779174
70	CP026256.1	9159311	9198725	chr1	7779299	7818713
71	CP026256.1	9198910	9200154	chr1	7818898	7820142
72	CP026256.1	9200508	9204333	chr1	7820496	7824321
73	CP026256.1	9204557	9233309	chr1	7824545	7853297
74	CP026256.1	9233548	9255773	chr1	7853536	7875761
75	CP026256.1	9255875	9268422	chr1	7875863	7888410
76	CP026256.1	9268730	9718355	chr1	7888718	8338343
77	CP026256.1	9718479	9729683	chr1	8338467	8349671
78	CP026256.1	9732128	9904114	chr1	8352116	8524102
79	CP026256.1	9904271	9992101	chr1	8524259	8612089
80	CP026256.1	9995854	10043045	chr1	8615842	8663033
81	CP026256.1	10043211	10085078	chr1	8663199	8705066
82	CP026256.1	10085352	10110335	chr1	8705340	8730323
83	CP026256.1	10111182	10143598	chr1	8731170	8763586

84	CP026256.1	10146749	10277812	chr1	8766737	8897800
85	CP026256.1	10278046	10376272	chr1	8898034	8996260
86	CP026256.1	10376518	10490912	chr1	8996506	9110900
87	CP026256.1	10491216	10685663	chr1	9111204	9305651
88	CP026256.1	10688676	10724328	chr1	9308664	9344316
89	CP026256.1	10724569	10754865	chr1	9344557	9374853
90	CP026256.1	10755010	10756937	chr1	9374998	9376925
91	CP026256.1	10757140	10787773	chr1	9377128	9407761
92	CP026256.1	10787959	10920753	chr1	9407947	9540741
93	CP026256.1	10921007	11154628	chr1	9540995	9774616
94	CP026256.1	11154855	11199856	chr1	9774843	9819844
95	CP026256.1	11199969	11213674	chr1	9819957	9833662
96	CP026256.1	11214646	11219569	chr1	9834634	9839557
97	CP026256.1	11219675	11248131	chr1	9839663	9868119
98	CP026256.1	11248251	11379845	chr1	9868239	9999833
99	CP026256.1	11379982	11456724	chr1	9999970	10076712
100	CP026256.1	11456848	11763324	chr1	10076836	10383312
101	CP026256.1	11763441	11799428	chr1	10383429	10419416
102	CP026256.1	11799573	11884707	chr1	10419561	10504695
103	CP026256.1	11885369	12009213	chr1	10505357	10629201
104	CP026256.1	12009616	12171958	chr1	10629604	10791946
105	CP026256.1	12172056	12172427	chr1	10792044	10792415
106	CP026256.1	12172829	12174379	chr1	10792817	10794367
107	CP026256.1	12174481	12207887	chr1	10794469	10827875
108	CP026256.1	12208040	12287275	chr1	10828028	10907263
109	CP026256.1	12287415	12549443	chr1	10907403	11169431
110	CP026256.1	12549540	12798786	chr1	11169528	11418774
111	CP026256.1	12799001	12917794	chr1	11418989	11537782
112	CP026256.1	12918155	13055762	chr1	11538143	11675750

113	CP026256.1	13055905	13503033	chr1	11675893	12123021
114	CP026256.1	13503280	13532670	chr1	12123268	12152658
115	CP026256.1	13533600	13593192	chr1	12153588	12213180
116	CP026256.1	13593471	13599948	chr1	12213459	12219936
117	CP026256.1	13600304	13691789	chr1	12220292	12311777
118	CP026256.1	13691908	13699640	chr1	12311896	12319628
119	CP026256.1	13699869	13802626	chr1	12319857	12422614
120	CP026256.1	13802768	13898887	chr1	12422756	12518875
121	CP026256.1	13899037	13924218	chr1	12519025	12544206
122	CP026256.1	13924384	14047263	chr1	12544372	12667251
123	CP026256.1	14047399	14071715	chr1	12667387	12691703
124	CP026256.1	14071907	14155121	chr1	12691895	12775109
125	CP026256.1	14155593	14232360	chr1	12775581	12852348
126	CP026256.1	14232449	14237770	chr1	12852437	12857758
127	CP026256.1	14238797	14263379	chr1	12858785	12883367
128	CP026256.1	14263501	14391074	chr1	12883489	13011062
129	CP026256.1	14391482	14507721	chr1	13011470	13127709
130	CP026256.1	14507833	14629595	chr1	13127821	13249583
131	CP026256.1	14629767	14696259	chr1	13249755	13316247
132	CP026256.1	14696572	14704514	chr1	13316560	13324502
133	CP026256.1	14704972	14722024	chr1	13324960	13342012
134	CP026256.1	14722206	14830218	chr1	13342194	13450206
135	CP026256.1	14830315	15158176	chr1	13450303	13778164
136	CP026256.1	15159439	15192057	chr1	13779427	13812045
137	CP026256.1	15192174	15221358	chr1	13812162	13841346
138	CP026256.1	15221683	15226180	chr1	13841671	13846168
139	CP026256.1	15226624	15256118	chr1	13846612	13876106
140	CP026256.1	15256231	15332191	chr1	13876219	13952179
141	CP026256.1	15334511	15357233	chr1	13954499	13977221

142	CP026256.1	15357371	15502646	chr1	13977359	14122634
143	CP026256.1	15502765	15509586	chr1	14122753	14129574
144	CP026256.1	15509756	15563162	chr1	14129744	14183150
145	CP026256.1	15563910	15567530	chr1	14183898	14187518
146	CP026256.1	15567783	15594665	chr1	14187771	14214653
147	CP026256.1	15594788	15611936	chr1	14214776	14231924
148	CP026256.1	15612426	15635633	chr1	14232414	14255621
149	CP026256.1	15635789	15647251	chr1	14255777	14267239
150	CP026256.1	15648993	15778425	chr1	14268981	14398413
151	CP026256.1	15781624	15800041	chr1	14401612	14420029
152	CP026256.1	15800170	15803595	chr1	14420158	14423583
153	CP026256.1	15804796	15873557	chr1	14424784	14493545
154	CP026256.1	15873842	15894417	chr1	14493830	14514405
155	CP026256.1	15894560	15909268	chr1	14514548	14529256
156	CP026256.1	15909366	15963419	chr1	14529354	14583407
157	CP026256.1	15964622	15992646	chr1	14584610	14612634
158	CP026256.1	15992935	15996090	chr1	14612923	14616078
159	CP026256.1	15997116	16035631	chr1	14617104	14655619
160	CP026256.1	16035833	16049266	chr1	14655821	14669254
161	CP026256.1	16049442	16074986	chr1	14669430	14694974
162	CP026256.1	16075230	16129364	chr1	14695218	14749352
163	CP026256.1	16129511	16136211	chr1	14749499	14756199
164	CP026256.1	16136378	16290839	chr1	14756366	14910827
165	CP026256.1	16290973	16293928	chr1	14910961	14913916
166	CP026256.1	16294582	16296232	chr1	14914570	14916220
167	CP026256.1	16296346	16300702	chr1	14916334	14920690
168	CP026256.1	16301895	16309769	chr1	14921883	14929757
169	CP026256.1	16312333	16313891	chr1	14932321	14933879
170	CP026256.1	16315193	16322696	chr1	14935181	14942684

171	CP026256.1	16325883	16343677	chr1	14945871	14963665
172	CP026256.1	16344052	16346210	chr1	14964040	14966198
173	CP026256.1	16346395	16351319	chr1	14966383	14971307
174	CP026256.1	16352447	16521877	chr1	14972435	15141865
175	CP026256.1	16522092	16530527	chr1	15142080	15150515
176	CP026256.1	16531544	16585085	chr1	15151532	15205073
177	CP026256.1	16585175	16618974	chr1	15205163	15238962
178	CP026256.1	16619261	16653400	chr1	15239249	15273388
179	CP026256.1	16653515	16698448	chr1	15273503	15318436
180	CP026256.1	16698776	16747963	chr1	15318764	15367951
181	CP026256.1	16748057	16769023	chr1	15368045	15389011
182	CP026256.1	16769378	16794947	chr1	15389366	15414935
183	CP026256.1	16795073	16864961	chr1	15415061	15484949
184	CP026256.1	16865155	16934783	chr1	15485143	15554771
185	CP026256.1	16934902	16944259	chr1	15554890	15564247
186	CP026256.1	16944415	16955620	chr1	15564403	15575608
187	CP026256.1	16957728	16974273	chr1	15577716	15594261
188	CP026256.1	16974492	16986691	chr1	15594480	15606679
189	CP026256.1	16986802	17001697	chr1	15606790	15621685
190	CP026256.1	17001965	17048595	chr1	15621953	15668583
191	CP026256.1	17048697	17067743	chr1	15668685	15687731
192	CP026256.1	17067914	17114457	chr1	15687902	15734445
193	CP026256.1	17114548	17170982	chr1	15734536	15790970
194	CP026256.1	17171317	17212090	chr1	15791305	15832078
195	CP026256.1	17212186	17216562	chr1	15832174	15836550
196	CP026256.1	17216842	17222684	chr1	15836830	15842672
197	CP026256.1	17222906	17234874	chr1	15842894	15854862
198	CP026256.1	17237863	17256409	chr1	15857851	15876397
199	CP026256.1	17256521	17261408	chr1	15876509	15881396

200	CP026256.1	17263004	17264771	chr1	15882992	15884759
201	CP026256.1	17265055	17286500	chr1	15885043	15906488
202	CP026256.1	17287448	17325338	chr1	15907436	15945326
203	CP026256.1	17325628	17364823	chr1	15945616	15984811
204	CP026256.1	17365042	17393816	chr1	15985030	16013804
205	CP026256.1	17394141	17396649	chr1	16014129	16016637
206	CP026256.1	17397817	17401101	chr1	16017805	16021089
207	CP026256.1	17401261	17403430	chr1	16021249	16023418
208	CP026256.1	17403576	17405686	chr1	16023564	16025674
209	CP026256.1	17405871	17409734	chr1	16025859	16029722
210	CP026256.1	17410895	17419683	chr1	16030883	16039671
211	CP026256.1	17419953	17432727	chr1	16039941	16052715
212	CP026256.1	17433022	17444692	chr1	16053010	16064680
213	CP026256.1	17444835	17465230	chr1	16064823	16085218
214	CP026256.1	17465330	17520689	chr1	16085318	16140677
215	CP026256.1	17521138	17526023	chr1	16141126	16146011
216	CP026256.1	17526332	17602553	chr1	16146320	16222541
217	CP026256.1	17602706	17609611	chr1	16222694	16229599
218	CP026256.1	17609697	17615710	chr1	16229685	16235698
219	CP026256.1	17618752	17623266	chr1	16238740	16243254
220	CP026256.1	17623383	17629816	chr1	16243371	16249804
221	CP026256.1	17629913	17633659	chr1	16249901	16253647
222	CP026256.1	17633747	17641396	chr1	16253735	16261384
223	CP026256.1	17641793	17665772	chr1	16261781	16285760
224	CP026256.1	17665896	17712340	chr1	16285884	16332328
225	CP026256.1	17712453	17755903	chr1	16332441	16375891
226	CP026256.1	17756123	17793109	chr1	16376111	16413097
227	CP026256.1	17793232	17806782	chr1	16413220	16426770
228	CP026256.1	17806903	17912062	chr1	16426891	16532050

229	CP026256.1	17912263	17922502	chr1	16532251	16542490
230	CP026256.1	17922606	17929515	chr1	16542594	16549503
231	CP026256.1	17929681	17932955	chr1	16549669	16552943
232	CP026256.1	17933210	17946643	chr1	16553198	16566631
233	CP026256.1	17946768	17970752	chr1	16566756	16590740
234	CP026256.1	17970860	17989013	chr1	16590848	16609001
235	CP026256.1	17991213	18047393	chr1	16611201	16667381
236	CP026256.1	18047527	18073396	chr1	16667515	16693384
237	CP026256.1	18075305	18138444	chr1	16695293	16758432
238	CP026256.1	18138580	18150456	chr1	16758568	16770444
239	CP026256.1	18155749	18160831	chr1	16770545	16775627
240	CP026256.1	18162518	18180541	chr1	16777314	16795337
241	CP026256.1	18180683	18181788	chr1	16795479	16796584
242	CP026256.1	18181903	18228667	chr1	16796699	16843463
243	CP026256.1	18229468	18239247	chr1	16844264	16854043
244	CP026256.1	18242506	18395263	chr1	16857302	17010059
245	CP026256.1	18403272	18407512	chr1	17018068	17022308
246	CP026256.1	18408517	18415541	chr1	17023313	17030337
247	CP026256.1	18415770	18419210	chr1	17030566	17034006
248	CP026256.1	18419523	18429741	chr1	17034319	17044537
249	CP026256.1	18430422	18439775	chr1	17045218	17054571
250	CP026256.1	18441170	18457287	chr1	17055966	17072083
251	CP026256.1	18459033	18464124	chr1	17073829	17078920
252	CP026256.1	18464394	18466675	chr1	17079190	17081471
253	CP026256.1	18467045	18470367	chr1	17081841	17085163
254	CP026256.1	18470598	18475323	chr1	17085394	17090119
255	CP026256.1	18478719	18480528	chr1	17093515	17095324
256	CP026256.1	18482402	18503691	chr1	17097198	17118487
257	CP026256.1	18504241	18510546	chr1	17119037	17125342

258	CP026256.1	18511026	18514168	chr1	17125822	17128964
259	CP026256.1	18514658	18516916	chr1	17129454	17131712
260	CP026256.1	18518104	18523231	chr1	17132900	17138027
261	CP026256.1	18523328	18528298	chr1	17138124	17143094
262	CP026256.1	18528406	18529223	chr1	17143202	17144019
263	CP026256.1	18531978	18602655	chr1	17146774	17217451
264	CP026256.1	18602761	18611655	chr1	17217557	17226451
265	CP026256.1	18611937	18647293	chr1	17226733	17262089
266	CP026256.1	18648600	18650257	chr1	17263396	17265053
267	CP026256.1	18655262	18660875	chr1	17270058	17275671
268	CP026256.1	18662767	18689006	chr1	17277563	17303802
269	CP026256.1	18689953	18696108	chr1	17304749	17310904
270	CP026256.1	18696325	18702326	chr1	17311121	17317122
271	CP026256.1	18702518	18704310	chr1	17317314	17319106
272	CP026256.1	18704402	18775368	chr1	17319198	17390164
273	CP026256.1	18775470	18794758	chr1	17390266	17409554
274	CP026256.1	18794985	18817575	chr1	17409781	17432371
275	CP026256.1	18817867	18849575	chr1	17432663	17464371
276	CP026256.1	18849922	18883929	chr1	17464718	17498725
277	CP026256.1	18884106	18908420	chr1	17498902	17523216
278	CP026256.1	18909177	18924862	chr1	17523973	17539658
279	CP026256.1	18925069	18971900	chr1	17539865	17586696
280	CP026256.1	18972006	18997390	chr1	17586802	17612186
281	CP026256.1	18997758	19012636	chr1	17612554	17627432
282	CP026256.1	19013196	19018698	chr1	17627992	17633494
283	CP026256.1	19022460	19046070	chr1	17637256	17660866
284	CP026256.1	19047034	19055561	chr1	17661830	17670357
285	CP026256.1	19057475	19059112	chr1	17672271	17673908
286	CP026256.1	19062157	19063457	chr1	17676953	17678253

287	CP026256.1	19063588	19071720	chr1	17678384	17686516
288	CP026256.1	19072133	19075377	chr1	17686929	17690173
289	CP026256.1	19075543	19086804	chr1	17690339	17701600
290	CP026256.1	19087131	19137348	chr1	17701927	17752144
291	CP026256.1	19137548	19195647	chr1	17752344	17810443
292	CP026256.1	19196803	19252228	chr1	17811599	17867024
293	CP026256.1	19252425	19315253	chr1	17867221	17930049
294	CP026256.1	19315375	19326096	chr1	17930171	17940892
295	CP026256.1	19326212	19355228	chr1	17941008	17970024
296	CP026256.1	19355330	19413470	chr1	17970126	18028266
297	CP026256.1	19414012	19415888	chr1	18028808	18030684
298	CP026256.1	19416399	19421142	chr1	18031195	18035938
299	CP026256.1	19421903	19431484	chr1	18036699	18046280
300	CP026256.1	19431592	19448606	chr1	18046388	18063402
301	CP026256.1	19448754	19450322	chr1	18063550	18065118
302	CP026256.1	19450798	19455254	chr1	18065594	18070050
303	CP026256.1	19457111	19458880	chr1	18071907	18073676
304	CP026256.1	19459744	19462790	chr1	18074540	18077586
305	CP026256.1	19463094	19466307	chr1	18077890	18081103
306	CP026256.1	19466528	19515227	chr1	18081324	18130023
307	CP026256.1	19517030	19523597	chr1	18131826	18138393
308	CP026256.1	19524280	19525652	chr1	18139076	18140448
309	CP026256.1	19525782	19528805	chr1	18140578	18143601
310	CP026256.1	19532044	19547949	chr1	18146840	18162745
311	CP026256.1	19548476	19552254	chr1	18163272	18167050
312	CP026256.1	19552353	19553610	chr1	18167149	18168406
313	CP026256.1	19554011	19558157	chr1	18168807	18172953
314	CP026256.1	19560070	19568499	chr1	18174866	18183295
315	CP026256.1	19568624	19571298	chr1	18183420	18186094

316	CP026256.1	19572816	19578570	chr1	18187612	18193366
317	CP026256.1	19578742	19587320	chr1	18193538	18202116
318	CP026256.1	19587725	19604997	chr1	18202521	18219793
319	CP026256.1	19607311	19625469	chr1	18222107	18240265
320	CP026256.1	19626544	19638786	chr1	18241340	18253582
321	CP026256.1	19639289	19652447	chr1	18254085	18267243
322	CP026256.1	19652590	19690698	chr1	18267386	18305494
323	CP026256.1	19690822	19692725	chr1	18305618	18307521
324	CP026256.1	19693807	19697360	chr1	18308603	18312156
325	CP026256.1	19697462	19700820	chr1	18312258	18315616
326	CP026256.1	19700944	19774887	chr1	18315740	18389683
327	CP026256.1	19775085	19778461	chr1	18389881	18393257
328	CP026256.1	19778787	19801975	chr1	18393583	18416771
329	CP026256.1	19802376	19808924	chr1	18417172	18423720
330	CP026256.1	19809963	19817640	chr1	18424759	18432436
331	CP026256.1	19820404	19830705	chr1	18435200	18445501
332	CP026256.1	19833263	19843177	chr1	18448059	18457973
333	CP026256.1	19844862	19919138	chr1	18459658	18533934
334	CP026256.1	19921684	19933826	chr1	18536480	18548622
335	CP026256.1	19934381	20008714	chr1	18549177	18623510
336	CP026256.1	20008843	20075890	chr1	18623639	18690686
337	CP026256.1	20078772	20085830	chr1	18693568	18700626
338	CP026256.1	20086137	20106057	chr1	18700933	18720853
339	CP026256.1	20106334	20110189	chr1	18721130	18724985
340	CP026256.1	20114560	20125645	chr1	18729356	18740441
341	CP026256.1	20127689	20129158	chr1	18742485	18743954
342	CP026256.1	20129284	20129752	chr1	18744080	18744548
343	CP026256.1	20129953	20133197	chr1	18744749	18747993
344	CP026256.1	20133442	20136539	chr1	18748238	18751335

345	CP026256.1	20137672	20142999	chr1	18752468	18757795
346	CP026256.1	20143371	20176162	chr1	18758167	18790958
347	CP026256.1	20176503	20188731	chr1	18791299	18803527
348	CP026256.1	20188864	20202303	chr1	18803660	18817099
349	CP026256.1	20202463	20221165	chr1	18817259	18835961
350	CP026256.1	20221256	20242586	chr1	18836052	18857382
351	CP026256.1	20242833	20251574	chr1	18857629	18866370
352	CP026256.1	20790434	20795691	chr1	19403577	19408834
353	CP026256.1	20796701	20800031	chr1	19409844	19413174
354	CP026256.1	20800828	20802613	chr1	19413971	19415756
355	CP026256.1	20805271	20808170	chr1	19418414	19421313
356	CP026256.1	20809569	20809930	chr1	19422712	19423073
357	CP026256.1	20810100	20817051	chr1	19423243	19430194
358	CP026256.1	20819110	20824448	chr1	19432253	19437591
359	CP026256.1	20825083	20827613	chr1	19438226	19440756
360	CP026256.1	20827887	20834252	chr1	19441030	19447395
361	CP026256.1	20834376	20860566	chr1	19447519	19473709
362	CP026256.1	20860716	20898453	chr1	19473859	19511596
363	CP026256.1	20898725	20905598	chr1	19511868	19518741
364	CP026256.1	20905724	20907755	chr1	19518867	19520898
365	CP026256.1	20908893	20910975	chr1	19522036	19524118
366	CP026256.1	20914468	20920749	chr1	19527611	19533892
367	CP026256.1	20922499	20962050	chr1	19535642	19575193
368	CP026256.1	20963138	20987732	chr1	19576281	19600875
369	CP026256.1	20987901	21005962	chr1	19601044	19619105
370	CP026256.1	21008288	21024322	chr1	19621431	19637465
371	CP026256.1	21024420	21033487	chr1	19637563	19646630
372	CP026256.1	21033848	21041510	chr1	19646991	19654653
373	CP026256.1	21042376	21074786	chr1	19655519	19687929

374	CP026256.1	21075132	21084608	chr1	19688275	19697751
375	CP026256.1	21087025	21103636	chr1	19700168	19716779
376	CP026256.1	21103835	21129311	chr1	19716978	19742454
377	CP026256.1	21130137	21133465	chr1	19743280	19746608
378	CP026256.1	21133628	21134091	chr1	19746771	19747234
379	CP026256.1	21134237	21169780	chr1	19747380	19782923
380	CP026256.1	21170050	21174270	chr1	19783193	19787413
381	CP026256.1	21174631	21182706	chr1	19787774	19795849
382	CP026256.1	21183084	21192711	chr1	19796227	19805854
383	CP026256.1	21197558	21230479	chr1	19810701	19843622
384	CP026256.1	21230961	21239081	chr1	19844104	19852224
385	CP026256.1	21239499	21242000	chr1	19852642	19855143
386	CP026256.1	21242393	21251146	chr1	19855536	19864289
387	CP026256.1	21251699	21260617	chr1	19864842	19873760
388	CP026256.1	21262098	21264513	chr1	19875241	19877656
389	CP026256.1	21265025	21269148	chr1	19878168	19882291
390	CP026256.1	21269383	21286862	chr1	19882526	19900005
391	CP026256.1	21287398	21288368	chr1	19900541	19901511
392	CP026256.1	21288503	21306399	chr1	19901646	19919542
393	CP026256.1	21307389	21311212	chr1	19920532	19924355
394	CP026256.1	21311606	21314636	chr1	19924749	19927779
395	CP026256.1	21316125	21323128	chr1	19929268	19936271
396	CP026256.1	21323634	21325988	chr1	19936777	19939131
397	CP026256.1	21327595	21345109	chr1	19940738	19958252
398	CP026256.1	21345221	21396972	chr1	19958364	20010115
399	CP026256.1	21397200	21422304	chr1	20010343	20035447
400	CP026256.1	21423129	21428053	chr1	20036272	20041196
401	CP026256.1	21428799	21455405	chr1	20041942	20068548
402	CP026256.1	21455669	21487985	chr1	20068812	20101128

403	CP026256.1	21488085	21495795	chr1	20101228	20108938
404	CP026256.1	21497097	21498889	chr1	20110240	20112032
405	CP026256.1	21499577	21501354	chr1	20112720	20114497
406	CP026256.1	21502434	21506050	chr1	20115577	20119193
407	CP026256.1	21506685	21508650	chr1	20119828	20121793
408	CP026256.1	21510128	21514608	chr1	20123271	20127751
409	CP026247.1	7337277	7450544	chr2	30216769	30216947
410	CP026247.1	7453487	7455484	chr2	30216589	30216771
411	CP026247.1	7495663	7519816	chr2	30213864	30214046
412	CP026247.1	7657738	7661674	chr2	28633568	28835988
413	CP026247.1	7661761	7762158	chr2	28569696	28633026
414	CP026247.1	7801437	7864079	chr2	28432380	28569556
415	CP026247.1	7924214	7932908	chr2	28312683	28432151
416	CP026247.1	8014432	8044207	chr2	28304959	28311668
417	CP026247.1	8176422	8179577	chr2	28288633	28304794
418	CP026247.1	8330443	8351537	chr2	28241539	28288534
419	CP026247.1	8500967	8548718	chr2	28170262	28241275
420	CP026247.1	8661771	8875887	chr2	28124009	28170051
421	CP026247.1	8943195	8945296	chr2	28082224	28123784
422	CP026247.1	8945388	8959840	chr2	28068417	28078471
423	CP026247.1	9284077	9294009	chr2	27896655	28068108
424	CP026247.1	9294100	9303293	chr2	27855986	27896415
425	CP026247.1	9396418	9760628	chr2	27647941	27855706
426	CP026247.1	9885876	9934762	chr2	27635752	27647791
427	CP026247.1	9966252	9972020	chr2	27393456	27635626
428	CP026247.1	10027884	10060467	chr2	27300196	27393281
429	CP026247.1	10148591	10182258	chr2	27250443	27300094
430	CP026247.1	10182347	10211927	chr2	27206643	27250351
431	CP026247.1	10246795	10343240	chr2	27135508	27206348

432	CP026247.1	10450556	10573569	chr2	27119965	27135367
433	CP026247.1	10886306	10910996	chr2	27051110	27119666
434	CP026247.1	11013523	11034333	chr2	26973857	27050868
435	CP026247.1	11092320	11130338	chr2	26940501	26973355
436	CP026247.1	11179711	11204993	chr2	26888148	26940362
437	CP026247.1	11281480	11294317	chr2	26880007	26888054
438	CP026247.1	11408243	11595774	chr2	26846666	26879916
439	CP026247.1	11642972	11680099	chr2	26688645	26846534
440	CP026247.1	11832048	11852890	chr2	26666022	26688018
441	CP026247.1	11889503	12068000	chr2	26638849	26665918
442	CP026247.1	12149473	12202557	chr2	26526748	26638680
443	CP026247.1	12382526	12495742	chr2	26499459	26525940
444	CP026247.1	12495841	12711299	chr2	26468332	26499215
445	CP026247.1	12748724	12780044	chr2	26455540	26468229
446	CP026247.1	13189831	13192454	chr2	26381317	26455424
447	CP026247.1	13268096	13282543	chr2	26323058	26380997
448	CP026247.1	13282631	13323054	chr2	26313682	26318534
449	CP026247.1	13359630	13397307	chr2	26157043	26313581
450	CP026247.1	13703694	13717318	chr2	26046419	26156834
451	CP026247.1	13853047	13868024	chr2	26040664	26044639
452	CP026247.1	13918728	13973250	chr2	26018213	26040504
453	CP026247.1	13973808	14081849	chr2	25909851	26018106
454	CP026247.1	14082090	14091973	chr2	25627434	25909719
455	CP026247.1	14092734	14211342	chr2	25502434	25627186
456	CP026247.1	14211666	14383425	chr2	25473819	25502328
457	CP026247.1	14383882	14420132	chr2	25440411	25473671
458	CP026247.1	14420276	14557465	chr2	25383569	25440299
459	CP026247.1	14557701	14608826	chr2	25309894	25383407
460	CP026247.1	14609282	14620529	chr2	25260132	25309773

461	CP026247.1	14621757	14671337	chr2	25128628	25259554
462	CP026247.1	14671443	14672759	chr2	24988826	25127489
463	CP026247.1	14672926	14674133	chr2	24981663	24987967
464	CP026247.1	14674427	14723206	chr2	24949951	24981562
465	CP026247.1	14723335	14735121	chr2	24942647	24948864
466	CP026247.1	14735692	14744609	chr2	24798964	24942529
467	CP026247.1	14745841	14784721	chr2	24635119	24798483
468	CP026247.1	14785019	14786875	chr2	24597008	24635031
469	CP026247.1	14787262	14824668	chr2	24547425	24596829
470	CP026247.1	14824893	15038463	chr2	24479548	24547283
471	CP026247.1	15038597	15219737	chr2	24256502	24479426
472	CP026247.1	15220335	15221870	chr2	24200126	24256312
473	CP026247.1	15222697	15254538	chr2	24177291	24200021
474	CP026247.1	15254651	15517310	chr2	24028656	24176125
475	CP026247.1	15518126	15519361	chr2	24026023	24028470
476	CP026247.1	15519629	16018756	chr2	23997541	24025873
477	CP026247.1	16018927	16051821	chr2	23990326	23997435
478	CP026247.1	16053721	16057479	chr2	23931118	23990175
479	CP026247.1	16058019	16134601	chr2	23908769	23930978
480	CP026247.1	16135153	16302075	chr2	23807010	23907743
481	CP026247.1	16302278	16316656	chr2	23781027	23806646
482	CP026247.1	16317214	16458625	chr2	23742584	23780786
483	CP026247.1	16458782	16621356	chr2	23494439	23725290
484	CP026247.1	16621476	16638701	chr2	23477559	23493093
485	CP026247.1	16638812	16663658	chr2	23444053	23477460
486	CP026247.1	16663833	16710245	chr2	23385544	23443950
487	CP026247.1	16710505	16721548	chr2	23296154	23385251
488	CP026247.1	16721737	17151503	chr2	23012793	23296005
489	CP026247.1	17151643	17178256	chr2	22976209	23012468

490	CP026247.1	17178612	17212891	chr2	22912986	22976087
491	CP026247.1	17213012	17234950	chr2	22737800	22912885
492	CP026247.1	17235082	17259884	chr2	22713596	22737640
493	CP026247.1	17272143	17287928	chr2	22435413	22713371
494	CP026247.1	17288029	17513045	chr2	22390312	22435006
495	CP026247.1	17513196	17557379	chr2	22361181	22390210
496	CP026247.1	17557580	17591304	chr2	22349957	22361055
497	CP026247.1	17591758	17609803	chr2	22315405	22349608
498	CP026247.1	17611012	17682386	chr2	22125027	22315306
499	CP026247.1	17683379	17732800	chr2	22071074	22124472
500	CP026247.1	17733034	17825139	chr2	21945545	22070937
501	CP026247.1	17825248	17832045	chr2	21844338	21944723
502	CP026247.1	17832155	17849280	chr2	21744119	21844236
503	CP026247.1	17871715	18142883	chr2	21687412	21743959
504	CP026247.1	18143464	18249044	chr2	21667448	21687161
505	CP026247.1	18249639	18312375	chr2	21664362	21666872
506	CP026247.1	18312608	18345741	chr2	21654190	21663039
507	CP026247.1	18345850	18358260	chr2	21454822	21654061
508	CP026247.1	18358384	18520530	chr2	21419161	21449595
509	CP026247.1	18520639	18655439	chr2	21390927	21418183
510	CP026247.1	18655548	18680863	chr2	21385417	21390820
511	CP026247.1	18682098	18899824	chr2	21310329	21385295
512	CP026247.1	18900803	18912190	chr2	21274577	21310160
513	CP026247.1	18913166	18945841	chr2	21167617	21274424
514	CP026247.1	18946072	18997695	chr2	21141531	21166768
515	CP026247.1	18998285	19137197	chr2	20929328	21141319
516	CP026247.1	19137312	19263529	chr2	20884852	20927440
517	CP026247.1	19263702	19306290	chr2	20758462	20884679
518	CP026247.1	19308178	19520169	chr2	20619435	20758347

519	CP026247.1	19520381	19545618	chr2	20567222	20618845
520	CP026247.1	19546467	19653274	chr2	20534316	20566991
521	CP026247.1	19653427	19689010	chr2	20521953	20533340
522	CP026247.1	19689179	19764145	chr2	20303248	20520974
523	CP026247.1	19764267	19769670	chr2	20276698	20302013
524	CP026247.1	19769777	19797033	chr2	20141789	20276589
525	CP026247.1	19798011	19828445	chr2	19979534	20141680
526	CP026247.1	19833672	20032911	chr2	19967000	19979410
527	CP026247.1	20033040	20041889	chr2	19933758	19966891
528	CP026247.1	20043212	20045722	chr2	19870789	19933525
529	CP026247.1	20046298	20066011	chr2	19764614	19870194
530	CP026247.1	20066262	20122809	chr2	19492865	19764033
531	CP026247.1	20122969	20223086	chr2	19453305	19470430
532	CP026247.1	20223188	20323573	chr2	19446398	19453195
533	CP026247.1	20324395	20449787	chr2	19354184	19446289
534	CP026247.1	20449924	20503322	chr2	19304529	19353950
535	CP026247.1	20503877	20694156	chr2	19232162	19303536
536	CP026247.1	20694255	20728458	chr2	19212908	19230953
537	CP026247.1	20728807	20739905	chr2	19178730	19212454
538	CP026247.1	20740031	20769060	chr2	19134346	19178529
539	CP026247.1	20769162	20813856	chr2	18909179	19134195
540	CP026247.1	20814263	21092221	chr2	18893293	18909078
541	CP026247.1	21092446	21116490	chr2	18868390	18893192
542	CP026247.1	21116650	21291735	chr2	18846320	18868258
543	CP026247.1	21291836	21354937	chr2	18811920	18846199
544	CP026247.1	21355059	21391318	chr2	18784951	18811564
545	CP026247.1	21391643	21674855	chr2	18355045	18784811
546	CP026247.1	21675004	21764101	chr2	18343813	18354856
547	CP026247.1	21764394	21822800	chr2	18297141	18343553

548	CP026247.1	21822903	21856310	chr2	18272120	18296966
549	CP026247.1	21856409	21871943	chr2	18254784	18272009
550	CP026247.1	21873289	22104140	chr2	18092090	18254664
551	CP026247.1	22121434	22159636	chr2	17950522	18091933
552	CP026247.1	22159877	22185496	chr2	17935586	17949964
553	CP026247.1	22185860	22286593	chr2	17768461	17935383
554	CP026247.1	22287619	22309828	chr2	17691327	17767909
555	CP026247.1	22309968	22369025	chr2	17687029	17690787
556	CP026247.1	22369176	22376285	chr2	17652235	17685129
557	CP026247.1	22376391	22404723	chr2	17152937	17652064
558	CP026247.1	22404873	22407320	chr2	17151434	17152669
559	CP026247.1	22407506	22554975	chr2	16887959	17150618
560	CP026247.1	22556141	22578871	chr2	16856005	16887846
561	CP026247.1	22578976	22635162	chr2	16853643	16855178
562	CP026247.1	22635352	22858276	chr2	16671905	16853045
563	CP026247.1	22858398	22926133	chr2	16458201	16671771
564	CP026247.1	22926275	22975679	chr2	16420570	16457976
565	CP026247.1	22975858	23013881	chr2	16418327	16420183
566	CP026247.1	23013969	23177333	chr2	16379149	16418029
567	CP026247.1	23177814	23321379	chr2	16369000	16377917
568	CP026247.1	23321497	23327714	chr2	16356643	16368429
569	CP026247.1	23328801	23360412	chr2	16307735	16356514
570	CP026247.1	23360513	23366817	chr2	16306234	16307441
571	CP026247.1	23367676	23506339	chr2	16304751	16306067
572	CP026247.1	23507478	23638404	chr2	16255065	16304645
573	CP026247.1	23638982	23688623	chr2	16242590	16253837
574	CP026247.1	23688744	23762257	chr2	16191009	16242134
575	CP026247.1	23762419	23819149	chr2	16053584	16190773
576	CP026247.1	23819261	23852521	chr2	16017190	16053440

577	CP026247.1	23852669	23881178	chr2	15844974	16016733
578	CP026247.1	23881284	24006036	chr2	15726042	15844650
579	CP026247.1	24006284	24288569	chr2	15715398	15725281
580	CP026247.1	24288701	24396956	chr2	15607116	15715157
581	CP026247.1	24397063	24419354	chr2	15552036	15606558
582	CP026247.1	24419514	24423489	chr2	15486355	15501332
583	CP026247.1	24425269	24535684	chr2	15337002	15350626
584	CP026247.1	24535893	24692431	chr2	14992938	15030615
585	CP026247.1	24692532	24697384	chr2	14915939	14956362
586	CP026247.1	24701908	24759847	chr2	14901404	14915851
587	CP026247.1	24760167	24834274	chr2	14823139	14825762
588	CP026247.1	24834390	24847079	chr2	14382032	14413352
589	CP026247.1	24847182	24878065	chr2	14129149	14344607
590	CP026247.1	24878309	24904790	chr2	14015834	14129050
591	CP026247.1	24905598	25017530	chr2	13782781	13835865
592	CP026247.1	25017699	25044768	chr2	13522811	13701308
593	CP026247.1	25044872	25066868	chr2	13465356	13486198
594	CP026247.1	25067495	25225384	chr2	13276280	13313407
595	CP026247.1	25225516	25258766	chr2	13041551	13229082
596	CP026247.1	25258857	25266904	chr2	12914788	12927625
597	CP026247.1	25266998	25319212	chr2	12813019	12838301
598	CP026247.1	25319351	25352205	chr2	12725628	12763646
599	CP026247.1	25352707	25429718	chr2	12646831	12667641
600	CP026247.1	25429960	25498516	chr2	12519614	12544304
601	CP026247.1	25498815	25514217	chr2	12083864	12206877
602	CP026247.1	25514358	25585198	chr2	11880103	11976548
603	CP026247.1	25585493	25629201	chr2	11815655	11845235
604	CP026247.1	25629293	25678944	chr2	11781899	11815566
605	CP026247.1	25679046	25772131	chr2	11661192	11693775

606	CP026247.1	25772306	26014476	chr2	11599560	11605328
607	CP026247.1	26014602	26026641	chr2	11519184	11568070
608	CP026247.1	26026791	26234556	chr2	11029726	11393936
609	CP026247.1	26234836	26275265	chr2	10927408	10936601
610	CP026247.1	26275505	26446958	chr2	10917385	10927317
611	CP026247.1	26447267	26457321	chr2	10578696	10593148
612	CP026247.1	26461074	26502634	chr2	10576503	10578604
613	CP026247.1	26502859	26548901	chr2	10295079	10509195
614	CP026247.1	26549112	26620125	chr2	10134275	10182026
615	CP026247.1	26620389	26667384	chr2	9963751	9984845
616	CP026247.1	26667483	26683644	chr2	9809730	9812885
617	CP026247.1	26683809	26690518	chr2	9647740	9677515
618	CP026247.1	26691533	26811001	chr2	9557522	9566216
619	CP026247.1	26811230	26948406	chr2	9434745	9497387
620	CP026247.1	26948546	27011876	chr2	9295069	9395466
621	CP026247.1	27012418	27214838	chr2	9291046	9294982
622	CP026247.1	28613467	28613649	chr2	9128971	9153124
623	CP026247.1	28616192	28616374	chr2	9086795	9088792
624	CP026247.1	28616728	28616906	chr2	8970585	9083852
625	CP026251.1	11988711	11994304	chr3	5774302	5768709
626	CP026251.1	11939196	11988478	chr3	5823817	5774535
627	CP026251.1	11859344	11938942	chr3	5903669	5824071
628	CP026251.1	11811318	11859053	chr3	5951695	5903960
629	CP026251.1	11732144	11810936	chr3	6030869	5952077
630	CP026251.1	11726159	11732011	chr3	6036854	6031002
631	CP026251.1	11719831	11725992	chr3	6043182	6037021
632	CP026251.1	11641781	11719722	chr3	6121232	6043291
633	CP026251.1	11584287	11641413	chr3	6178726	6121600
634	CP026251.1	11559472	11584191	chr3	6203541	6178822

635	CP026251.1	11491639	11558814	chr3	6271374	6204199
636	CP026251.1	11389975	11491524	chr3	6373038	6271489
637	CP026251.1	11305826	11389839	chr3	6457187	6373174
638	CP026251.1	11235527	11305655	chr3	6527486	6457358
639	CP026251.1	11160044	11235143	chr3	6602969	6527870
640	CP026251.1	11078731	11158964	chr3	6684282	6604049
641	CP026251.1	11060693	11078093	chr3	6702320	6684920
642	CP026251.1	11056065	11057173	chr3	6706948	6705840
643	CP026251.1	10868563	11052463	chr3	6894450	6710550
644	CP026251.1	10774756	10868430	chr3	6988257	6894583
645	CP026251.1	10763894	10773255	chr3	6999119	6989758
646	CP026251.1	10597019	10763312	chr3	7165994	6999701
647	CP026251.1	10579398	10596814	chr3	7183615	7166199
648	CP026251.1	10516297	10574199	chr3	7246716	7188814
649	CP026251.1	10505292	10516198	chr3	7257721	7246815
650	CP026251.1	10470276	10504278	chr3	7292737	7258735
651	CP026251.1	10382335	10469877	chr3	7380678	7293136
652	CP026251.1	10376312	10382137	chr3	7386701	7380876
653	CP026251.1	10250873	10376083	chr3	7512140	7386930
654	CP026251.1	10183439	10250566	chr3	7579574	7512447
655	CP026251.1	10097910	10183263	chr3	7665103	7579750
656	CP026251.1	10057310	10097721	chr3	7705703	7665292
657	CP026251.1	10042803	10057032	chr3	7720210	7705981
658	CP026251.1	10036986	10041812	chr3	7726027	7721201
659	CP026251.1	10018627	10036777	chr3	7744386	7726236
660	CP026251.1	9954675	10018245	chr3	7808338	7744768
661	CP026251.1	9896609	9954445	chr3	7866404	7808568
662	CP026251.1	9894195	9896173	chr3	7868818	7866840
663	CP026251.1	9888832	9890056	chr3	7874181	7872957

664	CP026251.1	9884334	9887062	chr3	7878679	7875951
665	CP026251.1	9844214	9883121	chr3	7918799	7879892
666	CP026251.1	9823627	9844080	chr3	7939386	7918933
667	CP026251.1	9793908	9823356	chr3	7969105	7939657
668	CP026251.1	9777574	9793770	chr3	7985439	7969243
669	CP026251.1	9723710	9776900	chr3	8039303	7986113
670	CP026251.1	9707381	9723616	chr3	8055632	8039397
671	CP026251.1	9662500	9706896	chr3	8100513	8056117
672	CP026251.1	9642497	9662413	chr3	8120516	8100600
673	CP026251.1	9602154	9642370	chr3	8160859	8120643
674	CP026251.1	9532257	9602010	chr3	8230756	8161003
675	CP026251.1	9525343	9532119	chr3	8237670	8230894
676	CP026251.1	9517494	9525189	chr3	8245519	8237824
677	CP026251.1	9484290	9517392	chr3	8278723	8245621
678	CP026251.1	9475618	9484051	chr3	8287395	8278962
679	CP026251.1	9458435	9475459	chr3	8304578	8287554
680	CP026251.1	9275327	9458300	chr3	8487686	8304713
681	CP026251.1	9269371	9275198	chr3	8493642	8487815
682	CP026251.1	9197864	9269212	chr3	8565149	8493801
683	CP026251.1	9171791	9197067	chr3	8591222	8565946
684	CP026251.1	9170978	9171646	chr3	8592035	8591367
685	CP026251.1	9133688	9170822	chr3	8629325	8592191
686	CP026251.1	8930076	9133537	chr3	8832937	8629476
687	CP026251.1	8924394	8929860	chr3	8838619	8833153
688	CP026251.1	8919572	8924272	chr3	8843441	8838741
689	CP026251.1	8707594	8919453	chr3	9055419	8843560
690	CP026251.1	8617485	8707460	chr3	9145528	9055553
691	CP026251.1	8598123	8617248	chr3	9164890	9145765
692	CP026251.1	8377516	8598019	chr3	9385497	9164994

693	CP026251.1	8047214	8377405	chr3	9715799	9385608
694	CP026251.1	7933599	8046856	chr3	9829414	9716157
695	CP026251.1	7878450	7933126	chr3	9884563	9829887
696	CP026251.1	7784410	7878321	chr3	9978603	9884692
697	CP026251.1	7428451	7784203	chr3	10334562	9978810
698	CP026251.1	7185758	7428291	chr3	10577255	10334722
699	CP026251.1	7162772	7185628	chr3	10600241	10577385
700	CP026251.1	7109331	7162651	chr3	10653682	10600362
701	CP026251.1	6343183	7109158	chr3	11419830	10653855
702	CP026251.1	6170922	6343068	chr3	11592091	11419945
703	CP026251.1	5981977	6170823	chr3	11781036	11592190
704	CP026251.1	5934892	5981771	chr3	11828121	11781242
705	CP026251.1	5900053	5934736	chr3	11862960	11828277
706	CP026251.1	5485269	5899931	chr3	12277744	11863082
707	CP026251.1	5319907	5484875	chr3	12443106	12278138
708	CP026251.1	5230681	5313878	chr3	12532332	12449135
709	CP026245.1	92526	610082	chr4	23823443	23825068
710	CP026245.1	610183	611515	chr4	23809056	23820553
711	CP026245.1	620784	712418	chr4	23796917	23808351
712	CP026245.1	712618	731295	chr4	23778039	23795329
713	CP026245.1	736126	815361	chr4	23745234	23777453
714	CP026245.1	818764	821896	chr4	23743608	23745102
715	CP026245.1	832294	854920	chr4	23702180	23743362
716	CP026245.1	908884	912205	chr4	23688797	23701748
717	CP026245.1	913693	955015	chr4	23654184	23688149
718	CP026245.1	955280	1062373	chr4	23639447	23653693
719	CP026245.1	1062465	1276494	chr4	23637597	23638845
720	CP026245.1	1276647	1528370	chr4	23621875	23637390
721	CP026245.1	1528529	1850154	chr4	23616619	23621774

722	CP026245.1	1851069	1978256	chr4	23600808	23613489
723	CP026245.1	1978625	2015940	chr4	23599051	23600473
724	CP026245.1	2016095	2401148	chr4	23591509	23597049
725	CP026245.1	2401264	2426903	chr4	23587682	23589573
726	CP026245.1	2427297	2598780	chr4	23585292	23586966
727	CP026245.1	2599399	2610747	chr4	23576870	23583634
728	CP026245.1	2610903	3082603	chr4	23556242	23576625
729	CP026245.1	3082871	3153473	chr4	23553469	23554856
730	CP026245.1	3153901	3172346	chr4	23550718	23552344
731	CP026245.1	3173965	3296082	chr4	23541235	23549717
732	CP026245.1	3296216	3351494	chr4	23530228	23540958
733	CP026245.1	3351720	3387330	chr4	23522506	23524529
734	CP026245.1	3387674	3433471	chr4	23517738	23519313
735	CP026245.1	3433662	3459584	chr4	23505823	23516887
736	CP026245.1	3459693	3631880	chr4	23490679	23504810
737	CP026245.1	3632895	3731387	chr4	23488107	23490158
738	CP026245.1	3731967	3765834	chr4	23480925	23486851
739	CP026245.1	3766133	3767592	chr4	23477281	23479102
740	CP026245.1	3771180	3780793	chr4	23474694	23476580
741	CP026245.1	3781042	3784504	chr4	23467045	23474570
742	CP026245.1	3784697	3872081	chr4	23455506	23466387
743	CP026245.1	3872286	3890931	chr4	23439891	23455329
744	CP026245.1	3891160	3894335	chr4	23424837	23438882
745	CP026245.1	3896466	3898957	chr4	23421989	23423594
746	CP026245.1	3899253	3918995	chr4	23415062	23420090
747	CP026245.1	3919561	3948680	chr4	23405397	23414460
748	CP026245.1	3948908	3957408	chr4	23322063	23323919
749	CP026245.1	3957600	3969307	chr4	23307495	23321885
750	CP026245.1	3969553	3978377	chr4	23292130	23307162

751	CP026245.1	3981218	3991692	chr4	23259939	23291533
752	CP026245.1	3991796	4016086	chr4	23240418	23258404
753	CP026245.1	4016214	4027330	chr4	23229395	23237372
754	CP026245.1	4027655	4070185	chr4	23224217	23228954
755	CP026245.1	4070275	4102357	chr4	23219940	23224080
756	CP026245.1	4102512	4160831	chr4	23215264	23216539
757	CP026245.1	4161038	4184786	chr4	23181043	23215163
758	CP026245.1	4184981	4203795	chr4	23170625	23178723
759	CP026245.1	4249672	4252279	chr4	23164486	23168492
760	CP026245.1	4253879	4309643	chr4	23153708	23163609
761	CP026245.1	4312403	4315517	chr4	23152226	23153501
762	CP026245.1	4318062	4394762	chr4	23149664	23151177
763	CP026245.1	4394895	4462407	chr4	23145518	23149351
764	CP026245.1	4462699	4526671	chr4	23144184	23145417
765	CP026245.1	4526976	4536659	chr4	23134627	23142991
766	CP026245.1	4537047	4540555	chr4	23125144	23134007
767	CP026245.1	4540824	4546642	chr4	23123561	23124888
768	CP026245.1	4546757	4553946	chr4	23117045	23121001
769	CP026245.1	4554267	4582509	chr4	23113198	23116944
770	CP026245.1	4582695	4782687	chr4	23102379	23110285
771	CP026245.1	4783200	4932281	chr4	23096028	23102041
772	CP026245.1	4932460	4978985	chr4	23085455	23094413
773	CP026245.1	4979815	5000594	chr4	23075951	23084932
774	CP026245.1	5000844	5032060	chr4	23066929	23074012
775	CP026245.1	5036140	5109031	chr4	23064032	23065798
776	CP026245.1	5109174	5140587	chr4	23055086	23062591
777	CP026245.1	5140679	5169295	chr4	23051692	23053128
778	CP026245.1	5169685	5212365	chr4	23041683	23047631
779	CP026245.1	5212833	5294691	chr4	23009295	23041519

780	CP026245.1	5295742	5326894	chr4	23007712	23009183
781	CP026245.1	5327441	5330708	chr4	23003799	23005377
782	CP026245.1	5330841	5424388	chr4	22977137	23003674
783	CP026245.1	5424476	5490615	chr4	22974324	22976919
784	CP026245.1	5490704	5519051	chr4	22967306	22973209
785	CP026245.1	5520415	5583566	chr4	22964915	22966684
786	CP026245.1	5584011	5588787	chr4	22961470	22964714
787	CP026245.1	5589135	5852777	chr4	22957985	22959550
788	CP026245.1	5852911	5868524	chr4	22954605	22957859
789	CP026245.1	5868665	5871694	chr4	22951218	22953560
790	CP026245.1	5871983	5879791	chr4	22948710	22950445
791	CP026245.1	5879961	5889464	chr4	22942176	22944935
792	CP026245.1	5890834	5946753	chr4	22934687	22942074
793	CP026245.1	5946998	5972778	chr4	22916416	22934304
794	CP026245.1	5972962	5977484	chr4	22910708	22915143
795	CP026245.1	5977650	5986131	chr4	22906078	22910440
796	CP026245.1	5986220	6018225	chr4	22899185	22904503
797	CP026245.1	6018442	6074582	chr4	22887021	22896335
798	CP026245.1	6074735	6112685	chr4	22878105	22886883
799	CP026245.1	6112795	6116351	chr4	22866703	22877872
800	CP026245.1	6116439	6142769	chr4	22860725	22863545
801	CP026245.1	6143303	6170744	chr4	22850645	22860015
802	CP026245.1	6170846	6199853	chr4	22842185	22850167
803	CP026245.1	6200408	6294960	chr4	22839130	22841735
804	CP026245.1	6295195	6308999	chr4	22827461	22838765
805	CP026245.1	6309156	6333763	chr4	22812707	22825189
806	CP026245.1	6334450	6341640	chr4	22809685	22810995
807	CP026245.1	6342036	6352480	chr4	22755117	22808835
808	CP026245.1	6352773	6459101	chr4	22735136	22752665

809	CP026245.1	6459512	6571698	chr4	22721883	22734658
810	CP026245.1	6571811	6596019	chr4	22674427	22721486
811	CP026245.1	6596140	6683654	chr4	22662730	22673540
812	CP026245.1	6683961	6689824	chr4	22633319	22661771
813	CP026245.1	6690911	6722910	chr4	22623993	22633097
814	CP026245.1	6723017	6726630	chr4	22622045	22623825
815	CP026245.1	6728052	6740255	chr4	22602050	22621537
816	CP026245.1	6740369	6764830	chr4	22597187	22601917
817	CP026245.1	6766355	7029747	chr4	22582165	22597086
818	CP026245.1	7030078	7043109	chr4	22562278	22581050
819	CP026245.1	7043524	7244921	chr4	22544664	22561817
820	CP026245.1	7245321	7274701	chr4	22535980	22544534
821	CP026245.1	7275054	7372528	chr4	22529278	22532397
822	CP026245.1	7372723	7398014	chr4	22525677	22528264
823	CP026245.1	7398232	7697965	chr4	22518802	22522743
824	CP026245.1	7698193	7875195	chr4	22514748	22518391
825	CP026245.1	7875338	8103201	chr4	22511233	22514457
826	CP026245.1	8103338	8204478	chr4	22502269	22511108
827	CP026245.1	8212229	8252488	chr4	22487759	22498879
828	CP026245.1	8252608	8273855	chr4	22486030	22487551
829	CP026245.1	8273992	8510966	chr4	22479516	22485929
830	CP026245.1	8511127	8525229	chr4	22470099	22478605
831	CP026245.1	8525478	8700218	chr4	22455496	22469590
832	CP026245.1	8700309	8792616	chr4	22451478	22455024
833	CP026245.1	8792712	8954275	chr4	22346472	22450109
834	CP026245.1	8954398	8983605	chr4	22332765	22346294
835	CP026245.1	9001940	9190980	chr4	22306020	22331631
836	CP026245.1	9213815	9252107	chr4	22295692	22305833
837	CP026245.1	9252242	9331618	chr4	22280318	22295116

838	CP026245.1	9332561	9845241	chr4	22274371	22279803
839	CP026245.1	9845334	9884090	chr4	22272087	22273661
840	CP026245.1	9884206	10025945	chr4	22255634	22268124
841	CP026245.1	10026085	10552816	chr4	22251864	22255482
842	CP026245.1	10552982	10635251	chr4	22247946	22251626
843	CP026245.1	10635569	10650504	chr4	22239237	22247660
844	CP026245.1	10650767	10866835	chr4	22237314	22239142
845	CP026245.1	11026928	11158281	chr4	22233620	22236222
846	CP026245.1	11158385	11198962	chr4	22223927	22231868
847	CP026245.1	11199082	11245681	chr4	22208016	22223693
848	CP026245.1	11245930	11285855	chr4	22199861	22207909
849	CP026245.1	11286256	11317755	chr4	22138246	22199739
850	CP026245.1	11317893	11319628	chr4	22135370	22138015
851	CP026245.1	11319782	11340510	chr4	22126403	22131853
852	CP026245.1	11340862	11354949	chr4	22121560	22126065
853	CP026245.1	11355201	11407498	chr4	22113336	22121137
854	CP026245.1	11407601	11447981	chr4	22096684	22112578
855	CP026245.1	11448142	11480366	chr4	22093227	22096312
856	CP026245.1	11480533	11493583	chr4	22083697	22092968
857	CP026245.1	11493986	11498665	chr4	22080465	22083179
858	CP026245.1	11498899	11589349	chr4	22073269	22080077
859	CP026245.1	11589814	11613921	chr4	22059436	22072567
860	CP026245.1	11614029	11883738	chr4	21988071	22059155
861	CP026245.1	11883890	11906990	chr4	21926885	21987399
862	CP026245.1	11907416	11913614	chr4	21924031	21926770
863	CP026245.1	11913706	11960892	chr4	21914560	21923172
864	CP026245.1	11961018	11972695	chr4	21880463	21914328
865	CP026245.1	11972865	12046651	chr4	21874869	21880273
866	CP026245.1	12046760	12262798	chr4	21872526	21874773

867	CP026245.1	12263005	12370148	chr4	21856885	21872111
868	CP026245.1	12370247	12537028	chr4	21839364	21856428
869	CP026245.1	12537517	12605063	chr4	21830982	21839231
870	CP026245.1	12605204	12646626	chr4	21807777	21829797
871	CP026245.1	12646800	12806305	chr4	21799395	21806339
872	CP026245.1	12806501	12823031	chr4	21786771	21798288
873	CP026245.1	12823224	12860417	chr4	21783649	21786613
874	CP026245.1	12860737	13016487	chr4	21780857	21782063
875	CP026245.1	13016733	13019017	chr4	21777026	21780755
876	CP026245.1	13019532	13053455	chr4	21763533	21775850
877	CP026245.1	13053564	13067725	chr4	21760876	21763078
878	CP026245.1	13067947	13080626	chr4	21758722	21760338
879	CP026245.1	13080834	13141700	chr4	21756284	21757867
880	CP026245.1	13141949	13163578	chr4	21741293	21755776
881	CP026245.1	13163678	13220118	chr4	21721127	21739585
882	CP026245.1	13220304	13266823	chr4	21708823	21717730
883	CP026245.1	13266912	13577781	chr4	21701825	21707630
884	CP026245.1	13577873	13604624	chr4	21698680	21699823
885	CP026245.1	13604750	13671941	chr4	21691905	21695120
886	CP026245.1	13672249	13759681	chr4	21687842	21691045
887	CP026245.1	13759791	13974432	chr4	21681721	21687137
888	CP026245.1	13974610	13992902	chr4	21667314	21681402
889	CP026245.1	13994233	14102789	chr4	21656993	21664534
890	CP026245.1	14104681	14140028	chr4	21651609	21656573
891	CP026245.1	14140131	14176020	chr4	21644445	21651267
892	CP026245.1	14176154	14215885	chr4	21642762	21644197
893	CP026245.1	14216094	14242948	chr4	21635154	21640860
894	CP026245.1	14243114	14247581	chr4	21617726	21634317
895	CP026245.1	14247859	14262425	chr4	21606404	21616856

896	CP026245.1	14262602	14264619	chr4	21590765	21606228
897	CP026245.1	14264724	14279146	chr4	21577967	21590660
898	CP026245.1	14279312	14287951	chr4	21566855	21577515
899	CP026245.1	14288081	14602441	chr4	21561839	21566537
900	CP026245.1	14602575	14608018	chr4	21545897	21560344
901	CP026245.1	14608160	14612791	chr4	21541346	21544043
902	CP026245.1	14612892	14620751	chr4	21536522	21541130
903	CP026245.1	14621022	14649949	chr4	21533411	21536238
904	CP026245.1	14651910	14720291	chr4	21530782	21533269
905	CP026245.1	14720408	14732115	chr4	21522361	21528777
906	CP026245.1	14732289	14751167	chr4	21513831	21521388
907	CP026245.1	14751329	14835700	chr4	21482079	21513697
908	CP026245.1	14835914	14899292	chr4	21472457	21481758
909	CP026245.1	14899397	15018490	chr4	21467999	21470213
910	CP026245.1	15018720	15125938	chr4	21448615	21467336
911	CP026245.1	15126040	15311172	chr4	21436731	21448278
912	CP026245.1	15311350	15382736	chr4	21399695	21435278
913	CP026245.1	15383536	15386227	chr4	21391203	21399535
914	CP026245.1	15386386	15456184	chr4	21388967	21390259
915	CP026245.1	15457474	15461249	chr4	21384831	21388843
916	CP026245.1	15461739	15543879	chr4	21378741	21384365
917	CP026245.1	15545099	15605279	chr4	21375300	21378598
918	CP026245.1	15605511	15613894	chr4	21362711	21373882
919	CP026245.1	15614009	15667854	chr4	21343856	21355361
920	CP026245.1	15668044	15809745	chr4	21326640	21342845
921	CP026245.1	15809849	15833193	chr4	21318383	21326244
922	CP026245.1	15833353	15865218	chr4	21305982	21317944
923	CP026245.1	15865366	15880890	chr4	21290850	21301115
924	CP026245.1	15881382	15906332	chr4	21286358	21289151

925	CP026245.1	15906439	15915691	chr4	21231975	21282543
926	CP026245.1	15916620	15957059	chr4	21177181	21231802
927	CP026245.1	15957161	15982432	chr4	21170782	21175737
928	CP026245.1	15983784	15994142	chr4	21160529	21170602
929	CP026245.1	15994388	16023023	chr4	21155204	21159944
930	CP026245.1	16023288	16165566	chr4	21144399	21153817
931	CP026245.1	16165684	16216290	chr4	21135430	21143664
932	CP026245.1	16216391	16321496	chr4	21130555	21134162
933	CP026245.1	16321596	16417459	chr4	21125410	21128575
934	CP026245.1	16417877	16439496	chr4	21116414	21124668
935	CP026245.1	16439597	16479156	chr4	21096228	21115379
936	CP026245.1	16479247	16545771	chr4	21089594	21094203
937	CP026245.1	16546274	16561682	chr4	21082528	21089462
938	CP026245.1	16561945	16638941	chr4	21080134	21082349
939	CP026245.1	16639092	16722816	chr4	21072134	21079928
940	CP026245.1	16723078	16736240	chr4	21068354	21071874
941	CP026245.1	16736355	16778963	chr4	21063625	21064834
942	CP026245.1	16779051	16899426	chr4	21055662	21061473
943	CP026245.1	16901757	16947632	chr4	21049907	21055413
944	CP026245.1	16947789	16960085	chr4	21045866	21049801
945	CP026245.1	16960424	16995870	chr4	21042652	21045736
946	CP026245.1	16997114	17021908	chr4	21032393	21042135
947	CP026245.1	17022115	17044435	chr4	20994271	21032153
948	CP026245.1	17044550	17123189	chr4	20980600	20993979
949	CP026245.1	17123531	17258622	chr4	20960361	20979743
950	CP026245.1	17259074	17349717	chr4	20938900	20960146
951	CP026245.1	17349927	17384886	chr4	20928691	20938796
952	CP026245.1	17386186	17402261	chr4	20922291	20928553
953	CP026245.1	17402474	17569848	chr4	20909215	20922017

954	CP026245.1	17570401	17624386	chr4	20872576	20908151
955	CP026245.1	17624773	17678374	chr4	20867570	20872438
956	CP026245.1	17679029	17683576	chr4	20796187	20866646
957	CP026245.1	17683737	17736597	chr4	20775761	20792917
958	CP026245.1	17737724	17751185	chr4	20766177	20775353
959	CP026245.1	17751364	17784002	chr4	20714437	20764563
960	CP026245.1	17784116	17836513	chr4	20642191	20714044
961	CP026245.1	17836723	17881276	chr4	20603413	20640977
962	CP026245.1	17881484	17884836	chr4	20544182	20602114
963	CP026245.1	17885108	17904990	chr4	20536388	20543768
964	CP026245.1	17905113	17972917	chr4	20526204	20536208
965	CP026245.1	17973123	17974986	chr4	20524359	20525898
966	CP026245.1	17976243	18020965	chr4	20518685	20523744
967	CP026245.1	18021833	18025498	chr4	20499396	20518299
968	CP026245.1	18025702	18033650	chr4	20471827	20499010
969	CP026245.1	18035550	18080685	chr4	20462786	20471411
970	CP026245.1	18080973	18116248	chr4	20407850	20462585
971	CP026245.1	18116569	18172650	chr4	20387253	20407383
972	CP026245.1	18173101	18209728	chr4	20379222	20386945
973	CP026245.1	18209967	18228383	chr4	20337601	20379075
974	CP026245.1	18228476	18231143	chr4	20306152	20337357
975	CP026245.1	18231388	18248577	chr4	20302882	20305368
976	CP026245.1	18248748	18314348	chr4	20296937	20302678
977	CP026245.1	18314472	18440885	chr4	20255712	20296817
978	CP026245.1	18440986	18517889	chr4	20215671	20255477
979	CP026245.1	18518076	18533652	chr4	20206145	20215561
980	CP026245.1	18533825	18559695	chr4	20203622	20206037
981	CP026245.1	18559782	18567973	chr4	20181526	20203475
982	CP026245.1	18568152	18596669	chr4	20175536	20179852

983	CP026245.1	18596842	18601324	chr4	20162701	20175035
984	CP026245.1	18603086	18611147	chr4	20157745	20161735
985	CP026245.1	18611807	18613282	chr4	20151298	20157629
986	CP026245.1	18613488	18659896	chr4	20136510	20151039
987	CP026245.1	18662525	18687201	chr4	20130868	20136081
988	CP026245.1	18687289	18689754	chr4	20128510	20130700
989	CP026245.1	18690042	18706593	chr4	20119892	20128268
990	CP026245.1	18706924	18710001	chr4	20115892	20119610
991	CP026245.1	18710226	18740801	chr4	20092413	20114647
992	CP026245.1	18741275	18744527	chr4	20081729	20091973
993	CP026245.1	18745731	18769659	chr4	20078318	20081506
994	CP026245.1	18770115	18793258	chr4	20075030	20076973
995	CP026245.1	18793387	18804851	chr4	20036209	20074892
996	CP026245.1	18805810	18826453	chr4	20022678	20036004
997	CP026245.1	18826699	18858179	chr4	19992025	20022504
998	CP026245.1	18858322	18883605	chr4	19950308	19991909
999	CP026245.1	18883707	18896685	chr4	19927543	19949696
1000	CP026245.1	18896821	18903551	chr4	19854899	19927057
1001	CP026245.1	18903700	18919073	chr4	19846312	19854689
1002	CP026245.1	18919160	18967129	chr4	19842359	19845437
1003	CP026245.1	18967309	18992332	chr4	19822344	19841991
1004	CP026245.1	18992925	19025687	chr4	19818786	19821819
1005	CP026245.1	19025914	19042515	chr4	19813448	19817502
1006	CP026245.1	19042759	19070330	chr4	19789320	19813325
1007	CP026245.1	19070672	19096794	chr4	19786150	19788817
1008	CP026245.1	19096890	19105614	chr4	19769490	19785874
1009	CP026245.1	19107157	19115120	chr4	19751185	19768636
1010	CP026245.1	19115242	19151154	chr4	19748981	19750956
1011	CP026245.1	19151409	19193574	chr4	19726035	19748610

1012	CP026245.1	19193713	19201731	chr4	19707131	19725006
1013	CP026245.1	19201875	19216164	chr4	19702328	19707012
1014	CP026245.1	19216460	19226287	chr4	19689377	19701847
1015	CP026245.1	19226532	19227851	chr4	19670665	19688132
1016	CP026245.1	19228019	19359645	chr4	19666480	19670090
1017	CP026245.1	19359823	19364073	chr4	19657268	19665814
1018	CP026245.1	19365227	19379393	chr4	19641067	19657179
1019	CP026245.1	19379551	19479002	chr4	19622179	19640737
1020	CP026245.1	19479969	19515765	chr4	19616482	19621962
1021	CP026245.1	19517892	19525990	chr4	19600043	19616234
1022	CP026245.1	19527887	19542724	chr4	19567925	19599920
1023	CP026245.1	19542907	19579361	chr4	19554545	19567465
1024	CP026245.1	19579475	19583651	chr4	19551287	19552701
1025	CP026245.1	19584047	19591322	chr4	19515893	19550223
1026	CP026245.1	19591518	19605939	chr4	19504664	19515718
1027	CP026245.1	19606114	19633736	chr4	19502050	19504489
1028	CP026245.1	19633844	19635290	chr4	19495163	19501956
1029	CP026245.1	19636109	19661313	chr4	19490399	19494948
1030	CP026245.1	19661416	19671244	chr4	19447358	19490295
1031	CP026245.1	19671565	19676034	chr4	19438344	19446628
1032	CP026245.1	19676134	19693185	chr4	19368212	19438203
1033	CP026245.1	19694479	19751652	chr4	19309309	19366482
1034	CP026245.1	19753382	19823373	chr4	19290964	19308015
1035	CP026245.1	19823514	19831798	chr4	19286395	19290864
1036	CP026245.1	19832528	19875465	chr4	19276246	19286074
1037	CP026245.1	19875569	19880118	chr4	19250939	19276143
1038	CP026245.1	19880333	19887126	chr4	19248674	19250120
1039	CP026245.1	19887220	19889659	chr4	19220944	19248566
1040	CP026245.1	19889834	19900888	chr4	19206348	19220769

1041	CP026245.1	19901063	19935393	chr4	19198877	19206152
1042	CP026245.1	19936457	19937871	chr4	19194305	19198481
1043	CP026245.1	19939715	19952635	chr4	19157737	19194191
1044	CP026245.1	19953095	19985090	chr4	19142717	19157554
1045	CP026245.1	19985213	20001404	chr4	19132722	19140820
1046	CP026245.1	20001652	20007132	chr4	19094799	19130595
1047	CP026245.1	20007349	20025907	chr4	18994381	19093832
1048	CP026245.1	20026237	20042349	chr4	18980057	18994223
1049	CP026245.1	20042438	20050984	chr4	18974653	18978903
1050	CP026245.1	20051650	20055260	chr4	18842849	18974475
1051	CP026245.1	20055835	20073302	chr4	18841362	18842681
1052	CP026245.1	20074547	20087017	chr4	18831290	18841117
1053	CP026245.1	20087498	20092182	chr4	18816705	18830994
1054	CP026245.1	20092301	20110176	chr4	18808543	18816561
1055	CP026245.1	20111205	20133780	chr4	18766239	18808404
1056	CP026245.1	20134151	20136126	chr4	18730072	18765984
1057	CP026245.1	20136355	20153806	chr4	18721987	18729950
1058	CP026245.1	20154660	20171044	chr4	18711720	18720444
1059	CP026245.1	20171320	20173987	chr4	18685502	18711624
1060	CP026245.1	20174490	20198495	chr4	18657589	18685160
1061	CP026245.1	20198618	20202672	chr4	18640744	18657345
1062	CP026245.1	20203956	20206989	chr4	18607755	18640517
1063	CP026245.1	20207514	20227161	chr4	18582139	18607162
1064	CP026245.1	20227529	20230607	chr4	18533990	18581959
1065	CP026245.1	20231482	20239859	chr4	18518530	18533903
1066	CP026245.1	20240069	20312227	chr4	18511651	18518381
1067	CP026245.1	20312713	20334866	chr4	18498537	18511515
1068	CP026245.1	20335478	20377079	chr4	18473152	18498435
1069	CP026245.1	20377195	20407674	chr4	18441529	18473009

1070	CP026245.1	20407848	20421174	chr4	18420640	18441283
1071	CP026245.1	20421379	20460062	chr4	18408217	18419681
1072	CP026245.1	20460200	20462143	chr4	18384945	18408088
1073	CP026245.1	20463488	20466676	chr4	18360561	18384489
1074	CP026245.1	20466899	20477143	chr4	18356105	18359357
1075	CP026245.1	20477583	20499817	chr4	18325056	18355631
1076	CP026245.1	20501062	20504780	chr4	18321754	18324831
1077	CP026245.1	20505062	20513438	chr4	18304872	18321423
1078	CP026245.1	20513680	20515870	chr4	18302119	18304584
1079	CP026245.1	20516038	20521251	chr4	18277355	18302031
1080	CP026245.1	20521680	20536209	chr4	18228318	18274726
1081	CP026245.1	20536468	20542799	chr4	18226637	18228112
1082	CP026245.1	20542915	20546905	chr4	18217916	18225977
1083	CP026245.1	20547871	20560205	chr4	18213333	18217815
1084	CP026245.1	20560706	20565022	chr4	18184643	18213160
1085	CP026245.1	20566696	20588645	chr4	18176273	18184464
1086	CP026245.1	20588792	20591207	chr4	18150316	18176186
1087	CP026245.1	20591315	20600731	chr4	18134567	18150143
1088	CP026245.1	20600841	20640647	chr4	18057477	18134380
1089	CP026245.1	20640882	20681987	chr4	17930963	18057376
1090	CP026245.1	20682107	20687848	chr4	17865239	17930839
1091	CP026245.1	20688052	20690538	chr4	17847879	17865068
1092	CP026245.1	20691322	20722527	chr4	17844967	17847634
1093	CP026245.1	20722771	20764245	chr4	17826458	17844874
1094	CP026245.1	20764392	20772115	chr4	17789592	17826219
1095	CP026245.1	20772423	20792553	chr4	17733060	17789141
1096	CP026245.1	20793020	20847755	chr4	17697464	17732739
1097	CP026245.1	20847956	20856581	chr4	17652041	17697176
1098	CP026245.1	20856997	20884180	chr4	17642193	17650141

1099	CP026245.1	20884566	20903469	chr4	17638324	17641989
1100	CP026245.1	20903855	20908914	chr4	17592734	17637456
1101	CP026245.1	20909529	20911068	chr4	17589614	17591477
1102	CP026245.1	20911374	20921378	chr4	17521604	17589408
1103	CP026245.1	20921558	20928938	chr4	17501599	17521481
1104	CP026245.1	20929352	20987284	chr4	17497975	17501327
1105	CP026245.1	20988583	21026147	chr4	17453214	17497767
1106	CP026245.1	21027361	21099214	chr4	17400607	17453004
1107	CP026245.1	21099607	21149733	chr4	17367855	17400493
1108	CP026245.1	21151347	21160523	chr4	17354215	17367676
1109	CP026245.1	21160931	21178087	chr4	17300228	17353088
1110	CP026245.1	21181357	21251816	chr4	17295520	17300067
1111	CP026245.1	21252740	21257608	chr4	17241264	17294865
1112	CP026245.1	21257746	21293321	chr4	17186892	17240877
1113	CP026245.1	21294385	21307187	chr4	17018965	17186339
1114	CP026245.1	21307461	21313723	chr4	17002677	17018752
1115	CP026245.1	21313861	21323966	chr4	16966418	17001377
1116	CP026245.1	21324070	21345316	chr4	16875565	16966208
1117	CP026245.1	21345531	21364913	chr4	16740022	16875113
1118	CP026245.1	21365770	21379149	chr4	16661041	16739680
1119	CP026245.1	21379441	21417323	chr4	16638606	16660926
1120	CP026245.1	21417563	21427305	chr4	16613605	16638399
1121	CP026245.1	21427822	21430906	chr4	16576915	16612361
1122	CP026245.1	21431036	21434971	chr4	16564280	16576576
1123	CP026245.1	21435077	21440583	chr4	16518248	16564123
1124	CP026245.1	21440832	21446643	chr4	16395542	16515917
1125	CP026245.1	21448795	21450004	chr4	16352846	16395454
1126	CP026245.1	21453524	21457044	chr4	16339569	16352731
1127	CP026245.1	21457304	21465098	chr4	16255583	16339307

1128	CP026245.1	21465304	21467519	chr4	16178436	16255432
1129	CP026245.1	21467698	21474632	chr4	16162765	16178173
1130	CP026245.1	21474764	21479373	chr4	16095738	16162262
1131	CP026245.1	21481398	21500549	chr4	16056088	16095647
1132	CP026245.1	21501584	21509838	chr4	16034368	16055987
1133	CP026245.1	21510580	21513745	chr4	15938087	16033950
1134	CP026245.1	21515725	21519332	chr4	15832882	15937987
1135	CP026245.1	21520600	21528834	chr4	15782175	15832781
1136	CP026245.1	21529569	21538987	chr4	15639779	15782057
1137	CP026245.1	21540374	21545114	chr4	15610879	15639514
1138	CP026245.1	21545699	21555772	chr4	15600275	15610633
1139	CP026245.1	21555952	21560907	chr4	15573652	15598923
1140	CP026245.1	21562351	21616972	chr4	15533111	15573550
1141	CP026245.1	21617145	21667713	chr4	15522930	15532182
1142	CP026245.1	21671528	21674321	chr4	15497873	15522823
1143	CP026245.1	21676020	21686285	chr4	15481857	15497381
1144	CP026245.1	21691152	21703114	chr4	15449844	15481709
1145	CP026245.1	21703553	21711414	chr4	15426340	15449684
1146	CP026245.1	21711810	21728015	chr4	15284535	15426236
1147	CP026245.1	21729026	21740531	chr4	15230500	15284345
1148	CP026245.1	21747881	21759052	chr4	15222002	15230385
1149	CP026245.1	21760470	21763768	chr4	15161590	15221770
1150	CP026245.1	21763911	21769535	chr4	15078230	15160370
1151	CP026245.1	21770001	21774013	chr4	15073965	15077740
1152	CP026245.1	21774137	21775429	chr4	15002877	15072675
1153	CP026245.1	21776373	21784705	chr4	15000027	15002718
1154	CP026245.1	21784865	21820448	chr4	14927841	14999227
1155	CP026245.1	21821901	21833448	chr4	14742531	14927663
1156	CP026245.1	21833785	21852506	chr4	14635211	14742429

1157	CP026245.1	21853169	21855383	chr4	14515888	14634981
1158	CP026245.1	21857627	21866928	chr4	14452405	14515783
1159	CP026245.1	21867249	21898867	chr4	14367820	14452191
1160	CP026245.1	21899001	21906558	chr4	14348780	14367658
1161	CP026245.1	21907531	21913947	chr4	14336899	14348606
1162	CP026245.1	21915952	21918439	chr4	14268401	14336782
1163	CP026245.1	21918581	21921408	chr4	14237513	14266440
1164	CP026245.1	21921692	21926300	chr4	14229383	14237242
1165	CP026245.1	21926516	21929213	chr4	14224651	14229282
1166	CP026245.1	21931067	21945514	chr4	14219066	14224509
1167	CP026245.1	21947009	21951707	chr4	13904572	14218932
1168	CP026245.1	21952025	21962685	chr4	13895803	13904442
1169	CP026245.1	21963137	21975830	chr4	13881215	13895637
1170	CP026245.1	21975935	21991398	chr4	13879093	13881110
1171	CP026245.1	21991574	22002026	chr4	13864350	13878916
1172	CP026245.1	22002896	22019487	chr4	13859605	13864072
1173	CP026245.1	22020324	22026030	chr4	13832585	13859439
1174	CP026245.1	22027932	22029367	chr4	13792645	13832376
1175	CP026245.1	22029615	22036437	chr4	13756622	13792511
1176	CP026245.1	22036779	22041743	chr4	13721172	13756519
1177	CP026245.1	22042163	22049704	chr4	13610724	13719280
1178	CP026245.1	22052484	22066572	chr4	13591101	13609393
1179	CP026245.1	22066891	22072307	chr4	13376282	13590923
1180	CP026245.1	22073012	22076215	chr4	13288740	13376172
1181	CP026245.1	22077075	22080290	chr4	13221241	13288432
1182	CP026245.1	22083850	22084993	chr4	13194364	13221115
1183	CP026245.1	22086995	22092800	chr4	12883403	13194272
1184	CP026245.1	22093993	22102900	chr4	12836795	12883314
1185	CP026245.1	22106297	22124755	chr4	12780169	12836609

1186	CP026245.1	22126463	22140946	chr4	12758440	12780069
1187	CP026245.1	22141454	22143037	chr4	12697325	12758191
1188	CP026245.1	22143892	22145508	chr4	12684438	12697117
1189	CP026245.1	22146046	22148248	chr4	12670055	12684216
1190	CP026245.1	22148703	22161020	chr4	12636023	12669946
1191	CP026245.1	22162196	22165925	chr4	12633224	12635508
1192	CP026245.1	22166027	22167233	chr4	12477228	12632978
1193	CP026245.1	22168819	22171783	chr4	12439715	12476908
1194	CP026245.1	22171941	22183458	chr4	12422992	12439522
1195	CP026245.1	22184565	22191509	chr4	12263291	12422796
1196	CP026245.1	22192947	22214967	chr4	12221695	12263117
1197	CP026245.1	22216152	22224401	chr4	12154008	12221554
1198	CP026245.1	22224534	22241598	chr4	11986738	12153519
1199	CP026245.1	22242055	22257281	chr4	11879496	11986639
1200	CP026245.1	22257696	22259943	chr4	11663251	11879289
1201	CP026245.1	22260039	22265443	chr4	11589356	11663142
1202	CP026245.1	22265633	22299498	chr4	11577509	11589186
1203	CP026245.1	22299730	22308342	chr4	11530197	11577383
1204	CP026245.1	22309201	22311940	chr4	11523907	11530105
1205	CP026245.1	22312055	22372569	chr4	11500381	11523481
1206	CP026245.1	22373241	22444325	chr4	11230520	11500229
1207	CP026245.1	22444606	22457737	chr4	11206305	11230412
1208	CP026245.1	22458439	22465247	chr4	11115390	11205840
1209	CP026245.1	22465635	22468349	chr4	11110477	11115156
1210	CP026245.1	22468867	22478138	chr4	11097024	11110074
1211	CP026245.1	22478397	22481482	chr4	11064633	11096857
1212	CP026245.1	22481854	22497748	chr4	11024092	11064472
1213	CP026245.1	22498506	22506307	chr4	10971692	11023989
1214	CP026245.1	22506730	22511235	chr4	10957353	10971440

1215	CP026245.1	22511573	22517023	chr4	10936273	10957001
1216	CP026245.1	22520540	22523185	chr4	10934384	10936119
1217	CP026245.1	22523416	22584909	chr4	10902747	10934246
1218	CP026245.1	22585031	22593079	chr4	10862421	10902346
1219	CP026245.1	22593186	22608863	chr4	10815573	10862172
1220	CP026245.1	22609097	22617038	chr4	10774876	10815453
1221	CP026245.1	22618790	22621392	chr4	10643419	10774772
1222	CP026245.1	22622484	22624312	chr4	10454278	10643318
1223	CP026245.1	22624407	22632830	chr4	10454177	10415885
1224	CP026245.1	22633116	22636796	chr4	10415750	10336374
1225	CP026245.1	22637034	22640652	chr4	10335431	9822751
1226	CP026245.1	22640804	22653294	chr4	9822658	9783902
1227	CP026245.1	22657257	22658831	chr4	9783786	9642047
1228	CP026245.1	22659541	22664973	chr4	9641907	9115176
1229	CP026245.1	22665488	22680286	chr4	9115010	9032741
1230	CP026245.1	22680862	22691003	chr4	9032423	9017488
1231	CP026245.1	22691190	22716801	chr4	9017225	8801157
1232	CP026245.1	22717935	22731464	chr4	8762972	8792179
1233	CP026245.1	22731642	22835279	chr4	8601286	8762849
1234	CP026245.1	22836648	22840194	chr4	8508883	8601190
1235	CP026245.1	22840666	22854760	chr4	8334052	8508792
1236	CP026245.1	22855269	22863775	chr4	8319701	8333803
1237	CP026245.1	22864686	22871099	chr4	8082566	8319540
1238	CP026245.1	22872863	22874384	chr4	8061182	8082429
1239	CP026245.1	22874592	22885712	chr4	8020803	8061062
1240	CP026245.1	22889102	22897941	chr4	7911912	8013052
1241	CP026245.1	22898066	22901290	chr4	7683912	7911775
1242	CP026245.1	22901581	22905224	chr4	7506767	7683769
1243	CP026245.1	22905635	22909576	chr4	7206806	7506539

1244	CP026245.1	22912510	22915097	chr4	7181297	7206588
1245	CP026245.1	22916111	22919230	chr4	7083628	7181102
1246	CP026245.1	22922813	22931367	chr4	7053895	7083275
1247	CP026245.1	22931497	22948650	chr4	6852098	7053495
1248	CP026245.1	22949111	22967883	chr4	6838652	6851683
1249	CP026245.1	22968998	22983919	chr4	6574929	6838321
1250	CP026245.1	22984020	22993083	chr4	6548943	6573404
1251	CP026245.1	22993685	22998713	chr4	6536626	6548829
1252	CP026245.1	23000612	23002217	chr4	6531591	6535204
1253	CP026245.1	23003460	23017505	chr4	6499485	6531484
1254	CP026245.1	23018514	23033952	chr4	6492535	6498398
1255	CP026245.1	23034129	23045010	chr4	6404714	6492228
1256	CP026245.1	23045668	23053193	chr4	6380385	6404593
1257	CP026245.1	23053317	23055203	chr4	6268086	6380272
1258	CP026245.1	23055904	23057725	chr4	6161347	6267675
1259	CP026245.1	23059548	23065474	chr4	6150610	6161054
1260	CP026245.1	23066730	23068781	chr4	6143024	6150214
1261	CP026245.1	23069302	23083433	chr4	6117730	6142337
1262	CP026245.1	23084446	23095510	chr4	6103769	6117573
1263	CP026245.1	23096361	23097936	chr4	6008982	6103534
1264	CP026245.1	23101129	23103152	chr4	5979420	6008427
1265	CP026245.1	23108851	23119581	chr4	5951877	5979318
1266	CP026245.1	23119858	23128340	chr4	5925013	5951343
1267	CP026245.1	23129341	23130967	chr4	5921369	5924925
1268	CP026245.1	23132092	23133479	chr4	5883309	5921259
1269	CP026245.1	23134865	23155248	chr4	5827016	5883156
1270	CP026245.1	23155493	23162257	chr4	5794794	5826799
1271	CP026245.1	23163915	23165589	chr4	5786224	5794705
1272	CP026245.1	23166305	23168196	chr4	5781536	5786058

1273	CP026245.1	23170132	23175672	chr4	5755572	5781352
1274	CP026245.1	23177674	23179096	chr4	5699408	5755327
1275	CP026245.1	23179431	23192112	chr4	5688535	5698038
1276	CP026245.1	23195242	23200397	chr4	5680557	5688365
1277	CP026245.1	23200498	23216013	chr4	5677239	5680268
1278	CP026245.1	23216220	23217468	chr4	5661485	5677098
1279	CP026245.1	23218070	23232316	chr4	5397709	5661351
1280	CP026245.1	23232807	23266772	chr4	5392585	5397361
1281	CP026245.1	23267420	23280371	chr4	5328989	5392140
1282	CP026245.1	23280803	23321985	chr4	5299278	5327625
1283	CP026245.1	23322231	23323725	chr4	5233050	5299189
1284	CP026245.1	23323857	23356076	chr4	5139415	5232962
1285	CP026245.1	23356662	23373952	chr4	5136015	5139282
1286	CP026245.1	23375540	23386974	chr4	5104316	5135468
1287	CP026245.1	23387679	23399176	chr4	5021407	5103265
1288	CP026245.1	23402066	23403691	chr4	4978259	5020939
1289	CP026245.1	23403792	23408522	chr4	4949253	4977869
1290	CP026245.1	23408655	23428142	chr4	4917748	4949161
1291	CP026245.1	23428650	23430430	chr4	4844714	4917605
1292	CP026245.1	23430598	23439702	chr4	4809418	4840634
1293	CP026245.1	23439924	23468376	chr4	4788389	4809168
1294	CP026245.1	23469335	23480145	chr4	4741034	4787559
1295	CP026245.1	23481032	23528091	chr4	4591774	4740855
1296	CP026245.1	23528488	23541263	chr4	4391269	4591261
1297	CP026245.1	23541741	23559270	chr4	4362841	4391083
1298	CP026245.1	23561722	23615440	chr4	4355331	4362520
1299	CP026245.1	23616290	23617600	chr4	4349398	4355216
1300	CP026245.1	23619312	23631794	chr4	4345621	4349129
1301	CP026245.1	23634066	23645370	chr4	4335550	4345233

1302	CP026245.1	23645735	23648340	chr4	4271273	4335245
1303	CP026245.1	23648790	23656772	chr4	4203469	4270981
1304	CP026245.1	23657250	23666620	chr4	4126636	4203336
1305	CP026245.1	23667330	23670150	chr4	4120977	4124091
1306	CP026245.1	23673308	23684477	chr4	4062453	4118217
1307	CP026245.1	23684710	23693488	chr4	4058246	4060853
1308	CP026245.1	23693626	23702940	chr4	4058145	3978910
1309	CP026245.1	23705790	23711108	chr4	3975507	3972375
1310	CP026245.1	23712683	23717045	chr4	3961977	3939351
1311	CP026245.1	23717313	23721748	chr4	3939250	3421694
1312	CP026245.1	23723021	23740909	chr4	3402916	3421593
1313	CP026245.1	23741292	23748679	chr4	3311082	3402716
1314	CP026245.1	23748781	23751540	chr4	3300481	3301813
1315	CP026245.1	23755315	23757050	chr4	3294912	3291591
1316	CP026245.1	23757823	23760165	chr4	3290103	3248781
1317	CP026245.1	23761210	23764464	chr4	3248516	3141423
1318	CP026245.1	23764590	23766155	chr4	3141331	2927302
1319	CP026245.1	23768075	23771319	chr4	2927149	2675426
1320	CP026245.1	23771520	23773289	chr4	2675267	2353642
1321	CP026245.1	23773911	23779814	chr4	2352727	2225540
1322	CP026245.1	23780929	23783524	chr4	2225171	2187856
1323	CP026245.1	23783742	23810279	chr4	2187701	1802648
1324	CP026245.1	23810404	23811982	chr4	1802532	1776893
1325	CP026245.1	23814317	23815788	chr4	1776499	1605016
1326	CP026245.1	23815900	23848124	chr4	1604397	1593049
1327	CP026245.1	23848288	23854236	chr4	1592893	1121193
1328	CP026245.1	23858297	23859733	chr4	1120925	1050323
1329	CP026245.1	23861691	23869196	chr4	1049895	1031450
1330	CP026245.1	23870637	23872403	chr4	1029831	907714

1331	CP026245.1	23873534	23880617	chr4	907580	852302
1332	CP026245.1	23882556	23891537	chr4	852076	816466
1333	CP026245.1	23892060	23901018	chr4	816122	770325
1334	CP026245.1	23902633	23908646	chr4	770134	744212
1335	CP026245.1	23908984	23916890	chr4	744103	571916
1336	CP026245.1	23919803	23923549	chr4	570901	472409
1337	CP026245.1	23923650	23927606	chr4	471829	437962
1338	CP026245.1	23930166	23931493	chr4	437663	436204
1339	CP026245.1	23931749	23940612	chr4	432616	423003
1340	CP026245.1	23941232	23949596	chr4	422754	419292
1341	CP026245.1	23950789	23952022	chr4	419099	331715
1342	CP026245.1	23952123	23953398	chr4	331510	312865
1343	CP026245.1	23956799	23960939	chr4	312636	309461
1344	CP026245.1	23961076	23965813	chr4	307330	304839
1345	CP026245.1	23966254	23974231	chr4	304543	284801
1346	CP026245.1	23977277	23995263	chr4	284235	255116
1347	CP026245.1	23996798	24028392	chr4	254888	246388
1348	CP026245.1	24028989	24044021	chr4	246196	234489
1349	CP026245.1	24044354	24058744	chr4	234243	225419
1350	CP026245.1	24058922	24060778	chr4	222578	212104
1351	CP026245.1	24060879	24064712	chr4	212000	187710
1352	CP026245.1	24065025	24066538	chr4	187582	176466
1353	CP026245.1	24067587	24068862	chr4	176141	133611
1354	CP026245.1	24069069	24078970	chr4	133521	101439
1355	CP026245.1	24079847	24083853	chr4	101284	42965
1356	CP026245.1	24085986	24094084	chr4	42758	19010
1357	CP026245.1	24096404	24130524	chr4	18815	1
1358	CP026253.1	8719129	8788921	chr5	22363673	22364397
1359	CP026253.1	8789149	8809991	chr5	22362753	22363515

1360	CP026253.1	8810441	8859071	chr5	21432554	21439639
1361	CP026253.1	8859212	9235728	chr5	21427231	21432385
1362	CP026253.1	9237321	9307790	chr5	21406025	21427131
1363	CP026253.1	9308027	9325770	chr5	21307826	21405911
1364	CP026253.1	9327262	9406140	chr5	21270987	21307712
1365	CP026253.1	9406241	9409396	chr5	21246361	21270733
1366	CP026253.1	9409482	9421794	chr5	21237131	21245914
1367	CP026253.1	9421935	9494420	chr5	21216946	21236901
1368	CP026253.1	9494538	9603036	chr5	21203658	21216857
1369	CP026253.1	9603256	9659204	chr5	21196108	21203072
1370	CP026253.1	9659374	9707244	chr5	21188948	21195652
1371	CP026253.1	9707380	9778281	chr5	21123913	21188033
1372	CP026253.1	9778566	9833774	chr5	21122331	21123825
1373	CP026253.1	9833938	9896964	chr5	21110182	21122091
1374	CP026253.1	9897077	9910549	chr5	21105826	21108034
1375	CP026253.1	9910640	9935301	chr5	21079669	21105681
1376	CP026253.1	9935456	9937773	chr5	21074969	21077409
1377	CP026253.1	9938021	9997852	chr5	21068234	21074876
1378	CP026253.1	9998052	10104795	chr5	20992149	21067981
1379	CP026253.1	10104881	10158127	chr5	20989451	20991603
1380	CP026253.1	10158322	10588509	chr5	20985986	20988832
1381	CP026253.1	10589093	10591714	chr5	20975496	20985520
1382	CP026253.1	10594956	10606025	chr5	20907745	20975313
1383	CP026253.1	10606155	10630663	chr5	20893110	20907630
1384	CP026253.1	10630804	10645539	chr5	20890706	20892212
1385	CP026253.1	10645646	10692345	chr5	20883269	20889568
1386	CP026253.1	10692570	10699023	chr5	20855193	20882906
1387	CP026253.1	10699464	10812922	chr5	20844231	20852955
1388	CP026253.1	10813184	10929137	chr5	20840692	20844079

1389	CP026253.1	10929286	10966800	chr5	20840092	20840606
1390	CP026253.1	10966914	10988789	chr5	20831316	20839550
1391	CP026253.1	10988878	11047966	chr5	20829310	20831210
1392	CP026253.1	11048235	11060032	chr5	20825041	20829092
1393	CP026253.1	11060185	11218894	chr5	20816246	20824761
1394	CP026253.1	11219192	11230011	chr5	20810500	20815521
1395	CP026253.1	11231353	11244115	chr5	20797712	20809393
1396	CP026253.1	11250451	11253886	chr5	20783339	20797562
1397	CP026253.1	11255112	11256764	chr5	20758517	20783188
1398	CP026253.1	11256910	11265162	chr5	20704605	20758218
1399	CP026253.1	11265524	11271323	chr5	20698861	20703601
1400	CP026253.1	11271752	11350024	chr5	20677002	20698623
1401	CP026253.1	11350173	11377084	chr5	20659202	20676750
1402	CP026253.1	11378133	11387550	chr5	20641408	20658995
1403	CP026253.1	11389277	11432418	chr5	20619617	20641275
1404	CP026253.1	11432526	11468653	chr5	20613121	20618744
1405	CP026253.1	11469459	11634612	chr5	20607547	20612983
1406	CP026253.1	11635493	11670347	chr5	20596896	20607262
1407	CP026253.1	11670462	11839664	chr5	20586992	20595800
1408	CP026253.1	11839973	11905301	chr5	20576050	20586883
1409	CP026253.1	11905448	12055602	chr5	20549185	20575933
1410	CP026253.1	12055715	12060650	chr5	20526700	20549043
1411	CP026253.1	12061752	12151003	chr5	20504767	20525750
1412	CP026253.1	12151102	12223726	chr5	20475141	20504655
1413	CP026253.1	12224080	12352969	chr5	20451800	20473815
1414	CP026253.1	12353475	12364162	chr5	20441910	20451613
1415	CP026253.1	12364740	12433102	chr5	20412491	20441740
1416	CP026253.1	12433296	12488386	chr5	20301064	20411375
1417	CP026253.1	12490226	12812264	chr5	20297924	20300312

1418	CP026253.1	12812351	12980502	chr5	20182660	20296860
1419	CP026253.1	12980833	13145153	chr5	20167161	20182537
1420	CP026253.1	13145255	13170771	chr5	20140138	20167074
1421	CP026253.1	13170879	13172995	chr5	20117803	20140031
1422	CP026253.1	13173302	13261998	chr5	20084745	20117713
1423	CP026253.1	13262293	13414948	chr5	20082382	20084649
1424	CP026253.1	13415215	13432726	chr5	20068212	20081276
1425	CP026253.1	13432812	13461563	chr5	20066156	20067664
1426	CP026253.1	13461664	13554780	chr5	20057871	20066001
1427	CP026253.1	13554875	13601022	chr5	20041936	20057633
1428	CP026253.1	13601264	13791582	chr5	20017374	20041665
1429	CP026253.1	13791674	13848118	chr5	19989630	20017115
1430	CP026253.1	13848293	13871956	chr5	19980857	19989495
1431	CP026253.1	13873261	13898171	chr5	19967219	19979214
1432	CP026253.1	13898361	14060130	chr5	19966916	19967122
1433	CP026253.1	14060544	14109168	chr5	19965013	19966821
1434	CP026253.1	14109742	14144574	chr5	19955464	19963814
1435	CP026253.1	14144757	14184883	chr5	19936679	19955265
1436	CP026253.1	14185143	14209946	chr5	19926590	19935412
1437	CP026253.1	14210598	14342727	chr5	19875829	19926270
1438	CP026253.1	14342877	14367907	chr5	19853247	19874502
1439	CP026253.1	14367996	14396213	chr5	19803509	19852086
1440	CP026253.1	14397141	14399639	chr5	19743828	19803422
1441	CP026253.1	14399869	14417712	chr5	19739896	19743715
1442	CP026253.1	14418313	14516676	chr5	19720171	19736336
1443	CP026253.1	14516814	14572217	chr5	19712666	19720023
1444	CP026253.1	14572441	14583066	chr5	19697113	19711190
1445	CP026253.1	14583189	14618520	chr5	19672225	19696915
1446	CP026253.1	14618891	14686251	chr5	19663804	19671431

1447	CP026253.1	14686415	14712871	chr5	19642177	19663304
1448	CP026253.1	14715367	14984394	chr5	19610097	19640953
1449	CP026253.1	14984580	14997086	chr5	19566496	19609176
1450	CP026253.1	14997255	15000210	chr5	19553939	19565439
1451	CP026253.1	15001839	15024785	chr5	19531650	19553310
1452	CP026253.1	15025143	15034381	chr5	19527483	19531515
1453	CP026253.1	15034547	15040965	chr5	19513764	19527133
1454	CP026253.1	15041123	15078471	chr5	19507204	19511688
1455	CP026253.1	15078863	15172845	chr5	19490896	19507030
1456	CP026253.1	15173012	15206827	chr5	19489746	19490803
1457	CP026253.1	15207220	15209392	chr5	19459513	19489521
1458	CP026253.1	15211829	15265537	chr5	19443691	19458994
1459	CP026253.1	15265643	15268525	chr5	19434438	19443596
1460	CP026253.1	15268727	15273935	chr5	19385060	19434265
1461	CP026253.1	15276004	15440182	chr5	19369633	19384852
1462	CP026253.1	15440305	15500671	chr5	19316498	19369399
1463	CP026253.1	15500804	15598079	chr5	19250777	19316406
1464	CP026253.1	15598191	15695035	chr5	19151968	19250462
1465	CP026253.1	15695138	15965522	chr5	19102638	19150232
1466	CP026253.1	15965748	16000617	chr5	19034669	19102538
1467	CP026253.1	16001207	16069125	chr5	18908907	19034419
1468	CP026253.1	16069293	16181730	chr5	18871700	18908381
1469	CP026253.1	16182388	16287997	chr5	18836995	18867119
1470	CP026253.1	16288366	16290568	chr5	18815705	18836754
1471	CP026253.1	16290915	16389001	chr5	18635486	18814828
1472	CP026253.1	16389137	16399810	chr5	18627380	18634064
1473	CP026253.1	16400060	16401754	chr5	18579880	18627265
1474	CP026253.1	16401897	16434583	chr5	18577132	18579721
1475	CP026253.1	16434699	16823032	chr5	18571682	18577026

1476	CP026253.1	16823149	16875557	chr5	18563341	18571235
1477	CP026253.1	16875933	16938976	chr5	18547344	18563046
1478	CP026253.1	16939277	17006081	chr5	18530565	18547224
1479	CP026253.1	17006255	17065308	chr5	18487708	18530363
1480	CP026253.1	17065414	17154803	chr5	18451716	18487587
1481	CP026253.1	17155015	17160802	chr5	18430830	18450273
1482	CP026253.1	17160997	17169870	chr5	18422832	18430567
1483	CP026253.1	17169967	17170250	chr5	18305820	18422483
1484	CP026253.1	17170357	17284675	chr5	18300066	18305718
1485	CP026253.1	17284953	17332934	chr5	18295804	18297699
1486	CP026253.1	17333122	17341783	chr5	18287814	18295643
1487	CP026253.1	17341884	17347000	chr5	18275888	18287718
1488	CP026253.1	17347138	17355257	chr5	18114065	18275751
1489	CP026253.1	17355487	17704749	chr5	18028871	18110031
1490	CP026253.1	17704957	17705916	chr5	18024759	18028123
1491	CP026253.1	17706021	17760783	chr5	18011242	18024424
1492	CP026253.1	17760871	17765678	chr5	18008480	18011103
1493	CP026253.1	17766066	17782325	chr5	17985207	18008054
1494	CP026253.1	17782675	17796631	chr5	17983306	17985120
1495	CP026253.1	17796764	17845947	chr5	17875739	17983089
1496	CP026253.1	17846080	17854152	chr5	17867848	17875498
1497	CP026253.1	17854513	17895500	chr5	17792556	17867683
1498	CP026253.1	17898107	17964460	chr5	17781419	17792368
1499	CP026253.1	17964583	17978663	chr5	17681265	17781330
1500	CP026253.1	17978778	17981559	chr5	17665151	17680566
1501	CP026253.1	17981723	18055918	chr5	17424757	17664660
1502	CP026253.1	18056013	18092200	chr5	17387208	17423395
1503	CP026253.1	18093562	18333465	chr5	17312918	17387113
1504	CP026253.1	18333956	18349371	chr5	17309973	17312754

1505	CP026253.1	18350070	18450135	chr5	17295778	17309858
1506	CP026253.1	18450224	18461173	chr5	17229302	17295655
1507	CP026253.1	18461361	18536488	chr5	17185708	17226695
1508	CP026253.1	18536653	18544303	chr5	17177275	17185347
1509	CP026253.1	18544544	18651894	chr5	17127959	17177142
1510	CP026253.1	18652111	18653925	chr5	17113870	17127826
1511	CP026253.1	18654012	18676859	chr5	17097261	17113520
1512	CP026253.1	18677285	18679908	chr5	17092066	17096873
1513	CP026253.1	18680047	18693229	chr5	17037216	17091978
1514	CP026253.1	18693564	18696928	chr5	17036152	17037111
1515	CP026253.1	18697676	18778836	chr5	16686682	17035944
1516	CP026253.1	18782870	18944556	chr5	16678333	16686452
1517	CP026253.1	18944693	18956523	chr5	16673079	16678195
1518	CP026253.1	18956619	18964448	chr5	16664317	16672978
1519	CP026253.1	18964609	18966504	chr5	16616148	16664129
1520	CP026253.1	18968871	18974523	chr5	16501552	16615870
1521	CP026253.1	18974625	19091288	chr5	16501162	16501445
1522	CP026253.1	19091637	19099372	chr5	16492192	16501065
1523	CP026253.1	19099635	19119078	chr5	16486210	16491997
1524	CP026253.1	19120521	19156392	chr5	16396609	16485998
1525	CP026253.1	19156513	19199168	chr5	16337450	16396503
1526	CP026253.1	19199370	19216029	chr5	16270472	16337276
1527	CP026253.1	19216149	19231851	chr5	16207128	16270171
1528	CP026253.1	19232146	19240040	chr5	16154344	16206752
1529	CP026253.1	19240487	19245831	chr5	15765894	16154227
1530	CP026253.1	19245937	19248526	chr5	15733092	15765778
1531	CP026253.1	19248685	19296070	chr5	15731255	15732949
1532	CP026253.1	19296185	19302869	chr5	15720332	15731005
1533	CP026253.1	19304291	19483633	chr5	15622110	15720196

1534	CP026253.1	19484510	19505559	chr5	15619561	15621763
1535	CP026253.1	19505800	19535924	chr5	15513583	15619192
1536	CP026253.1	19540505	19577186	chr5	15400488	15512925
1537	CP026253.1	19577712	19703224	chr5	15332402	15400320
1538	CP026253.1	19703474	19771343	chr5	15296943	15331812
1539	CP026253.1	19771443	19819037	chr5	15026333	15296717
1540	CP026253.1	19820773	19919267	chr5	14929386	15026230
1541	CP026253.1	19919582	19985211	chr5	14831999	14929274
1542	CP026253.1	19985303	20038204	chr5	14771500	14831866
1543	CP026253.1	20038438	20053657	chr5	14607199	14771377
1544	CP026253.1	20053865	20103070	chr5	14599922	14605130
1545	CP026253.1	20103243	20112401	chr5	14596838	14599720
1546	CP026253.1	20112496	20127799	chr5	14543024	14596732
1547	CP026253.1	20128318	20158326	chr5	14538415	14540587
1548	CP026253.1	20158551	20159608	chr5	14504207	14538022
1549	CP026253.1	20159701	20175835	chr5	14410058	14504040
1550	CP026253.1	20176009	20180493	chr5	14372318	14409666
1551	CP026253.1	20182569	20195938	chr5	14365742	14372160
1552	CP026253.1	20196288	20200320	chr5	14356338	14365576
1553	CP026253.1	20200455	20222115	chr5	14333034	14355980
1554	CP026253.1	20222744	20234244	chr5	14328450	14331405
1555	CP026253.1	20235301	20277981	chr5	14315775	14328281
1556	CP026253.1	20278902	20309758	chr5	14046562	14315589
1557	CP026253.1	20310982	20332109	chr5	14017610	14044066
1558	CP026253.1	20332609	20340236	chr5	13950086	14017446
1559	CP026253.1	20341030	20365720	chr5	13914384	13949715
1560	CP026253.1	20365918	20379995	chr5	13903636	13914261
1561	CP026253.1	20381471	20388828	chr5	13848009	13903412
1562	CP026253.1	20388976	20405141	chr5	13749508	13847871

1563	CP026253.1	20408701	20412520	chr5	13731064	13748907
1564	CP026253.1	20412633	20472227	chr5	13728336	13730834
1565	CP026253.1	20472314	20520891	chr5	13699191	13727408
1566	CP026253.1	20522052	20543307	chr5	13674072	13699102
1567	CP026253.1	20544634	20595075	chr5	13541793	13673922
1568	CP026253.1	20595395	20604217	chr5	13516338	13541141
1569	CP026253.1	20605484	20624070	chr5	13475952	13516078
1570	CP026253.1	20624269	20632619	chr5	13440937	13475769
1571	CP026253.1	20633818	20635626	chr5	13391739	13440363
1572	CP026253.1	20635721	20635927	chr5	13229556	13391325
1573	CP026253.1	20636024	20648019	chr5	13204456	13229366
1574	CP026253.1	20649662	20658300	chr5	13179488	13203151
1575	CP026253.1	20658435	20685920	chr5	13122869	13179313
1576	CP026253.1	20686179	20710470	chr5	12932459	13122777
1577	CP026253.1	20710741	20726438	chr5	12886070	12932217
1578	CP026253.1	20726676	20734806	chr5	12792859	12885975
1579	CP026253.1	20734961	20736469	chr5	12764007	12792758
1580	CP026253.1	20737017	20750081	chr5	12746410	12763921
1581	CP026253.1	20751187	20753454	chr5	12593488	12746143
1582	CP026253.1	20753550	20786518	chr5	12504497	12593193
1583	CP026253.1	20786608	20808836	chr5	12502074	12504190
1584	CP026253.1	20808943	20835879	chr5	12476450	12501966
1585	CP026253.1	20835966	20851342	chr5	12312028	12476348
1586	CP026253.1	20851465	20965665	chr5	12143546	12311697
1587	CP026253.1	20966729	20969117	chr5	11821421	12143459
1588	CP026253.1	20969869	21080180	chr5	11764491	11819581
1589	CP026253.1	21081296	21110545	chr5	11695935	11764297
1590	CP026253.1	21110715	21120418	chr5	11684670	11695357
1591	CP026253.1	21120605	21142620	chr5	11555275	11684164

1592	CP026253.1	21143946	21173460	chr5	11482297	11554921
1593	CP026253.1	21173572	21194555	chr5	11392947	11482198
1594	CP026253.1	21195505	21217848	chr5	11386910	11391845
1595	CP026253.1	21217990	21244738	chr5	11236643	11386797
1596	CP026253.1	21244855	21255688	chr5	11171168	11236496
1597	CP026253.1	21255797	21264605	chr5	11001657	11170859
1598	CP026253.1	21265701	21276067	chr5	10966688	11001542
1599	CP026253.1	21276352	21281788	chr5	10800654	10965807
1600	CP026253.1	21281926	21287549	chr5	10763721	10799848
1601	CP026253.1	21288422	21310080	chr5	10720472	10763613
1602	CP026253.1	21310213	21327800	chr5	10709328	10718745
1603	CP026253.1	21328007	21345555	chr5	10681368	10708279
1604	CP026253.1	21345807	21367428	chr5	10602947	10681219
1605	CP026253.1	21367666	21372406	chr5	10596719	10602518
1606	CP026253.1	21373410	21427023	chr5	10588105	10596357
1607	CP026253.1	21427322	21451993	chr5	10586307	10587959
1608	CP026253.1	21452144	21466367	chr5	10581646	10585081
1609	CP026253.1	21466517	21478198	chr5	10562548	10575310
1610	CP026253.1	21479305	21484326	chr5	10550387	10561206
1611	CP026253.1	21485051	21493566	chr5	10391380	10550089
1612	CP026253.1	21493846	21497897	chr5	10379430	10391227
1613	CP026253.1	21498115	21500015	chr5	10320073	10379161
1614	CP026253.1	21500121	21508355	chr5	10298109	10319984
1615	CP026253.1	21508897	21509411	chr5	10260481	10297995
1616	CP026253.1	21509497	21512884	chr5	10144379	10260332
1617	CP026253.1	21513036	21521760	chr5	10030659	10144117
1618	CP026253.1	21523998	21551711	chr5	10023765	10030218
1619	CP026253.1	21552074	21558373	chr5	9976841	10023540
1620	CP026253.1	21559511	21561017	chr5	9961999	9976734

1621	CP026253.1	21561915	21576435	chr5	9937350	9961858
1622	CP026253.1	21576550	21644118	chr5	9926151	9937220
1623	CP026253.1	21644301	21654325	chr5	9920288	9922909
1624	CP026253.1	21654791	21657637	chr5	9489517	9919704
1625	CP026253.1	21658256	21660408	chr5	9436076	9489322
1626	CP026253.1	21660954	21736786	chr5	9329247	9435990
1627	CP026253.1	21737039	21743681	chr5	9269216	9329047
1628	CP026253.1	21743774	21746214	chr5	9266651	9268968
1629	CP026253.1	21748474	21774486	chr5	9241835	9266496
1630	CP026253.1	21774631	21776839	chr5	9228272	9241744
1631	CP026253.1	21778987	21790896	chr5	9165133	9228159
1632	CP026253.1	21791136	21792630	chr5	9109761	9164969
1633	CP026253.1	21792718	21856838	chr5	9038575	9109476
1634	CP026253.1	21857753	21864457	chr5	8990569	9038439
1635	CP026253.1	21864913	21871877	chr5	8934451	8990399
1636	CP026253.1	21872463	21885662	chr5	8825733	8934231
1637	CP026253.1	21885751	21905706	chr5	8753130	8825615
1638	CP026253.1	21905936	21914719	chr5	8740677	8752989
1639	CP026253.1	21915166	21939538	chr5	8737436	8740591
1640	CP026253.1	21939792	21976517	chr5	8658457	8737335
1641	CP026253.1	21976631	22074716	chr5	8639222	8656965
1642	CP026253.1	22074830	22095936	chr5	8568516	8638985
1643	CP026253.1	22096036	22101190	chr5	8190407	8566923
1644	CP026253.1	22101359	22108444	chr5	8141636	8190266
1645	CP026253.1	23009051	23009775	chr5	8120344	8141186
1646	CP026253.1	23009933	23010695	chr5	8050324	8120116
1647	CP026260.1	20730485	20750279	chr6	559105	539311
1648	CP026260.1	20724671	20725949	chr6	564919	563641
1649	CP026260.1	20714822	20721188	chr6	574768	568402

1650	CP026260.1	20707569	20713871	chr6	582021	575719
1651	CP026260.1	20704476	20707186	chr6	585114	582404
1652	CP026260.1	20701619	20703734	chr6	587971	585856
1653	CP026260.1	20699053	20701147	chr6	590537	588443
1654	CP026260.1	20694195	20695987	chr6	595395	593603
1655	CP026260.1	20685290	20692962	chr6	604300	596628
1656	CP026260.1	20672273	20683502	chr6	617317	606088
1657	CP026260.1	20667119	20670701	chr6	622471	618889
1658	CP026260.1	20662467	20663662	chr6	627123	625928
1659	CP026260.1	20656185	20661254	chr6	633405	628336
1660	CP026260.1	20652462	20655057	chr6	637128	634533
1661	CP026260.1	20650287	20652347	chr6	639303	637243
1662	CP026260.1	20647946	20649638	chr6	641644	639952
1663	CP026260.1	20644230	20647782	chr6	645360	641808
1664	CP026260.1	20629806	20643904	chr6	659784	645686
1665	CP026260.1	20626697	20629513	chr6	662893	660077
1666	CP026260.1	20624585	20626577	chr6	665005	663013
1667	CP026260.1	20614461	20624254	chr6	675129	665336
1668	CP026260.1	20610965	20613728	chr6	678625	675862
1669	CP026260.1	20609062	20610842	chr6	680528	678748
1670	CP026260.1	20608086	20608926	chr6	681504	680664
1671	CP026260.1	20602199	20607988	chr6	687391	681602
1672	CP026260.1	20599953	20602000	chr6	689637	687590
1673	CP026260.1	20593989	20599513	chr6	695601	690077
1674	CP026260.1	20561859	20589168	chr6	727731	700422
1675	CP026260.1	20558873	20561082	chr6	730717	728508
1676	CP026260.1	20489433	20557611	chr6	800157	731979
1677	CP026260.1	20477588	20482923	chr6	812002	806667
1678	CP026260.1	20459857	20477285	chr6	829733	812305

1679	CP026260.1	20454361	20459770	chr6	835229	829820
1680	CP026260.1	20447065	20454075	chr6	842525	835515
1681	CP026260.1	20445327	20446931	chr6	844263	842659
1682	CP026260.1	20440293	20443707	chr6	849297	845883
1683	CP026260.1	20427898	20440091	chr6	861692	849499
1684	CP026260.1	20425103	20427367	chr6	864487	862223
1685	CP026260.1	20423772	20424662	chr6	865818	864928
1686	CP026260.1	20418541	20423680	chr6	871049	865910
1687	CP026260.1	20415889	20418426	chr6	873701	871164
1688	CP026260.1	20414503	20415761	chr6	875087	873829
1689	CP026260.1	20409316	20413102	chr6	880274	876488
1690	CP026260.1	20407014	20408315	chr6	882576	881275
1691	CP026260.1	20402361	20406496	chr6	887229	883094
1692	CP026260.1	20392854	20402246	chr6	896736	887344
1693	CP026260.1	20386058	20389415	chr6	903532	900175
1694	CP026260.1	20382272	20385944	chr6	907318	903646
1695	CP026260.1	20276997	20382129	chr6	1012593	907461
1696	CP026260.1	20267108	20276892	chr6	1022482	1012698
1697	CP026260.1	20256243	20264432	chr6	1033347	1025158
1698	CP026260.1	20250134	20255484	chr6	1039456	1034106
1699	CP026260.1	20221402	20249914	chr6	1068188	1039676
1700	CP026260.1	20189255	20221311	chr6	1100335	1068279
1701	CP026260.1	20186055	20188201	chr6	1103535	1101389
1702	CP026260.1	20182801	20185689	chr6	1106789	1103901
1703	CP026260.1	20113260	20182702	chr6	1176330	1106888
1704	CP026260.1	20084995	20112492	chr6	1204595	1177098
1705	CP026260.1	20066617	20084897	chr6	1222973	1204693
1706	CP026260.1	20059555	20064056	chr6	1230035	1225534
1707	CP026260.1	20057042	20058554	chr6	1232548	1231036

1708	CP026260.1	20041914	20056767	chr6	1247676	1232823
1709	CP026260.1	20037204	20041775	chr6	1252386	1247815
1710	CP026260.1	20033661	20035570	chr6	1255929	1254020
1711	CP026260.1	20026611	20033568	chr6	1262979	1256022
1712	CP026260.1	20022994	20024509	chr6	1266596	1265081
1713	CP026260.1	20015124	20022891	chr6	1274466	1266699
1714	CP026260.1	20010327	20014872	chr6	1279263	1274718
1715	CP026260.1	20007021	20010143	chr6	1282569	1279447
1716	CP026260.1	20000459	20006338	chr6	1289131	1283252
1717	CP026260.1	19992495	19999016	chr6	1297095	1290574
1718	CP026260.1	19989167	19991894	chr6	1300423	1297696
1719	CP026260.1	19988785	19989079	chr6	1300805	1300511
1720	CP026260.1	19982193	19985780	chr6	1307397	1303810
1721	CP026260.1	19980211	19981980	chr6	1309379	1307610
1722	CP026260.1	19954639	19978772	chr6	1334951	1310818
1723	CP026260.1	19932947	19954336	chr6	1356643	1335254
1724	CP026260.1	19911201	19932723	chr6	1378389	1356867
1725	CP026260.1	19904725	19910986	chr6	1384865	1378604
1726	CP026260.1	19900751	19904270	chr6	1388839	1385320
1727	CP026260.1	19884631	19900643	chr6	1404959	1388947
1728	CP026260.1	19881100	19884197	chr6	1408490	1405393
1729	CP026260.1	19879115	19880292	chr6	1410475	1409298
1730	CP026260.1	19875346	19878650	chr6	1414244	1410940
1731	CP026260.1	19870187	19873736	chr6	1419403	1415854
1732	CP026260.1	19866110	19869921	chr6	1423480	1419669
1733	CP026260.1	19862455	19865753	chr6	1427135	1423837
1734	CP026260.1	19851119	19861419	chr6	1438471	1428171
1735	CP026260.1	19803663	19850180	chr6	1485927	1439410
1736	CP026260.1	19797373	19803572	chr6	1492217	1486018

1737	CP026260.1	19793580	19796244	chr6	1496010	1493346
1738	CP026260.1	19775371	19792957	chr6	1514219	1496633
1739	CP026260.1	19769719	19775053	chr6	1519871	1514537
1740	CP026260.1	19753073	19769427	chr6	1536517	1520163
1741	CP026260.1	19714311	19752709	chr6	1575279	1536881
1742	CP026260.1	19708149	19713515	chr6	1581441	1576075
1743	CP026260.1	19699743	19707978	chr6	1589847	1581612
1744	CP026260.1	19653771	19699526	chr6	1635819	1590064
1745	CP026260.1	19619216	19653533	chr6	1670374	1636057
1746	CP026260.1	19613844	19618898	chr6	1675746	1670692
1747	CP026260.1	19524392	19613750	chr6	1765198	1675840
1748	CP026260.1	19372291	19523776	chr6	1917299	1765814
1749	CP026260.1	19324476	19372175	chr6	1965114	1917415
1750	CP026260.1	19303208	19324299	chr6	1986382	1965291
1751	CP026260.1	19294365	19301421	chr6	1995225	1988169
1752	CP026260.1	19290411	19293150	chr6	1999179	1996440
1753	CP026260.1	19267653	19290191	chr6	2021937	1999399
1754	CP026260.1	19258015	19267477	chr6	2031575	2022113
1755	CP026260.1	19244710	19252830	chr6	2044880	2036760
1756	CP026260.1	19226747	19244599	chr6	2062843	2044991
1757	CP026260.1	19211395	19226371	chr6	2078195	2063219
1758	CP026260.1	19193000	19209827	chr6	2096590	2079763
1759	CP026260.1	19189630	19191751	chr6	2099960	2097839
1760	CP026260.1	19174335	19189538	chr6	2115255	2100052
1761	CP026260.1	19166962	19173642	chr6	2122628	2115948
1762	CP026260.1	19163173	19166540	chr6	2126417	2123050
1763	CP026260.1	19159927	19162460	chr6	2129663	2127130
1764	CP026260.1	19155888	19159320	chr6	2133702	2130270
1765	CP026260.1	19153748	19155402	chr6	2135842	2134188

1766	CP026260.1	19123920	19152834	chr6	2165670	2136756
1767	CP026260.1	19121056	19123796	chr6	2168534	2165794
1768	CP026260.1	19097985	19120894	chr6	2191605	2168696
1769	CP026260.1	19087610	19097708	chr6	2201980	2191882
1770	CP026260.1	19085422	19087293	chr6	2204168	2202297
1771	CP026260.1	19060705	19084217	chr6	2228885	2205373
1772	CP026260.1	18982922	19060582	chr6	2306668	2229008
1773	CP026260.1	18937981	18982631	chr6	2351609	2306959
1774	CP026260.1	18921322	18937089	chr6	2368268	2352501
1775	CP026260.1	18915252	18921176	chr6	2374338	2368414
1776	CP026260.1	18910290	18915146	chr6	2379300	2374444
1777	CP026260.1	18899910	18909077	chr6	2389680	2380513
1778	CP026260.1	18896385	18899779	chr6	2393205	2389811
1779	CP026260.1	18867934	18896015	chr6	2421656	2393575
1780	CP026260.1	18824079	18867376	chr6	2465511	2422214
1781	CP026260.1	18811201	18823773	chr6	2478389	2465817
1782	CP026260.1	18808991	18810865	chr6	2480599	2478725
1783	CP026260.1	18796239	18808679	chr6	2493351	2480911
1784	CP026260.1	18794123	18796114	chr6	2495467	2493476
1785	CP026260.1	18791914	18793407	chr6	2497676	2496183
1786	CP026260.1	18785220	18791711	chr6	2504370	2497879
1787	CP026260.1	18781618	18783236	chr6	2507972	2506354
1788	CP026260.1	18760059	18778152	chr6	2529531	2511438
1789	CP026260.1	18753273	18759919	chr6	2536317	2529671
1790	CP026260.1	18736414	18752715	chr6	2553176	2536875
1791	CP026260.1	18733985	18736124	chr6	2555605	2553466
1792	CP026260.1	18731947	18733417	chr6	2557643	2556173
1793	CP026260.1	18726542	18731641	chr6	2563048	2557949
1794	CP026260.1	18706878	18723418	chr6	2582712	2566172

1795	CP026260.1	18704324	18706746	chr6	2585266	2582844
1796	CP026260.1	18700611	18704043	chr6	2588979	2585547
1797	CP026260.1	18690495	18700332	chr6	2599095	2589258
1798	CP026260.1	18668333	18690396	chr6	2621257	2599194
1799	CP026260.1	18666164	18668210	chr6	2623426	2621380
1800	CP026260.1	18645248	18666077	chr6	2644342	2623513
1801	CP026260.1	18635604	18644191	chr6	2653986	2645399
1802	CP026260.1	18632310	18635175	chr6	2657280	2654415
1803	CP026260.1	18585879	18632222	chr6	2703711	2657368
1804	CP026260.1	18577499	18584919	chr6	2712091	2704671
1805	CP026260.1	18537790	18577022	chr6	2751800	2712568
1806	CP026260.1	18502911	18536835	chr6	2786679	2752755
1807	CP026260.1	18495881	18502804	chr6	2793709	2786786
1808	CP026260.1	18476141	18495326	chr6	2813449	2794264
1809	CP026260.1	18472531	18475681	chr6	2817059	2813909
1810	CP026260.1	18464136	18471225	chr6	2825454	2818365
1811	CP026260.1	18463421	18464045	chr6	2826169	2825545
1812	CP026260.1	18459735	18463332	chr6	2829855	2826258
1813	CP026260.1	18442989	18459629	chr6	2846601	2829961
1814	CP026260.1	18383359	18442770	chr6	2906231	2846820
1815	CP026260.1	18303157	18382349	chr6	2986433	2907241
1816	CP026260.1	18231887	18298225	chr6	3057703	2991365
1817	CP026260.1	18227669	18231264	chr6	3061921	3058326
1818	CP026260.1	18154178	18227151	chr6	3135412	3062439
1819	CP026260.1	18123488	18128027	chr6	3166102	3161563
1820	CP026260.1	18107040	18122020	chr6	3181183	3166203
1821	CP026260.1	18048799	18106946	chr6	3239424	3181277
1822	CP026260.1	18011458	18048401	chr6	3276765	3239822
1823	CP026260.1	18004524	18011249	chr6	3283699	3276974

1824	CP026260.1	17997490	18004306	chr6	3290733	3283917
1825	CP026260.1	17991152	17997022	chr6	3297071	3291201
1826	CP026260.1	17983070	17991049	chr6	3305153	3297174
1827	CP026260.1	17979725	17982911	chr6	3308498	3305312
1828	CP026260.1	17904052	17979610	chr6	3384171	3308613
1829	CP026260.1	17877022	17903818	chr6	3411201	3384405
1830	CP026260.1	17874468	17876923	chr6	3413755	3411300
1831	CP026260.1	17869040	17874317	chr6	3419183	3413906
1832	CP026260.1	17826665	17868728	chr6	3461558	3419495
1833	CP026260.1	17825014	17826424	chr6	3463209	3461799
1834	CP026260.1	17814282	17824638	chr6	3473941	3463585
1835	CP026260.1	17812171	17813563	chr6	3476052	3474660
1836	CP026260.1	17809236	17810436	chr6	3478987	3477787
1837	CP026260.1	17807574	17809099	chr6	3480649	3479124
1838	CP026260.1	17797250	17807403	chr6	3490973	3480820
1839	CP026260.1	17796219	17797142	chr6	3492004	3491081
1840	CP026260.1	17748967	17794943	chr6	3539256	3493280
1841	CP026260.1	17736895	17748855	chr6	3551328	3539368
1842	CP026260.1	17720328	17735790	chr6	3567895	3552433
1843	CP026260.1	17720044	17720182	chr6	3568179	3568041
1844	CP026260.1	17707219	17716682	chr6	3581004	3571541
1845	CP026260.1	17686382	17707125	chr6	3601841	3581098
1846	CP026260.1	17680625	17686089	chr6	3607598	3602134
1847	CP026260.1	17666998	17680403	chr6	3621225	3607820
1848	CP026260.1	17649580	17666839	chr6	3638643	3621384
1849	CP026260.1	17647175	17649279	chr6	3641048	3638944
1850	CP026260.1	17639375	17646859	chr6	3648848	3641364
1851	CP026260.1	17636815	17639163	chr6	3651408	3649060
1852	CP026260.1	17630273	17635690	chr6	3657950	3652533

1853	CP026260.1	17612083	17630132	chr6	3676140	3658091
1854	CP026260.1	17602894	17611857	chr6	3685329	3676366
1855	CP026260.1	17597854	17601907	chr6	3690369	3686316
1856	CP026260.1	17595569	17597724	chr6	3692654	3690499
1857	CP026260.1	17544210	17595274	chr6	3744013	3692949
1858	CP026260.1	17542744	17544119	chr6	3745479	3744104
1859	CP026260.1	17537805	17542625	chr6	3750418	3745598
1860	CP026260.1	17528867	17532558	chr6	3759356	3755665
1861	CP026260.1	17525148	17528679	chr6	3763075	3759544
1862	CP026260.1	17513860	17523586	chr6	3774363	3764637
1863	CP026260.1	17505095	17512983	chr6	3783128	3775240
1864	CP026260.1	17504350	17505004	chr6	3783873	3783219
1865	CP026260.1	17499638	17503861	chr6	3788585	3784362
1866	CP026260.1	17497805	17499159	chr6	3790418	3789064
1867	CP026260.1	17477199	17497617	chr6	3811024	3790606
1868	CP026260.1	17475463	17476972	chr6	3812760	3811251
1869	CP026260.1	17464181	17474047	chr6	3824042	3814176
1870	CP026260.1	17456304	17464039	chr6	3831919	3824184
1871	CP026260.1	17448297	17455898	chr6	3839926	3832325
1872	CP026260.1	17438441	17447308	chr6	3849782	3840915
1873	CP026260.1	17402344	17438286	chr6	3885879	3849937
1874	CP026260.1	17398325	17402224	chr6	3889898	3885999
1875	CP026260.1	17396556	17397903	chr6	3891667	3890320
1876	CP026260.1	17384014	17396329	chr6	3904209	3891894
1877	CP026260.1	17328958	17383862	chr6	3959265	3904361
1878	CP026260.1	17261036	17328637	chr6	4027187	3959586
1879	CP026260.1	17258844	17260840	chr6	4029379	4027383
1880	CP026260.1	17250091	17258571	chr6	4038132	4029652
1881	CP026260.1	17203023	17249934	chr6	4085200	4038289

1882	CP026260.1	17190849	17202728	chr6	4097374	4085495
1883	CP026260.1	17175888	17190694	chr6	4112335	4097529
1884	CP026260.1	17122458	17175640	chr6	4165765	4112583
1885	CP026260.1	17024816	17121420	chr6	4263407	4166803
1886	CP026260.1	17018246	17024525	chr6	4269977	4263698
1887	CP026260.1	17013875	17016718	chr6	4274348	4271505
1888	CP026260.1	16993838	17013027	chr6	4294385	4275196
1889	CP026260.1	16987241	16993510	chr6	4300982	4294713
1890	CP026260.1	16976218	16987056	chr6	4312005	4301167
1891	CP026260.1	16967753	16976126	chr6	4320470	4312097
1892	CP026260.1	16855321	16965802	chr6	4432902	4322421
1893	CP026260.1	16817494	16855181	chr6	4470729	4433042
1894	CP026260.1	16745710	16817401	chr6	4542513	4470822
1895	CP026260.1	16744017	16745559	chr6	4544206	4542664
1896	CP026260.1	16728710	16743559	chr6	4559513	4544664
1897	CP026260.1	16724953	16728541	chr6	4563270	4559682
1898	CP026260.1	16698603	16724550	chr6	4589620	4563673
1899	CP026260.1	16690773	16698300	chr6	4597450	4589923
1900	CP026260.1	16687752	16690134	chr6	4600471	4598089
1901	CP026260.1	16633754	16687161	chr6	4654469	4601062
1902	CP026260.1	16580146	16632442	chr6	4708077	4655781
1903	CP026260.1	16574491	16579985	chr6	4713732	4708238
1904	CP026260.1	16457369	16573176	chr6	4830854	4715047
1905	CP026260.1	16413703	16457094	chr6	4874520	4831129
1906	CP026260.1	16402811	16413459	chr6	4885412	4874764
1907	CP026260.1	16377050	16402613	chr6	4911173	4885610
1908	CP026260.1	16356204	16376714	chr6	4932019	4911509
1909	CP026260.1	16307124	16355995	chr6	4981099	4932228
1910	CP026260.1	16148797	16307025	chr6	5139426	4981198

1911	CP026260.1	16105213	16147577	chr6	5183010	5140646
1912	CP026260.1	16102850	16104149	chr6	5185373	5184074
1913	CP026260.1	16064867	16102631	chr6	5223356	5185592
1914	CP026260.1	15969998	16064160	chr6	5318225	5224063
1915	CP026260.1	15875650	15969854	chr6	5412573	5318369
1916	CP026260.1	15787363	15875544	chr6	5500860	5412679
1917	CP026260.1	15737975	15784039	chr6	5550248	5504184
1918	CP026260.1	15736305	15737802	chr6	5551918	5550421
1919	CP026260.1	15700743	15736037	chr6	5587480	5552186
1920	CP026260.1	15661769	15697359	chr6	5626454	5590864
1921	CP026260.1	15630112	15661623	chr6	5658111	5626600
1922	CP026260.1	15620411	15629883	chr6	5667812	5658340
1923	CP026260.1	15616196	15619921	chr6	5672027	5668302
1924	CP026260.1	15609731	15616105	chr6	5678492	5672118
1925	CP026260.1	15609031	15609590	chr6	5679192	5678633
1926	CP026260.1	15592778	15608933	chr6	5695445	5679290
1927	CP026260.1	15580331	15592585	chr6	5707892	5695638
1928	CP026260.1	15541026	15580227	chr6	5747197	5707996
1929	CP026260.1	15478361	15540893	chr6	5809862	5747330
1930	CP026260.1	15385335	15477980	chr6	5902888	5810243
1931	CP026260.1	15296097	15384951	chr6	5992126	5903272
1932	CP026260.1	15292580	15295990	chr6	5995643	5992233
1933	CP026260.1	15089390	15292225	chr6	6198833	5995998
1934	CP026260.1	15002499	15089276	chr6	6285724	6198947
1935	CP026260.1	14859755	15002362	chr6	6428468	6285861
1936	CP026260.1	14829351	14859631	chr6	6458872	6428592
1937	CP026260.1	14779698	14829239	chr6	6508525	6458984
1938	CP026260.1	14538188	14779560	chr6	6750035	6508663
1939	CP026260.1	14380722	14537160	chr6	6907501	6751063

1940	CP026260.1	14158436	14380561	chr6	7129787	6907662
1941	CP026260.1	14103080	14158320	chr6	7185143	7129903
1942	CP026260.1	14077015	14102611	chr6	7211208	7185612
1943	CP026260.1	14036875	14075443	chr6	7251348	7212780
1944	CP026260.1	13736724	14036610	chr6	7551499	7251613
1945	CP026260.1	13512609	13736047	chr6	7775614	7552176
1946	CP026260.1	13252828	13512487	chr6	8035395	7775736
1947	CP026260.1	12841313	13251530	chr6	8446910	8036693
1948	CP026260.1	12632416	12841212	chr6	8655807	8447011
1949	CP026260.1	12631675	12632270	chr6	8656548	8655953
1950	CP026260.1	12614505	12628764	chr6	8673718	8659459
1951	CP026260.1	12467148	12613884	chr6	8821075	8674339
1952	CP026260.1	12280882	12466688	chr6	9007341	8821535
1953	CP026260.1	11982660	12280000	chr6	9305563	9008223
1954	CP026260.1	11838247	11982415	chr6	9449976	9305808
1955	CP026260.1	11696226	11835314	chr6	9591997	9452909
1956	CP026260.1	11617104	11695880	chr6	9671119	9592343
1957	CP026260.1	11593249	11613892	chr6	9694974	9674331
1958	CP026260.1	11358157	11593085	chr6	9930066	9695138
1959	CP026260.1	11354868	11358042	chr6	9933355	9930181
1960	CP026260.1	11284486	11354378	chr6	10003737	9933845
1961	CP026260.1	11230690	11284311	chr6	10057533	10003912
1962	CP026260.1	11225587	11230059	chr6	10062636	10058164
1963	CP026260.1	11213840	11225306	chr6	10074383	10062917
1964	CP026260.1	11191812	11213741	chr6	10096411	10074482
1965	CP026260.1	10752722	11190900	chr6	10535501	10097323
1966	CP026260.1	10746563	10752541	chr6	10541660	10535682
1967	CP026260.1	10331025	10745767	chr6	10957198	10542456
1968	CP026260.1	10313002	10330716	chr6	10975221	10957507

1969	CP026260.1	10217968	10312113	chr6	11070255	10976110
1970	CP026260.1	10199886	10217825	chr6	11088337	11070398
1971	CP026260.1	9853006	10199734	chr6	11435217	11088489
1972	CP026260.1	9836041	9852616	chr6	11452182	11435607
1973	CP026260.1	9802424	9835935	chr6	11485799	11452288
1974	CP026260.1	9413437	9801931	chr6	11874786	11486292
1975	CP026260.1	9363568	9413191	chr6	11924655	11875032
1976	CP026260.1	8887059	9363261	chr6	12401164	11924962
1977	CP026260.1	8739137	8886712	chr6	12549086	12401511
1978	CP026260.1	8737665	8738538	chr6	12550558	12549685
1979	CP026260.1	8708268	8737524	chr6	12579955	12550699
1980	CP026260.1	8636572	8707752	chr6	12651651	12580471
1981	CP026260.1	8451505	8636428	chr6	12836718	12651795
1982	CP026260.1	8442025	8451040	chr6	12846198	12837183
1983	CP026260.1	8340212	8440733	chr6	12948011	12847490
1984	CP026260.1	8291386	8340125	chr6	12996837	12948098
1985	CP026260.1	8240519	8290681	chr6	13047704	12997542
1986	CP026260.1	8136045	8240391	chr6	13152178	13047832
1987	CP026260.1	8116867	8135939	chr6	13171356	13152284
1988	CP026260.1	8093934	8116736	chr6	13194289	13171487
1989	CP026260.1	8057969	8093799	chr6	13230254	13194424
1990	CP026260.1	8029454	8057741	chr6	13258769	13230482
1991	CP026260.1	7971264	8029218	chr6	13316959	13259005
1992	CP026260.1	7874414	7971068	chr6	13413809	13317155
1993	CP026260.1	7776844	7874213	chr6	13511379	13414010
1994	CP026260.1	7770952	7776414	chr6	13517271	13511809
1995	CP026260.1	7760405	7769536	chr6	13527818	13518687
1996	CP026260.1	7613537	7760223	chr6	13674686	13528000
1997	CP026260.1	7417617	7613427	chr6	13870606	13674796

1998	CP026260.1	7361080	7417487	chr6	13927143	13870736
1999	CP026260.1	7190159	7360991	chr6	14098064	13927232
2000	CP026260.1	7148049	7190057	chr6	14140174	14098166
2001	CP026260.1	7118025	7147502	chr6	14170198	14140721
2002	CP026260.1	7085107	7117931	chr6	14203116	14170292
2003	CP026260.1	7084763	7084982	chr6	14203460	14203241
2004	CP026260.1	6743860	7084566	chr6	14544363	14203657
2005	CP026260.1	6732605	6743627	chr6	14555618	14544596
2006	CP026260.1	6680371	6731631	chr6	14607852	14556592
2007	CP026260.1	6639269	6680084	chr6	14648954	14608139
2008	CP026260.1	6618521	6639065	chr6	14669702	14649158
2009	CP026260.1	6575385	6618319	chr6	14712838	14669904
2010	CP026260.1	6556087	6575180	chr6	14732136	14713043
2011	CP026260.1	6552619	6555859	chr6	14735604	14732364
2012	CP026260.1	6244473	6552226	chr6	15043750	14735997
2013	CP026260.1	6182283	6244257	chr6	15105940	15043966
2014	CP026260.1	5990788	6182046	chr6	15297435	15106177
2015	CP026260.1	5966858	5990663	chr6	15321365	15297560
2016	CP026260.1	5886394	5966601	chr6	15401829	15321622
2017	CP026260.1	5842584	5885952	chr6	15445639	15402271
2018	CP026260.1	5720862	5842293	chr6	15567361	15445930
2019	CP026260.1	5649469	5720456	chr6	15638754	15567767
2020	CP026260.1	5637633	5649337	chr6	15650590	15638886
2021	CP026260.1	5345196	5637457	chr6	15943027	15650766
2022	CP026260.1	5187770	5344490	chr6	16100453	15943733
2023	CP026260.1	5173234	5187528	chr6	16114989	16100695
2024	CP026260.1	5145044	5173041	chr6	16143179	16115182
2025	CP026260.1	5098522	5144874	chr6	16189701	16143349
2026	CP026260.1	5036209	5098334	chr6	16252014	16189889

2027	CP026263.1	1	17109	chr7	18818802	18820081
2028	CP026263.1	17201	25842	chr7	18816999	18818523
2029	CP026263.1	26079	70575	chr7	18808488	18813767
2030	CP026263.1	70769	116907	chr7	18803900	18807781
2031	CP026263.1	117036	337569	chr7	18799086	18800785
2032	CP026263.1	337703	713744	chr7	18774344	18797377
2033	CP026263.1	609997	610097	chr7	18772118	18774138
2034	CP026263.1	715079	735021	chr7	18769757	18771636
2035	CP026263.1	735228	942875	chr7	18766966	18766584
2036	CP026263.1	943382	946788	chr7	18766516	18769595
2037	CP026263.1	946892	1041761	chr7	18765840	18765458
2038	CP026263.1	1042372	1110627	chr7	18762908	18765840
2039	CP026263.1	1110741	1143398	chr7	18758506	18761122
2040	CP026263.1	1143669	1150708	chr7	18753081	18756272
2041	CP026263.1	1150940	1279248	chr7	18750167	18751522
2042	CP026263.1	1279351	1432942	chr7	18747016	18748617
2043	CP026263.1	1433411	1559355	chr7	18736809	18745478
2044	CP026263.1	1559824	1681138	chr7	18710837	18735363
2045	CP026263.1	1681275	1723582	chr7	18704631	18710282
2046	CP026263.1	1723803	1733314	chr7	18702152	18704505
2047	CP026263.1	1733635	1744299	chr7	18696363	18701131
2048	CP026263.1	1744411	1879012	chr7	18687200	18695975
2049	CP026263.1	1879265	1899541	chr7	18685229	18687104
2050	CP026263.1	1899804	2275542	chr7	18682210	18684695
2051	CP026263.1	2069100	2070157	chr7	18676394	18681666
2052	CP026263.1	2275920	2319867	chr7	18674107	18675936
2053	CP026263.1	2319955	2557599	chr7	18667440	18673995
2054	CP026263.1	2557723	2558939	chr7	18663009	18667036
2055	CP026263.1	2559393	2598631	chr7	18658946	18661606

2056	CP026263.1	2598747	2618072	chr7	18626100	18655800
2057	CP026263.1	2618192	2676196	chr7	18616976	18624212
2058	CP026263.1	2676287	2680257	chr7	18613247	18616445
2059	CP026263.1	2680618	2842247	chr7	18607576	18610218
2060	CP026263.1	2842436	2905171	chr7	18603206	18607049
2061	CP026263.1	2905479	3091134	chr7	18586763	18601822
2062	CP026263.1	3091713	3143947	chr7	18585237	18586613
2063	CP026263.1	3144070	3215038	chr7	18577775	18584219
2064	CP026263.1	3215138	3233910	chr7	18565235	18577637
2065	CP026263.1	3234061	3241492	chr7	18555184	18564633
2066	CP026263.1	3242066	3273982	chr7	18551863	18554798
2067	CP026263.1	3274425	3458037	chr7	18551762	18549897
2068	CP026263.1	3458218	3474450	chr7	18548840	18546503
2069	CP026263.1	3474605	3483530	chr7	18546030	18541382
2070	CP026263.1	3483657	3492340	chr7	18539524	18533642
2071	CP026263.1	3492553	3502221	chr7	18534800	18534941
2072	CP026263.1	3502311	3506344	chr7	18532525	18519932
2073	CP026263.1	3506494	3515043	chr7	18531351	18531492
2074	CP026263.1	3516512	3569405	chr7	18519461	18517441
2075	CP026263.1	3569756	3614026	chr7	18516986	18515307
2076	CP026263.1	3614161	3622004	chr7	18512591	18502483
2077	CP026263.1	3622288	3624850	chr7	18501781	18494201
2078	CP026263.1	3625199	3644418	chr7	18493364	18477779
2079	CP026263.1	3644556	3666775	chr7	18477246	18475471
2080	CP026263.1	3666884	4014865	chr7	18474403	18460127
2081	CP026263.1	4014987	4019146	chr7	18459257	18455703
2082	CP026263.1	4019370	4098595	chr7	18455559	18455326
2083	CP026263.1	4098906	4248938	chr7	18455159	18442103
2084	CP026263.1	4137000	4137424	chr7	18441273	18434645

2085	CP026263.1	4137769	4138193	chr7	18431476	18417805
2086	CP026263.1	4249182	4250474	chr7	18417193	18412141
2087	CP026263.1	4250665	4377520	chr7	18412020	18406555
2088	CP026263.1	4378042	4386153	chr7	18405985	18389654
2089	CP026263.1	4386396	4441468	chr7	18392892	18393663
2090	CP026263.1	4441657	4488117	chr7	18389428	18388298
2091	CP026263.1	4491484	4578829	chr7	18388113	18378956
2092	CP026263.1	4578949	4775047	chr7	18378671	18376705
2093	CP026263.1	4761227	4761347	chr7	18375402	18371173
2094	CP026263.1	4763265	4763385	chr7	18370129	18364641
2095	CP026263.1	4776458	5056291	chr7	18363503	18361007
2096	CP026263.1	5001742	5002045	chr7	18357620	18356273
2097	CP026263.1	5002047	5002338	chr7	18354093	18350018
2098	CP026263.1	5002307	5002610	chr7	18353930	18354062
2099	CP026263.1	5002580	5002871	chr7	18349735	18345928
2100	CP026263.1	5010910	5011192	chr7	18345132	18343431
2101	CP026263.1	5056484	5079654	chr7	18343025	18341337
2102	CP026263.1	5079945	5092377	chr7	18339433	18334831
2103	CP026263.1	5083741	5084324	chr7	18334329	18332715
2104	CP026263.1	5092540	5117318	chr7	18329763	18328017
2105	CP026263.1	5117478	5137570	chr7	18326603	18312682
2106	CP026263.1	5138853	5143524	chr7	18309624	18307705
2107	CP026263.1	5143842	5151060	chr7	18305060	18296336
2108	CP026263.1	5151167	5193959	chr7	18296171	18291408
2109	CP026263.1	5196025	5204790	chr7	18290025	18284619
2110	CP026263.1	5204960	5210151	chr7	18284362	18279734
2111	CP026263.1	5210379	5252389	chr7	18276425	18275178
2112	CP026263.1	5252485	5350014	chr7	18274774	18271904
2113	CP026263.1	5307987	5308599	chr7	18270108	18267764

2114	CP026263.1	5308568	5308730	chr7	18266444	18259021
2115	CP026263.1	5308701	5309313	chr7	18257959	18247392
2116	CP026263.1	5309315	5309646	chr7	18246755	18244552
2117	CP026263.1	5309648	5309810	chr7	18244299	18233557
2118	CP026263.1	5309782	5310113	chr7	18232966	18227996
2119	CP026263.1	5350797	5363303	chr7	18226519	18224908
2120	CP026263.1	5363477	5455548	chr7	18223924	18221819
2121	CP026263.1	5413866	5414220	chr7	18219745	18217419
2122	CP026263.1	5455641	5543661	chr7	18217213	18212363
2123	CP026263.1	5511950	5512069	chr7	18209301	18205107
2124	CP026263.1	5543773	5545506	chr7	18203085	18194159
2125	CP026263.1	5545627	5582260	chr7	18192957	18190909
2126	CP026263.1	5582554	5605085	chr7	18188444	18182767
2127	CP026263.1	5605463	5609888	chr7	18184294	18184772
2128	CP026263.1	5610041	5632031	chr7	18183621	18184099
2129	CP026263.1	5632131	5653602	chr7	18178061	18176563
2130	CP026263.1	5653816	5696176	chr7	18175942	18174241
2131	CP026263.1	5696277	5727522	chr7	18173139	18171871
2132	CP026263.1	5727649	5772663	chr7	18171348	18163052
2133	CP026263.1	5772763	5795855	chr7	18162639	18153823
2134	CP026263.1	5796168	5875543	chr7	18159022	18159471
2135	CP026263.1	5875682	5877087	chr7	18158455	18158789
2136	CP026263.1	5877227	5911890	chr7	18158338	18158787
2137	CP026263.1	5912195	5926345	chr7	18158002	18158336
2138	CP026263.1	5929895	5931539	chr7	18153709	18152811
2139	CP026263.1	5933724	5937059	chr7	18152084	18150547
2140	CP026263.1	5938261	5953884	chr7	18150201	18138609
2141	CP026263.1	5954548	5983038	chr7	18138486	18135320
2142	CP026263.1	5983144	6026680	chr7	18135210	18124311

2143	CP026263.1	6026780	6081069	chr7	18123895	18113239
2144	CP026263.1	6081563	6199761	chr7	18113086	18090321
2145	CP026263.1	6199879	6254665	chr7	18089503	18082241
2146	CP026263.1	6254772	6286591	chr7	18079947	18075577
2147	CP026263.1	6286684	6300731	chr7	18075391	18066148
2148	CP026263.1	6300924	6552046	chr7	18065026	18063050
2149	CP026263.1	6552216	6633823	chr7	18062258	18054903
2150	CP026263.1	6633931	6864204	chr7	18054423	18043198
2151	CP026263.1	6864362	6888309	chr7	18042963	18039413
2152	CP026263.1	6888532	7134121	chr7	18038879	18034936
2153	CP026263.1	7134350	7147140	chr7	18034634	18031307
2154	CP026263.1	7147903	7156878	chr7	18030422	18006387
2155	CP026263.1	7157397	7172133	chr7	18005673	17999791
2156	CP026263.1	7172682	7207366	chr7	17999332	17994637
2157	CP026263.1	7207608	7306553	chr7	17993266	17985535
2158	CP026263.1	7249449	7249696	chr7	17974288	17985434
2159	CP026263.1	7249698	7249943	chr7	17968959	17973944
2160	CP026263.1	7308297	7371624	chr7	17965144	17967501
2161	CP026263.1	7371809	7480272	chr7	17957262	17964468
2162	CP026263.1	7480620	7595068	chr7	17897134	17957162
2163	CP026263.1	7595272	7607459	chr7	17882827	17895911
2164	CP026263.1	7607568	7653667	chr7	17732682	17882455
2165	CP026263.1	7653882	7674771	chr7	17726764	17732347
2166	CP026263.1	7674925	7685115	chr7	17715239	17725012
2167	CP026263.1	7685485	7819389	chr7	17714293	17715037
2168	CP026263.1	7819603	7932102	chr7	17671107	17714110
2169	CP026263.1	7932926	8016453	chr7	17667031	17670331
2170	CP026263.1	8016780	8169504	chr7	17597008	17666850
2171	CP026263.1	8169640	8279380	chr7	17589184	17596676

2172	CP026263.1	8279613	8351410	chr7	17586176	17588841
2173	CP026263.1	8351521	8358610	chr7	17583360	17585813
2174	CP026263.1	8358772	8479468	chr7	17570879	17583259
2175	CP026263.1	8405467	8405591	chr7	17568666	17570781
2176	CP026263.1	8406328	8406452	chr7	17553305	17566989
2177	CP026263.1	8479559	8582034	chr7	17544519	17551010
2178	CP026263.1	8582177	8583015	chr7	17523392	17544324
2179	CP026263.1	8583145	8583899	chr7	17521963	17522625
2180	CP026263.1	8584045	8628486	chr7	17500126	17499824
2181	CP026263.1	8628650	8651495	chr7	17476875	17521800
2182	CP026263.1	8651721	8701577	chr7	17474297	17476770
2183	CP026263.1	8701859	8710567	chr7	17455581	17473932
2184	CP026263.1	8710707	8730185	chr7	17450199	17453778
2185	CP026263.1	8730404	8822986	chr7	17440347	17449353
2186	CP026263.1	8823340	8950671	chr7	17434771	17440245
2187	CP026263.1	8951297	9207563	chr7	17433125	17434670
2188	CP026263.1	9207969	9238299	chr7	17429094	17431337
2189	CP026263.1	9238612	9286775	chr7	17418921	17428556
2190	CP026263.1	9286951	9294548	chr7	17413961	17413122
2191	CP026263.1	9294824	9325613	chr7	17401783	17418465
2192	CP026263.1	9325709	9380800	chr7	17356436	17401404
2193	CP026263.1	9380900	9387932	chr7	17215940	17215840
2194	CP026263.1	9388271	9390740	chr7	17143830	17356253
2195	CP026263.1	9390839	9664655	chr7	17011997	17143695
2196	CP026263.1	9664963	9675436	chr7	16989380	17011892
2197	CP026263.1	9675784	9712061	chr7	16928526	16989245
2198	CP026263.1	9713806	9722569	chr7	16889102	16888597
2199	CP026263.1	9722849	9810757	chr7	16873251	16927827
2200	CP026263.1	9811126	9840550	chr7	16870390	16872716

2201	CP026263.1	9840860	9885694	chr7	16863348	16869650
2202	CP026263.1	9858471	9858750	chr7	16813347	16862832
2203	CP026263.1	9885810	9891389	chr7	16802161	16812979
2204	CP026263.1	9891644	9929751	chr7	16795116	16801791
2205	CP026263.1	9930732	9976412	chr7	16788846	16791012
2206	CP026263.1	9976549	10005974	chr7	16756453	16788591
2207	CP026263.1	10006209	10008802	chr7	16703741	16756198
2208	CP026263.1	10009129	10015260	chr7	16700949	16703547
2209	CP026263.1	10015351	10158247	chr7	16684087	16700717
2210	CP026263.1	10158731	10215245	chr7	16644990	16683951
2211	CP026263.1	10215368	10271428	chr7	16630352	16644534
2212	CP026263.1	10271771	10282137	chr7	16612762	16630090
2213	CP026263.1	10282588	10285636	chr7	16556520	16609411
2214	CP026263.1	10285952	10412023	chr7	16542321	16555774
2215	CP026263.1	10412413	10474413	chr7	16537383	16538721
2216	CP026263.1	10474772	10496542	chr7	16517712	16535733
2217	CP026263.1	10496750	10641323	chr7	16510674	16517296
2218	CP026263.1	10641426	10649603	chr7	16506242	16510356
2219	CP026263.1	10649691	10733941	chr7	16471264	16505325
2220	CP026263.1	10734123	10763424	chr7	16467613	16470954
2221	CP026263.1	10763589	10781907	chr7	16458195	16466511
2222	CP026263.1	10785154	10801303	chr7	16440263	16458083
2223	CP026263.1	10801558	10826324	chr7	16418107	16439202
2224	CP026263.1	10826498	10830925	chr7	16415396	16416663
2225	CP026263.1	10831278	10918619	chr7	16410310	16412336
2226	CP026263.1	10918888	11064246	chr7	16396385	16410090
2227	CP026263.1	11065336	11073567	chr7	16387299	16394245
2228	CP026263.1	11073777	11119480	chr7	16375295	16386516
2229	CP026263.1	11119964	11133960	chr7	16362700	16375121

2230	CP026263.1	11134353	11139480	chr7	16361751	16362577
2231	CP026263.1	11140052	11185807	chr7	16344418	16361182
2232	CP026263.1	11186032	11237174	chr7	16337748	16343560
2233	CP026263.1	11237280	11304672	chr7	16335863	16337437
2234	CP026263.1	11304794	11310112	chr7	16319697	16335567
2235	CP026263.1	11310336	11324944	chr7	16272824	16319502
2236	CP026263.1	11325189	11333121	chr7	16261044	16272575
2237	CP026263.1	11333207	11355434	chr7	16247575	16260928
2238	CP026263.1	11355550	11395645	chr7	16231479	16247269
2239	CP026263.1	11395835	11408111	chr7	16187562	16230234
2240	CP026263.1	11408221	11417592	chr7	16126605	16187402
2241	CP026263.1	11417775	11423179	chr7	16122714	16122603
2242	CP026263.1	11424313	11427094	chr7	16122603	16126198
2243	CP026263.1	11427280	11462460	chr7	16117356	16117201
2244	CP026263.1	11445434	11445754	chr7	16117095	16116940
2245	CP026263.1	11448723	11449043	chr7	16114671	16120753
2246	CP026263.1	11463464	11492898	chr7	16112415	16114281
2247	CP026263.1	11493440	11496340	chr7	16106635	16111935
2248	CP026263.1	11496630	11498788	chr7	16102414	16106218
2249	CP026263.1	11498937	11500554	chr7	16099183	16102168
2250	CP026263.1	11501430	11545463	chr7	16092610	16098931
2251	CP026263.1	11546264	11565413	chr7	16088263	16092283
2252	CP026263.1	11565609	11579244	chr7	16076126	16088148
2253	CP026263.1	11579835	11582393	chr7	16058563	16075777
2254	CP026263.1	11582760	11586634	chr7	16047554	16058397
2255	CP026263.1	11587528	11588107	chr7	15999085	16046583
2256	CP026263.1	11587528	11592316	chr7	15946369	15997559
2257	CP026263.1	11592753	11594624	chr7	15934905	15945594
2258	CP026263.1	11594938	11612348	chr7	15924031	15934686

2259	CP026263.1	11612910	11624754	chr7	15904883	15923892
2260	CP026263.1	11624843	11631861	chr7	15891091	15904436
2261	CP026263.1	11632160	11640185	chr7	15878363	15890980
2262	CP026263.1	11642903	11646223	chr7	15872080	15878209
2263	CP026263.1	11646362	11687716	chr7	15865665	15871681
2264	CP026263.1	11666635	11667377	chr7	15805602	15864359
2265	CP026263.1	11687825	11742594	chr7	15759911	15804959
2266	CP026263.1	11742984	11771144	chr7	15748533	15759593
2267	CP026263.1	11771736	11791603	chr7	15627950	15748357
2268	CP026263.1	11792618	11831389	chr7	15614577	15627635
2269	CP026263.1	11831481	11945949	chr7	15610301	15613965
2270	CP026263.1	11863851	11864331	chr7	15609304	15610189
2271	CP026263.1	11867875	11868355	chr7	15603328	15609082
2272	CP026263.1	11946056	12019708	chr7	15572900	15603239
2273	CP026263.1	11987576	11987776	chr7	15569690	15572809
2274	CP026263.1	11988457	11988657	chr7	15561724	15569572
2275	CP026263.1	12019898	12023115	chr7	15557131	15558296
2276	CP026263.1	12023203	12036718	chr7	15520295	15555184
2277	CP026263.1	12037890	12042404	chr7	15507117	15519910
2278	CP026263.1	12042517	12045167	chr7	15500265	15506932
2279	CP026263.1	12046850	12058119	chr7	15401506	15499057
2280	CP026263.1	12058264	12078941	chr7	15380675	15401352
2281	CP026263.1	12079095	12176646	chr7	15369261	15380530
2282	CP026263.1	12177854	12184521	chr7	15364928	15367578
2283	CP026263.1	12184706	12197499	chr7	15360301	15364815
2284	CP026263.1	12197884	12232773	chr7	15345614	15359129
2285	CP026263.1	12234720	12235885	chr7	15342309	15345526
2286	CP026263.1	12239313	12247161	chr7	15311068	15310868
2287	CP026263.1	12247279	12250398	chr7	15310187	15309987

2288	CP026263.1	12250489	12280828	chr7	15268467	15342119
2289	CP026263.1	12280917	12286671	chr7	15190766	15190286
2290	CP026263.1	12286893	12287778	chr7	15186742	15186262
2291	CP026263.1	12287890	12291554	chr7	15153892	15268360
2292	CP026263.1	12292166	12305224	chr7	15115029	15153800
2293	CP026263.1	12305539	12425946	chr7	15094147	15114014
2294	CP026263.1	12426122	12437182	chr7	15065395	15093555
2295	CP026263.1	12437500	12482548	chr7	15010236	15065005
2296	CP026263.1	12483191	12541948	chr7	14989788	14989046
2297	CP026263.1	12516462	12517362	chr7	14968773	15010127
2298	CP026263.1	12543254	12549270	chr7	14965314	14968634
2299	CP026263.1	12549669	12555798	chr7	14954571	14962596
2300	CP026263.1	12555952	12568569	chr7	14947254	14954272
2301	CP026263.1	12568680	12582025	chr7	14935321	14947165
2302	CP026263.1	12582472	12601481	chr7	14917349	14934759
2303	CP026263.1	12601620	12612275	chr7	14915164	14917035
2304	CP026263.1	12612494	12623183	chr7	14910518	14909939
2305	CP026263.1	12623958	12675148	chr7	14909939	14914727
2306	CP026263.1	12676674	12724172	chr7	14905171	14909045
2307	CP026263.1	12725143	12735986	chr7	14902246	14904804
2308	CP026263.1	12736152	12753366	chr7	14888020	14901655
2309	CP026263.1	12753715	12765737	chr7	14868675	14887824
2310	CP026263.1	12765852	12769872	chr7	14823841	14867874
2311	CP026263.1	12770199	12776520	chr7	14821348	14822965
2312	CP026263.1	12776772	12779757	chr7	14819041	14821199
2313	CP026263.1	12780003	12783807	chr7	14815851	14818751
2314	CP026263.1	12784224	12789524	chr7	14785875	14815309
2315	CP026263.1	12790004	12791870	chr7	14771454	14771134
2316	CP026263.1	12792260	12798342	chr7	14768165	14767845

2317	CP026263.1	12794529	12794684	chr7	14749691	14784871
2318	CP026263.1	12794790	12794901	chr7	14746724	14749505
2319	CP026263.1	12794790	12794945	chr7	14740186	14745590
2320	CP026263.1	12800192	12803787	chr7	14730632	14740003
2321	CP026263.1	12804194	12864991	chr7	14718246	14730522
2322	CP026263.1	12865151	12907823	chr7	14677961	14718056
2323	CP026263.1	12909068	12924858	chr7	14655618	14677845
2324	CP026263.1	12925164	12938517	chr7	14647600	14655532
2325	CP026263.1	12938633	12950164	chr7	14632747	14647355
2326	CP026263.1	12950413	12997091	chr7	14627205	14632523
2327	CP026263.1	12997286	13013156	chr7	14559691	14627083
2328	CP026263.1	13013452	13015026	chr7	14508443	14559585
2329	CP026263.1	13015337	13021149	chr7	14462463	14508218
2330	CP026263.1	13022007	13038771	chr7	14456764	14461891
2331	CP026263.1	13039340	13040166	chr7	14442375	14456371
2332	CP026263.1	13040289	13052710	chr7	14396188	14441891
2333	CP026263.1	13052884	13064105	chr7	14387747	14395978
2334	CP026263.1	13064888	13071834	chr7	14241299	14386657
2335	CP026263.1	13073974	13087679	chr7	14153689	14241030
2336	CP026263.1	13087899	13089925	chr7	14148909	14153336
2337	CP026263.1	13092985	13094252	chr7	14123969	14148735
2338	CP026263.1	13095696	13116791	chr7	14107565	14123714
2339	CP026263.1	13117852	13135672	chr7	14086000	14104318
2340	CP026263.1	13135784	13144100	chr7	14056534	14085835
2341	CP026263.1	13145202	13148543	chr7	13972102	14056352
2342	CP026263.1	13148853	13182914	chr7	13963837	13972014
2343	CP026263.1	13183831	13187945	chr7	13819161	13963734
2344	CP026263.1	13188263	13194885	chr7	13797183	13818953
2345	CP026263.1	13195301	13213322	chr7	13734824	13796824

2346	CP026263.1	13214972	13216310	chr7	13608363	13734434
2347	CP026263.1	13219910	13233363	chr7	13604999	13608047
2348	CP026263.1	13234109	13287000	chr7	13594182	13604548
2349	CP026263.1	13290351	13307679	chr7	13537779	13593839
2350	CP026263.1	13307941	13322123	chr7	13481142	13537656
2351	CP026263.1	13322579	13361540	chr7	13337762	13480658
2352	CP026263.1	13361676	13378306	chr7	13331540	13337671
2353	CP026263.1	13378538	13381136	chr7	13328620	13331213
2354	CP026263.1	13381330	13433787	chr7	13298960	13328385
2355	CP026263.1	13434042	13466180	chr7	13253143	13298823
2356	CP026263.1	13466435	13468601	chr7	13214055	13252162
2357	CP026263.1	13472705	13479380	chr7	13208221	13213800
2358	CP026263.1	13479750	13490568	chr7	13181161	13180882
2359	CP026263.1	13490936	13540421	chr7	13163271	13208105
2360	CP026263.1	13540937	13547239	chr7	13133537	13162961
2361	CP026263.1	13547979	13550305	chr7	13045260	13133168
2362	CP026263.1	13550840	13605416	chr7	13036217	13044980
2363	CP026263.1	13566186	13566691	chr7	12998195	13034472
2364	CP026263.1	13606115	13666834	chr7	12987374	12997847
2365	CP026263.1	13666969	13689481	chr7	12713250	12987066
2366	CP026263.1	13689586	13821284	chr7	12710682	12713151
2367	CP026263.1	13821419	14033842	chr7	12703311	12710343
2368	CP026263.1	14034025	14078993	chr7	12648120	12703211
2369	CP026263.1	14079372	14096054	chr7	12617235	12648024
2370	CP026263.1	14090711	14091550	chr7	12609362	12616959
2371	CP026263.1	14096510	14106145	chr7	12561023	12609186
2372	CP026263.1	14106683	14108926	chr7	12530380	12560710
2373	CP026263.1	14110714	14112259	chr7	12273708	12529974
2374	CP026263.1	14122055	14124508	chr7	12145751	12273082

2375	CP026263.1	14124871	14127536	chr7	12052815	12145397
2376	CP026263.1	14127879	14135371	chr7	12033118	12052596
2377	CP026263.1	14135703	14205545	chr7	12024270	12032978
2378	CP026263.1	14205726	14209026	chr7	11974132	12023988
2379	CP026263.1	14209802	14252805	chr7	11951061	11973906
2380	CP026263.1	14252988	14253732	chr7	11906456	11950897
2381	CP026263.1	14253934	14263707	chr7	11905556	11906310
2382	CP026263.1	14265459	14271042	chr7	11904588	11905426
2383	CP026263.1	14271377	14421150	chr7	11801970	11904445
2384	CP026263.1	14421522	14434606	chr7	11728863	11728739
2385	CP026263.1	14435829	14495857	chr7	11728002	11727878
2386	CP026263.1	14495957	14503163	chr7	11681183	11801879
2387	CP026263.1	14503839	14506196	chr7	11673932	11681021
2388	CP026263.1	14507654	14512639	chr7	11602024	11673821
2389	CP026263.1	14512983	14524129	chr7	11492051	11601791
2390	CP026263.1	14524230	14527165	chr7	11339191	11491915
2391	CP026263.1	14527551	14537000	chr7	11255337	11338864
2392	CP026263.1	14537602	14550004	chr7	11142014	11254513
2393	CP026263.1	14550142	14556586	chr7	11007896	11141800
2394	CP026263.1	14557604	14558980	chr7	10997336	11007526
2395	CP026263.1	14559130	14574189	chr7	10976293	10997182
2396	CP026263.1	14575573	14579416	chr7	10929979	10976078
2397	CP026263.1	14579943	14582585	chr7	10917683	10929870
2398	CP026263.1	14585614	14588812	chr7	10803031	10917479
2399	CP026263.1	14589343	14596579	chr7	10694220	10802683
2400	CP026263.1	14598467	14628167	chr7	10630708	10694035
2401	CP026263.1	14631313	14633973	chr7	10572354	10572109
2402	CP026263.1	14635376	14639403	chr7	10572107	10571860
2403	CP026263.1	14639807	14646362	chr7	10530019	10628964

2404	CP026263.1	14646474	14648303	chr7	10495093	10529777
2405	CP026263.1	14648761	14654033	chr7	10479808	10494544
2406	CP026263.1	14654577	14657062	chr7	10470314	10479289
2407	CP026263.1	14657596	14659471	chr7	10456761	10469551
2408	CP026263.1	14659567	14668342	chr7	10210943	10456532
2409	CP026263.1	14668730	14673498	chr7	10186773	10210720
2410	CP026263.1	14674519	14676872	chr7	9956342	10186615
2411	CP026263.1	14676998	14682649	chr7	9874627	9956234
2412	CP026263.1	14683204	14707730	chr7	9623335	9874457
2413	CP026263.1	14709176	14717845	chr7	9609095	9623142
2414	CP026263.1	14711883	14711989	chr7	9577183	9609002
2415	CP026263.1	14719383	14720984	chr7	9522290	9577076
2416	CP026263.1	14722534	14723889	chr7	9403974	9522172
2417	CP026263.1	14725448	14728639	chr7	9349191	9403480
2418	CP026263.1	14730873	14733489	chr7	9305555	9349091
2419	CP026263.1	14735275	14738207	chr7	9276959	9305449
2420	CP026263.1	14737825	14738207	chr7	9260672	9276295
2421	CP026263.1	14738883	14741962	chr7	9256135	9259470
2422	CP026263.1	14738951	14739333	chr7	9252306	9253950
2423	CP026263.1	14742124	14744003	chr7	9234606	9248756
2424	CP026263.1	14744485	14746505	chr7	9199638	9234301
2425	CP026263.1	14746711	14769744	chr7	9198093	9199498
2426	CP026263.1	14771453	14773152	chr7	9118579	9197954
2427	CP026263.1	14776267	14780148	chr7	9095174	9118266
2428	CP026263.1	14780855	14786134	chr7	9050060	9095074
2429	CP026263.1	14789366	14790890	chr7	9018688	9049933
2430	CP026263.1	14791169	14792448	chr7	8976227	9018587
2431	CP026263.1	14997094	14998959	chr7	8954542	8976013
2432	CP026263.1	15000016	15002353	chr7	8932452	8954442

2433	CP026263.1	15002826	15007474	chr7	8927874	8932299
2434	CP026263.1	15009332	15015214	chr7	8904965	8927496
2435	CP026263.1	15013915	15014056	chr7	8868038	8904671
2436	CP026263.1	15016331	15028924	chr7	8866184	8867917
2437	CP026263.1	15017364	15017505	chr7	8778052	8866072
2438	CP026263.1	15029395	15031415	chr7	8736631	8736277
2439	CP026263.1	15031870	15033549	chr7	8685888	8777959
2440	CP026263.1	15036265	15046373	chr7	8673208	8685714
2441	CP026263.1	15047075	15054655	chr7	8632524	8632193
2442	CP026263.1	15055492	15071077	chr7	8632221	8632059
2443	CP026263.1	15071610	15073385	chr7	8632057	8631726
2444	CP026263.1	15074453	15088729	chr7	8631724	8631112
2445	CP026263.1	15089599	15093153	chr7	8631141	8630979
2446	CP026263.1	15093297	15093530	chr7	8631010	8630398
2447	CP026263.1	15093697	15106753	chr7	8574896	8672425
2448	CP026263.1	15107583	15114211	chr7	8532790	8574800
2449	CP026263.1	15117380	15131051	chr7	8527371	8532562
2450	CP026263.1	15131663	15136715	chr7	8518436	8527201
2451	CP026263.1	15136836	15142301	chr7	8473578	8516370
2452	CP026263.1	15142871	15159202	chr7	8466253	8473471
2453	CP026263.1	15155193	15155964	chr7	8461264	8465935
2454	CP026263.1	15159428	15160558	chr7	8439889	8459981
2455	CP026263.1	15160743	15169900	chr7	8414951	8439729
2456	CP026263.1	15170185	15172151	chr7	8406735	8406152
2457	CP026263.1	15173454	15177683	chr7	8402356	8414788
2458	CP026263.1	15177550	15177682	chr7	8378895	8402065
2459	CP026263.1	15178727	15184215	chr7	8333603	8333321
2460	CP026263.1	15185353	15187849	chr7	8325282	8324991
2461	CP026263.1	15191236	15192583	chr7	8325021	8324718

2462	CP026263.1	15194763	15198838	chr7	8324749	8324458
2463	CP026263.1	15199121	15202928	chr7	8324456	8324153
2464	CP026263.1	15203724	15205425	chr7	8098869	8378702
2465	CP026263.1	15205831	15207519	chr7	8085796	8085676
2466	CP026263.1	15209423	15214025	chr7	8083758	8083638
2467	CP026263.1	15214527	15216141	chr7	7901360	8097458
2468	CP026263.1	15219093	15220839	chr7	7813895	7901240
2469	CP026263.1	15222253	15236174	chr7	7764068	7810528
2470	CP026263.1	15239232	15241151	chr7	7708807	7763879
2471	CP026263.1	15243796	15252520	chr7	7700453	7708564
2472	CP026263.1	15252685	15257448	chr7	7573076	7699931
2473	CP026263.1	15258831	15264237	chr7	7571593	7572885
2474	CP026263.1	15264494	15269122	chr7	7460604	7460180
2475	CP026263.1	15272431	15273678	chr7	7459835	7459411
2476	CP026263.1	15274082	15276952	chr7	7421317	7571349
2477	CP026263.1	15278748	15281092	chr7	7341781	7421006
2478	CP026263.1	15282412	15289835	chr7	7337398	7341557
2479	CP026263.1	15290897	15301464	chr7	6989295	7337276
2480	CP026263.1	15302101	15304304	chr7	6966967	6989186
2481	CP026263.1	15304557	15315299	chr7	6947610	6966829
2482	CP026263.1	15315890	15320860	chr7	6944699	6947261
2483	CP026263.1	15322337	15323948	chr7	6936572	6944415
2484	CP026263.1	15324932	15327037	chr7	6892167	6936437
2485	CP026263.1	15329111	15331437	chr7	6838923	6891816
2486	CP026263.1	15331643	15336493	chr7	6828905	6837454
2487	CP026263.1	15339555	15343749	chr7	6824722	6828755
2488	CP026263.1	15345771	15354697	chr7	6814964	6824632
2489	CP026263.1	15355899	15357947	chr7	6806068	6814751
2490	CP026263.1	15360412	15366089	chr7	6797016	6805941

2491	CP026263.1	15364084	15364562	chr7	6780629	6796861
2492	CP026263.1	15364757	15365235	chr7	6596836	6780448
2493	CP026263.1	15370795	15372293	chr7	6564477	6596393
2494	CP026263.1	15372914	15374615	chr7	6556472	6563903
2495	CP026263.1	15375717	15376985	chr7	6537549	6556321
2496	CP026263.1	15377508	15385804	chr7	6466481	6537449
2497	CP026263.1	15386217	15395033	chr7	6414124	6466358
2498	CP026263.1	15389385	15389834	chr7	6227890	6413545
2499	CP026263.1	15390067	15390401	chr7	6164847	6227582
2500	CP026263.1	15390069	15390518	chr7	6003029	6164658
2501	CP026263.1	15390520	15390854	chr7	5998698	6002668
2502	CP026263.1	15395147	15396045	chr7	5940603	5998607
2503	CP026263.1	15396772	15398309	chr7	5921158	5940483
2504	CP026263.1	15398655	15410247	chr7	5881804	5921042
2505	CP026263.1	15410370	15413536	chr7	5880134	5881350
2506	CP026263.1	15413646	15424545	chr7	5642366	5880010
2507	CP026263.1	15424961	15435617	chr7	5598331	5642278
2508	CP026263.1	15435770	15458535	chr7	5392568	5391511
2509	CP026263.1	15459353	15466615	chr7	5222215	5597953
2510	CP026263.1	15468909	15473279	chr7	5201676	5221952
2511	CP026263.1	15473465	15482708	chr7	5066822	5201423
2512	CP026263.1	15483830	15485806	chr7	5056046	5066710
2513	CP026263.1	15486598	15493953	chr7	5046214	5055725
2514	CP026263.1	15494433	15505658	chr7	5003686	5045993
2515	CP026263.1	15505893	15509443	chr7	4882235	5003549
2516	CP026263.1	15509977	15513920	chr7	4755822	4881766
2517	CP026263.1	15514222	15517549	chr7	4601762	4755353
2518	CP026263.1	15518434	15542469	chr7	4473351	4601659
2519	CP026263.1	15543183	15549065	chr7	4466080	4473119

2520	CP026263.1	15549524	15554219	chr7	4433152	4465809
2521	CP026263.1	15555590	15563321	chr7	4364783	4433038
2522	CP026263.1	15734482	15739956	chr7	3543764	3542864
2523	CP026263.1	15740058	15749064	chr7	3534646	3534540
2524	CP026263.1	15749910	15753489	chr7	1041761	1024653
2525	CP026263.1	15755292	15773643	chr7	1024561	1015920
2526	CP026263.1	15774008	15776481	chr7	1015683	971187
2527	CP026263.1	15776586	15821511	chr7	970993	924855
2528	CP026263.1	15799535	15799837	chr7	924726	704193
2529	CP026263.1	15821674	15822336	chr7	704059	328018
2530	CP026263.1	15823103	15844035	chr7	326683	306741
2531	CP026263.1	15844230	15850721	chr7	306534	98887
2532	CP026263.1	15853016	15866700	chr7	193678	193797
2533	CP026263.1	15868377	15870492	chr7	98380	94974
2534	CP026263.1	15870590	15882970	chr7	94870	1
2535	CP026244.1	38331	44190	chr8	23021537	23153220
2536	CP026244.1	44540	52458	chr8	22989545	23021217
2537	CP026244.1	53026	62184	chr8	22927027	22989249
2538	CP026244.1	62377	77799	chr8	22909169	22926836
2539	CP026244.1	84247	96921	chr8	22873752	22909000
2540	CP026244.1	97022	115489	chr8	22872469	22873658
2541	CP026244.1	115678	138527	chr8	22865219	22871184
2542	CP026244.1	139497	158512	chr8	22850127	22865132
2543	CP026244.1	158762	177575	chr8	22750497	22849762
2544	CP026244.1	181314	198421	chr8	22745341	22750284
2545	CP026244.1	198737	204017	chr8	22745240	22737669
2546	CP026244.1	205352	211049	chr8	22737517	22734422
2547	CP026244.1	212179	215507	chr8	22733957	22701681
2548	CP026244.1	216698	228434	chr8	22700458	22698427

2549	CP026244.1	229391	235883	chr8	22698073	22690645
2550	CP026244.1	237697	254128	chr8	22690349	22682213
2551	CP026244.1	254541	257293	chr8	22680429	22669209
2552	CP026244.1	258010	265056	chr8	22668773	22660007
2553	CP026244.1	265275	267223	chr8	22658879	22637048
2554	CP026244.1	268626	270186	chr8	22636353	22593215
2555	CP026244.1	272124	273760	chr8	22592615	22590970
2556	CP026244.1	275120	282833	chr8	22590727	22587242
2557	CP026244.1	284499	286912	chr8	22587077	22556456
2558	CP026244.1	287961	289906	chr8	22556289	22541579
2559	CP026244.1	291181	292680	chr8	22541248	22519094
2560	CP026244.1	293751	297987	chr8	22518906	22517257
2561	CP026244.1	300994	305740	chr8	22517123	22500150
2562	CP026244.1	306663	308225	chr8	22499761	22497433
2563	CP026244.1	308745	323484	chr8	22497315	22491926
2564	CP026244.1	324506	328719	chr8	22491471	22474519
2565	CP026244.1	332823	335043	chr8	22474429	22467477
2566	CP026244.1	339648	346316	chr8	22467191	22449503
2567	CP026244.1	349851	352650	chr8	22449277	22428380
2568	CP026244.1	353170	365034	chr8	22427795	22424574
2569	CP026244.1	365300	372228	chr8	22424286	22414742
2570	CP026244.1	373463	379002	chr8	22414656	22393399
2571	CP026244.1	379276	385348	chr8	22393274	22389801
2572	CP026244.1	390077	398733	chr8	22388101	22381207
2573	CP026244.1	399311	400580	chr8	22380687	22376346
2574	CP026244.1	401371	405281	chr8	22375901	22374437
2575	CP026244.1	405468	406769	chr8	22373666	22364810
2576	CP026244.1	408797	410640	chr8	22364713	22363091
2577	CP026244.1	475137	486882	chr8	22361350	22358745

2578	CP026244.1	489225	491199	chr8	22354372	22350945
2579	CP026244.1	494717	499743	chr8	22349569	22347165
2580	CP026244.1	500415	502863	chr8	22346653	22338740
2581	CP026244.1	503157	505416	chr8	22336586	22312477
2582	CP026244.1	508952	512502	chr8	22312129	22275433
2583	CP026244.1	512992	520551	chr8	22275289	22272653
2584	CP026244.1	521477	552532	chr8	22267384	22248156
2585	CP026244.1	556076	560530	chr8	22248047	22246622
2586	CP026244.1	562742	585362	chr8	22246622	22101835
2587	CP026244.1	585841	599935	chr8	22101450	22034832
2588	CP026244.1	600141	625793	chr8	22033460	22024171
2589	CP026244.1	626611	647326	chr8	22023805	22021592
2590	CP026244.1	632798	634082	chr8	22021373	22000592
2591	CP026244.1	647618	651820	chr8	21999561	21995821
2592	CP026244.1	652095	676969	chr8	21995715	21981595
2593	CP026244.1	677762	679455	chr8	21981329	21980206
2594	CP026244.1	679620	704461	chr8	21979849	21978141
2595	CP026244.1	704687	713280	chr8	21978014	21944361
2596	CP026244.1	713558	722643	chr8	21944095	21928391
2597	CP026244.1	722974	748278	chr8	21927172	21890742
2598	CP026244.1	748774	752454	chr8	21890650	21855259
2599	CP026244.1	752727	755384	chr8	21868015	21869278
2600	CP026244.1	755905	758441	chr8	21854721	21849419
2601	CP026244.1	761727	763195	chr8	21849277	21816370
2602	CP026244.1	765846	776014	chr8	21816138	21804941
2603	CP026244.1	776209	815383	chr8	21804763	21800372
2604	CP026244.1	816758	884853	chr8	21800024	21785196
2605	CP026244.1	885447	907449	chr8	21784965	21745835
2606	CP026244.1	908109	955954	chr8	21745649	21741593

2607	CP026244.1	956059	1028444	chr8	21741349	21737371
2608	CP026244.1	1028810	1031945	chr8	21737264	21704255
2609	CP026244.1	1032840	1034853	chr8	21703472	21702173
2610	CP026244.1	1035506	1045116	chr8	21700468	21694565
2611	CP026244.1	1045671	1047227	chr8	21694440	21682317
2612	CP026244.1	1048487	1050567	chr8	21682101	21643515
2613	CP026244.1	1051760	1059256	chr8	21643171	21619084
2614	CP026244.1	1061456	1088379	chr8	21618963	21617161
2615	CP026244.1	1089638	1119531	chr8	21616660	21596733
2616	CP026244.1	1120649	1131311	chr8	21594849	21583486
2617	CP026244.1	1131660	1133595	chr8	21583338	21574918
2618	CP026244.1	1134674	1138772	chr8	21574674	21543468
2619	CP026244.1	1142547	1143897	chr8	21541072	21536732
2620	CP026244.1	1144888	1149355	chr8	21534102	21505943
2621	CP026244.1	1149705	1181579	chr8	21502803	21501090
2622	CP026244.1	1182646	1197808	chr8	21497390	21485116
2623	CP026244.1	1198336	1204681	chr8	21484827	21480570
2624	CP026244.1	1204852	1209538	chr8	21479935	21473678
2625	CP026244.1	1210941	1239587	chr8	21473350	21466425
2626	CP026244.1	1242578	1244499	chr8	21466260	21463978
2627	CP026244.1	1245039	1246457	chr8	21463857	21461691
2628	CP026244.1	1247230	1254594	chr8	21460882	21445689
2629	CP026244.1	1255238	1262695	chr8	21445502	21443003
2630	CP026244.1	1263265	1269268	chr8	21442773	21434383
2631	CP026244.1	1269400	1271909	chr8	21433389	21429782
2632	CP026244.1	1272271	1294873	chr8	21429506	21425174
2633	CP026244.1	1295901	1305213	chr8	21424428	21421185
2634	CP026244.1	1307504	1308787	chr8	21419932	21418000
2635	CP026244.1	1311428	1327517	chr8	21414849	21410427

2636	CP026244.1	1328746	1334016	chr8	21410305	21402181
2637	CP026244.1	1334647	1337096	chr8	21400918	21388485
2638	CP026244.1	1339118	1362314	chr8	21387559	21377016
2639	CP026244.1	1362791	1371310	chr8	21376878	21373215
2640	CP026244.1	1373152	1381741	chr8	21371847	21370469
2641	CP026244.1	1384206	1386047	chr8	21370193	21350016
2642	CP026244.1	1386879	1389770	chr8	21349911	21347250
2643	CP026244.1	1391393	1393306	chr8	21346681	21336314
2644	CP026244.1	1393701	1398396	chr8	21336212	21325186
2645	CP026244.1	1398713	1419388	chr8	21324005	21299627
2646	CP026244.1	1419862	1424892	chr8	21299541	21296898
2647	CP026244.1	1426540	1431648	chr8	21296726	21295404
2648	CP026244.1	1433038	1438453	chr8	21294421	21236013
2649	CP026244.1	1439051	1457303	chr8	21235889	21228975
2650	CP026244.1	1457955	1460718	chr8	21225253	21223678
2651	CP026244.1	1460851	1461894	chr8	21218323	21211707
2652	CP026244.1	1462410	1468220	chr8	21211101	21207259
2653	CP026244.1	1468723	1474585	chr8	21206978	21203530
2654	CP026244.1	1476134	1478336	chr8	21203400	21195706
2655	CP026244.1	1483897	1503087	chr8	21191946	21189568
2656	CP026244.1	1503566	1508595	chr8	21186620	21044255
2657	CP026244.1	1508939	1512553	chr8	21044131	21031862
2658	CP026244.1	1512654	1519008	chr8	21031621	20987914
2659	CP026244.1	1519437	1523170	chr8	20985933	20939754
2660	CP026244.1	1523517	1525620	chr8	20939374	20935194
2661	CP026244.1	1528438	1553004	chr8	20935081	20932271
2662	CP026244.1	1554190	1570266	chr8	20932146	20910814
2663	CP026244.1	1572723	1578609	chr8	20910579	20892645
2664	CP026244.1	1578713	1585505	chr8	20891712	20882680

2665	CP026244.1	1585619	1611388	chr8	20881871	20879437
2666	CP026244.1	1612372	1616443	chr8	20878667	20828628
2667	CP026244.1	1616736	1618164	chr8	20828117	20767259
2668	CP026244.1	1618888	1629468	chr8	20765639	20746561
2669	CP026244.1	1630848	1654667	chr8	20746343	20721578
2670	CP026244.1	1654964	1657779	chr8	20721114	20711063
2671	CP026244.1	1659576	1661166	chr8	20710967	20706576
2672	CP026244.1	1661278	1674273	chr8	20706425	20702243
2673	CP026244.1	1674532	1707383	chr8	20701732	20699607
2674	CP026244.1	1707709	1731969	chr8	20698880	20692735
2675	CP026244.1	1738254	1815142	chr8	20692502	20672104
2676	CP026244.1	1815555	1821791	chr8	20671993	20646836
2677	CP026244.1	1823010	1837568	chr8	20646728	20618025
2678	CP026244.1	1838231	1843383	chr8	20617828	20570793
2679	CP026244.1	1843504	1850537	chr8	20570687	20512282
2680	CP026244.1	1851047	1872309	chr8	20512172	20492860
2681	CP026244.1	1872791	1875906	chr8	20492614	20478991
2682	CP026244.1	1876180	1879566	chr8	20475891	20474479
2683	CP026244.1	1880098	1887290	chr8	20472776	20471531
2684	CP026244.1	1887557	1892541	chr8	20471173	20457554
2685	CP026244.1	1892869	1895335	chr8	20457040	20454427
2686	CP026244.1	1895541	1896717	chr8	20454311	20447041
2687	CP026244.1	1896812	1898755	chr8	20446818	20441857
2688	CP026244.1	1899081	1932347	chr8	20441768	20435234
2689	CP026244.1	1932458	1966556	chr8	20434844	20427704
2690	CP026244.1	1970071	2032311	chr8	20427505	20376965
2691	CP026244.1	2033252	2071235	chr8	20376657	20360849
2692	CP026244.1	2073259	2151165	chr8	20360694	20334117
2693	CP026244.1	2152054	2155663	chr8	20333122	20292454

2694	CP026244.1	2156111	2159289	chr8	20292186	20281099
2695	CP026244.1	2159842	2167141	chr8	20280568	20276201
2696	CP026244.1	2167512	2168879	chr8	20276025	20267936
2697	CP026244.1	2169394	2173073	chr8	20267814	20256414
2698	CP026244.1	2173212	2175151	chr8	20256007	20254277
2699	CP026244.1	2175309	2182510	chr8	20254069	20251600
2700	CP026244.1	2182752	2186187	chr8	20251294	20211244
2701	CP026244.1	2187570	2191073	chr8	20210977	20208974
2702	CP026244.1	2192281	2197889	chr8	20207979	20205902
2703	CP026244.1	2198141	2206511	chr8	20205619	20181020
2704	CP026244.1	2206618	2242432	chr8	20180773	20174037
2705	CP026244.1	2242538	2251720	chr8	20173525	20155968
2706	CP026244.1	2253393	2262162	chr8	20154625	20151351
2707	CP026244.1	2262920	2305520	chr8	20146521	20151250
2708	CP026244.1	2305946	2325621	chr8	20122271	20146162
2709	CP026244.1	2325947	2352924	chr8	20112090	20122170
2710	CP026244.1	2353122	2359681	chr8	20103554	20109783
2711	CP026244.1	2361232	2366169	chr8	20083946	20103279
2712	CP026244.1	2367524	2381726	chr8	20081015	20083220
2713	CP026244.1	2383105	2385643	chr8	20066035	20080495
2714	CP026244.1	2385757	2387547	chr8	20034391	20064357
2715	CP026244.1	2388309	2398024	chr8	19997168	20034254
2716	CP026244.1	2398156	2399963	chr8	19993311	19996558
2717	CP026244.1	2400093	2429296	chr8	19982324	19992775
2718	CP026244.1	2429512	2457648	chr8	19967469	19981637
2719	CP026244.1	2457857	2466043	chr8	19959797	19967068
2720	CP026244.1	2466142	2488550	chr8	19952967	19957573
2721	CP026244.1	2489091	2513957	chr8	19949023	19952781
2722	CP026244.1	2514051	2521645	chr8	19945452	19947864

2723	CP026244.1	2522595	2544968	chr8	19920636	19944740
2724	CP026244.1	2545105	2564513	chr8	19911394	19920111
2725	CP026244.1	2564716	2587661	chr8	19897658	19910730
2726	CP026244.1	2587859	2602172	chr8	19894544	19896602
2727	CP026244.1	2603566	2639047	chr8	19891015	19893994
2728	CP026244.1	2639339	2664206	chr8	19889162	19890634
2729	CP026244.1	2664363	2680648	chr8	19866000	19888609
2730	CP026244.1	2682185	2706514	chr8	19865899	19852519
2731	CP026244.1	2706620	2725626	chr8	19852269	19848884
2732	CP026244.1	2729234	2735195	chr8	19848233	19832774
2733	CP026244.1	2735364	2770355	chr8	19832547	19830365
2734	CP026244.1	2771893	2773687	chr8	19829215	19827669
2735	CP026244.1	2775116	2784412	chr8	19824777	19804190
2736	CP026244.1	2784877	2800537	chr8	19802154	19798555
2737	CP026244.1	2800627	2809044	chr8	19797558	19788704
2738	CP026244.1	2809421	2812691	chr8	19787877	19785573
2739	CP026244.1	2812996	2814702	chr8	19784539	19775322
2740	CP026244.1	2814819	2818699	chr8	19774788	19772368
2741	CP026244.1	2819039	2822472	chr8	19771320	19766604
2742	CP026244.1	2823048	2829282	chr8	19766391	19748693
2743	CP026244.1	2829387	2845472	chr8	19747028	19733029
2744	CP026244.1	2845573	2853144	chr8	19732480	19729416
2745	CP026244.1	2853296	2856391	chr8	19729201	19726745
2746	CP026244.1	2856856	2889132	chr8	19726241	19709932
2747	CP026244.1	2890355	2892386	chr8	19709356	19706128
2748	CP026244.1	2892740	2900168	chr8	19705248	19681996
2749	CP026244.1	2900464	2908600	chr8	19681904	19680709
2750	CP026244.1	2910384	2921604	chr8	19679366	19660763
2751	CP026244.1	2922040	2930806	chr8	19659389	19655745

2752	CP026244.1	2931934	2953765	chr8	19655588	19650350
2753	CP026244.1	2954460	2997598	chr8	19649928	19648762
2754	CP026244.1	2998198	2999843	chr8	19647864	19643606
2755	CP026244.1	3000086	3003571	chr8	19643473	19618577
2756	CP026244.1	3003736	3034357	chr8	19618081	19589834
2757	CP026244.1	3034524	3049234	chr8	19588697	19579866
2758	CP026244.1	3049565	3071719	chr8	19557511	19579765
2759	CP026244.1	3071907	3073556	chr8	19549602	19557323
2760	CP026244.1	3073690	3090663	chr8	19548407	19549498
2761	CP026244.1	3091052	3093380	chr8	19544023	19547312
2762	CP026244.1	3093498	3098887	chr8	19540715	19543638
2763	CP026244.1	3099342	3116294	chr8	19534194	19538478
2764	CP026244.1	3116384	3123336	chr8	19530386	19533993
2765	CP026244.1	3123622	3141310	chr8	19521031	19530123
2766	CP026244.1	3141536	3162433	chr8	19518240	19520906
2767	CP026244.1	3163018	3166239	chr8	19512550	19518119
2768	CP026244.1	3166527	3176071	chr8	19508651	19512285
2769	CP026244.1	3176157	3197414	chr8	19492585	19507679
2770	CP026244.1	3197539	3201012	chr8	19488609	19492285
2771	CP026244.1	3202712	3209606	chr8	19484854	19488468
2772	CP026244.1	3210126	3214467	chr8	19476260	19484668
2773	CP026244.1	3214912	3216376	chr8	19467794	19475051
2774	CP026244.1	3217147	3226003	chr8	19465206	19467400
2775	CP026244.1	3226100	3227722	chr8	19461081	19465111
2776	CP026244.1	3229463	3232068	chr8	19445745	19460177
2777	CP026244.1	3236441	3239868	chr8	19441994	19445604
2778	CP026244.1	3241244	3243648	chr8	19377261	19441862
2779	CP026244.1	3244160	3252073	chr8	19374993	19377121
2780	CP026244.1	3254227	3278336	chr8	19336984	19374035

2781	CP026244.1	3278684	3315380	chr8	19316165	19336132
2782	CP026244.1	3315524	3318160	chr8	19309043	19315587
2783	CP026244.1	3323429	3342657	chr8	19307098	19308708
2784	CP026244.1	3342766	3344191	chr8	19301667	19306891
2785	CP026244.1	3344292	3367925	chr8	19293173	19300182
2786	CP026244.1	3368446	3414197	chr8	19263608	19292636
2787	CP026244.1	3414309	3430672	chr8	19250281	19263307
2788	CP026244.1	3431084	3463710	chr8	19241312	19250067
2789	CP026244.1	3464011	3465978	chr8	19229173	19240184
2790	CP026244.1	3466137	3481644	chr8	19226123	19228908
2791	CP026244.1	3488968	3492789	chr8	19214849	19225792
2792	CP026244.1	3492905	3588585	chr8	19211219	19213824
2793	CP026244.1	3588885	3674844	chr8	19205969	19210159
2794	CP026244.1	3675288	3686632	chr8	19204459	19205571
2795	CP026244.1	3686850	3690768	chr8	19192441	19204323
2796	CP026244.1	3691001	3701897	chr8	19187316	19192297
2797	CP026244.1	3702073	3762522	chr8	19171125	19187212
2798	CP026244.1	3762649	3828166	chr8	19167675	19170515
2799	CP026244.1	3830045	3874335	chr8	19090496	19167397
2800	CP026244.1	3877866	3880454	chr8	19081466	19090234
2801	CP026244.1	3882701	3896857	chr8	19068757	19081312
2802	CP026244.1	3897136	3922565	chr8	19063187	19067424
2803	CP026244.1	3922693	3936072	chr8	19025928	19062772
2804	CP026244.1	3936597	3937737	chr8	18894741	19025362
2805	CP026244.1	3937969	3945134	chr8	18890535	18894347
2806	CP026244.1	3946748	3962898	chr8	18878135	18890224
2807	CP026244.1	3962999	3975879	chr8	18872645	18877979
2808	CP026244.1	3976094	4049131	chr8	18862963	18864885
2809	CP026244.1	4049526	4054170	chr8	18861285	18862423

2810	CP026244.1	4055038	4113367	chr8	18856861	18860708
2811	CP026244.1	4113512	4135602	chr8	18844718	18855514
2812	CP026244.1	4136973	4151292	chr8	18837567	18844017
2813	CP026244.1	4151677	4162718	chr8	18826721	18837389
2814	CP026244.1	4162966	4167489	chr8	18821920	18826144
2815	CP026244.1	4168956	4170731	chr8	18820291	18821594
2816	CP026244.1	4172009	4176644	chr8	18792868	18819493
2817	CP026244.1	4178483	4180044	chr8	18748919	18792101
2818	CP026244.1	4180381	4187032	chr8	18740353	18748818
2819	CP026244.1	4187990	4191873	chr8	18730167	18738793
2820	CP026244.1	4192916	4244307	chr8	18702530	18729514
2821	CP026244.1	4244633	4257169	chr8	18691034	18702310
2822	CP026244.1	4257315	4260134	chr8	18669310	18690682
2823	CP026244.1	4262287	4266945	chr8	18645164	18668910
2824	CP026244.1	4267538	4275229	chr8	18638557	18644517
2825	CP026244.1	4275425	4276709	chr8	18636982	18638457
2826	CP026244.1	4279873	4283296	chr8	18634526	18636713
2827	CP026244.1	4286962	4288606	chr8	18629847	18634158
2828	CP026244.1	4290093	4294900	chr8	18623690	18629440
2829	CP026244.1	4295074	4306433	chr8	18619442	18623565
2830	CP026244.1	4307619	4310690	chr8	18611170	18617706
2831	CP026244.1	4311104	4339538	chr8	18611069	18607830
2832	CP026244.1	4339826	4342765	chr8	18605568	18590428
2833	CP026244.1	4342865	4378507	chr8	18589756	18563666
2834	CP026244.1	4379077	4390548	chr8	18562503	18543302
2835	CP026244.1	4390649	4510709	chr8	18543131	18540891
2836	CP026244.1	4511050	4627341	chr8	18540461	18537617
2837	CP026244.1	4627428	4663684	chr8	18537177	18529244
2838	CP026244.1	4663775	4672628	chr8	18528246	18516294

2839	CP026244.1	4673273	4742071	chr8	18515949	18502786
2840	CP026244.1	4742157	4746211	chr8	18502207	18496901
2841	CP026244.1	4746364	4867519	chr8	18496184	18475660
2842	CP026244.1	4867695	4954182	chr8	18475356	18471130
2843	CP026244.1	4954321	5067664	chr8	18470267	18446630
2844	CP026244.1	5067831	5077164	chr8	18446529	18439589
2845	CP026244.1	5077354	5115721	chr8	18439458	18112376
2846	CP026244.1	5116275	5128746	chr8	18111890	18062062
2847	CP026244.1	5128876	5144422	chr8	18061929	18060145
2848	CP026244.1	5144668	5151243	chr8	18059836	18009118
2849	CP026244.1	5151466	5153423	chr8	18008817	17994061
2850	CP026244.1	5155246	5187814	chr8	17993775	17914575
2851	CP026244.1	5189606	5199004	chr8	17914267	17794764
2852	CP026244.1	5199362	5208078	chr8	17794461	17717056
2853	CP026244.1	5208247	5210964	chr8	17716935	17706034
2854	CP026244.1	5211164	5241220	chr8	17705932	17654002
2855	CP026244.1	5242990	5257761	chr8	17653901	17639105
2856	CP026244.1	5258058	5271332	chr8	17638997	17630909
2857	CP026244.1	5271434	5289028	chr8	17629205	17563336
2858	CP026244.1	5289262	5305971	chr8	17563227	17423844
2859	CP026244.1	5306472	5308641	chr8	17423666	17367801
2860	CP026244.1	5311371	5359643	chr8	17367629	17263050
2861	CP026244.1	5359783	5396536	chr8	17262943	17202280
2862	CP026244.1	5397043	5405570	chr8	17202177	17180170
2863	CP026244.1	5405743	5433232	chr8	17180079	17033682
2864	CP026244.1	5433545	5470798	chr8	17033419	17012007
2865	CP026244.1	5474515	5481672	chr8	17011661	16987038
2866	CP026244.1	5482668	5490344	chr8	16986600	16984651
2867	CP026244.1	5490437	5530084	chr8	16984447	16917666

2868	CP026244.1	5530241	5534539	chr8	16917557	16913513
2869	CP026244.1	5534795	5537019	chr8	16913407	16860396
2870	CP026244.1	5538022	5554479	chr8	16860205	16841908
2871	CP026244.1	5555045	5560046	chr8	16841755	16758530
2872	CP026244.1	5561055	5573993	chr8	16758290	16754924
2873	CP026244.1	5574128	5599123	chr8	16754665	16730170
2874	CP026244.1	5599557	5745593	chr8	16730067	16725988
2875	CP026244.1	5745803	5752055	chr8	16725359	16722830
2876	CP026244.1	5752263	5840838	chr8	16722456	16698192
2877	CP026244.1	5841208	5883105	chr8	16697890	16654192
2878	CP026244.1	5883208	5889814	chr8	16653547	16642359
2879	CP026244.1	5890071	5934766	chr8	16642245	16628550
2880	CP026244.1	5936166	6129024	chr8	16627603	16510513
2881	CP026244.1	6129793	6131961	chr8	16509916	16505371
2882	CP026244.1	6132062	6435740	chr8	16505285	16489345
2883	CP026244.1	6435848	7000396	chr8	16489241	16440975
2884	CP026244.1	7000525	7069600	chr8	16440802	16338717
2885	CP026244.1	7069720	7275084	chr8	16338294	16293651
2886	CP026244.1	7276254	7487220	chr8	16293547	16281531
2887	CP026244.1	7491142	7666366	chr8	16281350	16278827
2888	CP026244.1	7666985	7679186	chr8	16278534	16192060
2889	CP026244.1	7680076	7682272	chr8	16191698	15965408
2890	CP026244.1	7682408	7685364	chr8	15965268	15952046
2891	CP026244.1	7685493	7755671	chr8	15951808	15937199
2892	CP026244.1	7755806	7761207	chr8	15936864	15595851
2893	CP026244.1	7761444	7795272	chr8	15595463	15576662
2894	CP026244.1	7795373	8185769	chr8	15576320	15551300
2895	CP026244.1	8185875	8340036	chr8	15550473	15512968
2896	CP026244.1	8340892	8388023	chr8	15512765	15481899

2897	CP026244.1	8388343	8403962	chr8	15481786	15478411
2898	CP026244.1	8404096	8456430	chr8	15476468	15360645
2899	CP026244.1	8456570	8483415	chr8	15360215	15260291
2900	CP026244.1	8483610	8495058	chr8	15259946	15131990
2901	CP026244.1	8495218	8637041	chr8	15131756	15117444
2902	CP026244.1	8637127	8662586	chr8	15117348	15090069
2903	CP026244.1	8662816	8663949	chr8	15089915	15037365
2904	CP026244.1	8664069	8796565	chr8	15037042	15032113
2905	CP026244.1	8796843	8883741	chr8	15031872	14996659
2906	CP026244.1	8883842	9035946	chr8	14996536	14977158
2907	CP026244.1	9036249	9123220	chr8	14976999	14970000
2908	CP026244.1	9123331	9305538	chr8	14969808	14827633
2909	CP026244.1	9306304	9506848	chr8	14827526	14661961
2910	CP026244.1	9507036	9676012	chr8	14661816	14563625
2911	CP026244.1	9676120	9742476	chr8	14563262	14558204
2912	CP026244.1	9742576	9836455	chr8	14557936	14430180
2913	CP026244.1	9836708	9896734	chr8	14356155	14430079
2914	CP026244.1	9896909	9963112	chr8	14347416	14356007
2915	CP026244.1	9963798	10346533	chr8	14276239	14346144
2916	CP026244.1	10346625	10394210	chr8	14194404	14275965
2917	CP026244.1	10394401	10491001	chr8	14140538	14194142
2918	CP026244.1	10491376	10534377	chr8	14076439	14140313
2919	CP026244.1	10534478	10560221	chr8	14068657	14076251
2920	CP026244.1	10562732	10567958	chr8	14046409	14067509
2921	CP026244.1	11009567	11099014	chr8	14044176	14046303
2922	CP026244.1	11099369	11288278	chr8	14042481	14043752
2923	CP026244.1	11288419	11291465	chr8	14030584	14041378
2924	CP026244.1	11294493	11314989	chr8	14010493	14030401
2925	CP026244.1	11316226	11350296	chr8	13977254	14009711

2926	CP026244.1	11350461	11426590	chr8	13909451	13976922
2927	CP026244.1	11428721	11459397	chr8	13828241	13909255
2928	CP026244.1	11459579	11486597	chr8	13821744	13827312
2929	CP026244.1	11489804	11562211	chr8	13785303	13821635
2930	CP026244.1	11563166	11640131	chr8	13728599	13785028
2931	CP026244.1	11640320	11643413	chr8	13591083	13728476
2932	CP026244.1	11643774	11789955	chr8	13512380	13590862
2933	CP026244.1	11790041	12031436	chr8	13486179	13511836
2934	CP026244.1	12031572	12046569	chr8	13448363	13485933
2935	CP026244.1	12047481	12063276	chr8	13380811	13448064
2936	CP026244.1	12063546	12064752	chr8	13291630	13380653
2937	CP026244.1	12065472	12107401	chr8	13287789	13290571
2938	CP026244.1	12107509	12170548	chr8	13253806	13287679
2939	CP026244.1	12172317	12211174	chr8	13029098	13253600
2940	CP026244.1	12211316	12427653	chr8	13025707	13028945
2941	CP026244.1	12428177	12449471	chr8	12954570	13025587
2942	CP026244.1	12450055	12452420	chr8	12904309	12954269
2943	CP026244.1	12452573	12468917	chr8	12874770	12903671
2944	CP026244.1	12469033	12504780	chr8	12747538	12874596
2945	CP026244.1	12504908	12648973	chr8	12723951	12747324
2946	CP026244.1	12649070	12656037	chr8	12692909	12723831
2947	CP026244.1	12656235	12680786	chr8	12681948	12692621
2948	CP026244.1	12680874	12725100	chr8	12637388	12680677
2949	CP026244.1	12725368	12774306	chr8	12606084	12637080
2950	CP026244.1	12774429	12785678	chr8	12578998	12605803
2951	CP026244.1	12786156	12789363	chr8	12570240	12578810
2952	CP026244.1	12789745	12794387	chr8	12532626	12569813
2953	CP026244.1	12794488	12812571	chr8	12522342	12532368
2954	CP026244.1	12812855	12826260	chr8	12471254	12522074

2955	CP026244.1	12826376	12832658	chr8	12460220	12470702
2956	CP026244.1	12832767	12864149	chr8	12430333	12459186
2957	CP026244.1	12864421	12904072	chr8	12424996	12430139
2958	CP026244.1	12904323	12930349	chr8	12415763	12424617
2959	CP026244.1	12930829	12965990	chr8	12408653	12415379
2960	CP026244.1	12966960	13037989	chr8	12322597	12408408
2961	CP026244.1	13039080	13041219	chr8	12232438	12321514
2962	CP026244.1	13041398	13114915	chr8	12198776	12232314
2963	CP026244.1	13115022	13129357	chr8	12191250	12198632
2964	CP026244.1	13129448	13137026	chr8	12170964	12190892
2965	CP026244.1	13137338	13148536	chr8	12142973	12170792
2966	CP026244.1	13149372	13177191	chr8	12130939	12142137
2967	CP026244.1	13177363	13197291	chr8	12123049	12130627
2968	CP026244.1	13197649	13205031	chr8	12108623	12122958
2969	CP026244.1	13205175	13238713	chr8	12034999	12108516
2970	CP026244.1	13238837	13327913	chr8	12032681	12034820
2971	CP026244.1	13328996	13414807	chr8	11960561	12031590
2972	CP026244.1	13415052	13421778	chr8	11924430	11959591
2973	CP026244.1	13422162	13431016	chr8	11897924	11923950
2974	CP026244.1	13431395	13436538	chr8	11858022	11897673
2975	CP026244.1	13436732	13465585	chr8	11826368	11857750
2976	CP026244.1	13466619	13477101	chr8	11819977	11826259
2977	CP026244.1	13477653	13528473	chr8	11806456	11819861
2978	CP026244.1	13528741	13538767	chr8	11788089	11806172
2979	CP026244.1	13539025	13576212	chr8	11783346	11787988
2980	CP026244.1	13576639	13585209	chr8	11779757	11782964
2981	CP026244.1	13585397	13612202	chr8	11768030	11779279
2982	CP026244.1	13612483	13643479	chr8	11718969	11767907
2983	CP026244.1	13643787	13687076	chr8	11674475	11718701

2984	CP026244.1	13688347	13699020	chr8	11649836	11674387
2985	CP026244.1	13699308	13730230	chr8	11642671	11649638
2986	CP026244.1	13730350	13753723	chr8	11498509	11642574
2987	CP026244.1	13753937	13880995	chr8	11462634	11498381
2988	CP026244.1	13881169	13910070	chr8	11446174	11462518
2989	CP026244.1	13910708	13960668	chr8	11443656	11446021
2990	CP026244.1	13960969	14031986	chr8	11421778	11443072
2991	CP026244.1	14032106	14035344	chr8	11204917	11421254
2992	CP026244.1	14035497	14259999	chr8	11165918	11204775
2993	CP026244.1	14260205	14294078	chr8	11101110	11164149
2994	CP026244.1	14294188	14296970	chr8	11059073	11101002
2995	CP026244.1	14298029	14387052	chr8	11057147	11058353
2996	CP026244.1	14387210	14454463	chr8	11041082	11056877
2997	CP026244.1	14454762	14492332	chr8	11025173	11040170
2998	CP026244.1	14492578	14518235	chr8	10783642	11025037
2999	CP026244.1	14518779	14597261	chr8	10637375	10783556
3000	CP026244.1	14597482	14734875	chr8	10633921	10637014
3001	CP026244.1	14734998	14791427	chr8	10556767	10633732
3002	CP026244.1	14791702	14828034	chr8	10551440	10556666
3003	CP026244.1	14828143	14833711	chr8	10523186	10548929
3004	CP026244.1	14834640	14915654	chr8	10484910	10395463
3005	CP026244.1	14915850	14983321	chr8	10395108	10206199
3006	CP026244.1	14983653	15016110	chr8	10206058	10203012
3007	CP026244.1	15016892	15036800	chr8	10199984	10179488
3008	CP026244.1	15036983	15047777	chr8	10178251	10144181
3009	CP026244.1	15048880	15050151	chr8	10144016	10067887
3010	CP026244.1	15050575	15052702	chr8	10065756	10035080
3011	CP026244.1	15052808	15073908	chr8	10034898	10007880
3012	CP026244.1	15075056	15082650	chr8	10004673	9932266

3013	CP026244.1	15082838	15146712	chr8	9889164	9932165
3014	CP026244.1	15146937	15200541	chr8	9792189	9888789
3015	CP026244.1	15200803	15282364	chr8	9744413	9791998
3016	CP026244.1	15282638	15352543	chr8	9361586	9744321
3017	CP026244.1	15353815	15362406	chr8	9294697	9360900
3018	CP026244.1	15362554	15436478	chr8	9234496	9294522
3019	CP026244.1	15436579	15443519	chr8	9140364	9234243
3020	CP026244.1	15443650	15770732	chr8	9073908	9140264
3021	CP026244.1	15771218	15821046	chr8	8904824	9073800
3022	CP026244.1	15821179	15822963	chr8	8704092	8904636
3023	CP026244.1	15823272	15873990	chr8	8521119	8703326
3024	CP026244.1	15874291	15889047	chr8	8434037	8521008
3025	CP026244.1	15889333	15968533	chr8	8281630	8433734
3026	CP026244.1	15968841	16088344	chr8	8277915	8281529
3027	CP026244.1	16088647	16166052	chr8	8272542	8277571
3028	CP026244.1	16166173	16177074	chr8	8252873	8272063
3029	CP026244.1	16177176	16229106	chr8	8245110	8247312
3030	CP026244.1	16229207	16244003	chr8	8237699	8243561
3031	CP026244.1	16244111	16252199	chr8	8231386	8237196
3032	CP026244.1	16253903	16319772	chr8	8229827	8230870
3033	CP026244.1	16319881	16459264	chr8	8226931	8229694
3034	CP026244.1	16459442	16515307	chr8	8208027	8226279
3035	CP026244.1	16515479	16620058	chr8	8202014	8207429
3036	CP026244.1	16620165	16680828	chr8	8195516	8200624
3037	CP026244.1	16680931	16702938	chr8	8188838	8193868
3038	CP026244.1	16703029	16849426	chr8	8167689	8188364
3039	CP026244.1	16849689	16871101	chr8	8162677	8167372
3040	CP026244.1	16871447	16896070	chr8	8160369	8162282
3041	CP026244.1	16896508	16898457	chr8	8155855	8158746

3042	CP026244.1	16898661	16965442	chr8	8153182	8155023
3043	CP026244.1	16965551	16969595	chr8	8142128	8150717
3044	CP026244.1	16969701	17022712	chr8	8131767	8140286
3045	CP026244.1	17022903	17041200	chr8	8108094	8131290
3046	CP026244.1	17041353	17124578	chr8	8103623	8106072
3047	CP026244.1	17124818	17128184	chr8	8097722	8102992
3048	CP026244.1	17128443	17152938	chr8	8080404	8096493
3049	CP026244.1	17153041	17157120	chr8	8076480	8077763
3050	CP026244.1	17157749	17160278	chr8	8064877	8074189
3051	CP026244.1	17160652	17184916	chr8	8041247	8063849
3052	CP026244.1	17185218	17228916	chr8	8038376	8040885
3053	CP026244.1	17229561	17240749	chr8	8032241	8038244
3054	CP026244.1	17240863	17254558	chr8	8024214	8031671
3055	CP026244.1	17255505	17372595	chr8	8016206	8023570
3056	CP026244.1	17373192	17377737	chr8	8014015	8015433
3057	CP026244.1	17377823	17393763	chr8	8011554	8013475
3058	CP026244.1	17393867	17442133	chr8	7979917	8008563
3059	CP026244.1	17442306	17544391	chr8	7973828	7978514
3060	CP026244.1	17544814	17589457	chr8	7967312	7973657
3061	CP026244.1	17589561	17601577	chr8	7951622	7966784
3062	CP026244.1	17601758	17604281	chr8	7918681	7950555
3063	CP026244.1	17604574	17691048	chr8	7913864	7918331
3064	CP026244.1	17691410	17917700	chr8	7911523	7912873
3065	CP026244.1	17917840	17931062	chr8	7903650	7907748
3066	CP026244.1	17931300	17945909	chr8	7900636	7902571
3067	CP026244.1	17946244	18287257	chr8	7889625	7900287
3068	CP026244.1	18287645	18306446	chr8	7858614	7888507
3069	CP026244.1	18306788	18331808	chr8	7830432	7857355
3070	CP026244.1	18332635	18370140	chr8	7820736	7828232

3071	CP026244.1	18370343	18401209	chr8	7817463	7819543
3072	CP026244.1	18401322	18404697	chr8	7814647	7816203
3073	CP026244.1	18406640	18522463	chr8	7804482	7814092
3074	CP026244.1	18522893	18622817	chr8	7801816	7803829
3075	CP026244.1	18623162	18751118	chr8	7797786	7800921
3076	CP026244.1	18751352	18765664	chr8	7725035	7797420
3077	CP026244.1	18765760	18793039	chr8	7677085	7724930
3078	CP026244.1	18793193	18845743	chr8	7654423	7676425
3079	CP026244.1	18846066	18850995	chr8	7585734	7653829
3080	CP026244.1	18851236	18886449	chr8	7545185	7584359
3081	CP026244.1	18886572	18905950	chr8	7534822	7544990
3082	CP026244.1	18906109	18913108	chr8	7530703	7532171
3083	CP026244.1	18913300	19055475	chr8	7524881	7527417
3084	CP026244.1	19055582	19221147	chr8	7521703	7524360
3085	CP026244.1	19221292	19319483	chr8	7517750	7521430
3086	CP026244.1	19319846	19324904	chr8	7491950	7517254
3087	CP026244.1	19325172	19452928	chr8	7482534	7491619
3088	CP026244.1	19453029	19457972	chr8	7473663	7482256
3089	CP026244.1	19458185	19557450	chr8	7448596	7473437
3090	CP026244.1	19557815	19572820	chr8	7446738	7448431
3091	CP026244.1	19572907	19578872	chr8	7421071	7445945
3092	CP026244.1	19580157	19581346	chr8	7416594	7420796
3093	CP026244.1	19581440	19616688	chr8	7403058	7401774
3094	CP026244.1	19616857	19634524	chr8	7395587	7416302
3095	CP026244.1	19634715	19696937	chr8	7369117	7394769
3096	CP026244.1	19697233	19728905	chr8	7354817	7368911
3097	CP026244.1	19729225	19860908	chr8	7331718	7354338
3098	CP026244.1	19871956	20016743	chr8	7325052	7329506
3099	CP026244.1	20017128	20083746	chr8	7290453	7321508

3100	CP026244.1	20085118	20094407	chr8	7281968	7289527
3101	CP026244.1	20094773	20096986	chr8	7277928	7281478
3102	CP026244.1	20097205	20117986	chr8	7272133	7274392
3103	CP026244.1	20119017	20122757	chr8	7269391	7271839
3104	CP026244.1	20122863	20136983	chr8	7263693	7268719
3105	CP026244.1	20137249	20138372	chr8	7258201	7260175
3106	CP026244.1	20138729	20140437	chr8	7244113	7255858
3107	CP026244.1	20140564	20174217	chr8	7244012	7237658
3108	CP026244.1	20174483	20190187	chr8	7237229	7233496
3109	CP026244.1	20191406	20227836	chr8	7233149	7231046
3110	CP026244.1	20227928	20263319	chr8	7228228	7203662
3111	CP026244.1	20249300	20250563	chr8	7202476	7186400
3112	CP026244.1	20263857	20269159	chr8	7183943	7178057
3113	CP026244.1	20269301	20302208	chr8	7177953	7171161
3114	CP026244.1	20302440	20313637	chr8	7171047	7145278
3115	CP026244.1	20313815	20318206	chr8	7144294	7140223
3116	CP026244.1	20318554	20333382	chr8	7139930	7138502
3117	CP026244.1	20333613	20372743	chr8	7137778	7127198
3118	CP026244.1	20372929	20376985	chr8	7125818	7101999
3119	CP026244.1	20377229	20381207	chr8	7101702	7098887
3120	CP026244.1	20381314	20414323	chr8	7097090	7095500
3121	CP026244.1	20415106	20416405	chr8	7095388	7082393
3122	CP026244.1	20418110	20424013	chr8	7082134	7049283
3123	CP026244.1	20424138	20436261	chr8	7048957	7024697
3124	CP026244.1	20436477	20475063	chr8	7018412	6941524
3125	CP026244.1	20475407	20499494	chr8	6941111	6934875
3126	CP026244.1	20499615	20501417	chr8	6933656	6919098
3127	CP026244.1	20501918	20521845	chr8	6918435	6913283
3128	CP026244.1	20523729	20535092	chr8	6913162	6906129

3129	CP026244.1	20535240	20543660	chr8	6905619	6884357
3130	CP026244.1	20543904	20575110	chr8	6883875	6880760
3131	CP026244.1	20577506	20581846	chr8	6880486	6877100
3132	CP026244.1	20584476	20612635	chr8	6876568	6869376
3133	CP026244.1	20615775	20617488	chr8	6869109	6864125
3134	CP026244.1	20621188	20633462	chr8	6863797	6861331
3135	CP026244.1	20633751	20638008	chr8	6861125	6859949
3136	CP026244.1	20638643	20644900	chr8	6859854	6857911
3137	CP026244.1	20645228	20652153	chr8	6857585	6824319
3138	CP026244.1	20652318	20654600	chr8	6824208	6790110
3139	CP026244.1	20654721	20656887	chr8	6786595	6724355
3140	CP026244.1	20657696	20672889	chr8	6723414	6685431
3141	CP026244.1	20673076	20675575	chr8	6683407	6605501
3142	CP026244.1	20675805	20684195	chr8	6604612	6601003
3143	CP026244.1	20685189	20688796	chr8	6600555	6597377
3144	CP026244.1	20689072	20693404	chr8	6596824	6589525
3145	CP026244.1	20694150	20697393	chr8	6589154	6587787
3146	CP026244.1	20698646	20700578	chr8	6587272	6583593
3147	CP026244.1	20703729	20708151	chr8	6583454	6581515
3148	CP026244.1	20708273	20716397	chr8	6581357	6574156
3149	CP026244.1	20717660	20730093	chr8	6573914	6570479
3150	CP026244.1	20731019	20741562	chr8	6569096	6565593
3151	CP026244.1	20741700	20745363	chr8	6564385	6558777
3152	CP026244.1	20746731	20748109	chr8	6558525	6550155
3153	CP026244.1	20748385	20768562	chr8	6550048	6514234
3154	CP026244.1	20768667	20771328	chr8	6514128	6504946
3155	CP026244.1	20771897	20782264	chr8	6503273	6494504
3156	CP026244.1	20782366	20793392	chr8	6493746	6451146
3157	CP026244.1	20794573	20818951	chr8	6450720	6431045

3158	CP026244.1	20819037	20821680	chr8	6430719	6403742
3159	CP026244.1	20821852	20823174	chr8	6403544	6396985
3160	CP026244.1	20824157	20882565	chr8	6395434	6390497
3161	CP026244.1	20882689	20889603	chr8	6389142	6374940
3162	CP026244.1	20893325	20894900	chr8	6373561	6371023
3163	CP026244.1	20900255	20906871	chr8	6370909	6369119
3164	CP026244.1	20907477	20911319	chr8	6368357	6358642
3165	CP026244.1	20911600	20915048	chr8	6358510	6356703
3166	CP026244.1	20915178	20922872	chr8	6356573	6327370
3167	CP026244.1	20926632	20929010	chr8	6327154	6299018
3168	CP026244.1	20931958	21074323	chr8	6298809	6290623
3169	CP026244.1	21074447	21086716	chr8	6290524	6268116
3170	CP026244.1	21086957	21130664	chr8	6267575	6242709
3171	CP026244.1	21132645	21178824	chr8	6242615	6235021
3172	CP026244.1	21179204	21183384	chr8	6234071	6211698
3173	CP026244.1	21183497	21186307	chr8	6211561	6192153
3174	CP026244.1	21186432	21207764	chr8	6191950	6169005
3175	CP026244.1	21207999	21225933	chr8	6168807	6154494
3176	CP026244.1	21226866	21235898	chr8	6153100	6117619
3177	CP026244.1	21236707	21239141	chr8	6117327	6092460
3178	CP026244.1	21239911	21289950	chr8	6092303	6076018
3179	CP026244.1	21290461	21351319	chr8	6074481	6050152
3180	CP026244.1	21352939	21372017	chr8	6050046	6031040
3181	CP026244.1	21372235	21397000	chr8	6027432	6021471
3182	CP026244.1	21397464	21407515	chr8	6021302	5986311
3183	CP026244.1	21407611	21412002	chr8	5984773	5982979
3184	CP026244.1	21412153	21416335	chr8	5981550	5972254
3185	CP026244.1	21416846	21418971	chr8	5971789	5956129
3186	CP026244.1	21419698	21425843	chr8	5956039	5947622

3187	CP026244.1	21426076	21446474	chr8	5947245	5943975
3188	CP026244.1	21446585	21471742	chr8	5943670	5941964
3189	CP026244.1	21471850	21500553	chr8	5941847	5937967
3190	CP026244.1	21500750	21547785	chr8	5937627	5934194
3191	CP026244.1	21547891	21606296	chr8	5933618	5927384
3192	CP026244.1	21606406	21625718	chr8	5927279	5911194
3193	CP026244.1	21625964	21639587	chr8	5911093	5892626
3194	CP026244.1	21642687	21644099	chr8	5892437	5869588
3195	CP026244.1	21645802	21647047	chr8	5868618	5849603
3196	CP026244.1	21647405	21661024	chr8	5849353	5830540
3197	CP026244.1	21661538	21664151	chr8	5826801	5809694
3198	CP026244.1	21664267	21671537	chr8	5809378	5804098
3199	CP026244.1	21671760	21676721	chr8	5802763	5797066
3200	CP026244.1	21676810	21683344	chr8	5795936	5792608
3201	CP026244.1	21683734	21690874	chr8	5791417	5779681
3202	CP026244.1	21691073	21741613	chr8	5778724	5772232
3203	CP026244.1	21741921	21757729	chr8	5770418	5753987
3204	CP026244.1	21757884	21784461	chr8	5753574	5750822
3205	CP026244.1	21785456	21826124	chr8	5750105	5743059
3206	CP026244.1	21826392	21837479	chr8	5742840	5740892
3207	CP026244.1	21838010	21842377	chr8	5739489	5737929
3208	CP026244.1	21842553	21850642	chr8	5735991	5734355
3209	CP026244.1	21850764	21862164	chr8	5732995	5725282
3210	CP026244.1	21862571	21864301	chr8	5723616	5721203
3211	CP026244.1	21864509	21866978	chr8	5720154	5718209
3212	CP026244.1	21867284	21907334	chr8	5716934	5715435
3213	CP026244.1	21907601	21909604	chr8	5714364	5710128
3214	CP026244.1	21910599	21912676	chr8	5707121	5702375
3215	CP026244.1	21912959	21937558	chr8	5701452	5699890

3216	CP026244.1	21937805	21944541	chr8	5699370	5684631
3217	CP026244.1	21945053	21962610	chr8	5683609	5679396
3218	CP026244.1	21963953	21967227	chr8	5675292	5673072
3219	CP026244.1	22113108	22156290	chr8	5668467	5661799
3220	CP026244.1	22157057	22183682	chr8	5658264	5655465
3221	CP026244.1	22184480	22185783	chr8	5654945	5643081
3222	CP026244.1	22186109	22190333	chr8	5642815	5635887
3223	CP026244.1	22190910	22201578	chr8	5634652	5629113
3224	CP026244.1	22201756	22208206	chr8	5628839	5622767
3225	CP026244.1	22208907	22219703	chr8	5618038	5609382
3226	CP026244.1	22221050	22224897	chr8	5608804	5607535
3227	CP026244.1	22225474	22226612	chr8	5606744	5602834
3228	CP026244.1	22227152	22229074	chr8	5602647	5601346
3229	CP026244.1	22236834	22242168	chr8	5599318	5597475
3230	CP026244.1	22242324	22254413	chr8	5584700	5597374
3231	CP026244.1	22254724	22258536	chr8	5562830	5578252
3232	CP026244.1	22258930	22389551	chr8	5553479	5562637
3233	CP026244.1	22390117	22426961	chr8	5544993	5552911
3234	CP026244.1	22427376	22431613	chr8	5538784	5544643
3235	CP026244.1	22432946	22445501	chr8	5523176	5538683
3236	CP026244.1	22445655	22454423	chr8	5521050	5523017
3237	CP026244.1	22454685	22531586	chr8	5488123	5520749
3238	CP026244.1	22531864	22534704	chr8	5471348	5487711
3239	CP026244.1	22535314	22551401	chr8	5425485	5471236
3240	CP026244.1	22551505	22556486	chr8	5401331	5424964
3241	CP026244.1	22556630	22568512	chr8	5399062	5401230
3242	CP026244.1	22568648	22569760	chr8	5205435	5398293
3243	CP026244.1	22570158	22574348	chr8	5159340	5204035
3244	CP026244.1	22575408	22578013	chr8	5152477	5159083

3245	CP026244.1	22579038	22589981	chr8	5110477	5152374
3246	CP026244.1	22590312	22593097	chr8	5021532	5110107
3247	CP026244.1	22593362	22604373	chr8	5015072	5021324
3248	CP026244.1	22605501	22614256	chr8	4868826	5014862
3249	CP026244.1	22614470	22627496	chr8	4843397	4868392
3250	CP026244.1	22627797	22656825	chr8	4830324	4843262
3251	CP026244.1	22657362	22664371	chr8	4824314	4829315
3252	CP026244.1	22665856	22671080	chr8	4807291	4823748
3253	CP026244.1	22671287	22672897	chr8	4804064	4806288
3254	CP026244.1	22673232	22679776	chr8	4799510	4803808
3255	CP026244.1	22680354	22700321	chr8	4759706	4799353
3256	CP026244.1	22701173	22738224	chr8	4751937	4759613
3257	CP026244.1	22739182	22741310	chr8	4743784	4750941
3258	CP026244.1	22741450	22806051	chr8	4702814	4740067
3259	CP026244.1	22806183	22809793	chr8	4675012	4702501
3260	CP026244.1	22809934	22824366	chr8	4666312	4674839
3261	CP026244.1	22825270	22829300	chr8	4629052	4665805
3262	CP026244.1	22829395	22831589	chr8	4580640	4628912
3263	CP026244.1	22831983	22839240	chr8	4575741	4577910
3264	CP026244.1	22840449	22848857	chr8	4558531	4575240
3265	CP026244.1	22849043	22852657	chr8	4540703	4558297
3266	CP026244.1	22852798	22856474	chr8	4527327	4540601
3267	CP026244.1	22856774	22871868	chr8	4512259	4527030
3268	CP026244.1	22872840	22876474	chr8	4480433	4510489
3269	CP026244.1	22876739	22882308	chr8	4477516	4480233
3270	CP026244.1	22882429	22885095	chr8	4468631	4477347
3271	CP026244.1	22885220	22894312	chr8	4458875	4468273
3272	CP026244.1	22894575	22898182	chr8	4424515	4457083
3273	CP026244.1	22898383	22902667	chr8	4420735	4422692

3274	CP026244.1	22904904	22907827	chr8	4413937	4420512
3275	CP026244.1	22908212	22911501	chr8	4398145	4413691
3276	CP026244.1	22912596	22913687	chr8	4385544	4398015
3277	CP026244.1	22913791	22921512	chr8	4346623	4384990
3278	CP026244.1	22921700	22943954	chr8	4337100	4346433
3279	CP026244.1	22944055	22957435	chr8	4223590	4336933
3280	CP026244.1	22957685	22961070	chr8	4136964	4223451
3281	CP026244.1	22961721	22977180	chr8	4015633	4136788
3282	CP026244.1	22977407	22979589	chr8	4011426	4015480
3283	CP026244.1	22980739	22982285	chr8	3942542	4011340
3284	CP026244.1	22985177	23005764	chr8	3933044	3941897
3285	CP026244.1	23007800	23011399	chr8	3896697	3932953
3286	CP026244.1	23012396	23021250	chr8	3780319	3896610
3287	CP026244.1	23022077	23024381	chr8	3659918	3779978
3288	CP026244.1	23025415	23034632	chr8	3648346	3659817
3289	CP026244.1	23035166	23037586	chr8	3612134	3647776
3290	CP026244.1	23038634	23043350	chr8	3609095	3612034
3291	CP026244.1	23043563	23061261	chr8	3580373	3608807
3292	CP026244.1	23062926	23076925	chr8	3576888	3579959
3293	CP026244.1	23077474	23080538	chr8	3564343	3575702
3294	CP026244.1	23080753	23083209	chr8	3559362	3564169
3295	CP026244.1	23083713	23100022	chr8	3556231	3557875
3296	CP026244.1	23100598	23103826	chr8	3549142	3552565
3297	CP026244.1	23104706	23127958	chr8	3544694	3545978
3298	CP026244.1	23128050	23129245	chr8	3536807	3544498
3299	CP026244.1	23130588	23149191	chr8	3531556	3536214
3300	CP026244.1	23150565	23154209	chr8	3526584	3529403
3301	CP026244.1	23154366	23159604	chr8	3513902	3526438
3302	CP026244.1	23160026	23161192	chr8	3462185	3513576

3303	CP026244.1	23162090	23166348	chr8	3457259	3461142
3304	CP026244.1	23166481	23191377	chr8	3449650	3456301
3305	CP026244.1	23191873	23220120	chr8	3447752	3449313
3306	CP026244.1	23221257	23230088	chr8	3441278	3445913
3307	CP026244.1	23231094	23253703	chr8	3438225	3440000
3308	CP026244.1	23254256	23255728	chr8	3432235	3436758
3309	CP026244.1	23256109	23259088	chr8	3420946	3431987
3310	CP026244.1	23259638	23261696	chr8	3406242	3420561
3311	CP026244.1	23262752	23275824	chr8	3382781	3404871
3312	CP026244.1	23276488	23285205	chr8	3324307	3382636
3313	CP026244.1	23285730	23309834	chr8	3319562	3324206
3314	CP026244.1	23310546	23312958	chr8	3246130	3319167
3315	CP026244.1	23314117	23317875	chr8	3233035	3245915
3316	CP026244.1	23318061	23322667	chr8	3216784	3232934
3317	CP026244.1	23324891	23332162	chr8	3208005	3215170
3318	CP026244.1	23332563	23346731	chr8	3206633	3207773
3319	CP026244.1	23347418	23357869	chr8	3192729	3206108
3320	CP026244.1	23358405	23361652	chr8	3167172	3192601
3321	CP026244.1	23362262	23399348	chr8	3152737	3166893
3322	CP026244.1	23399485	23429451	chr8	3147902	3150490
3323	CP026244.1	23431129	23445589	chr8	3100081	3144371
3324	CP026244.1	23446109	23448314	chr8	3032685	3098202
3325	CP026244.1	23449040	23468373	chr8	2972109	3032558
3326	CP026244.1	23468648	23474877	chr8	2961037	2971933
3327	CP026244.1	23477184	23487264	chr8	2956886	2960804
3328	CP026244.1	23487365	23511256	chr8	2945324	2956668
3329	CP026244.1	23511615	23516344	chr8	2858921	2944880
3330	CP026244.1	23516445	23519684	chr8	2762941	2858621
3331	CP026244.1	23521946	23537086	chr8	2759004	2762825

3332	CP026244.1	23537758	23563848	chr8	2751680	2448002
3333	CP026244.1	23565011	23584212	chr8	2447894	1883346
3334	CP026244.1	23584383	23586623	chr8	1883217	1814142
3335	CP026244.1	23587053	23589897	chr8	1814022	1608658
3336	CP026244.1	23590337	23598270	chr8	1607488	1396522
3337	CP026244.1	23599268	23611220	chr8	1392600	1217376
3338	CP026244.1	23611565	23624728	chr8	1216757	1204556
3339	CP026244.1	23625307	23630613	chr8	1203666	1201470
3340	CP026244.1	23631330	23651854	chr8	1201334	1198378
3341	CP026244.1	23652158	23656384	chr8	1198249	1128071
3342	CP026244.1	23657247	23680884	chr8	1127936	1122535
3343	CP026244.1	23699049	23705585	chr8	1122298	1088470
3344	CP026244.1	23707321	23711444	chr8	1001471	1088369
3345	CP026244.1	23711569	23717319	chr8	868697	1001193
3346	CP026244.1	23717726	23722037	chr8	867444	868577
3347	CP026244.1	23722405	23724592	chr8	841755	867214
3348	CP026244.1	23724861	23726336	chr8	699846	841669
3349	CP026244.1	23726436	23732396	chr8	688238	699686
3350	CP026244.1	23733043	23756789	chr8	661198	688043
3351	CP026244.1	23757189	23778561	chr8	608724	661058
3352	CP026244.1	23778913	23790189	chr8	592971	608590
3353	CP026244.1	23790409	23817393	chr8	545520	592651
3354	CP026244.1	23818046	23826672	chr8	390503	544664
3355	CP026244.1	23828232	23836697	chr8	1	390397
3356	CP026261.1	14498873	14760684	chr9	5915359	5653548
3357	CP026261.1	14481757	14498508	chr9	5932475	5915724
3358	CP026261.1	14478544	14481518	chr9	5935688	5932714
3359	CP026261.1	14418259	14478008	chr9	5995973	5936224
3360	CP026261.1	14414815	14418018	chr9	5999417	5996214

3361	CP026261.1	14395628	14414635	chr9	6018604	5999597
3362	CP026261.1	14282587	14395451	chr9	6131645	6018781
3363	CP026261.1	14200344	14282489	chr9	6213888	6131743
3364	CP026261.1	14190641	14196868	chr9	6223591	6217364
3365	CP026261.1	14156547	14188865	chr9	6257685	6225367
3366	CP026261.1	14130687	14156393	chr9	6283545	6257839
3367	CP026261.1	14086685	14130359	chr9	6327547	6283873
3368	CP026261.1	14080166	14086587	chr9	6334066	6327645
3369	CP026261.1	14068844	14079897	chr9	6345388	6334335
3370	CP026261.1	14057703	14067435	chr9	6356529	6346797
3371	CP026261.1	13989135	14057366	chr9	6425097	6356866
3372	CP026261.1	13982440	13989028	chr9	6431792	6425204
3373	CP026261.1	13900641	13982228	chr9	6513591	6432004
3374	CP026261.1	13865408	13900276	chr9	6548824	6513956
3375	CP026261.1	13832532	13865318	chr9	6581700	6548914
3376	CP026261.1	13685167	13832432	chr9	6729065	6581800
3377	CP026261.1	13517393	13683017	chr9	6896839	6731215
3378	CP026261.1	13371530	13517306	chr9	7042702	6896926
3379	CP026261.1	13300842	13371431	chr9	7113390	7042801
3380	CP026261.1	13249524	13300632	chr9	7164708	7113600
3381	CP026261.1	13208137	13249388	chr9	7206095	7164844
3382	CP026261.1	13195358	13207852	chr9	7218874	7206380
3383	CP026261.1	13189909	13195234	chr9	7224323	7218998
3384	CP026261.1	13159865	13189793	chr9	7254367	7224439
3385	CP026261.1	12872145	13158191	chr9	7542087	7256041
3386	CP026261.1	12825192	12872030	chr9	7589040	7542202
3387	CP026261.1	12654228	12824986	chr9	7760004	7589246
3388	CP026261.1	12621819	12654127	chr9	7792413	7760105
3389	CP026261.1	12616410	12621539	chr9	7797822	7792693

3390	CP026261.1	12578068	12616198	chr9	7836164	7798034
3391	CP026261.1	12442098	12577935	chr9	7972134	7836297
3392	CP026261.1	12437021	12441976	chr9	7977211	7972256
3393	CP026261.1	12433911	12436913	chr9	7980321	7977319
3394	CP026261.1	12232534	12433418	chr9	8181698	7980814
3395	CP026261.1	12195438	12232446	chr9	8218794	8181786
3396	CP026261.1	12152732	12195275	chr9	8261500	8218957
3397	CP026261.1	12135765	12152426	chr9	8278467	8261806
3398	CP026261.1	12127585	12135599	chr9	8286647	8278633
3399	CP026261.1	12119210	12127344	chr9	8295022	8286888
3400	CP026261.1	11946306	12119012	chr9	8467926	8295220
3401	CP026261.1	11887176	11946040	chr9	8527056	8468192
3402	CP026261.1	11883136	11887008	chr9	8531096	8527224
3403	CP026261.1	11875261	11882863	chr9	8538971	8531369
3404	CP026261.1	11757208	11875126	chr9	8657024	8539106
3405	CP026261.1	11577210	11757103	chr9	8837022	8657129
3406	CP026261.1	11467162	11577087	chr9	8947070	8837145
3407	CP026261.1	11448387	11466981	chr9	8965845	8947251
3408	CP026261.1	11341128	11447984	chr9	9073104	8966248
3409	CP026261.1	11328994	11340992	chr9	9085238	9073240
3410	CP026261.1	11324777	11328728	chr9	9089455	9085504
3411	CP026261.1	11243832	11324663	chr9	9170400	9089569
3412	CP026261.1	11093560	11243741	chr9	9320672	9170491
3413	CP026261.1	11061688	11093010	chr9	9352544	9321222
3414	CP026261.1	11045914	11061583	chr9	9368318	9352649
3415	CP026261.1	10944666	11045784	chr9	9469566	9368448
3416	CP026261.1	10927029	10944468	chr9	9487203	9469764
3417	CP026261.1	10897916	10926808	chr9	9516316	9487424
3418	CP026261.1	10872381	10897409	chr9	9541851	9516823

3419	CP026261.1	10856597	10872050	chr9	9557635	9542182
3420	CP026261.1	10784349	10856294	chr9	9629883	9557938
3421	CP026261.1	10782179	10784242	chr9	9632053	9629990
3422	CP026261.1	10761368	10782092	chr9	9652864	9632140
3423	CP026261.1	10704745	10761194	chr9	9709487	9653038
3424	CP026261.1	10581085	10704176	chr9	9833147	9710056
3425	CP026261.1	10555242	10580615	chr9	9858990	9833617
3426	CP026261.1	10397048	10555151	chr9	10017184	9859081
3427	CP026261.1	10386806	10393811	chr9	10027426	10020421
3428	CP026261.1	10337567	10381361	chr9	10076665	10032871
3429	CP026261.1	10335819	10337218	chr9	10078413	10077014
3430	CP026261.1	10319705	10335649	chr9	10094527	10078583
3431	CP026261.1	10302282	10319585	chr9	10111950	10094647
3432	CP026261.1	10288534	10300961	chr9	10125698	10113271
3433	CP026261.1	10209228	10287867	chr9	10205004	10126365
3434	CP026261.1	9763450	10209078	chr9	10650782	10205154
3435	CP026261.1	9748246	9763320	chr9	10665986	10650912
3436	CP026261.1	9743125	9748098	chr9	10671107	10666134
3437	CP026261.1	9663128	9742900	chr9	10751104	10671332
3438	CP026261.1	9538412	9662042	chr9	10875820	10752190
3439	CP026261.1	9304287	9538011	chr9	11109945	10876221
3440	CP026261.1	9267482	9304176	chr9	11146750	11110056
3441	CP026261.1	9239984	9267323	chr9	11174248	11146909
3442	CP026261.1	9216393	9238612	chr9	11197839	11175620
3443	CP026261.1	9093034	9216164	chr9	11321198	11198068
3444	CP026261.1	9002575	9092540	chr9	11411657	11321692
3445	CP026261.1	8980670	9001949	chr9	11433562	11412283
3446	CP026261.1	8976074	8980432	chr9	11438158	11433800
3447	CP026261.1	8957393	8975569	chr9	11456839	11438663

3448	CP026261.1	8926299	8957050	chr9	11487933	11457182
3449	CP026261.1	8921955	8925817	chr9	11492277	11488415
3450	CP026261.1	8921044	8921834	chr9	11493188	11492398
3451	CP026261.1	8916185	8918536	chr9	11498047	11495696
3452	CP026261.1	8718918	8914763	chr9	11695314	11499469
3453	CP026261.1	8668127	8718685	chr9	11746105	11695547
3454	CP026261.1	8643753	8667552	chr9	11770479	11746680
3455	CP026261.1	8403894	8643623	chr9	12010338	11770609
3456	CP026261.1	8370268	8403697	chr9	12043964	12010535
3457	CP026261.1	8362789	8370082	chr9	12051443	12044150
3458	CP026261.1	8313184	8362693	chr9	12101048	12051539
3459	CP026261.1	8229574	8313025	chr9	12184658	12101207
3460	CP026261.1	8125347	8229480	chr9	12288885	12184752
3461	CP026261.1	8088999	8125063	chr9	12325233	12289169
3462	CP026261.1	8050801	8087918	chr9	12363431	12326314
3463	CP026261.1	8039696	8050675	chr9	12374536	12363557
3464	CP026261.1	7962906	8039314	chr9	12451326	12374918
3465	CP026261.1	7927997	7962792	chr9	12486235	12451440
3466	CP026261.1	7918592	7927719	chr9	12495640	12486513
3467	CP026261.1	7874230	7918406	chr9	12540002	12495826
3468	CP026261.1	7870907	7874004	chr9	12543325	12540228
3469	CP026261.1	7817972	7870633	chr9	12596260	12543599
3470	CP026261.1	7791676	7817811	chr9	12622556	12596421
3471	CP026261.1	7721588	7790737	chr9	12692644	12623495
3472	CP026261.1	7657776	7721468	chr9	12756456	12692764
3473	CP026261.1	7616353	7657630	chr9	12797879	12756602
3474	CP026261.1	7600039	7616180	chr9	12814193	12798052
3475	CP026261.1	7587786	7599679	chr9	12826446	12814553
3476	CP026261.1	7582001	7587565	chr9	12832231	12826667

3477	CP026248.1	24215519	24215908	chr10	3081384	3080995
3478	CP026248.1	22251510	22448559	chr10	5093726	4896677
3479	CP026248.1	22174036	22251376	chr10	5171200	5093860
3480	CP026248.1	22147552	22173815	chr10	5197684	5171421
3481	CP026248.1	22118538	22147426	chr10	5226698	5197810
3482	CP026248.1	21932931	22118361	chr10	5412305	5226875
3483	CP026248.1	21881961	21932549	chr10	5463275	5412687
3484	CP026248.1	21876991	21881693	chr10	5468245	5463543
3485	CP026248.1	21862527	21876897	chr10	5482709	5468339
3486	CP026248.1	21860304	21862434	chr10	5484932	5482802
3487	CP026248.1	21793715	21860143	chr10	5551521	5485093
3488	CP026248.1	21730534	21793601	chr10	5614702	5551635
3489	CP026248.1	21724079	21729538	chr10	5621157	5615698
3490	CP026248.1	21707865	21723844	chr10	5637371	5621392
3491	CP026248.1	21665099	21707509	chr10	5680137	5637727
3492	CP026248.1	21661363	21664773	chr10	5683873	5680463
3493	CP026248.1	21648610	21660264	chr10	5696626	5684972
3494	CP026248.1	21645087	21648505	chr10	5700149	5696731
3495	CP026248.1	21618618	21644856	chr10	5726618	5700380
3496	CP026248.1	21599907	21618460	chr10	5745329	5726776
3497	CP026248.1	21403183	21599101	chr10	5942053	5746135
3498	CP026248.1	21363595	21402936	chr10	5981641	5942300
3499	CP026248.1	21315584	21363420	chr10	6029652	5981816
3500	CP026248.1	21094686	21315455	chr10	6250550	6029781
3501	CP026248.1	21060120	21094445	chr10	6285116	6250791
3502	CP026248.1	21042455	21059976	chr10	6302781	6285260
3503	CP026248.1	20992740	21042134	chr10	6352496	6303102
3504	CP026248.1	20796520	20992645	chr10	6548716	6352591
3505	CP026248.1	20676115	20796207	chr10	6669121	6549029

3506	CP026248.1	20566157	20664199	chr10	6779079	6681037
3507	CP026248.1	20549535	20565859	chr10	6795701	6779377
3508	CP026248.1	20219240	20549430	chr10	7125996	6795806
3509	CP026248.1	20192066	20218609	chr10	7153170	7126627
3510	CP026248.1	20142135	20191954	chr10	7203101	7153282
3511	CP026248.1	20053093	20142038	chr10	7292143	7203198
3512	CP026248.1	20048079	20052893	chr10	7297157	7292343
3513	CP026248.1	19956291	20047892	chr10	7388945	7297344
3514	CP026248.1	19940903	19955431	chr10	7404333	7389805
3515	CP026248.1	19733429	19940801	chr10	7611807	7404435
3516	CP026248.1	19729350	19733199	chr10	7615886	7612037
3517	CP026248.1	19699915	19729165	chr10	7645321	7616071
3518	CP026248.1	19610208	19699580	chr10	7735028	7645656
3519	CP026248.1	19465952	19608742	chr10	7879284	7736494
3520	CP026248.1	19181134	19465267	chr10	8164102	7879969
3521	CP026248.1	19173291	19180565	chr10	8171945	8164671
3522	CP026248.1	19161425	19172904	chr10	8183811	8172332
3523	CP026248.1	19069299	19161303	chr10	8275937	8183933
3524	CP026248.1	19063653	19068864	chr10	8281583	8276372
3525	CP026248.1	19036210	19063238	chr10	8309026	8281998
3526	CP026248.1	18989741	19036030	chr10	8355495	8309206
3527	CP026248.1	18958761	18989334	chr10	8386475	8355902
3528	CP026248.1	18941126	18958646	chr10	8404110	8386590
3529	CP026248.1	18897211	18941013	chr10	8448025	8404223
3530	CP026248.1	18845353	18896199	chr10	8499883	8449037
3531	CP026248.1	18730974	18845251	chr10	8614262	8499985
3532	CP026248.1	18655734	18730841	chr10	8689502	8614395
3533	CP026248.1	18608616	18651201	chr10	8736620	8694035
3534	CP026248.1	18585763	18608301	chr10	8759473	8736935

3535	CP026248.1	18538970	18585636	chr10	8806266	8759600
3536	CP026248.1	18402816	18538879	chr10	8942420	8806357
3537	CP026248.1	18393285	18402657	chr10	8951951	8942579
3538	CP026248.1	18276292	18392557	chr10	9068944	8952679
3539	CP026248.1	18274137	18275860	chr10	9071099	9069376
3540	CP026248.1	18233476	18274043	chr10	9111760	9071193
3541	CP026248.1	18073032	18233170	chr10	9272204	9112066
3542	CP026248.1	17828419	18072773	chr10	9516817	9272463
3543	CP026248.1	17816440	17828270	chr10	9528796	9516966
3544	CP026248.1	17716901	17815862	chr10	9628335	9529374
3545	CP026248.1	17693136	17713343	chr10	9652100	9631893
3546	CP026248.1	17670199	17692865	chr10	9675037	9652371
3547	CP026248.1	17477192	17670087	chr10	9868044	9675149
3548	CP026248.1	17315557	17476561	chr10	10029679	9868675
3549	CP026248.1	17222478	17315265	chr10	10122758	10029971
3550	CP026248.1	17183836	17222266	chr10	10161400	10122970
3551	CP026248.1	17155249	17183702	chr10	10189987	10161534
3552	CP026248.1	16909594	17154986	chr10	10435642	10190250
3553	CP026248.1	16762678	16908207	chr10	10582558	10437029
3554	CP026248.1	16667424	16762417	chr10	10677812	10582819
3555	CP026248.1	16544134	16667182	chr10	10801102	10678054
3556	CP026248.1	16486787	16543289	chr10	10858449	10801947
3557	CP026248.1	16483546	16486693	chr10	10861690	10858543
3558	CP026248.1	16472041	16483390	chr10	10873195	10861846
3559	CP026248.1	16405564	16471921	chr10	10939672	10873315
3560	CP026248.1	16375408	16405395	chr10	10969828	10939841
3561	CP026248.1	16258852	16375211	chr10	11086384	10970025
3562	CP026248.1	16132962	16258721	chr10	11212274	11086515
3563	CP026248.1	16069525	16132834	chr10	11275711	11212402

3564	CP026248.1	15941826	16069248	chr10	11403410	11275988
3565	CP026248.1	15899081	15941705	chr10	11446155	11403531
3566	CP026248.1	15759663	15898964	chr10	11585573	11446272
3567	CP026248.1	15739323	15759177	chr10	11605913	11586059
3568	CP026248.1	15733359	15738313	chr10	11611877	11606923
3569	CP026248.1	15667277	15733190	chr10	11677959	11612046
3570	CP026248.1	15646008	15667190	chr10	11699228	11678046
3571	CP026248.1	15644480	15645904	chr10	11700756	11699332
3572	CP026248.1	15631607	15644299	chr10	11713629	11700937
3573	CP026248.1	15607287	15630092	chr10	11737949	11715144
3574	CP026248.1	15598456	15607152	chr10	11746780	11738084
3575	CP026248.1	15504986	15598359	chr10	11840250	11746877
3576	CP026248.1	14975139	15504362	chr10	12370097	11840874
3577	CP026248.1	14852497	14974355	chr10	12492739	12370881
3578	CP026248.1	14829210	14851876	chr10	12516026	12493360
3579	CP026248.1	14794828	14828163	chr10	12550408	12517073
3580	CP026248.1	14774691	14794582	chr10	12570545	12550654
3581	CP026248.1	14637394	14773933	chr10	12707842	12571303
3582	CP026248.1	14379008	14637069	chr10	12966228	12708167
3583	CP026248.1	14368599	14378871	chr10	12976637	12966365
3584	CP026248.1	14305552	14368388	chr10	13039684	12976848
3585	CP026248.1	14251601	14304808	chr10	13093635	13040428
3586	CP026248.1	14229662	14250854	chr10	13115574	13094382
3587	CP026248.1	14002310	14229464	chr10	13342926	13115772
3588	CP026248.1	13978288	14002064	chr10	13366948	13343172
3589	CP026248.1	13893031	13977563	chr10	13452205	13367673
3590	CP026248.1	13868604	13892441	chr10	13476632	13452795
3591	CP026248.1	13766932	13868503	chr10	13578304	13476733
3592	CP026248.1	13754310	13766747	chr10	13590926	13578489

3593	CP026248.1	13626080	13754172	chr10	13719156	13591064
3594	CP026248.1	13545897	13625867	chr10	13799339	13719369
3595	CP026248.1	13307970	13545414	chr10	14037266	13799822
3596	CP026248.1	13275758	13307602	chr10	14069478	14037634
3597	CP026248.1	13221973	13275665	chr10	14123263	14069571
3598	CP026248.1	13187240	13221169	chr10	14157996	14124067
3599	CP026248.1	13131754	13187021	chr10	14213482	14158215
3600	CP026248.1	13034039	13131588	chr10	14311197	14213648
3601	CP026248.1	13021443	13033914	chr10	14323793	14311322
3602	CP026248.1	12982686	13020392	chr10	14362550	14324844
3603	CP026248.1	12889014	12982169	chr10	14456222	14363067
3604	CP026248.1	12744481	12888926	chr10	14600755	14456310
3605	CP026248.1	12639610	12744339	chr10	14705626	14600897
3606	CP026248.1	12638134	12638635	chr10	14707102	14706601
3607	CP026248.1	12634865	12637989	chr10	14710371	14707247
3608	CP026248.1	12482241	12634605	chr10	14862995	14710631
3609	CP026248.1	12288906	12482142	chr10	15056330	14863094
3610	CP026248.1	12282667	12288446	chr10	15062569	15056790
3611	CP026248.1	12079929	12254936	chr10	15265307	15090300
3612	CP026248.1	12067192	12078945	chr10	15278044	15266291
3613	CP026248.1	11997917	12067106	chr10	15347319	15278130
3614	CP026248.1	11916546	11997787	chr10	15428690	15347449
3615	CP026248.1	11866672	11916413	chr10	15478564	15428823
3616	CP026248.1	11760068	11866484	chr10	15585168	15478752
3617	CP026248.1	11580170	11759680	chr10	15765066	15585556
3618	CP026248.1	11478060	11579452	chr10	15867176	15765784
3619	CP026248.1	11476136	11477665	chr10	15869100	15867571
3620	CP026248.1	11430469	11476033	chr10	15914767	15869203
3621	CP026248.1	11270433	11429216	chr10	16074803	15916020

3622	CP026248.1	11117098	11270194	chr10	16228138	16075042
3623	CP026248.1	10914377	11116988	chr10	16430859	16228248
3624	CP026248.1	10747003	10914013	chr10	16598233	16431223
3625	CP026248.1	10732708	10746895	chr10	16612528	16598341
3626	CP026248.1	10722643	10732129	chr10	16622593	16613107
3627	CP026248.1	10605441	10722537	chr10	16739795	16622699
3628	CP026248.1	10516199	10605150	chr10	16829037	16740086
3629	CP026248.1	10361451	10515923	chr10	16983785	16829313
3630	CP026248.1	10353837	10361122	chr10	16991399	16984114
3631	CP026248.1	10301864	10349211	chr10	17043372	16996025
3632	CP026248.1	10249814	10301763	chr10	17095422	17043473
3633	CP026248.1	10244307	10249354	chr10	17100929	17095882
3634	CP026248.1	10193305	10244165	chr10	17151931	17101071
3635	CP026248.1	10170197	10193087	chr10	17175039	17152149
3636	CP026248.1	10024241	10170028	chr10	17320995	17175208
3637	CP026248.1	10017576	10023891	chr10	17327660	17321345
3638	CP026248.1	9914657	10017225	chr10	17430579	17328011
3639	CP026248.1	9886683	9914439	chr10	17458553	17430797
3640	CP026248.1	9842379	9886436	chr10	17502857	17458800
3641	CP026248.1	9815349	9842231	chr10	17529887	17503005
3642	CP026248.1	9658506	9815178	chr10	17686730	17530058
3643	CP026248.1	9538592	9658378	chr10	17806644	17686858
3644	CP026248.1	9522844	9538502	chr10	17822392	17806734
3645	CP026248.1	9490444	9522738	chr10	17854792	17822498
3646	CP026248.1	9482683	9490343	chr10	17862553	17854893
3647	CP026248.1	9457033	9482273	chr10	17888203	17862963
3648	CP026248.1	9222244	9456698	chr10	18122992	17888538
3649	CP026248.1	9138767	9221380	chr10	18206469	18123856
3650	CP026248.1	9028646	9138590	chr10	18316590	18206646

3651	CP026248.1	8976769	9028377	chr10	18368467	18316859
3652	CP026248.1	8857669	8976609	chr10	18487567	18368627
3653	CP026248.1	8770302	8857518	chr10	18574934	18487718
3654	CP026248.1	8767623	8770188	chr10	18577613	18575048
3655	CP026248.1	8708526	8767494	chr10	18636710	18577742
3656	CP026248.1	8698221	8708413	chr10	18647015	18636823
3657	CP026248.1	8688736	8697497	chr10	18656500	18647739
3658	CP026248.1	8654659	8688573	chr10	18690577	18656663
3659	CP026248.1	8604360	8654543	chr10	18740876	18690693
3660	CP026248.1	8532341	8604113	chr10	18812895	18741123
3661	CP026248.1	8491685	8532007	chr10	18853551	18813229
3662	CP026248.1	8479999	8491423	chr10	18865237	18853813
3663	CP026248.1	8478945	8479729	chr10	18866291	18865507
3664	CP026248.1	8475574	8478850	chr10	18869662	18866386
3665	CP026248.1	8435988	8475417	chr10	18909248	18869819
3666	CP026248.1	8431578	8435155	chr10	18913658	18910081
3667	CP026248.1	8420911	8430230	chr10	18924325	18915006
3668	CP026248.1	8417635	8420799	chr10	18927601	18924437
3669	CP026248.1	8350005	8417360	chr10	18995231	18927876
3670	CP026248.1	8262279	8349411	chr10	19082957	18995825
3671	CP026248.1	8259286	8262160	chr10	19085950	19083076
3672	CP026248.1	8204443	8258711	chr10	19140793	19086525
3673	CP026248.1	8152742	8204353	chr10	19192494	19140883
3674	CP026248.1	8116640	8151760	chr10	19228596	19193476
3675	CP026248.1	8063514	8116550	chr10	19281722	19228686
3676	CP026248.1	8051691	8063358	chr10	19293545	19281878
3677	CP026248.1	8031556	8047865	chr10	19313680	19297371
3678	CP026248.1	8021456	8031331	chr10	19323780	19313905
3679	CP026248.1	8012938	8021336	chr10	19332298	19323900

3680	CP026248.1	7986451	8012805	chr10	19358785	19332431
3681	CP026248.1	7982901	7986354	chr10	19362335	19358882
3682	CP026248.1	7813623	7982733	chr10	19531613	19362503
3683	CP026248.1	7760700	7813513	chr10	19584536	19531723
3684	CP026248.1	7705739	7760596	chr10	19639497	19584640
3685	CP026248.1	7699343	7705437	chr10	19645893	19639799
3686	CP026248.1	7578213	7699092	chr10	19767023	19646144
3687	CP026248.1	7454326	7577871	chr10	19890910	19767365
3688	CP026248.1	7440334	7454214	chr10	19904902	19891022
3689	CP026248.1	7364220	7440087	chr10	19981016	19905149
3690	CP026248.1	7288961	7362653	chr10	20056275	19982583
3691	CP026248.1	7241012	7287818	chr10	20104224	20057418
3692	CP026248.1	7234340	7240763	chr10	20110896	20104473
3693	CP026248.1	7173607	7234231	chr10	20171629	20111005
3694	CP026248.1	7169545	7173269	chr10	20175691	20171967
3695	CP026248.1	7161396	7169362	chr10	20183840	20175874
3696	CP026248.1	7148149	7160919	chr10	20197087	20184317
3697	CP026248.1	7140398	7147969	chr10	20204838	20197267
3698	CP026248.1	7115066	7140092	chr10	20230170	20205144
3699	CP026248.1	7107204	7114218	chr10	20238032	20231018
3700	CP026248.1	7102267	7106925	chr10	20242969	20238311
3701	CP026248.1	7080747	7101625	chr10	20264489	20243611
3702	CP026248.1	7068255	7080512	chr10	20276981	20264724
3703	CP026248.1	6956084	7067991	chr10	20389152	20277245
3704	CP026248.1	6952429	6955861	chr10	20392807	20389375
3705	CP026248.1	6948525	6952079	chr10	20396711	20393157
3706	CP026248.1	6926679	6947977	chr10	20418557	20397259
3707	CP026248.1	6917279	6925796	chr10	20427957	20419440
3708	CP026248.1	6889212	6917046	chr10	20456024	20428190

3709	CP026248.1	6799021	6888978	chr10	20546215	20456258
3710	CP026248.1	6756400	6798821	chr10	20588836	20546415
3711	CP026248.1	6732475	6756299	chr10	20612761	20588937
3712	CP026248.1	6719326	6731898	chr10	20625910	20613338
3713	CP026248.1	6692943	6719033	chr10	20652293	20626203
3714	CP026248.1	6658904	6692632	chr10	20686332	20652604
3715	CP026248.1	3648798	3648935	chr10	23649559	23649423
3716	CP026248.1	3648418	3648554	chr10	23649940	23649803
3717	CP026264.1	368	1688	chr11	15227957	15207695
3718	CP026264.1	2914	4124	chr11	15191411	15038375
3719	CP026264.1	4707	20751	chr11	15038266	15036914
3720	CP026264.1	21535	23063	chr11	15036809	15034478
3721	CP026264.1	24058	39983	chr11	15033154	14926836
3722	CP026264.1	40446	64615	chr11	14926326	14924441
3723	CP026264.1	65114	93998	chr11	14920900	14917666
3724	CP026264.1	94398	96396	chr11	14915451	14868563
3725	CP026264.1	96839	103530	chr11	14761755	14868462
3726	CP026264.1	103887	109284	chr11	14759950	14761302
3727	CP026264.1	110356	112108	chr11	14619508	14758339
3728	CP026264.1	112538	158304	chr11	14156204	14160980
3729	CP026264.1	158412	164363	chr11	14023393	14121332
3730	CP026264.1	164629	193904	chr11	14000902	14023155
3731	CP026264.1	194653	236839	chr11	13996544	14000222
3732	CP026264.1	237185	238308	chr11	13966975	13996169
3733	CP026264.1	239195	247483	chr11	13944485	13966876
3734	CP026264.1	247684	250010	chr11	13933745	13944388
3735	CP026264.1	250780	252070	chr11	13929220	13932485
3736	CP026264.1	252485	255524	chr11	13923077	13929088
3737	CP026264.1	257142	261141	chr11	13912031	13922874

3738	CP026264.1	261594	265680	chr11	13891691	13911737
3739	CP026264.1	265938	274048	chr11	13813749	13891483
3740	CP026264.1	275765	277763	chr11	13765402	13813393
3741	CP026264.1	281146	311188	chr11	13756470	13765262
3742	CP026264.1	311315	312870	chr11	13656356	13756326
3743	CP026264.1	313386	316274	chr11	13642002	13656060
3744	CP026264.1	316701	321256	chr11	13551410	13641115
3745	CP026264.1	321554	327901	chr11	13525656	13550720
3746	CP026264.1	331820	399921	chr11	13465952	13525510
3747	CP026264.1	400570	402251	chr11	13433761	13465605
3748	CP026264.1	403270	407667	chr11	13414150	13433455
3749	CP026264.1	408452	427505	chr11	13396255	13413950
3750	CP026264.1	428139	429604	chr11	13386419	13395721
3751	CP026264.1	429736	483983	chr11	13344105	13386125
3752	CP026264.1	484265	489385	chr11	13206720	13343775
3753	CP026264.1	490611	492585	chr11	13205241	13206528
3754	CP026264.1	492721	500616	chr11	13169040	13205035
3755	CP026264.1	501957	504569	chr11	13135250	13168886
3756	CP026264.1	505289	522298	chr11	12836916	13135130
3757	CP026264.1	525130	534282	chr11	12824217	12836812
3758	CP026264.1	534481	537430	chr11	12739665	12823613
3759	CP026264.1	538710	561341	chr11	12691959	12739556
3760	CP026264.1	562101	607039	chr11	12675367	12690714
3761	CP026264.1	607288	611297	chr11	12665995	12675038
3762	CP026264.1	611430	614115	chr11	12663949	12665642
3763	CP026264.1	616139	625485	chr11	12611082	12663848
3764	CP026264.1	626709	629998	chr11	12552136	12610342
3765	CP026264.1	630338	637737	chr11	12471187	12550604
3766	CP026264.1	639384	640603	chr11	12322232	12471059

3767	CP026264.1	641534	654855	chr11	12316673	12322136
3768	CP026264.1	655895	667320	chr11	12228725	12316486
3769	CP026264.1	667835	679014	chr11	12113854	12228559
3770	CP026264.1	679957	698838	chr11	12079184	12113747
3771	CP026264.1	698978	703605	chr11	12069442	12079097
3772	CP026264.1	703755	704880	chr11	11961453	12069329
3773	CP026264.1	704994	723262	chr11	11911188	11961366
3774	CP026264.1	723633	728297	chr11	11905870	11911092
3775	CP026264.1	728767	734961	chr11	11830107	11904660
3776	CP026264.1	735375	737325	chr11	11495186	11825263
3777	CP026264.1	737819	742757	chr11	11495085	11410550
3778	CP026264.1	743084	748246	chr11	11410030	10973717
3779	CP026264.1	748796	750099	chr11	10971856	10943988
3780	CP026264.1	752448	754011	chr11	10943777	10914305
3781	CP026264.1	754776	758458	chr11	10914141	10868932
3782	CP026264.1	758978	760179	chr11	10868661	10556646
3783	CP026264.1	760281	761528	chr11	10471871	10552115
3784	CP026264.1	762648	778364	chr11	10468529	10471553
3785	CP026264.1	779040	780361	chr11	10459067	10468431
3786	CP026264.1	783161	791500	chr11	10456505	10458791
3787	CP026264.1	793896	799645	chr11	10405286	10455405
3788	CP026264.1	799752	803283	chr11	10336345	10404939
3789	CP026264.1	803389	806714	chr11	10296929	10334216
3790	CP026264.1	808259	812125	chr11	10278278	10296814
3791	CP026264.1	812644	814284	chr11	10229048	10278134
3792	CP026264.1	814526	825339	chr11	10166437	10228518
3793	CP026264.1	825601	834075	chr11	10060113	10165379
3794	CP026264.1	834346	837546	chr11	10033825	10057161
3795	CP026264.1	838582	840379	chr11	9987453	10033601

3796	CP026264.1	841631	843913	chr11	9964349	9985992
3797	CP026264.1	847314	849530	chr11	9922254	9964165
3798	CP026264.1	851174	854555	chr11	9898546	9922140
3799	CP026264.1	855997	859366	chr11	9883162	9898291
3800	CP026264.1	860172	861417	chr11	9876165	9880612
3801	CP026264.1	862618	871112	chr11	9844729	9868975
3802	CP026264.1	872605	882478	chr11	9825292	9844616
3803	CP026264.1	882760	906687	chr11	9793774	9825204
3804	CP026264.1	908652	910218	chr11	9728639	9793514
3805	CP026264.1	912569	923960	chr11	9682354	9728202
3806	CP026264.1	924105	932081	chr11	9669390	9682024
3807	CP026264.1	933632	936402	chr11	9662848	9669135
3808	CP026264.1	937142	939772	chr11	9657074	9662745
3809	CP026264.1	940086	951398	chr11	9650198	9656960
3810	CP026264.1	951580	964513	chr11	9648155	9650015
3811	CP026264.1	966874	976093	chr11	9625760	9647613
3812	CP026264.1	976198	978038	chr11	9617117	9625400
3813	CP026264.1	978394	982654	chr11	9599369	9616266
3814	CP026264.1	982755	983847	chr11	9562059	9599263
3815	CP026264.1	985741	994600	chr11	9451186	9560840
3816	CP026264.1	994962	1000152	chr11	9444212	9450069
3817	CP026264.1	1000658	1003956	chr11	9422018	9443769
3818	CP026264.1	1004340	1011423	chr11	9254801	9421931
3819	CP026264.1	1011751	1096932	chr11	9032980	9254711
3820	CP026264.1	1098132	1139364	chr11	8952427	9032811
3821	CP026264.1	1142849	1172469	chr11	8888993	8952340
3822	CP026264.1	1172797	1181697	chr11	8782133	8888687
3823	CP026264.1	1182192	1227591	chr11	8551429	8781963
3824	CP026264.1	1227718	1233086	chr11	8526948	8551221

3825	CP026264.1	1235214	1248530	chr11	8461301	8526791
3826	CP026264.1	1249329	1278893	chr11	8437739	8461197
3827	CP026264.1	1279813	1281209	chr11	8385673	8437649
3828	CP026264.1	1281371	1285644	chr11	8362288	8385508
3829	CP026264.1	1286636	1288617	chr11	8261008	8362154
3830	CP026264.1	1288901	1302500	chr11	8251428	8260814
3831	CP026264.1	1302685	1307568	chr11	8200422	8251181
3832	CP026264.1	1307678	1311600	chr11	8197389	8200209
3833	CP026264.1	1311732	1317023	chr11	8110179	8196855
3834	CP026264.1	1317344	1323787	chr11	8106105	8109973
3835	CP026264.1	1324873	1332807	chr11	8093250	8105603
3836	CP026264.1	1333473	1335047	chr11	8086603	8092888
3837	CP026264.1	1337231	1341232	chr11	8031382	8086262
3838	CP026264.1	1341504	1351181	chr11	8001598	8031172
3839	CP026264.1	1351448	1353833	chr11	7972960	8001276
3840	CP026264.1	1354227	1356076	chr11	7967940	7972459
3841	CP026264.1	1365908	1399357	chr11	7929472	7967678
3842	CP026264.1	1399593	1404790	chr11	7921293	7928762
3843	CP026264.1	1405059	1408490	chr11	7908962	7921188
3844	CP026264.1	1408875	1412037	chr11	7867621	7908807
3845	CP026264.1	1413186	1418292	chr11	7757600	7867398
3846	CP026264.1	1418565	1432073	chr11	7750905	7757484
3847	CP026264.1	1432241	1436186	chr11	7610915	7749896
3848	CP026264.1	1436746	1439473	chr11	7534767	7609664
3849	CP026264.1	1441402	1458321	chr11	7471092	7534141
3850	CP026264.1	1458840	1466261	chr11	7433536	7470928
3851	CP026264.1	1466481	1470917	chr11	7428427	7433450
3852	CP026264.1	1471659	1474794	chr11	7313030	7428324
3853	CP026264.1	1475407	1501384	chr11	7196400	7312505

3854	CP026264.1	1501474	1507288	chr11	7150699	7196263
3855	CP026264.1	1508313	1526365	chr11	7133428	7150596
3856	CP026264.1	1526719	1531502	chr11	7029892	7133149
3857	CP026264.1	1532324	1600342	chr11	7012592	7029562
3858	CP026264.1	1600508	1647635	chr11	6940657	7012485
3859	CP026264.1	1647789	1657854	chr11	6935195	6940408
3860	CP026264.1	1658269	1754818	chr11	6929356	6935105
3861	CP026264.1	1755760	1779433	chr11	6924052	6929262
3862	CP026264.1	1779694	1783006	chr11	6747094	6923588
3863	CP026264.1	1783327	1798479	chr11	6719839	6746946
3864	CP026264.1	1799958	1801688	chr11	6605030	6719743
3865	CP026264.1	1805195	1810155	chr11	6529352	6604789
3866	CP026264.1	1811050	1841544	chr11	6409120	6529207
3867	CP026264.1	1842011	1846199	chr11	6200031	6400626
3868	CP026264.1	1846292	1852633	chr11	6033753	6199851
3869	CP026264.1	1853506	1855004	chr11	6000053	6033622
3870	CP026264.1	1855292	1856871	chr11	5975561	5999859
3871	CP026264.1	1857218	1859329	chr11	5962161	5975011
3872	CP026264.1	1860194	1865585	chr11	5920486	5962010
3873	CP026264.1	1865703	1866744	chr11	5906206	5920317
3874	CP026264.1	1867401	1871218	chr11	5904111	5905280
3875	CP026264.1	1871937	1875468	chr11	5890292	5903843
3876	CP026264.1	1875621	1889825	chr11	5853594	5890022
3877	CP026264.1	1889983	1904455	chr11	5624255	5853447
3878	CP026264.1	1908729	1910846	chr11	5406446	5623230
3879	CP026264.1	1910971	1919673	chr11	5403729	5406191
3880	CP026264.1	1921045	1930577	chr11	5347407	5403299
3881	CP026264.1	1932106	1933684	chr11	5320761	5347227
3882	CP026264.1	1934460	1941524	chr11	5297182	5320611

3883	CP026264.1	1943866	1945291	chr11	5295088	5296970
3884	CP026264.1	1946957	1958989	chr11	5271303	5294765
3885	CP026264.1	1960107	1965972	chr11	5262963	5271127
3886	CP026264.1	1966259	1981842	chr11	5257925	5262470
3887	CP026264.1	1981929	1986363	chr11	5251389	5257760
3888	CP026264.1	1986558	2002833	chr11	5246437	5251298
3889	CP026264.1	2004636	2014133	chr11	5235351	5245656
3890	CP026264.1	2015019	2019781	chr11	5226368	5235123
3891	CP026264.1	2021283	2023780	chr11	5217487	5226055
3892	CP026264.1	2024061	2029389	chr11	5181476	5217088
3893	CP026264.1	2029685	2037790	chr11	5165209	5181296
3894	CP026264.1	2041221	2043721	chr11	5047217	5164888
3895	CP026264.1	2043948	2046864	chr11	5022340	5047078
3896	CP026264.1	2046951	2048295	chr11	5000212	5022033
3897	CP026264.1	2048389	2077449	chr11	4993441	4999945
3898	CP026264.1	2077604	2095548	chr11	4924543	4993257
3899	CP026264.1	2095950	2116991	chr11	4915354	4923284
3900	CP026264.1	2117376	2126308	chr11	4907445	4914971
3901	CP026264.1	2126792	2134137	chr11	4881818	4906856
3902	CP026264.1	2134274	2141873	chr11	4807800	4881726
3903	CP026264.1	2142016	2145762	chr11	4804522	4807656
3904	CP026264.1	2145876	2151880	chr11	4797889	4804428
3905	CP026264.1	2152327	2160028	chr11	4769170	4797686
3906	CP026264.1	2160336	2162539	chr11	4727392	4767297
3907	CP026264.1	2162743	2179979	chr11	4676032	4727164
3908	CP026264.1	2181787	2187575	chr11	4653023	4674473
3909	CP026264.1	2187771	2197805	chr11	4650487	4652907
3910	CP026264.1	2197969	2215301	chr11	4648294	4650302
3911	CP026264.1	2215470	2311109	chr11	4646801	4648195

3912	CP026264.1	2311409	2314474	chr11	4642031	4646605
3913	CP026264.1	2314629	2325389	chr11	4640488	4641833
3914	CP026264.1	2326876	2332556	chr11	4636517	4640214
3915	CP026264.1	2332651	2404128	chr11	4628477	4635284
3916	CP026264.1	2404381	2407088	chr11	4626762	4628310
3917	CP026264.1	2407459	2420476	chr11	4624501	4626673
3918	CP026264.1	2420637	2438830	chr11	4598772	4623516
3919	CP026264.1	2439092	2452314	chr11	4572390	4598654
3920	CP026264.1	2454042	2468675	chr11	4559005	4572163
3921	CP026264.1	2468933	2511662	chr11	4501537	4558504
3922	CP026264.1	2512188	2554091	chr11	4456552	4501427
3923	CP026264.1	2554227	2608148	chr11	4447193	4456352
3924	CP026264.1	2608248	2616733	chr11	4441193	4446949
3925	CP026264.1	2620973	2665768	chr11	4436744	4441083
3926	CP026264.1	2665966	2825177	chr11	4431870	4436609
3927	CP026264.1	2825978	2829754	chr11	4429359	4431062
3928	CP026264.1	2829942	2834319	chr11	4426205	4428045
3929	CP026264.1	2834602	2862607	chr11	4384709	4423111
3930	CP026264.1	2863314	2865480	chr11	4380544	4383447
3931	CP026264.1	2866097	2877264	chr11	4375046	4379303
3932	CP026264.1	2877354	2882970	chr11	4366078	4373602
3933	CP026264.1	2883281	2885329	chr11	4358554	4365974
3934	CP026264.1	2885423	2890950	chr11	4302104	4358255
3935	CP026264.1	2891137	2897260	chr11	4298618	4300903
3936	CP026264.1	2899035	2945248	chr11	4296676	4298468
3937	CP026264.1	2947522	2982761	chr11	4260984	4296459
3938	CP026264.1	2983608	2984875	chr11	4198869	4260793
3939	CP026264.1	2985611	2988381	chr11	4188316	4198730
3940	CP026264.1	2990184	2993647	chr11	4092048	4188208

3941	CP026264.1	2996132	3016262	chr11	4073820	4091843
3942	CP026264.1	3016701	3071283	chr11	4070584	4073717
3943	CP026264.1	3071512	3074925	chr11	4060676	4069656
3944	CP026264.1	3076147	3078773	chr11	4049797	4059418
3945	CP026264.1	3080185	3083878	chr11	4045232	4049144
3946	CP026264.1	3088064	3094420	chr11	4038571	4044658
3947	CP026264.1	3095822	3158297	chr11	4033883	4038214
3948	CP026264.1	3158732	3166565	chr11	4031368	4033717
3949	CP026264.1	3166850	3187675	chr11	4025691	4031258
3950	CP026264.1	3187775	3203858	chr11	4015335	4025599
3951	CP026264.1	3204017	3210291	chr11	4007472	4014872
3952	CP026264.1	3210684	3211840	chr11	3958079	4007194
3953	CP026264.1	3217421	3224646	chr11	3923181	3957989
3954	CP026264.1	3227087	3231231	chr11	3917479	3923053
3955	CP026264.1	3232575	3236608	chr11	3785117	3917118
3956	CP026264.1	3237650	3239114	chr11	3780401	3784989
3957	CP026264.1	3240935	3248467	chr11	3729866	3780199
3958	CP026264.1	3248747	3271674	chr11	3670882	3729752
3959	CP026264.1	3272061	3291800	chr11	3664051	3669095
3960	CP026264.1	3292032	3313955	chr11	3660884	3663831
3961	CP026264.1	3314431	3334049	chr11	3618879	3660671
3962	CP026264.1	3334194	3335892	chr11	3615368	3618700
3963	CP026264.1	3336060	3346667	chr11	3547811	3615002
3964	CP026264.1	3346957	3349947	chr11	3533613	3546491
3965	CP026264.1	3350263	3354633	chr11	3513432	3530404
3966	CP026264.1	3354863	3361459	chr11	3496085	3513072
3967	CP026264.1	3361559	3368474	chr11	3472251	3493344
3968	CP026264.1	3368683	3388685	chr11	3466878	3472062
3969	CP026264.1	3388851	3398333	chr11	3458058	3466778

3970	CP026264.1	3398541	3403517	chr11	3437340	3457473
3971	CP026264.1	3403718	3425659	chr11	3416328	3437187
3972	CP026264.1	3426426	3447285	chr11	3393620	3415561
3973	CP026264.1	3447438	3467571	chr11	3388443	3393419
3974	CP026264.1	3468156	3476876	chr11	3378753	3388235
3975	CP026264.1	3476976	3482160	chr11	3358585	3378587
3976	CP026264.1	3482349	3503442	chr11	3351461	3358376
3977	CP026264.1	3506183	3523170	chr11	3344765	3351361
3978	CP026264.1	3523530	3540502	chr11	3340165	3344535
3979	CP026264.1	3543711	3556589	chr11	3336859	3339849
3980	CP026264.1	3557909	3625100	chr11	3325962	3336569
3981	CP026264.1	3625466	3628798	chr11	3324096	3325794
3982	CP026264.1	3628977	3670769	chr11	3304333	3323951
3983	CP026264.1	3670982	3673929	chr11	3281934	3303857
3984	CP026264.1	3674149	3679193	chr11	3261963	3281702
3985	CP026264.1	3680980	3739850	chr11	3238649	3261576
3986	CP026264.1	3739964	3790297	chr11	3230837	3238369
3987	CP026264.1	3790499	3795087	chr11	3227552	3229016
3988	CP026264.1	3795215	3927216	chr11	3222477	3226510
3989	CP026264.1	3927577	3933151	chr11	3216989	3221133
3990	CP026264.1	3933279	3968087	chr11	3207323	3214548
3991	CP026264.1	3968177	4017292	chr11	3200586	3201742
3992	CP026264.1	4017570	4024970	chr11	3193919	3200193
3993	CP026264.1	4025433	4035697	chr11	3177677	3193760
3994	CP026264.1	4035789	4041356	chr11	3156752	3177577
3995	CP026264.1	4041466	4043815	chr11	3148634	3156467
3996	CP026264.1	4043981	4048312	chr11	3085724	3148199
3997	CP026264.1	4048669	4054756	chr11	3077966	3084322
3998	CP026264.1	4055330	4059242	chr11	3070087	3073780

3999	CP026264.1	4059895	4069516	chr11	3066049	3068675
4000	CP026264.1	4070774	4079754	chr11	3061414	3064827
4001	CP026264.1	4080682	4083815	chr11	3006603	3061185
4002	CP026264.1	4083918	4101941	chr11	2986034	3006164
4003	CP026264.1	4102146	4198306	chr11	2980086	2983549
4004	CP026264.1	4198414	4208828	chr11	2975513	2978283
4005	CP026264.1	4208967	4270891	chr11	2973510	2974777
4006	CP026264.1	4271082	4306557	chr11	2937424	2972663
4007	CP026264.1	4306774	4308566	chr11	2888937	2935150
4008	CP026264.1	4308716	4311001	chr11	2881039	2887162
4009	CP026264.1	4312202	4368353	chr11	2875325	2880852
4010	CP026264.1	4368652	4376072	chr11	2873183	2875231
4011	CP026264.1	4376176	4383700	chr11	2867256	2872872
4012	CP026264.1	4385144	4389401	chr11	2855999	2867166
4013	CP026264.1	4390642	4393545	chr11	2853216	2855382
4014	CP026264.1	4394807	4433209	chr11	2824504	2852509
4015	CP026264.1	4436303	4438143	chr11	2819844	2824221
4016	CP026264.1	4439457	4441160	chr11	2815880	2819656
4017	CP026264.1	4441968	4446707	chr11	2655868	2815079
4018	CP026264.1	4446842	4451181	chr11	2610875	2655670
4019	CP026264.1	4451291	4457047	chr11	2598150	2606635
4020	CP026264.1	4457291	4466450	chr11	2544129	2598050
4021	CP026264.1	4466650	4511525	chr11	2502090	2543993
4022	CP026264.1	4511635	4568602	chr11	2458835	2501564
4023	CP026264.1	4569103	4582261	chr11	2443944	2458577
4024	CP026264.1	4582488	4608752	chr11	2428994	2442216
4025	CP026264.1	4608870	4633614	chr11	2410539	2428732
4026	CP026264.1	4634599	4636771	chr11	2397361	2410378
4027	CP026264.1	4636860	4638408	chr11	2394283	2396990

4028	CP026264.1	4638575	4645382	chr11	2322553	2394030
4029	CP026264.1	4646615	4650312	chr11	2316778	2322458
4030	CP026264.1	4650586	4651931	chr11	2304531	2315291
4031	CP026264.1	4652129	4656703	chr11	2301311	2304376
4032	CP026264.1	4656899	4658293	chr11	2205372	2301011
4033	CP026264.1	4658392	4660400	chr11	2187871	2205203
4034	CP026264.1	4660585	4663005	chr11	2177673	2187707
4035	CP026264.1	4663121	4684571	chr11	2171689	2177477
4036	CP026264.1	4686130	4737262	chr11	2152645	2169881
4037	CP026264.1	4737490	4777395	chr11	2150238	2152441
4038	CP026264.1	4779268	4807784	chr11	2142229	2149930
4039	CP026264.1	4807987	4814526	chr11	2135778	2141782
4040	CP026264.1	4814620	4817754	chr11	2131918	2135664
4041	CP026264.1	4817898	4891824	chr11	2124176	2131775
4042	CP026264.1	4891916	4916954	chr11	2116694	2124039
4043	CP026264.1	4917543	4925069	chr11	2107278	2116210
4044	CP026264.1	4925452	4933382	chr11	2085852	2106893
4045	CP026264.1	4934641	5003355	chr11	2067506	2085450
4046	CP026264.1	5003539	5010043	chr11	2038291	2067351
4047	CP026264.1	5010310	5032131	chr11	2036853	2038197
4048	CP026264.1	5032438	5057176	chr11	2033850	2036766
4049	CP026264.1	5057315	5174986	chr11	2031123	2033623
4050	CP026264.1	5175307	5191394	chr11	2019587	2027692
4051	CP026264.1	5191574	5227186	chr11	2013963	2019291
4052	CP026264.1	5227585	5236153	chr11	2011185	2013682
4053	CP026264.1	5236466	5245221	chr11	2004921	2009683
4054	CP026264.1	5245449	5255754	chr11	1994538	2004035
4055	CP026264.1	5256535	5261396	chr11	1976460	1992735
4056	CP026264.1	5261487	5267858	chr11	1971831	1976265

4057	CP026264.1	5268023	5272568	chr11	1956161	1971744
4058	CP026264.1	5273061	5281225	chr11	1950009	1955874
4059	CP026264.1	5281401	5304863	chr11	1936859	1948891
4060	CP026264.1	5305186	5307068	chr11	1933768	1935193
4061	CP026264.1	5307280	5330709	chr11	1924362	1931426
4062	CP026264.1	5330859	5357325	chr11	1922008	1923586
4063	CP026264.1	5357505	5413397	chr11	1910947	1920479
4064	CP026264.1	5413827	5416289	chr11	1900873	1909575
4065	CP026264.1	5416544	5633328	chr11	1898631	1900748
4066	CP026264.1	5634353	5863545	chr11	1879885	1894357
4067	CP026264.1	5863692	5900120	chr11	1865523	1879727
4068	CP026264.1	5900390	5913941	chr11	1861839	1865370
4069	CP026264.1	5914209	5915378	chr11	1857303	1861120
4070	CP026264.1	5916304	5930415	chr11	1855605	1856646
4071	CP026264.1	5930584	5972108	chr11	1850096	1855487
4072	CP026264.1	5972259	5985109	chr11	1847120	1849231
4073	CP026264.1	5985659	6009957	chr11	1845194	1846773
4074	CP026264.1	6010151	6043720	chr11	1843408	1844906
4075	CP026264.1	6043851	6209949	chr11	1836194	1842535
4076	CP026264.1	6210129	6410724	chr11	1831913	1836101
4077	CP026264.1	6419218	6539305	chr11	1800952	1831446
4078	CP026264.1	6539450	6614887	chr11	1795097	1800057
4079	CP026264.1	6615128	6729841	chr11	1789860	1791590
4080	CP026264.1	6729937	6757044	chr11	1773229	1788381
4081	CP026264.1	6757192	6933686	chr11	1769596	1772908
4082	CP026264.1	6934150	6939360	chr11	1745662	1769335
4083	CP026264.1	6939454	6945203	chr11	1648171	1744720
4084	CP026264.1	6945293	6950506	chr11	1637691	1647756
4085	CP026264.1	6950755	7022583	chr11	1590410	1637537

4086	CP026264.1	7022690	7039660	chr11	1522226	1590244
4087	CP026264.1	7039990	7143247	chr11	1516621	1521404
4088	CP026264.1	7143526	7160694	chr11	1498215	1516267
4089	CP026264.1	7160797	7206361	chr11	1491376	1497190
4090	CP026264.1	7206498	7322603	chr11	1465309	1491286
4091	CP026264.1	7323128	7438422	chr11	1461561	1464696
4092	CP026264.1	7438525	7443548	chr11	1456383	1460819
4093	CP026264.1	7443634	7481026	chr11	1448742	1456163
4094	CP026264.1	7481190	7544239	chr11	1431304	1448223
4095	CP026264.1	7544865	7619762	chr11	1426648	1429375
4096	CP026264.1	7621013	7759994	chr11	1422143	1426088
4097	CP026264.1	7761003	7767582	chr11	1408467	1421975
4098	CP026264.1	7767698	7877496	chr11	1403088	1408194
4099	CP026264.1	7877719	7918905	chr11	1398777	1401939
4100	CP026264.1	7919060	7931286	chr11	1394961	1398392
4101	CP026264.1	7931391	7938860	chr11	1389495	1394692
4102	CP026264.1	7939570	7977776	chr11	1355810	1389259
4103	CP026264.1	7978038	7982557	chr11	1353860	1355709
4104	CP026264.1	7983058	8011374	chr11	1351081	1353466
4105	CP026264.1	8011696	8041270	chr11	1341137	1350814
4106	CP026264.1	8041480	8096360	chr11	1336864	1340865
4107	CP026264.1	8096701	8102986	chr11	1333106	1334680
4108	CP026264.1	8103348	8115701	chr11	1324506	1332440
4109	CP026264.1	8116203	8120071	chr11	1316977	1323420
4110	CP026264.1	8120277	8206953	chr11	1311365	1316656
4111	CP026264.1	8207487	8210307	chr11	1307311	1311233
4112	CP026264.1	8210520	8261279	chr11	1302318	1307201
4113	CP026264.1	8261526	8270912	chr11	1288534	1302133
4114	CP026264.1	8271106	8372252	chr11	1286269	1288250

4115	CP026264.1	8372386	8395606	chr11	1281004	1285277
4116	CP026264.1	8395771	8447747	chr11	1279446	1280842
4117	CP026264.1	8447837	8471295	chr11	1248962	1278526
4118	CP026264.1	8471399	8536889	chr11	1234847	1248163
4119	CP026264.1	8537046	8561319	chr11	1227351	1232719
4120	CP026264.1	8561527	8792061	chr11	1181825	1227224
4121	CP026264.1	8792231	8898785	chr11	1172430	1181330
4122	CP026264.1	8899091	8962438	chr11	1142482	1172102
4123	CP026264.1	8962525	9042909	chr11	1097765	1138997
4124	CP026264.1	9043078	9264809	chr11	1011384	1096565
4125	CP026264.1	9264899	9432029	chr11	1003973	1011056
4126	CP026264.1	9432116	9453867	chr11	1000291	1003589
4127	CP026264.1	9454310	9460167	chr11	994595	999785
4128	CP026264.1	9461284	9570938	chr11	985374	994233
4129	CP026264.1	9572157	9609361	chr11	982388	983480
4130	CP026264.1	9609467	9626364	chr11	978027	982287
4131	CP026264.1	9627215	9635498	chr11	975831	977671
4132	CP026264.1	9635858	9657711	chr11	966507	975726
4133	CP026264.1	9658253	9660113	chr11	951213	964146
4134	CP026264.1	9660296	9667058	chr11	939719	951031
4135	CP026264.1	9667172	9672843	chr11	936775	939405
4136	CP026264.1	9672946	9679233	chr11	933265	936035
4137	CP026264.1	9679488	9692122	chr11	923738	931714
4138	CP026264.1	9692452	9738300	chr11	912202	923593
4139	CP026264.1	9738737	9803612	chr11	908285	909851
4140	CP026264.1	9803872	9835302	chr11	882393	906320
4141	CP026264.1	9835390	9854714	chr11	872238	882111
4142	CP026264.1	9854827	9879073	chr11	862251	870745
4143	CP026264.1	9886263	9890710	chr11	859805	861050

4144	CP026264.1	9893260	9908389	chr11	855630	858999
4145	CP026264.1	9908644	9932238	chr11	850807	854188
4146	CP026264.1	9932352	9974263	chr11	846947	849163
4147	CP026264.1	9974447	9996090	chr11	841264	843546
4148	CP026264.1	9997551	10043699	chr11	838215	840012
4149	CP026264.1	10043923	10067259	chr11	833979	837179
4150	CP026264.1	10070211	10175477	chr11	825234	833708
4151	CP026264.1	10176535	10238616	chr11	814159	824972
4152	CP026264.1	10239146	10288232	chr11	812277	813917
4153	CP026264.1	10288376	10306912	chr11	807892	811758
4154	CP026264.1	10307027	10344314	chr11	803022	806347
4155	CP026264.1	10346443	10415037	chr11	799385	802916
4156	CP026264.1	10415384	10465503	chr11	793529	799278
4157	CP026264.1	10466603	10468889	chr11	782794	791133
4158	CP026264.1	10469165	10478529	chr11	778673	779994
4159	CP026264.1	10478627	10481651	chr11	762281	777997
4160	CP026264.1	10481969	10562213	chr11	759914	761161
4161	CP026264.1	10565752	10567445	chr11	758611	759812
4162	CP026264.1	10567798	10576841	chr11	754409	758091
4163	CP026264.1	10577170	10592517	chr11	752081	753644
4164	CP026264.1	10593762	10641359	chr11	748429	749732
4165	CP026264.1	10641468	10725416	chr11	742717	747879
4166	CP026264.1	10726020	10738615	chr11	737452	742390
4167	CP026264.1	10738719	11036933	chr11	735008	736958
4168	CP026264.1	11037053	11070689	chr11	728400	734594
4169	CP026264.1	11070843	11106838	chr11	723266	727930
4170	CP026264.1	11107044	11108331	chr11	704627	722895
4171	CP026264.1	11108523	11245578	chr11	703388	704513
4172	CP026264.1	11245908	11287928	chr11	698611	703238

4173	CP026264.1	11288222	11297524	chr11	679590	698471
4174	CP026264.1	11298058	11315753	chr11	667468	678647
4175	CP026264.1	11315953	11335258	chr11	655528	666953
4176	CP026264.1	11335564	11367408	chr11	641167	654488
4177	CP026264.1	11367755	11427313	chr11	639017	640236
4178	CP026264.1	11427459	11452523	chr11	629971	637370
4179	CP026264.1	11453213	11542918	chr11	626342	629631
4180	CP026264.1	11543805	11557863	chr11	615772	625118
4181	CP026264.1	11558159	11658129	chr11	611063	613748
4182	CP026264.1	11658273	11667065	chr11	606921	610930
4183	CP026264.1	11667205	11715196	chr11	561734	606672
4184	CP026264.1	11715552	11793286	chr11	538343	560974
4185	CP026264.1	11793494	11813540	chr11	534114	537063
4186	CP026264.1	11813834	11824677	chr11	524763	533915
4187	CP026264.1	11824880	11830891	chr11	504922	521931
4188	CP026264.1	11831023	11834288	chr11	501590	504202
4189	CP026264.1	11835548	11846191	chr11	492354	500249
4190	CP026264.1	11846288	11868679	chr11	490244	492218
4191	CP026264.1	11868778	11897972	chr11	483898	489018
4192	CP026264.1	11898347	11902025	chr11	429369	483616
4193	CP026264.1	11902705	11924958	chr11	427772	429237
4194	CP026264.1	11925196	12023135	chr11	408085	427138
4195	CP026264.1	12058007	12062783	chr11	402903	407300
4196	CP026264.1	12202957	12287492	chr11	400203	401884
4197	CP026264.1	12288012	12724325	chr11	331453	399554
4198	CP026264.1	12726186	12754054	chr11	321187	327534
4199	CP026264.1	12754265	12783737	chr11	316334	320889
4200	CP026264.1	12783901	12829110	chr11	313019	315907
4201	CP026264.1	12829381	13141396	chr11	310948	312503

4202	CP026264.1	13141497	13471574	chr11	280779	310821
4203	CP026264.1	13476418	13550971	chr11	275398	277396
4204	CP026264.1	13552181	13557403	chr11	265571	273681
4205	CP026264.1	13557499	13607677	chr11	261227	265313
4206	CP026264.1	13607764	13715640	chr11	256775	260774
4207	CP026264.1	13715753	13725408	chr11	252118	255157
4208	CP026264.1	13725495	13760058	chr11	250413	251703
4209	CP026264.1	13760165	13874870	chr11	247317	249643
4210	CP026264.1	13875036	13962797	chr11	238828	247116
4211	CP026264.1	13962984	13968447	chr11	236818	237941
4212	CP026264.1	13968543	14117370	chr11	194286	236472
4213	CP026264.1	14117498	14196915	chr11	164262	193537
4214	CP026264.1	14198447	14256653	chr11	158045	163996
4215	CP026264.1	14257393	14310159	chr11	112171	157937
4216	CP026264.1	14310260	14330522	chr11	109989	111741
4217	CP026264.1	14346806	14499842	chr11	103520	108917
4218	CP026264.1	14499951	14501303	chr11	96472	103163
4219	CP026264.1	14501408	14503739	chr11	94031	96029
4220	CP026264.1	14505063	14611381	chr11	64747	93631
4221	CP026264.1	14611891	14613776	chr11	40079	64248
4222	CP026264.1	14617317	14620551	chr11	23691	39616
4223	CP026264.1	14622766	14669654	chr11	21168	22696
4224	CP026264.1	14669755	14808586	chr11	4340	20384
4225	CP026264.1	14810197	14811549	chr11	2547	3757
4226	CP026264.1	14812002	14918709	chr11	1	1321
4227	CP026257.1	4270946	4282723	chr12	11588680	11694087
4228	CP026257.1	4282820	4317135	chr12	11535701	11588274
4229	CP026257.1	4317492	4336184	chr12	11280050	11535495
4230	CP026257.1	4336655	4354591	chr12	11277770	11279789

4231	CP026257.1	4355819	4489723	chr12	11252509	11276617
4232	CP026257.1	4489952	4560751	chr12	11240905	11252059
4233	CP026257.1	4560843	4906490	chr12	11138701	11240733
4234	CP026257.1	4906580	4910167	chr12	11047383	11138600
4235	CP026257.1	4910255	4949136	chr12	11014809	11047258
4236	CP026257.1	4949260	4981580	chr12	11002786	11013755
4237	CP026257.1	4982813	5096911	chr12	10904996	11002558
4238	CP026257.1	5097211	5132150	chr12	10856975	10904133
4239	CP026257.1	5132776	5222474	chr12	10843706	10856718
4240	CP026257.1	5222938	5264861	chr12	10812454	10842471
4241	CP026257.1	5264960	5267365	chr12	10705420	10811970
4242	CP026257.1	5267454	5273617	chr12	10691097	10705290
4243	CP026257.1	5274624	5276183	chr12	10418075	10690992
4244	CP026257.1	5276579	5433716	chr12	9854902	10417983
4245	CP026257.1	5434366	5436007	chr12	9806207	9853622
4246	CP026257.1	5436221	5466260	chr12	9794221	9806078
4247	CP026257.1	5466868	5493553	chr12	9650503	9793914
4248	CP026257.1	5493932	5511380	chr12	9586199	9650001
4249	CP026257.1	5511474	5533185	chr12	9548639	9586079
4250	CP026257.1	5533351	5536866	chr12	9540062	9548327
4251	CP026257.1	5536972	5569497	chr12	9299163	9539966
4252	CP026257.1	5571396	5665565	chr12	9245453	9299029
4253	CP026257.1	5665763	5782769	chr12	9233298	9242697
4254	CP026257.1	5782901	5835998	chr12	9231191	9233197
4255	CP026257.1	5836093	5839533	chr12	9229977	9230657
4256	CP026257.1	5839908	5905834	chr12	9158189	9229880
4257	CP026257.1	5905958	5968629	chr12	9116939	9157869
4258	CP026257.1	5969472	5993100	chr12	9100393	9116806
4259	CP026257.1	5993337	6111929	chr12	9070992	9100265

4260	CP026257.1	6112237	6220331	chr12	8909414	9070849
4261	CP026257.1	6220453	6256800	chr12	8702721	8907587
4262	CP026257.1	6257093	6301277	chr12	8676831	8701378
4263	CP026257.1	6301508	6335857	chr12	8600624	8676678
4264	CP026257.1	6336707	6366371	chr12	8576922	8599893
4265	CP026257.1	6366574	6427512	chr12	8481312	8576679
4266	CP026257.1	6428406	6484521	chr12	8462189	8481163
4267	CP026257.1	6485979	6538170	chr12	8415020	8462083
4268	CP026257.1	6538355	6563033	chr12	8372152	8414509
4269	CP026257.1	6563165	6622427	chr12	8338923	8371266
4270	CP026257.1	6622656	6822632	chr12	8251897	8338693
4271	CP026257.1	6822828	6844083	chr12	8048024	8251457
4272	CP026257.1	6844174	6856007	chr12	8037111	8047062
4273	CP026257.1	6856144	6894774	chr12	7976655	8036748
4274	CP026257.1	6894909	6980583	chr12	7941409	7976357
4275	CP026257.1	6980750	6982899	chr12	7875739	7940446
4276	CP026257.1	6983099	6991854	chr12	7827890	7875080
4277	CP026257.1	6992057	7090961	chr12	7658412	7827569
4278	CP026257.1	7091203	7096303	chr12	7487428	7657936
4279	CP026257.1	7096454	7136051	chr12	7426376	7487195
4280	CP026257.1	7136156	7162749	chr12	7275135	7426067
4281	CP026257.1	7162973	7190677	chr12	7254671	7274850
4282	CP026257.1	7192207	7199429	chr12	6973692	7254582
4283	CP026257.1	7199948	7206053	chr12	6967078	6973183
4284	CP026257.1	7206562	7487452	chr12	6959337	6966559
4285	CP026257.1	7487541	7507720	chr12	6930103	6957807
4286	CP026257.1	7508005	7658937	chr12	6903286	6929879
4287	CP026257.1	7659246	7720065	chr12	6863584	6903181
4288	CP026257.1	7720298	7890806	chr12	6858333	6863433

4289	CP026257.1	7891282	8060439	chr12	6759187	6858091
4290	CP026257.1	8060760	8107950	chr12	6750229	6758984
4291	CP026257.1	8108609	8173316	chr12	6747880	6750029
4292	CP026257.1	8174279	8209227	chr12	6662039	6747713
4293	CP026257.1	8209525	8269618	chr12	6623274	6661904
4294	CP026257.1	8269981	8279932	chr12	6611304	6623137
4295	CP026257.1	8280894	8484327	chr12	6589958	6611213
4296	CP026257.1	8484767	8571563	chr12	6389786	6589762
4297	CP026257.1	8571793	8604136	chr12	6330295	6389557
4298	CP026257.1	8605022	8647379	chr12	6305485	6330163
4299	CP026257.1	8647890	8694953	chr12	6253109	6305300
4300	CP026257.1	8695059	8714033	chr12	6195536	6251651
4301	CP026257.1	8714182	8809549	chr12	6133704	6194642
4302	CP026257.1	8809792	8832763	chr12	6103837	6133501
4303	CP026257.1	8833494	8909548	chr12	6068638	6102987
4304	CP026257.1	8909701	8934248	chr12	6024223	6068407
4305	CP026257.1	8935591	9140457	chr12	5987583	6023930
4306	CP026257.1	9142284	9303719	chr12	5879367	5987461
4307	CP026257.1	9303862	9333135	chr12	5760467	5879059
4308	CP026257.1	9333263	9349676	chr12	5736602	5760230
4309	CP026257.1	9349809	9390739	chr12	5673088	5735759
4310	CP026257.1	9391059	9462750	chr12	5607038	5672964
4311	CP026257.1	9462847	9463527	chr12	5603223	5606663
4312	CP026257.1	9464061	9466067	chr12	5550031	5603128
4313	CP026257.1	9466168	9475567	chr12	5432893	5549899
4314	CP026257.1	9478323	9531899	chr12	5338526	5432695
4315	CP026257.1	9532033	9772836	chr12	5304102	5336627
4316	CP026257.1	9772932	9781197	chr12	5300481	5303996
4317	CP026257.1	9781509	9818949	chr12	5278604	5300315

4318	CP026257.1	9819069	9882871	chr12	5261062	5278510
4319	CP026257.1	9883373	10026784	chr12	5233998	5260683
4320	CP026257.1	10027091	10038948	chr12	5203351	5233390
4321	CP026257.1	10039077	10086492	chr12	5201496	5203137
4322	CP026257.1	10087772	10650853	chr12	5043709	5200846
4323	CP026257.1	10650945	10923862	chr12	5041754	5043313
4324	CP026257.1	10923967	10938160	chr12	5034584	5040747
4325	CP026257.1	10938290	11044840	chr12	5032090	5034495
4326	CP026257.1	11045324	11075341	chr12	4990068	5031991
4327	CP026257.1	11076576	11089588	chr12	4899906	4989604
4328	CP026257.1	11089845	11137003	chr12	4864341	4899280
4329	CP026257.1	11137866	11235428	chr12	4749943	4864041
4330	CP026257.1	11235656	11246625	chr12	4716390	4748710
4331	CP026257.1	11247679	11280128	chr12	4677385	4716266
4332	CP026257.1	11280253	11371470	chr12	4673710	4677297
4333	CP026257.1	11371571	11473603	chr12	4327973	4673620
4334	CP026257.1	11473775	11484929	chr12	4257082	4327881
4335	CP026257.1	11485379	11509487	chr12	4122949	4256853
4336	CP026257.1	11510640	11512659	chr12	4103785	4121721
4337	CP026257.1	11512920	11768365	chr12	4084622	4103314
4338	CP026257.1	11768571	11821144	chr12	4049950	4084265
4339	CP026257.1	11821550	11926957	chr12	4038076	4049853
4340	CP026250.1	177066	177340	chr13	17546962	17549005
4341	CP026250.1	178210	178484	chr13	17541976	17545111
4342	CP026250.1	1022579	1034077	chr13	17538689	17541798
4343	CP026250.1	1037588	1050336	chr13	17535795	17538084
4344	CP026250.1	1051160	1055828	chr13	17532442	17534696
4345	CP026250.1	1058137	1067065	chr13	17529493	17530971
4346	CP026250.1	1067249	1075848	chr13	17521707	17529377

4347	CP026250.1	1076190	1085235	chr13	17501405	17521391
4348	CP026250.1	1085964	1093563	chr13	17473118	17499120
4349	CP026250.1	1094537	1098243	chr13	17465594	17472775
4350	CP026250.1	1098356	1104502	chr13	17441538	17465475
4351	CP026250.1	1104603	1132891	chr13	17432225	17436960
4352	CP026250.1	1134491	1135880	chr13	17431671	17432007
4353	CP026250.1	1136736	1138914	chr13	17419450	17431551
4354	CP026250.1	1140069	1161816	chr13	17415233	17418668
4355	CP026250.1	1162195	1188815	chr13	17400270	17408465
4356	CP026250.1	1189321	1191068	chr13	17389954	17399496
4357	CP026250.1	1191170	1192698	chr13	17378587	17389076
4358	CP026250.1	1192876	1193666	chr13	17375697	17378297
4359	CP026250.1	1193824	1195392	chr13	17350501	17374684
4360	CP026250.1	1196197	1197688	chr13	17344016	17350136
4361	CP026250.1	1198216	1200637	chr13	17336002	17343901
4362	CP026250.1	1202729	1209343	chr13	17210945	17333382
4363	CP026250.1	1210071	1211256	chr13	17164623	17210851
4364	CP026250.1	1211432	1212527	chr13	17113664	17164472
4365	CP026250.1	1212658	1219317	chr13	17088722	17112928
4366	CP026250.1	1219601	1255641	chr13	17018310	17088366
4367	CP026250.1	1256235	1266538	chr13	16948735	17017024
4368	CP026250.1	1267576	1271488	chr13	16932636	16948591
4369	CP026250.1	1271805	1284122	chr13	16896034	16932342
4370	CP026250.1	1285244	1293330	chr13	16819753	16895894
4371	CP026250.1	1296178	1302573	chr13	16792973	16818438
4372	CP026250.1	1303118	1316812	chr13	16688387	16792887
4373	CP026250.1	1317186	1320242	chr13	16652651	16687911
4374	CP026250.1	1320974	1327799	chr13	16631084	16651337
4375	CP026250.1	1328151	1330193	chr13	16599989	16629967

4376	CP026250.1	1330284	1367685	chr13	16589465	16599886
4377	CP026250.1	1370291	1373443	chr13	16546526	16589207
4378	CP026250.1	1373557	1374943	chr13	16534529	16543606
4379	CP026250.1	1376515	1393884	chr13	16446838	16534413
4380	CP026250.1	1394256	1407493	chr13	16426025	16446223
4381	CP026250.1	1408776	1443036	chr13	16356228	16425865
4382	CP026250.1	1443302	1445245	chr13	16337143	16356127
4383	CP026250.1	1446423	1448966	chr13	16292396	16335850
4384	CP026250.1	1449808	1471198	chr13	16251012	16291714
4385	CP026250.1	1471357	1490634	chr13	16173884	16250902
4386	CP026250.1	1490896	1513671	chr13	16162256	16173495
4387	CP026250.1	1513803	1521613	chr13	16047389	16162099
4388	CP026250.1	1522759	1545689	chr13	16035089	16047201
4389	CP026250.1	1545859	1581407	chr13	16026808	16034638
4390	CP026250.1	1581591	1611889	chr13	16007953	16025470
4391	CP026250.1	1613557	1616490	chr13	15990333	16007835
4392	CP026250.1	1616576	1635281	chr13	15988948	15990193
4393	CP026250.1	1635610	1636934	chr13	15885003	15988828
4394	CP026250.1	1637817	1639551	chr13	15852375	15883135
4395	CP026250.1	2825156	2829931	chr13	15531839	15851152
4396	CP026250.1	2830312	2832835	chr13	15286745	15531549
4397	CP026250.1	2836912	2839086	chr13	15251396	15286579
4398	CP026250.1	2840483	2860149	chr13	15248431	15251272
4399	CP026250.1	2860250	2861547	chr13	15216458	15248261
4400	CP026250.1	2862611	2867745	chr13	15135834	15216227
4401	CP026250.1	2868452	2889620	chr13	15007362	15135660
4402	CP026250.1	2890683	2892938	chr13	14941322	15007054
4403	CP026250.1	2893763	2895583	chr13	14852819	14940870
4404	CP026250.1	2896011	2918797	chr13	14845305	14851685

4405	CP026250.1	2920242	2952381	chr13	14779571	14845053
4406	CP026250.1	2952616	2962055	chr13	14646538	14779465
4407	CP026250.1	2962188	2985654	chr13	14616019	14646426
4408	CP026250.1	2986548	2994221	chr13	14614634	14615738
4409	CP026250.1	2994315	3002698	chr13	14544830	14614497
4410	CP026250.1	3003461	3006924	chr13	14453021	14544375
4411	CP026250.1	3007391	3015927	chr13	14390919	14452918
4412	CP026250.1	3017698	3024834	chr13	14363698	14390502
4413	CP026250.1	3026455	3037108	chr13	14317901	14363472
4414	CP026250.1	3037292	3039075	chr13	14308446	14317799
4415	CP026250.1	3039317	3041005	chr13	14261425	14308357
4416	CP026250.1	3041722	3043255	chr13	14078464	14261332
4417	CP026250.1	3043996	3049430	chr13	14048620	14078111
4418	CP026250.1	3053436	3065113	chr13	14000610	14048182
4419	CP026250.1	3065911	3178625	chr13	13875563	13999472
4420	CP026250.1	3179708	3188362	chr13	13864394	13874517
4421	CP026250.1	3189206	3212311	chr13	13746183	13864191
4422	CP026250.1	3213262	3275157	chr13	13743765	13746015
4423	CP026250.1	3275450	3342466	chr13	13691208	13743537
4424	CP026250.1	3344010	3356791	chr13	13525970	13690434
4425	CP026250.1	3357972	3382206	chr13	13502148	13525882
4426	CP026250.1	3382324	3396849	chr13	13456424	13502062
4427	CP026250.1	3397032	3404773	chr13	13364609	13456297
4428	CP026250.1	3404952	3425826	chr13	13271403	13363149
4429	CP026250.1	3425976	3446071	chr13	13249684	13271235
4430	CP026250.1	3447045	3462627	chr13	13167971	13249365
4431	CP026250.1	3462855	3475207	chr13	13152236	13167823
4432	CP026250.1	3475321	3476793	chr13	13147784	13150416
4433	CP026250.1	3477025	3478898	chr13	13088614	13147695

4434	CP026250.1	3479074	3520341	chr13	13027470	13088453
4435	CP026250.1	3520562	3523633	chr13	13011173	13027349
4436	CP026250.1	3524005	3525171	chr13	12971034	13011059
4437	CP026250.1	3525470	3536807	chr13	12946763	12970760
4438	CP026250.1	3537748	3545982	chr13	12836631	12946637
4439	CP026250.1	3546218	3554537	chr13	12793926	12836451
4440	CP026250.1	3555928	3563240	chr13	12701257	12792738
4441	CP026250.1	3564470	3575721	chr13	12537395	12700647
4442	CP026250.1	3577699	3579397	chr13	12426588	12537112
4443	CP026250.1	3579511	3582221	chr13	12406000	12426460
4444	CP026250.1	3582652	3585871	chr13	12318413	12405301
4445	CP026250.1	3586696	3630553	chr13	12305215	12318302
4446	CP026250.1	3630699	3688226	chr13	12236073	12305013
4447	CP026250.1	3689208	3690457	chr13	12069667	12235736
4448	CP026250.1	3690637	3717759	chr13	11862193	12069462
4449	CP026250.1	3718037	3723748	chr13	11842121	11862071
4450	CP026250.1	3726031	3728300	chr13	11819417	11841996
4451	CP026250.1	3728989	3734077	chr13	11807098	11819113
4452	CP026250.1	3734371	3738890	chr13	11794710	11806969
4453	CP026250.1	3739130	3740551	chr13	11728270	11794591
4454	CP026250.1	3741569	3742851	chr13	11690810	11728183
4455	CP026250.1	3743026	3745097	chr13	11684981	11690712
4456	CP026250.1	3745198	3754148	chr13	11668908	11684879
4457	CP026250.1	3754461	3777314	chr13	11638300	11668773
4458	CP026250.1	3782107	3791655	chr13	11563231	11637974
4459	CP026250.1	3791862	3809646	chr13	11527654	11563138
4460	CP026250.1	3809867	3818771	chr13	11482257	11523728
4461	CP026250.1	3819436	3823651	chr13	11459474	11481635
4462	CP026250.1	3827272	3853978	chr13	11372909	11459256

4463	CP026250.1	3854111	3871518	chr13	11295343	11372742
4464	CP026250.1	3872916	3887044	chr13	11281470	11295119
4465	CP026250.1	3887140	3934733	chr13	11177014	11281252
4466	CP026250.1	3935772	3946845	chr13	11089871	11176777
4467	CP026250.1	3947049	3993086	chr13	10990780	11089676
4468	CP026250.1	3993321	4020685	chr13	10966718	10990569
4469	CP026250.1	4021244	4038175	chr13	10944395	10966348
4470	CP026250.1	4038280	4040861	chr13	10920826	10944126
4471	CP026250.1	4042715	4044438	chr13	10899274	10920306
4472	CP026250.1	4044607	4050195	chr13	10879970	10899176
4473	CP026250.1	4050627	4053332	chr13	10832564	10879840
4474	CP026250.1	4053449	4058841	chr13	10830422	10831731
4475	CP026250.1	4058993	4078183	chr13	10799083	10830317
4476	CP026250.1	4080313	4088886	chr13	10778109	10798904
4477	CP026250.1	4089551	4097469	chr13	10744599	10777954
4478	CP026250.1	4097967	4099456	chr13	10742348	10744283
4479	CP026250.1	4099621	4108887	chr13	10681226	10742206
4480	CP026250.1	4109032	4111651	chr13	10679097	10680926
4481	CP026250.1	4112474	4115959	chr13	10412032	10678592
4482	CP026250.1	4116379	4128044	chr13	10406118	10411546
4483	CP026250.1	4129116	4134638	chr13	10322924	10405804
4484	CP026250.1	4134818	4149440	chr13	10268048	10322476
4485	CP026250.1	4149547	4162469	chr13	10242429	10267527
4486	CP026250.1	4164528	4167117	chr13	10161301	10241931
4487	CP026250.1	4167220	4173351	chr13	9937679	10161058
4488	CP026250.1	4173729	4177772	chr13	9926781	9937578
4489	CP026250.1	4177901	4204577	chr13	9880064	9926596
4490	CP026250.1	4205117	4212430	chr13	9864920	9879704
4491	CP026250.1	4212731	4220049	chr13	9697180	9863980

4492	CP026250.1	4220216	4243129	chr13	9629753	9697032
4493	CP026250.1	4243563	4293105	chr13	9585689	9629547
4494	CP026250.1	4293390	4360046	chr13	9283742	9585575
4495	CP026250.1	4360185	4388800	chr13	9214263	9283635
4496	CP026250.1	4389028	4413260	chr13	9155232	9213265
4497	CP026250.1	4413931	4426955	chr13	9140094	9154930
4498	CP026250.1	4427655	4430934	chr13	9131228	9139898
4499	CP026250.1	4431037	4435010	chr13	9112270	9131065
4500	CP026250.1	4435141	4479014	chr13	9072191	9112154
4501	CP026250.1	4480249	4485433	chr13	9044351	9071449
4502	CP026250.1	4488910	4525641	chr13	9042027	9044119
4503	CP026250.1	4525844	4552746	chr13	9033178	9041899
4504	CP026250.1	4552848	4564183	chr13	8961162	9032913
4505	CP026250.1	4564289	4583008	chr13	8915345	8960985
4506	CP026250.1	4584004	4586982	chr13	8908126	8914655
4507	CP026250.1	4587123	4587507	chr13	8762196	8907988
4508	CP026250.1	4587756	4604373	chr13	8601033	8762079
4509	CP026250.1	4604540	4632794	chr13	8591909	8600706
4510	CP026250.1	4634647	4669206	chr13	8583170	8591586
4511	CP026250.1	4669785	4696892	chr13	8550526	8582977
4512	CP026250.1	4697002	4724722	chr13	8514398	8550279
4513	CP026250.1	4724967	4727842	chr13	8494757	8514260
4514	CP026250.1	4728663	4788182	chr13	8438067	8483486
4515	CP026250.1	4788325	4790752	chr13	8409396	8437536
4516	CP026250.1	4792042	4793575	chr13	8276003	8409160
4517	CP026250.1	4793731	4797470	chr13	8274095	8275658
4518	CP026250.1	4799197	4809347	chr13	8073048	8274007
4519	CP026250.1	4810134	4826650	chr13	8030915	8072896
4520	CP026250.1	4826816	4835032	chr13	8011885	8030803

4521	CP026250.1	4835359	4839712	chr13	7798438	8011012
4522	CP026250.1	4841147	4842640	chr13	7655065	7797567
4523	CP026250.1	4844951	4924340	chr13	7647028	7654891
4524	CP026250.1	4926056	4943094	chr13	7436682	7646866
4525	CP026250.1	4944322	4977640	chr13	7398460	7436512
4526	CP026250.1	4978187	4987741	chr13	7349801	7397494
4527	CP026250.1	4988028	4998751	chr13	7311078	7349652
4528	CP026250.1	4998959	5003197	chr13	7231564	7310947
4529	CP026250.1	5003508	5008230	chr13	7195684	7231246
4530	CP026250.1	5008317	5138526	chr13	7107931	7195511
4531	CP026250.1	5142426	5148455	chr13	7101669	7106149
4532	CP026250.1	5148640	5148827	chr13	7088498	7101456
4533	CP026250.1	5148934	5149917	chr13	7034907	7088333
4534	CP026250.1	5150539	5156922	chr13	7021206	7034802
4535	CP026250.1	5157015	5164499	chr13	6931487	7020977
4536	CP026250.1	5164841	5169965	chr13	6901303	6931358
4537	CP026250.1	5170079	5173510	chr13	6898477	6900707
4538	CP026250.1	5173666	5198924	chr13	6896778	6898194
4539	CP026250.1	5199086	5213603	chr13	6874446	6896592
4540	CP026250.1	5213736	5222717	chr13	6821062	6874137
4541	CP026250.1	5222837	5257495	chr13	6813146	6819799
4542	CP026250.1	5257779	5261811	chr13	6810277	6812872
4543	CP026250.1	5261998	5278123	chr13	6731937	6809774
4544	CP026250.1	5278212	5295946	chr13	6723559	6731742
4545	CP026250.1	5296037	5306294	chr13	6629698	6723420
4546	CP026250.1	5307020	5319614	chr13	6607804	6629086
4547	CP026250.1	5319856	5338647	chr13	6594728	6607685
4548	CP026250.1	5339055	5348440	chr13	6287911	6594581
4549	CP026250.1	5348537	5349890	chr13	6257950	6287362

4550	CP026250.1	5350081	5371224	chr13	6231916	6257369
4551	CP026250.1	5372197	5438896	chr13	6184992	6231742
4552	CP026250.1	5439089	5442335	chr13	6157755	6184582
4553	CP026250.1	5442726	5654863	chr13	6124493	6156567
4554	CP026250.1	5655746	5678644	chr13	6109919	6124398
4555	CP026250.1	5679330	5682714	chr13	6051766	6109291
4556	CP026250.1	5683462	5686946	chr13	5917169	6051550
4557	CP026250.1	5689127	5731373	chr13	5860599	5917061
4558	CP026250.1	5737545	5761136	chr13	5839815	5859612
4559	CP026250.1	5761281	5989220	chr13	5835437	5839437
4560	CP026250.1	5989343	6023927	chr13	5825031	5834393
4561	CP026250.1	6024186	6033548	chr13	5790188	5824772
4562	CP026250.1	6034592	6038592	chr13	5562126	5790065
4563	CP026250.1	6038970	6058767	chr13	5538390	5561981
4564	CP026250.1	6059754	6116216	chr13	5489972	5532218
4565	CP026250.1	6116324	6250705	chr13	5484307	5487791
4566	CP026250.1	6250921	6308446	chr13	5480175	5483559
4567	CP026250.1	6309074	6323553	chr13	5456591	5479489
4568	CP026250.1	6323648	6355722	chr13	5243571	5455708
4569	CP026250.1	6356910	6383737	chr13	5239934	5243180
4570	CP026250.1	6384147	6430897	chr13	5173042	5239741
4571	CP026250.1	6431071	6456524	chr13	5150926	5172069
4572	CP026250.1	6457105	6486517	chr13	5149382	5150735
4573	CP026250.1	6487066	6793736	chr13	5139900	5149285
4574	CP026250.1	6793883	6806840	chr13	5120701	5139492
4575	CP026250.1	6806959	6828241	chr13	5107865	5120459
4576	CP026250.1	6828853	6922575	chr13	5096882	5107139
4577	CP026250.1	6922714	6930897	chr13	5079057	5096791
4578	CP026250.1	6931092	7008929	chr13	5062843	5078968

4579	CP026250.1	7009432	7012027	chr13	5058624	5062656
4580	CP026250.1	7012301	7018954	chr13	5023682	5058340
4581	CP026250.1	7020217	7073292	chr13	5014581	5023562
4582	CP026250.1	7073601	7095747	chr13	4999931	5014448
4583	CP026250.1	7095933	7097349	chr13	4974511	4999769
4584	CP026250.1	7097632	7099862	chr13	4970924	4974355
4585	CP026250.1	7100458	7130513	chr13	4965686	4970810
4586	CP026250.1	7130642	7220132	chr13	4957860	4965344
4587	CP026250.1	7220361	7233957	chr13	4951384	4957767
4588	CP026250.1	7234062	7287488	chr13	4949779	4950762
4589	CP026250.1	7287653	7300611	chr13	4949485	4949672
4590	CP026250.1	7300824	7305304	chr13	4943271	4949300
4591	CP026250.1	7307086	7394666	chr13	4809162	4939371
4592	CP026250.1	7394839	7430401	chr13	4804353	4809075
4593	CP026250.1	7430719	7510102	chr13	4799804	4804042
4594	CP026250.1	7510233	7548807	chr13	4788873	4799596
4595	CP026250.1	7548956	7596649	chr13	4779032	4788586
4596	CP026250.1	7597615	7635667	chr13	4745167	4778485
4597	CP026250.1	7635837	7846021	chr13	4726901	4743939
4598	CP026250.1	7846183	7854046	chr13	4645796	4725185
4599	CP026250.1	7854220	7996722	chr13	4641992	4643485
4600	CP026250.1	7997593	8210167	chr13	4636204	4640557
4601	CP026250.1	8211040	8229958	chr13	4627661	4635877
4602	CP026250.1	8230070	8272051	chr13	4610979	4627495
4603	CP026250.1	8272203	8473162	chr13	4600042	4610192
4604	CP026250.1	8473250	8474813	chr13	4594576	4598315
4605	CP026250.1	8475158	8608315	chr13	4592887	4594420
4606	CP026250.1	8608551	8636691	chr13	4589170	4591597
4607	CP026250.1	8637222	8682641	chr13	4529508	4589027

4608	CP026250.1	8693912	8713415	chr13	4525812	4528687
4609	CP026250.1	8713553	8749434	chr13	4497847	4525567
4610	CP026250.1	8749681	8782132	chr13	4470630	4497737
4611	CP026250.1	8782325	8790741	chr13	4435492	4470051
4612	CP026250.1	8791064	8799861	chr13	4405385	4433639
4613	CP026250.1	8800188	8961234	chr13	4388601	4405218
4614	CP026250.1	8961351	9107143	chr13	4387968	4388352
4615	CP026250.1	9107281	9113810	chr13	4384849	4387827
4616	CP026250.1	9114500	9160140	chr13	4365134	4383853
4617	CP026250.1	9160317	9232068	chr13	4353693	4365028
4618	CP026250.1	9232333	9241054	chr13	4326689	4353591
4619	CP026250.1	9241182	9243274	chr13	4289755	4326486
4620	CP026250.1	9243506	9270604	chr13	4281094	4286278
4621	CP026250.1	9271346	9311309	chr13	4235986	4279859
4622	CP026250.1	9311425	9330220	chr13	4231882	4235855
4623	CP026250.1	9330383	9339053	chr13	4228500	4231779
4624	CP026250.1	9339249	9354085	chr13	4214776	4227800
4625	CP026250.1	9354387	9412420	chr13	4189873	4214105
4626	CP026250.1	9413418	9482790	chr13	4161030	4189645
4627	CP026250.1	9482897	9784730	chr13	4094235	4160891
4628	CP026250.1	9784844	9828702	chr13	4044408	4093950
4629	CP026250.1	9828908	9896187	chr13	4021061	4043974
4630	CP026250.1	9896335	10063135	chr13	4013576	4020894
4631	CP026250.1	10064075	10078859	chr13	4005962	4013275
4632	CP026250.1	10079219	10125751	chr13	3978746	4005422
4633	CP026250.1	10125936	10136733	chr13	3974574	3978617
4634	CP026250.1	10136834	10360213	chr13	3968065	3974196
4635	CP026250.1	10360456	10441086	chr13	3965373	3967962
4636	CP026250.1	10441584	10466682	chr13	3950392	3963314

4637	CP026250.1	10467203	10521631	chr13	3935663	3950285
4638	CP026250.1	10522079	10604959	chr13	3929961	3935483
4639	CP026250.1	10605273	10610701	chr13	3917224	3928889
4640	CP026250.1	10611187	10877747	chr13	3913319	3916804
4641	CP026250.1	10878252	10880081	chr13	3909877	3912496
4642	CP026250.1	10880381	10941361	chr13	3900466	3909732
4643	CP026250.1	10941503	10943438	chr13	3898812	3900301
4644	CP026250.1	10943754	10977109	chr13	3890396	3898314
4645	CP026250.1	10977264	10998059	chr13	3881158	3889731
4646	CP026250.1	10998238	11029472	chr13	3859838	3879028
4647	CP026250.1	11029577	11030886	chr13	3854294	3859686
4648	CP026250.1	11031719	11078995	chr13	3851472	3854177
4649	CP026250.1	11079125	11098331	chr13	3845452	3851040
4650	CP026250.1	11098429	11119461	chr13	3843560	3845283
4651	CP026250.1	11119981	11143281	chr13	3839125	3841706
4652	CP026250.1	11143550	11165503	chr13	3822089	3839020
4653	CP026250.1	11165873	11189724	chr13	3794166	3821530
4654	CP026250.1	11189935	11288831	chr13	3747894	3793931
4655	CP026250.1	11289026	11375932	chr13	3736617	3747690
4656	CP026250.1	11376169	11480407	chr13	3687985	3735578
4657	CP026250.1	11480625	11494274	chr13	3673761	3687889
4658	CP026250.1	11494498	11571897	chr13	3654956	3672363
4659	CP026250.1	11572064	11658411	chr13	3628117	3654823
4660	CP026250.1	11658629	11680790	chr13	3620281	3624496
4661	CP026250.1	11681412	11722883	chr13	3610712	3619616
4662	CP026250.1	11726809	11762293	chr13	3592707	3610491
4663	CP026250.1	11762386	11837129	chr13	3582952	3592500
4664	CP026250.1	11837455	11867928	chr13	3555306	3578159
4665	CP026250.1	11868063	11884034	chr13	3546043	3554993

4666	CP026250.1	11884136	11889867	chr13	3543871	3545942
4667	CP026250.1	11889965	11927338	chr13	3542414	3543696
4668	CP026250.1	11927425	11993746	chr13	3539975	3541396
4669	CP026250.1	11993865	12006124	chr13	3535216	3539735
4670	CP026250.1	12006253	12018268	chr13	3529834	3534922
4671	CP026250.1	12018572	12041151	chr13	3526876	3529145
4672	CP026250.1	12041276	12061226	chr13	3518882	3524593
4673	CP026250.1	12061348	12268617	chr13	3491482	3518604
4674	CP026250.1	12268822	12434891	chr13	3490053	3491302
4675	CP026250.1	12435228	12504168	chr13	3431544	3489071
4676	CP026250.1	12504370	12517457	chr13	3387541	3431398
4677	CP026250.1	12517568	12604456	chr13	3383497	3386716
4678	CP026250.1	12605155	12625615	chr13	3380356	3383066
4679	CP026250.1	12625743	12736267	chr13	3378544	3380242
4680	CP026250.1	12736550	12899802	chr13	3365315	3376566
4681	CP026250.1	12900412	12991893	chr13	3356773	3364085
4682	CP026250.1	12993081	13035606	chr13	3347063	3355382
4683	CP026250.1	13035786	13145792	chr13	3338593	3346827
4684	CP026250.1	13145918	13169915	chr13	3326315	3337652
4685	CP026250.1	13170189	13210214	chr13	3324850	3326016
4686	CP026250.1	13210328	13226504	chr13	3321407	3324478
4687	CP026250.1	13226625	13287608	chr13	3279919	3321186
4688	CP026250.1	13287769	13346850	chr13	3277870	3279743
4689	CP026250.1	13346939	13349571	chr13	3276166	3277638
4690	CP026250.1	13351391	13366978	chr13	3263700	3276052
4691	CP026250.1	13367126	13448520	chr13	3247890	3263472
4692	CP026250.1	13448839	13470390	chr13	3226821	3246916
4693	CP026250.1	13470558	13562304	chr13	3205797	3226671
4694	CP026250.1	13563764	13655452	chr13	3197877	3205618

4695	CP026250.1	13655579	13701217	chr13	3183169	3197694
4696	CP026250.1	13701303	13725037	chr13	3158817	3183051
4697	CP026250.1	13725125	13889589	chr13	3144855	3157636
4698	CP026250.1	13890363	13942692	chr13	3076295	3143311
4699	CP026250.1	13942920	13945170	chr13	3014107	3076002
4700	CP026250.1	13945338	14063346	chr13	2990051	3013156
4701	CP026250.1	14063549	14073672	chr13	2980553	2989207
4702	CP026250.1	14074718	14198627	chr13	2866756	2979470
4703	CP026250.1	14199765	14247337	chr13	2854281	2865958
4704	CP026250.1	14247775	14277266	chr13	2844841	2850275
4705	CP026250.1	14277619	14460487	chr13	2842567	2844100
4706	CP026250.1	14460580	14507512	chr13	2840162	2841850
4707	CP026250.1	14507601	14516954	chr13	2838137	2839920
4708	CP026250.1	14517056	14562627	chr13	2827300	2837953
4709	CP026250.1	14562853	14589657	chr13	2818543	2825679
4710	CP026250.1	14590074	14652073	chr13	2808236	2816772
4711	CP026250.1	14652176	14743530	chr13	2804306	2807769
4712	CP026250.1	14743985	14813652	chr13	2795160	2803543
4713	CP026250.1	14813789	14814893	chr13	2787393	2795066
4714	CP026250.1	14815174	14845581	chr13	2763033	2786499
4715	CP026250.1	14845693	14978620	chr13	2753461	2762900
4716	CP026250.1	14978726	15044208	chr13	2721087	2753226
4717	CP026250.1	15044460	15050840	chr13	2696856	2719642
4718	CP026250.1	15051974	15140025	chr13	2694608	2696428
4719	CP026250.1	15140477	15206209	chr13	2691528	2693783
4720	CP026250.1	15206517	15334815	chr13	2669297	2690465
4721	CP026250.1	15334989	15415382	chr13	2663456	2668590
4722	CP026250.1	15415613	15447416	chr13	2661095	2662392
4723	CP026250.1	15447586	15450427	chr13	2641328	2660994

4724	CP026250.1	15450551	15485734	chr13	2637757	2639931
4725	CP026250.1	15485900	15730704	chr13	2631157	2633680
4726	CP026250.1	15730994	16050307	chr13	2626001	2630776
4727	CP026250.1	16051530	16082290	chr13	1439149	1440883
4728	CP026250.1	16084158	16187983	chr13	1436942	1438266
4729	CP026250.1	16188103	16189348	chr13	1417908	1436613
4730	CP026250.1	16189488	16206990	chr13	1414889	1417822
4731	CP026250.1	16207108	16224625	chr13	1382923	1413221
4732	CP026250.1	16225963	16233793	chr13	1347191	1382739
4733	CP026250.1	16234244	16246356	chr13	1324091	1347021
4734	CP026250.1	16246544	16361254	chr13	1315135	1322945
4735	CP026250.1	16361411	16372650	chr13	1292228	1315003
4736	CP026250.1	16373039	16450057	chr13	1272689	1291966
4737	CP026250.1	16450167	16490869	chr13	1251140	1272530
4738	CP026250.1	16491551	16535005	chr13	1247755	1250298
4739	CP026250.1	16536298	16555282	chr13	1244634	1246577
4740	CP026250.1	16555383	16625020	chr13	1210108	1244368
4741	CP026250.1	16625180	16645378	chr13	1195588	1208825
4742	CP026250.1	16645993	16733568	chr13	1177847	1195216
4743	CP026250.1	16733684	16742761	chr13	1174889	1176275
4744	CP026250.1	16745681	16788362	chr13	1171623	1174775
4745	CP026250.1	16788620	16799041	chr13	1131616	1169017
4746	CP026250.1	16799144	16829122	chr13	1129483	1131525
4747	CP026250.1	16830239	16850492	chr13	1122306	1129131
4748	CP026250.1	16851806	16887066	chr13	1118518	1121574
4749	CP026250.1	16887542	16992042	chr13	1104450	1118144
4750	CP026250.1	16992128	17017593	chr13	1097510	1103905
4751	CP026250.1	17018908	17095049	chr13	1086576	1094662
4752	CP026250.1	17095189	17131497	chr13	1073137	1085454

4753	CP026250.1	17131791	17147746	chr13	1068908	1072820
4754	CP026250.1	17147890	17216179	chr13	1057567	1067870
4755	CP026250.1	17217465	17287521	chr13	1020933	1056973
4756	CP026250.1	17287877	17312083	chr13	1013990	1020649
4757	CP026250.1	17312819	17363627	chr13	1012764	1013859
4758	CP026250.1	17363778	17410006	chr13	1011403	1012588
4759	CP026250.1	17410100	17532537	chr13	1004061	1010675
4760	CP026250.1	17535157	17543056	chr13	999548	1001969
4761	CP026250.1	17543171	17549291	chr13	997529	999020
4762	CP026250.1	17549656	17573839	chr13	995156	996724
4763	CP026250.1	17574852	17577452	chr13	994208	994998
4764	CP026250.1	17577742	17588231	chr13	992502	994030
4765	CP026250.1	17589109	17598651	chr13	990653	992400
4766	CP026250.1	17599425	17607620	chr13	963527	990147
4767	CP026250.1	17614388	17617823	chr13	941401	963148
4768	CP026250.1	17618605	17630706	chr13	938068	940246
4769	CP026250.1	17630826	17631162	chr13	935823	937212
4770	CP026250.1	17631380	17636115	chr13	905935	934223
4771	CP026250.1	17640693	17664630	chr13	899688	905834
4772	CP026250.1	17664749	17671930	chr13	895869	899575
4773	CP026250.1	17672273	17698275	chr13	887296	894895
4774	CP026250.1	17700560	17720546	chr13	877522	886567
4775	CP026250.1	17720862	17728532	chr13	868581	877180
4776	CP026250.1	17728648	17730126	chr13	859469	868397
4777	CP026250.1	17731597	17733851	chr13	852492	857160
4778	CP026250.1	17734950	17737239	chr13	838920	851668
4779	CP026250.1	17737844	17740953	chr13	823911	835409
4780	CP026250.1	17741131	17744266	chr13	2093	2367
4781	CP026250.1	17746117	17748160	chr13	949	1223

4782	CP026262.1	19809792	19811222	chr14	1431	1
4783	CP026262.1	19801871	19807927	chr14	9352	3296
4784	CP026262.1	19795635	19801291	chr14	15588	9932
4785	CP026262.1	19791677	19795503	chr14	19546	15720
4786	CP026262.1	19788574	19791073	chr14	22649	20150
4787	CP026262.1	19776316	19785235	chr14	34907	25988
4788	CP026262.1	19763567	19775683	chr14	47656	35540
4789	CP026262.1	19759136	19763279	chr14	52087	47944
4790	CP026262.1	19754552	19758179	chr14	56671	53044
4791	CP026262.1	19751767	19753914	chr14	59456	57309
4792	CP026262.1	19748714	19751197	chr14	62509	60026
4793	CP026262.1	19741305	19748612	chr14	69918	62611
4794	CP026262.1	19735665	19739677	chr14	75558	71546
4795	CP026262.1	19732100	19734053	chr14	79123	77170
4796	CP026262.1	19727165	19730694	chr14	84058	80529
4797	CP026262.1	19724019	19726210	chr14	87204	85013
4798	CP026262.1	19708073	19723716	chr14	103150	87507
4799	CP026262.1	19689642	19707885	chr14	121581	103338
4800	CP026262.1	19685881	19688426	chr14	125342	122797
4801	CP026262.1	19683786	19685178	chr14	127437	126045
4802	CP026262.1	19676279	19683482	chr14	134944	127741
4803	CP026262.1	19670535	19674217	chr14	140688	137006
4804	CP026262.1	19666527	19668689	chr14	144696	142534
4805	CP026262.1	19641536	19665333	chr14	169687	145890
4806	CP026262.1	19639031	19641174	chr14	172192	170049
4807	CP026262.1	19637004	19637941	chr14	174219	173282
4808	CP026262.1	19624950	19636546	chr14	186273	174677
4809	CP026262.1	19620691	19622678	chr14	190532	188545
4810	CP026262.1	19615803	19620550	chr14	195420	190673

4811	CP026262.1	19610681	19614150	chr14	200542	197073
4812	CP026262.1	19602109	19610527	chr14	209114	200696
4813	CP026262.1	19598756	19601120	chr14	212467	210103
4814	CP026262.1	19594465	19597073	chr14	216758	214150
4815	CP026262.1	19592873	19593679	chr14	218350	217544
4816	CP026262.1	19592522	19592749	chr14	218701	218474
4817	CP026262.1	19580473	19590528	chr14	230750	220695
4818	CP026262.1	19571740	19578044	chr14	237155	230851
4819	CP026262.1	19566563	19570541	chr14	242332	238354
4820	CP026262.1	19559200	19566220	chr14	249695	242675
4821	CP026262.1	19555715	19558914	chr14	253180	249981
4822	CP026262.1	19547532	19554691	chr14	261363	254204
4823	CP026262.1	19536667	19546762	chr14	272228	262133
4824	CP026262.1	19524736	19535554	chr14	284159	273341
4825	CP026262.1	19511431	19522339	chr14	297464	286556
4826	CP026262.1	19507327	19510601	chr14	301568	298294
4827	CP026262.1	19503080	19506530	chr14	305815	302365
4828	CP026262.1	19485715	19492291	chr14	323180	316604
4829	CP026262.1	19462071	19484605	chr14	346824	324290
4830	CP026262.1	19455569	19461790	chr14	353326	347105
4831	CP026262.1	19452159	19455162	chr14	356736	353733
4832	CP026262.1	19442779	19451232	chr14	366116	357663
4833	CP026262.1	19401873	19440432	chr14	407022	368463
4834	CP026262.1	19395313	19401125	chr14	413582	407770
4835	CP026262.1	19377910	19394602	chr14	430985	414293
4836	CP026262.1	19372495	19376910	chr14	436400	431985
4837	CP026262.1	19349286	19371528	chr14	459609	437367
4838	CP026262.1	19342504	19348695	chr14	466391	460200
4839	CP026262.1	19338626	19341558	chr14	470269	467337

4840	CP026262.1	19328952	19338464	chr14	479943	470431
4841	CP026262.1	19306947	19328638	chr14	501948	480257
4842	CP026262.1	19299793	19305624	chr14	509102	503271
4843	CP026262.1	19291336	19299393	chr14	517559	509502
4844	CP026262.1	19288754	19291164	chr14	520141	517731
4845	CP026262.1	19280355	19288432	chr14	528540	520463
4846	CP026262.1	19277753	19279966	chr14	531142	528929
4847	CP026262.1	19273696	19277572	chr14	535199	531323
4848	CP026262.1	19259037	19273335	chr14	549858	535560
4849	CP026262.1	19237821	19258767	chr14	571074	550128
4850	CP026262.1	19230746	19237662	chr14	578149	571233
4851	CP026262.1	19217497	19229818	chr14	591398	579077
4852	CP026262.1	19172049	19217376	chr14	636846	591519
4853	CP026262.1	19163560	19171922	chr14	645335	636973
4854	CP026262.1	19155622	19163175	chr14	653273	645720
4855	CP026262.1	19148636	19155363	chr14	660259	653532
4856	CP026262.1	19142925	19147789	chr14	665970	661106
4857	CP026262.1	19133834	19141984	chr14	675061	666911
4858	CP026262.1	19124064	19133650	chr14	684831	675245
4859	CP026262.1	19113896	19123469	chr14	694999	685426
4860	CP026262.1	19107186	19113729	chr14	701709	695166
4861	CP026262.1	19093901	19107086	chr14	714994	701809
4862	CP026262.1	19082565	19092269	chr14	726330	716626
4863	CP026262.1	19023250	19082023	chr14	785645	726872
4864	CP026262.1	19003627	19021203	chr14	805268	787692
4865	CP026262.1	19000891	19002461	chr14	808004	806434
4866	CP026262.1	18996875	18998977	chr14	812020	809918
4867	CP026262.1	18995343	18996721	chr14	813552	812174
4868	CP026262.1	18991305	18995142	chr14	817590	813753

4869	CP026262.1	18978421	18991084	chr14	830474	817811
4870	CP026262.1	18944884	18977960	chr14	864011	830935
4871	CP026262.1	18938313	18943247	chr14	870582	865648
4872	CP026262.1	18930654	18937880	chr14	878241	871015
4873	CP026262.1	18926462	18929823	chr14	882433	879072
4874	CP026262.1	18919162	18925817	chr14	889733	883078
4875	CP026262.1	18879089	18918875	chr14	929806	890020
4876	CP026262.1	18871961	18878108	chr14	936934	930787
4877	CP026262.1	18818279	18871686	chr14	990616	937209
4878	CP026262.1	18807118	18818150	chr14	1001777	990745
4879	CP026262.1	18799960	18806652	chr14	1008935	1002243
4880	CP026262.1	18793872	18796285	chr14	1015023	1012610
4881	CP026262.1	18788627	18793381	chr14	1020268	1015514
4882	CP026262.1	18782658	18788511	chr14	1026237	1020384
4883	CP026262.1	18763889	18782169	chr14	1045006	1026726
4884	CP026262.1	18749672	18763653	chr14	1059223	1045242
4885	CP026262.1	18746199	18749575	chr14	1062696	1059320
4886	CP026262.1	18741785	18746078	chr14	1067110	1062817
4887	CP026262.1	18732315	18737985	chr14	1076580	1070910
4888	CP026262.1	18717209	18731893	chr14	1091686	1077002
4889	CP026262.1	18712392	18717020	chr14	1096503	1091875
4890	CP026262.1	18706628	18712297	chr14	1102267	1096598
4891	CP026262.1	18703394	18705680	chr14	1105501	1103215
4892	CP026262.1	18699952	18703059	chr14	1108943	1105836
4893	CP026262.1	18695779	18699863	chr14	1113116	1109032
4894	CP026262.1	18682295	18691452	chr14	1126600	1117443
4895	CP026262.1	18676503	18681996	chr14	1132392	1126899
4896	CP026262.1	18673097	18674938	chr14	1135798	1133957
4897	CP026262.1	18668554	18671759	chr14	1140341	1137136

4898	CP026262.1	18646132	18666974	chr14	1162763	1141921
4899	CP026262.1	18635605	18645666	chr14	1173290	1163229
4900	CP026262.1	18630961	18631954	chr14	1177934	1176941
4901	CP026262.1	18615864	18630842	chr14	1193031	1178053
4902	CP026262.1	18605437	18614989	chr14	1203458	1193906
4903	CP026262.1	18602291	18605324	chr14	1206604	1203571
4904	CP026262.1	18593499	18601961	chr14	1215396	1206934
4905	CP026262.1	18566104	18592667	chr14	1242791	1216228
4906	CP026262.1	18558346	18565294	chr14	1250549	1243601
4907	CP026262.1	18485396	18558151	chr14	1323499	1250744
4908	CP026262.1	18483773	18484995	chr14	1325122	1323900
4909	CP026262.1	18477958	18483649	chr14	1330937	1325246
4910	CP026262.1	18460871	18477421	chr14	1348024	1331474
4911	CP026262.1	18460256	18460763	chr14	1348639	1348132
4912	CP026262.1	18452411	18459367	chr14	1356484	1349528
4913	CP026262.1	18445862	18451687	chr14	1363033	1357208
4914	CP026262.1	18439385	18443563	chr14	1369510	1365332
4915	CP026262.1	18433796	18438842	chr14	1375099	1370053
4916	CP026262.1	18428963	18431726	chr14	1379932	1377169
4917	CP026262.1	18423431	18428815	chr14	1385464	1380080
4918	CP026262.1	18409448	18423179	chr14	1399447	1385716
4919	CP026262.1	18407712	18409123	chr14	1401183	1399772
4920	CP026262.1	18385406	18407199	chr14	1423489	1401696
4921	CP026262.1	18374803	18384732	chr14	1434092	1424163
4922	CP026262.1	18369898	18374643	chr14	1438997	1434252
4923	CP026262.1	18365467	18368310	chr14	1443428	1440585
4924	CP026262.1	18358899	18365150	chr14	1449996	1443745
4925	CP026262.1	18325150	18358467	chr14	1483745	1450428
4926	CP026262.1	18320794	18325009	chr14	1488101	1483886

4927	CP026262.1	18275525	18320488	chr14	1533370	1488407
4928	CP026262.1	18248517	18275321	chr14	1560378	1533574
4929	CP026262.1	18245636	18247742	chr14	1563259	1561153
4930	CP026262.1	18237050	18245401	chr14	1571845	1563494
4931	CP026262.1	18156143	18236114	chr14	1652752	1572781
4932	CP026262.1	18141790	18155888	chr14	1667105	1653007
4933	CP026262.1	18135878	18141667	chr14	1673017	1667228
4934	CP026262.1	18125573	18135210	chr14	1683322	1673685
4935	CP026262.1	18105097	18125084	chr14	1703798	1683811
4936	CP026262.1	18069512	18104962	chr14	1739383	1703933
4937	CP026262.1	18042078	18069377	chr14	1766817	1739518
4938	CP026262.1	18028120	18041910	chr14	1780775	1766985
4939	CP026262.1	17902952	18027927	chr14	1905943	1780968
4940	CP026262.1	17898923	17902866	chr14	1909972	1906029
4941	CP026262.1	17839699	17898098	chr14	1969196	1910797
4942	CP026262.1	17817786	17839022	chr14	1991109	1969873
4943	CP026262.1	17811736	17813605	chr14	1997159	1995290
4944	CP026262.1	17805719	17807641	chr14	2003176	2001254
4945	CP026262.1	17783179	17805592	chr14	2025716	2003303
4946	CP026262.1	17748413	17782954	chr14	2060482	2025941
4947	CP026262.1	17695781	17746793	chr14	2113114	2062102
4948	CP026262.1	17687029	17695573	chr14	2121866	2113322
4949	CP026262.1	17683483	17686383	chr14	2125412	2122512
4950	CP026262.1	17647126	17682852	chr14	2161769	2126043
4951	CP026262.1	17643450	17645803	chr14	2165445	2163092
4952	CP026262.1	17606765	17643245	chr14	2202130	2165650
4953	CP026262.1	17573586	17606546	chr14	2235309	2202349
4954	CP026262.1	17566903	17572134	chr14	2241992	2236761
4955	CP026262.1	17545707	17566679	chr14	2263188	2242216

4956	CP026262.1	17542096	17545550	chr14	2266799	2263345
4957	CP026262.1	17512949	17541974	chr14	2295946	2266921
4958	CP026262.1	17497981	17511688	chr14	2310914	2297207
4959	CP026262.1	17495117	17497869	chr14	2313778	2311026
4960	CP026262.1	17480926	17494690	chr14	2327969	2314205
4961	CP026262.1	17467345	17480290	chr14	2341550	2328605
4962	CP026262.1	17460377	17467135	chr14	2348518	2341760
4963	CP026262.1	17420691	17460289	chr14	2388204	2348606
4964	CP026262.1	17418779	17420488	chr14	2390116	2388407
4965	CP026262.1	17404812	17418284	chr14	2404083	2390611
4966	CP026262.1	17403198	17404717	chr14	2405697	2404178
4967	CP026262.1	17391384	17403097	chr14	2417511	2405798
4968	CP026262.1	17382298	17391154	chr14	2426597	2417741
4969	CP026262.1	17367961	17381985	chr14	2440934	2426910
4970	CP026262.1	17354471	17365425	chr14	2454424	2443470
4971	CP026262.1	17343088	17354137	chr14	2465807	2454758
4972	CP026262.1	17293180	17342863	chr14	2515715	2466032
4973	CP026262.1	17280137	17293091	chr14	2528758	2515804
4974	CP026262.1	17272474	17279642	chr14	2536421	2529253
4975	CP026262.1	17264660	17271266	chr14	2544235	2537629
4976	CP026262.1	17245838	17264354	chr14	2563057	2544541
4977	CP026262.1	17234166	17245499	chr14	2574729	2563396
4978	CP026262.1	17213356	17233938	chr14	2595539	2574957
4979	CP026262.1	17210625	17211744	chr14	2598270	2597151
4980	CP026262.1	17206415	17210307	chr14	2602480	2598588
4981	CP026262.1	17153768	17205646	chr14	2655127	2603249
4982	CP026262.1	17119682	17150055	chr14	2689213	2658840
4983	CP026262.1	17115061	17119511	chr14	2693834	2689384
4984	CP026262.1	17073825	17114036	chr14	2735070	2694859

4985	CP026262.1	17003626	17070546	chr14	2805269	2738349
4986	CP026262.1	16998825	17002262	chr14	2810070	2806633
4987	CP026262.1	16977661	16998579	chr14	2831234	2810316
4988	CP026262.1	16974275	16977458	chr14	2834620	2831437
4989	CP026262.1	16970685	16974169	chr14	2838210	2834726
4990	CP026262.1	16928545	16969947	chr14	2880350	2838948
4991	CP026262.1	16926667	16927989	chr14	2882228	2880906
4992	CP026262.1	16895209	16926540	chr14	2913686	2882355
4993	CP026262.1	16847356	16893497	chr14	2961539	2915398
4994	CP026262.1	16804257	16847231	chr14	3004638	2961664
4995	CP026262.1	16780277	16803573	chr14	3028618	3005322
4996	CP026262.1	16763513	16780001	chr14	3045382	3028894
4997	CP026262.1	16728248	16763302	chr14	3080647	3045593
4998	CP026262.1	16702755	16722845	chr14	3106140	3086050
4999	CP026262.1	16690865	16702559	chr14	3118030	3106336
5000	CP026262.1	16669134	16690716	chr14	3139761	3118179
5001	CP026262.1	16657444	16668920	chr14	3151451	3139975
5002	CP026262.1	16645307	16657098	chr14	3163588	3151797
5003	CP026262.1	16629009	16645099	chr14	3179886	3163796
5004	CP026262.1	16623115	16628523	chr14	3185780	3180372
5005	CP026262.1	16613932	16622621	chr14	3194963	3186274
5006	CP026262.1	16612500	16613673	chr14	3196395	3195222
5007	CP026262.1	16611309	16612392	chr14	3197586	3196503
5008	CP026262.1	16595492	16611046	chr14	3213403	3197849
5009	CP026262.1	16590466	16595345	chr14	3218429	3213550
5010	CP026262.1	16587761	16589991	chr14	3221134	3218904
5011	CP026262.1	16581844	16586760	chr14	3227051	3222135
5012	CP026262.1	16571948	16580052	chr14	3236947	3228843
5013	CP026262.1	16565551	16571312	chr14	3243344	3237583

5014	CP026262.1	16563451	16564731	chr14	3245444	3244164
5015	CP026262.1	16553008	16558915	chr14	3255887	3249980
5016	CP026262.1	16523109	16552021	chr14	3285786	3256874
5017	CP026262.1	16494515	16522931	chr14	3314380	3285964
5018	CP026262.1	16492670	16494001	chr14	3316225	3314894
5019	CP026262.1	16481698	16492390	chr14	3327197	3316505
5020	CP026262.1	16337783	16480754	chr14	3471112	3328141
5021	CP026262.1	16326247	16336580	chr14	3482648	3472315
5022	CP026262.1	16314015	16325990	chr14	3494880	3482905
5023	CP026262.1	16274680	16313831	chr14	3534215	3495064
5024	CP026262.1	16265916	16274433	chr14	3542979	3534462
5025	CP026262.1	16193069	16265729	chr14	3615826	3543166
5026	CP026262.1	16122070	16192917	chr14	3686825	3615978
5027	CP026262.1	16112327	16121857	chr14	3696568	3687038
5028	CP026262.1	16083182	16110435	chr14	3725713	3698460
5029	CP026262.1	16075160	16082905	chr14	3733735	3725990
5030	CP026262.1	16065395	16074925	chr14	3743500	3733970
5031	CP026262.1	16062257	16063764	chr14	3746638	3745131
5032	CP026262.1	16058185	16060774	chr14	3750710	3748121
5033	CP026262.1	16052256	16057736	chr14	3756639	3751159
5034	CP026262.1	16006003	16051316	chr14	3802892	3757579
5035	CP026262.1	16003682	16005156	chr14	3805213	3803739
5036	CP026262.1	15964610	16003313	chr14	3844285	3805582
5037	CP026262.1	15956198	15964153	chr14	3852697	3844742
5038	CP026262.1	15928517	15955989	chr14	3880378	3852906
5039	CP026262.1	15922250	15927299	chr14	3886645	3881596
5040	CP026262.1	15908522	15922019	chr14	3900373	3886876
5041	CP026262.1	15900760	15908407	chr14	3908135	3900488
5042	CP026262.1	15869497	15900118	chr14	3939398	3908777

5043	CP026262.1	15851590	15868385	chr14	3957305	3940510
5044	CP026262.1	15849149	15851283	chr14	3959746	3957612
5045	CP026262.1	15844783	15847844	chr14	3964112	3961051
5046	CP026262.1	15836703	15844543	chr14	3972192	3964352
5047	CP026262.1	15830206	15835495	chr14	3978689	3973400
5048	CP026262.1	15798965	15828110	chr14	4009930	3980785
5049	CP026262.1	15774774	15798744	chr14	4034121	4010151
5050	CP026262.1	15770940	15774234	chr14	4037955	4034661
5051	CP026262.1	15726622	15770217	chr14	4082273	4038678
5052	CP026262.1	15714265	15726437	chr14	4094630	4082458
5053	CP026262.1	15709341	15713496	chr14	4099554	4095399
5054	CP026262.1	15688950	15708793	chr14	4119945	4100102
5055	CP026262.1	15686039	15688723	chr14	4122856	4120172
5056	CP026262.1	15569823	15684353	chr14	4239072	4124542
5057	CP026262.1	15493204	15569571	chr14	4315691	4239324
5058	CP026262.1	15482959	15491423	chr14	4325936	4317472
5059	CP026262.1	15331990	15482854	chr14	4476905	4326041
5060	CP026262.1	15320296	15331813	chr14	4488599	4477082
5061	CP026262.1	15313249	15318779	chr14	4495646	4490116
5062	CP026262.1	15304646	15313104	chr14	4504249	4495791
5063	CP026262.1	15288617	15303535	chr14	4520278	4505360
5064	CP026262.1	15260932	15288412	chr14	4547963	4520483
5065	CP026262.1	15259336	15260804	chr14	4549559	4548091
5066	CP026262.1	15255024	15258646	chr14	4553871	4550249
5067	CP026262.1	15194827	15254638	chr14	4614068	4554257
5068	CP026262.1	15117936	15193352	chr14	4690959	4615543
5069	CP026262.1	15053386	15117306	chr14	4755509	4691589
5070	CP026262.1	15047720	15052370	chr14	4761175	4756525
5071	CP026262.1	14984375	15047578	chr14	4824520	4761317

5072	CP026262.1	14964441	14984278	chr14	4844454	4824617
5073	CP026262.1	14684895	14964122	chr14	5124000	4844773
5074	CP026262.1	14462615	14684597	chr14	5346280	5124298
5075	CP026262.1	10805005	10821604	chr14	9002986	8986387
5076	CP026262.1	10799308	10804909	chr14	9008683	9003082
5077	CP026262.1	10608654	10799146	chr14	9199337	9008845
5078	CP026262.1	10583745	10608162	chr14	9224246	9199829
5079	CP026262.1	10424173	10529527	chr14	9383818	9278464
5080	CP026262.1	10408802	10423905	chr14	9399189	9384086
5081	CP026262.1	10385241	10408689	chr14	9422750	9399302
5082	CP026262.1	10357449	10385152	chr14	9450542	9422839
5083	CP026262.1	10336712	10357334	chr14	9471279	9450657
5084	CP026262.1	10003855	10336522	chr14	9804136	9471469
5085	CP026262.1	9998095	10003450	chr14	9809896	9804541
5086	CP026262.1	9962316	9997862	chr14	9845675	9810129
5087	CP026262.1	9951709	9962103	chr14	9856282	9845888
5088	CP026262.1	9943962	9951547	chr14	9864029	9856444
5089	CP026262.1	9877420	9943669	chr14	9930571	9864322
5090	CP026262.1	9815057	9877059	chr14	9992934	9930932
5091	CP026262.1	9738082	9814964	chr14	10069909	9993027
5092	CP026262.1	9705666	9737878	chr14	10102325	10070113
5093	CP026262.1	9519833	9705542	chr14	10288158	10102449
5094	CP026262.1	9162792	9519664	chr14	10645199	10288327
5095	CP026262.1	9051510	9162692	chr14	10756481	10645299
5096	CP026262.1	8976805	9051404	chr14	10831186	10756587
5097	CP026262.1	8894187	8975999	chr14	10913804	10831992
5098	CP026262.1	8802672	8894093	chr14	11005319	10913898
5099	CP026262.1	8790176	8801798	chr14	11017815	11006193
5100	CP026262.1	8680441	8790079	chr14	11127550	11017912

5101	CP026262.1	8643729	8676954	chr14	11164262	11131037
5102	CP026262.1	8366651	8643525	chr14	11441340	11164466
5103	CP026262.1	8350590	8365975	chr14	11457401	11442016
5104	CP026262.1	8231057	8350108	chr14	11576934	11457883
5105	CP026262.1	8167738	8229701	chr14	11640253	11578290
5106	CP026262.1	8149329	8167515	chr14	11658662	11640476
5107	CP026262.1	8108687	8149148	chr14	11699304	11658843
5108	CP026262.1	8088627	8106155	chr14	11719364	11701836
5109	CP026262.1	8027101	8088445	chr14	11780890	11719546
5110	CP026262.1	7941258	8027004	chr14	11866733	11780987
5111	CP026262.1	7938654	7940716	chr14	11869337	11867275
5112	CP026262.1	7491742	7938526	chr14	12316249	11869465
5113	CP026262.1	7432520	7491349	chr14	12375471	12316642
5114	CP026262.1	7415165	7432090	chr14	12392826	12375901
5115	CP026262.1	7378141	7414960	chr14	12429850	12393031
5116	CP026262.1	7324663	7378040	chr14	12483328	12429951
5117	CP026262.1	7305725	7324439	chr14	12502266	12483552
5118	CP026262.1	7177016	7305340	chr14	12630975	12502651
5119	CP026262.1	6985261	7176904	chr14	12822730	12631087
5120	CP026262.1	6927123	6985159	chr14	12880868	12822832
5121	CP026262.1	6911959	6927001	chr14	12896032	12880990
5122	CP026262.1	6898756	6911833	chr14	12909235	12896158
5123	CP026262.1	6857707	6898545	chr14	12950284	12909446
5124	CP026262.1	6763833	6857585	chr14	13044158	12950406
5125	CP026262.1	6699540	6762755	chr14	13108451	13045236
5126	CP026262.1	6664441	6699348	chr14	13143550	13108643
5127	CP026262.1	6638793	6664282	chr14	13169198	13143709
5128	CP026262.1	6548470	6638675	chr14	13259521	13169316
5129	CP026262.1	6538949	6547933	chr14	13269042	13260058

5130	CP026262.1	6432823	6538861	chr14	13375168	13269130
5131	CP026262.1	6383898	6432579	chr14	13424093	13375412
5132	CP026262.1	6275232	6383800	chr14	13532759	13424191
5133	CP026262.1	6100733	6275134	chr14	13707258	13532857
5134	CP026262.1	6099441	6100643	chr14	13708550	13707348
5135	CP026262.1	6091526	6099294	chr14	13716465	13708697
5136	CP026262.1	5976611	6091405	chr14	13831380	13716586
5137	CP026262.1	5918460	5976022	chr14	13889531	13831969
5138	CP026262.1	5654790	5918239	chr14	14153201	13889752
5139	CP026262.1	5651429	5654081	chr14	14156562	14153910
5140	CP026262.1	5609282	5651079	chr14	14198709	14156912
5141	CP026262.1	5606959	5609145	chr14	14201032	14198846
5142	CP026262.1	5591127	5606790	chr14	14216864	14201201
5143	CP026262.1	5560843	5590801	chr14	14247148	14217190
5144	CP026262.1	5556000	5559457	chr14	14251991	14248534
5145	CP026262.1	5449046	5555541	chr14	14358945	14252450
5146	CP026262.1	5353387	5448878	chr14	14454604	14359113
5147	CP026262.1	5296510	5353185	chr14	14511481	14454806
5148	CP026262.1	5160215	5296422	chr14	14647776	14511569
5149	CP026262.1	5158556	5160102	chr14	14649435	14647889
5150	CP026262.1	5101370	5158230	chr14	14706621	14649761
5151	CP026262.1	5062589	5101223	chr14	14745402	14706768
5152	CP026262.1	4977909	5062463	chr14	14830082	14745528
5153	CP026262.1	4952735	4977681	chr14	14855256	14830310
5154	CP026262.1	4938845	4952626	chr14	14869146	14855365
5155	CP026262.1	4917709	4938709	chr14	14890282	14869282
5156	CP026262.1	4869215	4917543	chr14	14938776	14890448
5157	CP026262.1	4854052	4869074	chr14	14953939	14938917
5158	CP026262.1	4816499	4853951	chr14	14991492	14954040

5159	CP026262.1	4808305	4816385	chr14	14999686	14991606
5160	CP026262.1	4739192	4807758	chr14	15068799	15000233
5161	CP026262.1	4719542	4738869	chr14	15088449	15069122
5162	CP026262.1	4698806	4719413	chr14	15109185	15088578
5163	CP026262.1	4671801	4698666	chr14	15136190	15109325
5164	CP026262.1	4664358	4671677	chr14	15143633	15136314
5165	CP026262.1	4640777	4664249	chr14	15167214	15143742
5166	CP026262.1	4526572	4640662	chr14	15281419	15167329
5167	CP026262.1	4462803	4526440	chr14	15345188	15281551
5168	CP026262.1	4460953	4462496	chr14	15347038	15345495
5169	CP026262.1	4419528	4459480	chr14	15388463	15348511
5170	CP026262.1	4366126	4419318	chr14	15441865	15388673
5171	CP026262.1	4346806	4366015	chr14	15461185	15441976
5172	CP026262.1	4327763	4346699	chr14	15480228	15461292
5173	CP026262.1	4307244	4327597	chr14	15500747	15480394
5174	CP026262.1	4295091	4307119	chr14	15512900	15500872
5175	CP026262.1	4153616	4294445	chr14	15654375	15513546
5176	CP026262.1	4083166	4152740	chr14	15724825	15655251
5177	CP026262.1	4068607	4080379	chr14	15739384	15727612
5178	CP026262.1	1526625	1526796	chr14	18272668	18272503
5179	CP026262.1	1522800	1522965	chr14	18276499	18276328
5180	CP026255.1	6050679	6084402	chr15	16011059	16301534
5181	CP026255.1	6084525	6221682	chr15	15948578	16010768
5182	CP026255.1	6222284	6338555	chr15	15925411	15948490
5183	CP026255.1	6339615	6387148	chr15	15907296	15925158
5184	CP026255.1	6387634	6428751	chr15	15893383	15906854
5185	CP026255.1	6429054	6526855	chr15	15716083	15893271
5186	CP026255.1	6526944	6569718	chr15	15608165	15715430
5187	CP026255.1	6570276	6572010	chr15	15597149	15608062

5188	CP026255.1	6573648	6722272	chr15	15515311	15596649
5189	CP026255.1	6722442	6786044	chr15	15358928	15515185
5190	CP026255.1	6786148	6933005	chr15	15283099	15358730
5191	CP026255.1	6933288	7034440	chr15	15235728	15282734
5192	CP026255.1	7034569	7037228	chr15	15077152	15235587
5193	CP026255.1	7037559	7270277	chr15	15025025	15077016
5194	CP026255.1	7270370	7384552	chr15	14825890	15024226
5195	CP026255.1	7384650	7406496	chr15	14800986	14825605
5196	CP026255.1	7406590	7531830	chr15	14550839	14800544
5197	CP026255.1	7531931	7538596	chr15	14455433	14550725
5198	CP026255.1	7538783	7859751	chr15	14431630	14454935
5199	CP026255.1	7859863	8073118	chr15	14425703	14431538
5200	CP026255.1	8073255	8087970	chr15	14377232	14425174
5201	CP026255.1	8088196	8210729	chr15	14249127	14377125
5202	CP026255.1	8211027	8218837	chr15	14245081	14248896
5203	CP026255.1	8219162	8240640	chr15	14240046	14244929
5204	CP026255.1	8240966	8788045	chr15	14222285	14239112
5205	CP026255.1	8788973	8825778	chr15	14215931	14222191
5206	CP026255.1	8827469	8829585	chr15	14175420	14215698
5207	CP026255.1	8829888	9082815	chr15	14148234	14175101
5208	CP026255.1	9083848	9104953	chr15	14127001	14148106
5209	CP026255.1	9105081	9131948	chr15	13873041	14125968
5210	CP026255.1	9132267	9172545	chr15	13870622	13872738
5211	CP026255.1	9172778	9179038	chr15	13832126	13868931
5212	CP026255.1	9179132	9195959	chr15	13284119	13831198
5213	CP026255.1	9196893	9201776	chr15	13262315	13283793
5214	CP026255.1	9201928	9205743	chr15	13254180	13261990
5215	CP026255.1	9205974	9333972	chr15	13131349	13253882
5216	CP026255.1	9334079	9382021	chr15	13116408	13131123

5217	CP026255.1	9382550	9388385	chr15	12903016	13116271
5218	CP026255.1	9388477	9411782	chr15	12581936	12902904
5219	CP026255.1	9412280	9507572	chr15	12575084	12581749
5220	CP026255.1	9507686	9757391	chr15	12449743	12574983
5221	CP026255.1	9757833	9782452	chr15	12427803	12449649
5222	CP026255.1	9782737	9981073	chr15	12313523	12427705
5223	CP026255.1	9981872	10033863	chr15	12080712	12313430
5224	CP026255.1	10033999	10192434	chr15	12077722	12080381
5225	CP026255.1	10192575	10239581	chr15	11976441	12077593
5226	CP026255.1	10239946	10315577	chr15	11829301	11976158
5227	CP026255.1	10315775	10472032	chr15	11765595	11829197
5228	CP026255.1	10472158	10553496	chr15	11616801	11765425
5229	CP026255.1	10553996	10564909	chr15	11613429	11615163
5230	CP026255.1	10565012	10672277	chr15	11570097	11612871
5231	CP026255.1	10672930	10850118	chr15	11472207	11570008
5232	CP026255.1	10850230	10863701	chr15	11430787	11471904
5233	CP026255.1	10864143	10882005	chr15	11382768	11430301
5234	CP026255.1	10882258	10905337	chr15	11265437	11381708
5235	CP026255.1	10905425	10967615	chr15	11127678	11264835
5236	CP026255.1	10967906	11258381	chr15	11093832	11127555
5237	CP026252.1	21564634	21588220	chr16	3577070	3553484
5238	CP026252.1	21522662	21564478	chr16	3619042	3577226
5239	CP026252.1	21494426	21521245	chr16	3647278	3620459
5240	CP026252.1	21472230	21494186	chr16	3669474	3647518
5241	CP026252.1	21463751	21472063	chr16	3677953	3669641
5242	CP026252.1	21457027	21463577	chr16	3684677	3678127
5243	CP026252.1	21437408	21453387	chr16	3704296	3688317
5244	CP026252.1	21436668	21437275	chr16	3705036	3704429
5245	CP026252.1	21408011	21436558	chr16	3733693	3705146

5246	CP026252.1	21390331	21407454	chr16	3751373	3734250
5247	CP026252.1	21383319	21389667	chr16	3758385	3752037
5248	CP026252.1	21372657	21383190	chr16	3769047	3758514
5249	CP026252.1	21370443	21372480	chr16	3771261	3769224
5250	CP026252.1	21368048	21369541	chr16	3773656	3772163
5251	CP026252.1	21358470	21367883	chr16	3783234	3773821
5252	CP026252.1	21337753	21358091	chr16	3803951	3783613
5253	CP026252.1	21335184	21337216	chr16	3806520	3804488
5254	CP026252.1	21311384	21334868	chr16	3830320	3806836
5255	CP026252.1	21302369	21311138	chr16	3839335	3830566
5256	CP026252.1	21272571	21302058	chr16	3869133	3839646
5257	CP026252.1	21265190	21270934	chr16	3876514	3870770
5258	CP026252.1	21260290	21263499	chr16	3881414	3878205
5259	CP026252.1	21257965	21260182	chr16	3883739	3881522
5260	CP026252.1	21256475	21257681	chr16	3885229	3884023
5261	CP026252.1	21253435	21256345	chr16	3888269	3885359
5262	CP026252.1	21247561	21252396	chr16	3894143	3889308
5263	CP026252.1	21241646	21247360	chr16	3900058	3894344
5264	CP026252.1	21240334	21241106	chr16	3901370	3900598
5265	CP026252.1	21239570	21240221	chr16	3902134	3901483
5266	CP026252.1	21237615	21239369	chr16	3904089	3902335
5267	CP026252.1	21228646	21237087	chr16	3913058	3904617
5268	CP026252.1	21226328	21228195	chr16	3915376	3913509
5269	CP026252.1	21221207	21225582	chr16	3920497	3916122
5270	CP026252.1	21218106	21221099	chr16	3923598	3920605
5271	CP026252.1	21201489	21217845	chr16	3940215	3923859
5272	CP026252.1	21197197	21198429	chr16	3944507	3943275
5273	CP026252.1	21194439	21197046	chr16	3947265	3944658
5274	CP026252.1	21190982	21194186	chr16	3950722	3947518

5275	CP026252.1	21183392	21189823	chr16	3958312	3951881
5276	CP026252.1	21180888	21182272	chr16	3960816	3959432
5277	CP026252.1	21178093	21179435	chr16	3963611	3962269
5278	CP026252.1	21170256	21176341	chr16	3971448	3965363
5279	CP026252.1	21166970	21168060	chr16	3974734	3973644
5280	CP026252.1	21159962	21165294	chr16	3981742	3976410
5281	CP026252.1	21156766	21158601	chr16	3984938	3983103
5282	CP026252.1	21152995	21155418	chr16	3988709	3986286
5283	CP026252.1	21151261	21152516	chr16	3990443	3989188
5284	CP026252.1	21147137	21150841	chr16	3994567	3990863
5285	CP026252.1	21141489	21144737	chr16	4000215	3996967
5286	CP026252.1	21138522	21141361	chr16	4003182	4000343
5287	CP026252.1	21135143	21138044	chr16	4006561	4003660
5288	CP026252.1	21130365	21133686	chr16	4011339	4008018
5289	CP026252.1	21106493	21130040	chr16	4035211	4011664
5290	CP026252.1	21034597	21106300	chr16	4107107	4035404
5291	CP026252.1	21030759	21033882	chr16	4110945	4107822
5292	CP026252.1	21024063	21029948	chr16	4117641	4111756
5293	CP026252.1	21019222	21023502	chr16	4122482	4118202
5294	CP026252.1	20936443	21019050	chr16	4205261	4122654
5295	CP026252.1	20921088	20936254	chr16	4220616	4205450
5296	CP026252.1	20809086	20919293	chr16	4332618	4222411
5297	CP026252.1	20769316	20808867	chr16	4372388	4332837
5298	CP026252.1	20765973	20769229	chr16	4375731	4372475
5299	CP026252.1	20732064	20765666	chr16	4409640	4376038
5300	CP026252.1	20716194	20731963	chr16	4425510	4409741
5301	CP026252.1	20655119	20715806	chr16	4486585	4425898
5302	CP026252.1	20632257	20652273	chr16	4509447	4489431
5303	CP026252.1	20629365	20631953	chr16	4512339	4509751

5304	CP026252.1	20622027	20626920	chr16	4519677	4514784
5305	CP026252.1	20611306	20620947	chr16	4530398	4520757
5306	CP026252.1	20609010	20610315	chr16	4532694	4531389
5307	CP026252.1	20553045	20608869	chr16	4588659	4532835
5308	CP026252.1	20524108	20552796	chr16	4617596	4588908
5309	CP026252.1	20509522	20524003	chr16	4632182	4617701
5310	CP026252.1	20503063	20509428	chr16	4638641	4632276
5311	CP026252.1	20485135	20501482	chr16	4656569	4640222
5312	CP026252.1	20473359	20485033	chr16	4668345	4656671
5313	CP026252.1	20405660	20473239	chr16	4736044	4668465
5314	CP026252.1	20396571	20405537	chr16	4745133	4736167
5315	CP026252.1	20359396	20396390	chr16	4782308	4745314
5316	CP026252.1	20326613	20359196	chr16	4815091	4782508
5317	CP026252.1	20320059	20326473	chr16	4821645	4815231
5318	CP026252.1	20313159	20319893	chr16	4828545	4821811
5319	CP026252.1	20255066	20313041	chr16	4886638	4828663
5320	CP026252.1	20244120	20254706	chr16	4897584	4886998
5321	CP026252.1	20237741	20239103	chr16	4903963	4902601
5322	CP026252.1	20230268	20237507	chr16	4911436	4904197
5323	CP026252.1	20218402	20229964	chr16	4923302	4911740
5324	CP026252.1	20182512	20218195	chr16	4959192	4923509
5325	CP026252.1	20152809	20182389	chr16	4988895	4959315
5326	CP026252.1	20142910	20152498	chr16	4998794	4989206
5327	CP026252.1	20120613	20142708	chr16	5021091	4998996
5328	CP026252.1	20115668	20120517	chr16	5026036	5021187
5329	CP026252.1	20044231	20115256	chr16	5097473	5026448
5330	CP026252.1	20038412	20044137	chr16	5103292	5097567
5331	CP026252.1	20027250	20038248	chr16	5114454	5103456
5332	CP026252.1	20017531	20027085	chr16	5124173	5114619

5333	CP026252.1	19928176	20017426	chr16	5213528	5124278
5334	CP026252.1	19900040	19927167	chr16	5241664	5214537
5335	CP026252.1	19861848	19899883	chr16	5279856	5241821
5336	CP026252.1	19849011	19861668	chr16	5292693	5280036
5337	CP026252.1	19820976	19848924	chr16	5320728	5292780
5338	CP026252.1	19787495	19820851	chr16	5354209	5320853
5339	CP026252.1	19780619	19787394	chr16	5361085	5354310
5340	CP026252.1	19752667	19780506	chr16	5389037	5361198
5341	CP026252.1	19729750	19752558	chr16	5411954	5389146
5342	CP026252.1	19710310	19729413	chr16	5431394	5412291
5343	CP026252.1	19682064	19709185	chr16	5459640	5432519
5344	CP026252.1	19597946	19681694	chr16	5543758	5460010
5345	CP026252.1	19548324	19597708	chr16	5593380	5543996
5346	CP026252.1	19481809	19548097	chr16	5659895	5593607
5347	CP026252.1	19454716	19481601	chr16	5686988	5660103
5348	CP026252.1	19423038	19454577	chr16	5718666	5687127
5349	CP026252.1	19419806	19421295	chr16	5721898	5720409
5350	CP026252.1	19400330	19419511	chr16	5741374	5722193
5351	CP026252.1	19390476	19399724	chr16	5751228	5741980
5352	CP026252.1	19366560	19389782	chr16	5775144	5751922
5353	CP026252.1	19292750	19366436	chr16	5848954	5775268
5354	CP026252.1	19285540	19291540	chr16	5856164	5850164
5355	CP026252.1	19281850	19285445	chr16	5859854	5856259
5356	CP026252.1	19274064	19280927	chr16	5867640	5860777
5357	CP026252.1	19267006	19273598	chr16	5874698	5868106
5358	CP026252.1	19230909	19266346	chr16	5910795	5875358
5359	CP026252.1	19186752	19229956	chr16	5954952	5911748
5360	CP026252.1	19089482	19186641	chr16	6052222	5955063
5361	CP026252.1	19086741	19089029	chr16	6054963	6052675

5362	CP026252.1	19076759	19086597	chr16	6064945	6055107
5363	CP026252.1	19070152	19076492	chr16	6071552	6065212
5364	CP026252.1	19057913	19068126	chr16	6083791	6073578
5365	CP026252.1	19052820	19057562	chr16	6088884	6084142
5366	CP026252.1	19034676	19052675	chr16	6107028	6089029
5367	CP026252.1	19026791	19033641	chr16	6114913	6108063
5368	CP026252.1	18951122	19026655	chr16	6190582	6115049
5369	CP026252.1	18947663	18950983	chr16	6194041	6190721
5370	CP026252.1	18915196	18947516	chr16	6226508	6194188
5371	CP026252.1	18882900	18915093	chr16	6258804	6226611
5372	CP026252.1	18877917	18882538	chr16	6263787	6259166
5373	CP026252.1	18861136	18877799	chr16	6280568	6263905
5374	CP026252.1	18848345	18861000	chr16	6293359	6280704
5375	CP026252.1	18717766	18848004	chr16	6423938	6293700
5376	CP026252.1	18689474	18717590	chr16	6452230	6424114
5377	CP026252.1	18683563	18689242	chr16	6458141	6452462
5378	CP026252.1	18671283	18683215	chr16	6470421	6458489
5379	CP026252.1	18646221	18670611	chr16	6495483	6471093
5380	CP026252.1	18531209	18645998	chr16	6610495	6495706
5381	CP026252.1	18345892	18530803	chr16	6795812	6610901
5382	CP026252.1	18328150	18345743	chr16	6813554	6795961
5383	CP026252.1	18297218	18328027	chr16	6844486	6813677
5384	CP026252.1	18293861	18296829	chr16	6847843	6844875
5385	CP026252.1	18280917	18293750	chr16	6860787	6847954
5386	CP026252.1	18183972	18280735	chr16	6957732	6860969
5387	CP026252.1	18171757	18172061	chr16	6958137	6957833
5388	CP026252.1	18105120	18164009	chr16	7024774	6965885
5389	CP026252.1	18067170	18104879	chr16	7062724	7025015
5390	CP026252.1	18028301	18067008	chr16	7101593	7062886

5391	CP026252.1	17980832	18027778	chr16	7149062	7102116
5392	CP026252.1	17918105	17980545	chr16	7211789	7149349
5393	CP026252.1	17876814	17918019	chr16	7253080	7211875
5394	CP026252.1	17776517	17876345	chr16	7353377	7253549
5395	CP026252.1	17766713	17776253	chr16	7363181	7353641
5396	CP026252.1	17665576	17766566	chr16	7464318	7363328
5397	CP026252.1	17633099	17665216	chr16	7496795	7464678
5398	CP026252.1	17590871	17632941	chr16	7539023	7496953
5399	CP026252.1	17532051	17590682	chr16	7597843	7539212
5400	CP026252.1	17478386	17531766	chr16	7651508	7598128
5401	CP026252.1	17356610	17477602	chr16	7773284	7652292
5402	CP026252.1	17242560	17355099	chr16	7887334	7774795
5403	CP026252.1	17230776	17242087	chr16	7899118	7887807
5404	CP026252.1	17222579	17230647	chr16	7907315	7899247
5405	CP026252.1	17207437	17222388	chr16	7922457	7907506
5406	CP026252.1	17114359	17207331	chr16	8015535	7922563
5407	CP026252.1	17089046	17114187	chr16	8040848	8015707
5408	CP026252.1	16610167	17088930	chr16	8519727	8040964
5409	CP026252.1	16423005	16609979	chr16	8706889	8519915
5410	CP026252.1	16395897	16422787	chr16	8733997	8707107
5411	CP026252.1	16305388	16395694	chr16	8824506	8734200
5412	CP026252.1	16132802	16305300	chr16	8997092	8824594
5413	CP026252.1	16048531	16132097	chr16	9081363	8997797
5414	CP026252.1	16007642	16047973	chr16	9122252	9081921
5415	CP026252.1	15844995	16007037	chr16	9284899	9122857
5416	CP026252.1	15581438	15844905	chr16	9548456	9284989
5417	CP026252.1	15541460	15581144	chr16	9588434	9548750
5418	CP026252.1	15513664	15540598	chr16	9616230	9589296
5419	CP026252.1	15486502	15512766	chr16	9643392	9617128

5420	CP026252.1	15347124	15486351	chr16	9782770	9643543
5421	CP026252.1	15268201	15346905	chr16	9861693	9782989
5422	CP026252.1	15213863	15266577	chr16	9916031	9863317
5423	CP026252.1	15138732	15213253	chr16	9991162	9916641
5424	CP026252.1	15114764	15138465	chr16	10015130	9991429
5425	CP026252.1	14900633	15114615	chr16	10229261	10015279
5426	CP026252.1	14881928	14899020	chr16	10247966	10230874
5427	CP026252.1	14876613	14880595	chr16	10253281	10249299
5428	CP026252.1	14862793	14876443	chr16	10267101	10253451
5429	CP026252.1	14857210	14862691	chr16	10272684	10267203
5430	CP026252.1	14763035	14856987	chr16	10366859	10272907
5431	CP026252.1	14691025	14762893	chr16	10438869	10367001
5432	CP026252.1	14690045	14690937	chr16	10439849	10438957
5433	CP026252.1	14688600	14689929	chr16	10441294	10439965
5434	CP026252.1	14601869	14688437	chr16	10528025	10441457
5435	CP026252.1	14589986	14601647	chr16	10539908	10528247
5436	CP026252.1	14377705	14588360	chr16	10752189	10541534
5437	CP026252.1	14201405	14377615	chr16	10928489	10752279
5438	CP026252.1	14161351	14201277	chr16	10968543	10928617
5439	CP026252.1	13901670	14159727	chr16	11228224	10970167
5440	CP026252.1	13879003	13899553	chr16	11250891	11230341
5441	CP026252.1	13863388	13878875	chr16	11266506	11251019
5442	CP026252.1	13859918	13863214	chr16	11269976	11266680
5443	CP026252.1	13857256	13859692	chr16	11272638	11270202
5444	CP026252.1	13775906	13857163	chr16	11353988	11272731
5445	CP026252.1	13625719	13775259	chr16	11504175	11354635
5446	CP026252.1	13532297	13625457	chr16	11597597	11504437
5447	CP026252.1	13384218	13530635	chr16	11745676	11599259
5448	CP026252.1	13255349	13384123	chr16	11874545	11745771

5449	CP026252.1	13205605	13255165	chr16	11924289	11874729
5450	CP026252.1	13167421	13205439	chr16	11962473	11924455
5451	CP026252.1	13160797	13167019	chr16	11969097	11962875
5452	CP026252.1	13159023	13160108	chr16	11970871	11969786
5453	CP026252.1	12970565	13158911	chr16	12159329	11970983
5454	CP026252.1	12966258	12970458	chr16	12163636	12159436
5455	CP026252.1	12964003	12965794	chr16	12165891	12164100
5456	CP026252.1	12948471	12963844	chr16	12181423	12166050
5457	CP026252.1	12815589	12946726	chr16	12314305	12183168
5458	CP026252.1	12648831	12815419	chr16	12481063	12314475
5459	CP026252.1	12644959	12647799	chr16	12484935	12482095
5460	CP026252.1	12640543	12644862	chr16	12489351	12485032
5461	CP026252.1	12542622	12640436	chr16	12587272	12489458
5462	CP026252.1	12486998	12541590	chr16	12642896	12588304
5463	CP026252.1	12405617	12486893	chr16	12724277	12643001
5464	CP026252.1	12259232	12405494	chr16	12870662	12724400
5465	CP026252.1	12209338	12258627	chr16	12920556	12871267
5466	CP026252.1	11845628	12208225	chr16	13284266	12921669
5467	CP026252.1	11612664	11845307	chr16	13517230	13284587
5468	CP026252.1	11587564	11612509	chr16	13542330	13517385
5469	CP026252.1	11466954	11585528	chr16	13662940	13544366
5470	CP026252.1	11453146	11466722	chr16	13676748	13663172
5471	CP026252.1	11187459	11452918	chr16	13942435	13676976
5472	CP026252.1	11104730	11186885	chr16	14025164	13943009
5473	CP026252.1	11028715	11103289	chr16	14101179	14026605
5474	CP026252.1	10939572	11028373	chr16	14190322	14101521
5475	CP026252.1	10888553	10935568	chr16	14241341	14194326
5476	CP026252.1	10873663	10888247	chr16	14256231	14241647
5477	CP026252.1	10843888	10873541	chr16	14286006	14256353

5478	CP026252.1	10756550	10843667	chr16	14373344	14286227
5479	CP026252.1	10732850	10756424	chr16	14397044	14373470
5480	CP026252.1	10568584	10732573	chr16	14561310	14397321
5481	CP026252.1	10478846	10568317	chr16	14651048	14561577
5482	CP026252.1	10322776	10477916	chr16	14807118	14651978
5483	CP026252.1	9959545	10322639	chr16	15170349	14807255
5484	CP026252.1	9890391	9959415	chr16	15239503	15170479
5485	CP026252.1	9812030	9889368	chr16	15317864	15240526
5486	CP026252.1	9781600	9811844	chr16	15348294	15318050
5487	CP026252.1	9594651	9781474	chr16	15535243	15348420
5488	CP026252.1	9548519	9594515	chr16	15581375	15535379
5489	CP026252.1	9532620	9547731	chr16	15597274	15582163
5490	CP026252.1	9491041	9532431	chr16	15638853	15597463
5491	CP026252.1	9312786	9490879	chr16	15817108	15639015
5492	CP026252.1	9233818	9312667	chr16	15896076	15817227
5493	CP026252.1	9045259	9233711	chr16	16084635	15896183
5494	CP026252.1	9021579	9045127	chr16	16108315	16084767
5495	CP026252.1	9000557	9021463	chr16	16129337	16108431
5496	CP026252.1	8972622	9000380	chr16	16157272	16129514
5497	CP026254.1	22937937	22938114	chr17	842438	842260
5498	CP026254.1	16932932	17014911	chr17	6851556	6769577
5499	CP026254.1	16911702	16932802	chr17	6872786	6851686
5500	CP026254.1	16884252	16911480	chr17	6900236	6873008
5501	CP026254.1	16730580	16884160	chr17	7053908	6900328
5502	CP026254.1	16611228	16730182	chr17	7173260	7054306
5503	CP026254.1	16605176	16609908	chr17	7179312	7174580
5504	CP026254.1	16551479	16605043	chr17	7233009	7179445
5505	CP026254.1	16393220	16551287	chr17	7391268	7233201
5506	CP026254.1	16375638	16392315	chr17	7408850	7392173

5507	CP026254.1	16326204	16375324	chr17	7458284	7409164
5508	CP026254.1	16311671	16325979	chr17	7472817	7458509
5509	CP026254.1	16217795	16311136	chr17	7566693	7473352
5510	CP026254.1	16026065	16217654	chr17	7758423	7566834
5511	CP026254.1	16023121	16025975	chr17	7761367	7758513
5512	CP026254.1	15937981	16023007	chr17	7846507	7761481
5513	CP026254.1	15933697	15937852	chr17	7850791	7846636
5514	CP026254.1	15876477	15933460	chr17	7908011	7851028
5515	CP026254.1	15840324	15876299	chr17	7944164	7908189
5516	CP026254.1	15805579	15840232	chr17	7978909	7944256
5517	CP026254.1	15788406	15805399	chr17	7996082	7979089
5518	CP026254.1	15673536	15788149	chr17	8110952	7996339
5519	CP026254.1	15664579	15673346	chr17	8119909	8111142
5520	CP026254.1	15660383	15664310	chr17	8124105	8120178
5521	CP026254.1	15485812	15660101	chr17	8298676	8124387
5522	CP026254.1	15460763	15485665	chr17	8323725	8298823
5523	CP026254.1	15437756	15460557	chr17	8346732	8323931
5524	CP026254.1	15425928	15437595	chr17	8358560	8346893
5525	CP026254.1	15409938	15425714	chr17	8374550	8358774
5526	CP026254.1	15362006	15409731	chr17	8422482	8374757
5527	CP026254.1	15281393	15361703	chr17	8503095	8422785
5528	CP026254.1	15256902	15281294	chr17	8527586	8503194
5529	CP026254.1	15207516	15256705	chr17	8576972	8527783
5530	CP026254.1	15009602	15207425	chr17	8774886	8577063
5531	CP026254.1	14999573	15009514	chr17	8784915	8774974
5532	CP026254.1	14723580	14999178	chr17	9060908	8785310
5533	CP026254.1	14683498	14723347	chr17	9100990	9061141
5534	CP026254.1	14668685	14681955	chr17	9115803	9102533
5535	CP026254.1	14654607	14668597	chr17	9129881	9115891

5536	CP026254.1	14615827	14654492	chr17	9168661	9129996
5537	CP026254.1	14595458	14615658	chr17	9189030	9168830
5538	CP026254.1	14592580	14595279	chr17	9191908	9189209
5539	CP026254.1	14586069	14592487	chr17	9198419	9192001
5540	CP026254.1	14576197	14583319	chr17	9208291	9201169
5541	CP026254.1	14559678	14575974	chr17	9224810	9208514
5542	CP026254.1	14555695	14559370	chr17	9228793	9225118
5543	CP026254.1	14539896	14555541	chr17	9244592	9228947
5544	CP026254.1	14491249	14539784	chr17	9293239	9244704
5545	CP026254.1	14446600	14490262	chr17	9337888	9294226
5546	CP026254.1	14424360	14443019	chr17	9360128	9341469
5547	CP026254.1	14402007	14424089	chr17	9382481	9360399
5548	CP026254.1	14386515	14401405	chr17	9397973	9383083
5549	CP026254.1	14307618	14385393	chr17	9476870	9399095
5550	CP026254.1	14282555	14307233	chr17	9501933	9477255
5551	CP026254.1	14242156	14282454	chr17	9542332	9502034
5552	CP026254.1	14173568	14241994	chr17	9610920	9542494
5553	CP026254.1	14157439	14173247	chr17	9627049	9611241
5554	CP026254.1	14156544	14157350	chr17	9627944	9627138
5555	CP026254.1	14128520	14156347	chr17	9655968	9628141
5556	CP026254.1	14073071	14128351	chr17	9711417	9656137
5557	CP026254.1	14039832	14072909	chr17	9744656	9711579
5558	CP026254.1	14013579	14039569	chr17	9770909	9744919
5559	CP026254.1	13976029	14013470	chr17	9808459	9771018
5560	CP026254.1	13925225	13975884	chr17	9859263	9808604
5561	CP026254.1	13846068	13925052	chr17	9938420	9859436
5562	CP026254.1	13825339	13845281	chr17	9959149	9939207
5563	CP026254.1	13816971	13825252	chr17	9967517	9959236
5564	CP026254.1	13522689	13816837	chr17	10261799	9967651

5565	CP026254.1	13289947	13522455	chr17	10494541	10262033
5566	CP026254.1	12907883	13289687	chr17	10876605	10494801
5567	CP026254.1	12801515	12907482	chr17	10982973	10877006
5568	CP026254.1	12411708	12801319	chr17	11372780	10983169
5569	CP026254.1	12325851	12411548	chr17	11458637	11372940
5570	CP026254.1	12095683	12324876	chr17	11688805	11459612
5571	CP026254.1	12088111	12095585	chr17	11696377	11688903
5572	CP026254.1	11879119	12086645	chr17	11905369	11697843
5573	CP026254.1	11846328	11878982	chr17	11938160	11905506
5574	CP026254.1	11823897	11846237	chr17	11960591	11938251
5575	CP026254.1	11801699	11823624	chr17	11982789	11960864
5576	CP026254.1	11494480	11801506	chr17	12290008	11982982
5577	CP026254.1	11326565	11494369	chr17	12457923	12290119
5578	CP026254.1	11225146	11325841	chr17	12559342	12458647
5579	CP026254.1	11095306	11224973	chr17	12689182	12559515
5580	CP026254.1	11087606	11095099	chr17	12696882	12689389
5581	CP026254.1	11009315	11087384	chr17	12775173	12697104
5582	CP026254.1	10987536	11009130	chr17	12796952	12775358
5583	CP026254.1	10962925	10987439	chr17	12821563	12797049
5584	CP026254.1	10796782	10962817	chr17	12987706	12821671
5585	CP026254.1	10780224	10796485	chr17	13004264	12988003
5586	CP026254.1	10750022	10780131	chr17	13034466	13004357
5587	CP026254.1	10728824	10749897	chr17	13055664	13034591
5588	CP026254.1	10609954	10728359	chr17	13174534	13056129
5589	CP026254.1	10608056	10609500	chr17	13176432	13174988
5590	CP026254.1	10605647	10607909	chr17	13178841	13176579
5591	CP026254.1	10557598	10605467	chr17	13226890	13179021
5592	CP026254.1	10495784	10557123	chr17	13288704	13227365
5593	CP026254.1	10336959	10495672	chr17	13447529	13288816

5594	CP026254.1	10301466	10336792	chr17	13483022	13447696
5595	CP026254.1	10140868	10301248	chr17	13643620	13483240
5596	CP026254.1	10137691	10140782	chr17	13646797	13643706
5597	CP026254.1	10132047	10136863	chr17	13652441	13647625
5598	CP026254.1	10127359	10131754	chr17	13657129	13652734
5599	CP026254.1	10120932	10126977	chr17	13663556	13657511
5600	CP026254.1	9609228	10120684	chr17	14175260	13663804
5601	CP026254.1	9599791	9609109	chr17	14184697	14175379
5602	CP026254.1	9591518	9596358	chr17	14192970	14188130
5603	CP026254.1	9551957	9591432	chr17	14232531	14193056
5604	CP026254.1	9455680	9551847	chr17	14328808	14232641
5605	CP026254.1	9441865	9455407	chr17	14342623	14329081
5606	CP026254.1	9422920	9441757	chr17	14361568	14342731
5607	CP026254.1	9414722	9422781	chr17	14369766	14361707
5608	CP026254.1	9404016	9414553	chr17	14380472	14369935
5609	CP026254.1	9120036	9403909	chr17	14664452	14380579
5610	CP026254.1	9057882	9119702	chr17	14726606	14664786
5611	CP026254.1	9021639	9057632	chr17	14762849	14726856
5612	CP026254.1	9018883	9021508	chr17	14765605	14762980
5613	CP026254.1	8771827	9018562	chr17	15012661	14765926
5614	CP026254.1	8760668	8771712	chr17	15023820	15012776
5615	CP026254.1	8697460	8760450	chr17	15087028	15024038
5616	CP026254.1	8694418	8697338	chr17	15090070	15087150
5617	CP026254.1	8611192	8694299	chr17	15173296	15090189
5618	CP026254.1	8560991	8610617	chr17	15223497	15173871
5619	CP026254.1	8275939	8560869	chr17	15508549	15223619
5620	CP026254.1	8219278	8275615	chr17	15565210	15508873
5621	CP026254.1	8215194	8219142	chr17	15569294	15565346
5622	CP026254.1	8129116	8215015	chr17	15655372	15569473

5623	CP026254.1	8097347	8128876	chr17	15687141	15655612
5624	CP026254.1	8072426	8096367	chr17	15712062	15688121
5625	CP026254.1	7966997	8072040	chr17	15817491	15712448
5626	CP026254.1	7836485	7966682	chr17	15948003	15817806
5627	CP026254.1	7818600	7836377	chr17	15965888	15948111
5628	CP026254.1	7814656	7818463	chr17	15969832	15966025
5629	CP026254.1	7689554	7814543	chr17	16094934	15969945
5630	CP026254.1	7655596	7689463	chr17	16128892	16095025
5631	CP026254.1	7651225	7655384	chr17	16133263	16129104
5632	CP026254.1	7621488	7651089	chr17	16163000	16133399
5633	CP026254.1	7616238	7621284	chr17	16168250	16163204
5634	CP026254.1	7572288	7615412	chr17	16212200	16169076
5635	CP026254.1	7567076	7571980	chr17	16217412	16212508
5636	CP026254.1	7554442	7566861	chr17	16230046	16217627
5637	CP026254.1	7539673	7553810	chr17	16244815	16230678
5638	CP026254.1	7534388	7539423	chr17	16250100	16245065
5639	CP026254.1	7447905	7534193	chr17	16336583	16250295
5640	CP026254.1	7287795	7447512	chr17	16496693	16336976
5641	CP026254.1	7195483	7287629	chr17	16589005	16496859
5642	CP026254.1	7171977	7195293	chr17	16612511	16589195
5643	CP026254.1	7169855	7171808	chr17	16614633	16612680
5644	CP026254.1	6876244	7169747	chr17	16908244	16614741
5645	CP026254.1	6869290	6876064	chr17	16915198	16908424
5646	CP026254.1	6734340	6869177	chr17	17050148	16915311
5647	CP026254.1	6722638	6734232	chr17	17061850	17050256
5648	CP026254.1	6715737	6722355	chr17	17068751	17062133
5649	CP026254.1	6689883	6715646	chr17	17094605	17068842
5650	CP026254.1	6655513	6689748	chr17	17128975	17094740
5651	CP026254.1	6648184	6655215	chr17	17136304	17129273

5652	CP026254.1	6566180	6648007	chr17	17218308	17136481
5653	CP026254.1	6520122	6565815	chr17	17264366	17218673
5654	CP026254.1	6496626	6519901	chr17	17287862	17264587
5655	CP026254.1	6476495	6496373	chr17	17307993	17288115
5656	CP026254.1	6467275	6476402	chr17	17317213	17308086
5657	CP026254.1	6454429	6465996	chr17	17330059	17318492
5658	CP026254.1	6416246	6454297	chr17	17368242	17330191
5659	CP026254.1	6398054	6415674	chr17	17386434	17368814
5660	CP026254.1	6389511	6397677	chr17	17394977	17386811
5661	CP026254.1	6303008	6388210	chr17	17481480	17396278
5662	CP026254.1	6298454	6302150	chr17	17486034	17482338
5663	CP026254.1	6293040	6295825	chr17	17491448	17488663
5664	CP026254.1	6288980	6292628	chr17	17495508	17491860
5665	CP026254.1	6263529	6288875	chr17	17520959	17495613
5666	CP026254.1	6249176	6263285	chr17	17535312	17521203
5667	CP026254.1	6239154	6249083	chr17	17545334	17535405
5668	CP026254.1	6197748	6238151	chr17	17586740	17546337
5669	CP026254.1	6178494	6197050	chr17	17605994	17587438
5670	CP026254.1	6174132	6178132	chr17	17610356	17606356
5671	CP026254.1	6164726	6173272	chr17	17619762	17611216
5672	CP026254.1	6124423	6163521	chr17	17660065	17620967
5673	CP026254.1	6117247	6124150	chr17	17667241	17660338
5674	CP026254.1	6113000	6116692	chr17	17671488	17667796
5675	CP026254.1	6111155	6112808	chr17	17673333	17671680
5676	CP026254.1	6098071	6111052	chr17	17686417	17673436
5677	CP026254.1	6093913	6096594	chr17	17690575	17687894
5678	CP026254.1	6090783	6093563	chr17	17693705	17690925
5679	CP026254.1	6077163	6090417	chr17	17707325	17694071
5680	CP026254.1	6019648	6076749	chr17	17764840	17707739

5681	CP026254.1	6013967	6019538	chr17	17770521	17764950
5682	CP026254.1	5994614	6013879	chr17	17789874	17770609
5683	CP026254.1	5969519	5994392	chr17	17814969	17790096
5684	CP026254.1	5930748	5969284	chr17	17853740	17815204
5685	CP026254.1	5907658	5929525	chr17	17876830	17854963
5686	CP026254.1	5903626	5907427	chr17	17880862	17877061
5687	CP026254.1	5815079	5902581	chr17	17969409	17881907
5688	CP026254.1	5769070	5814860	chr17	18015418	17969628
5689	CP026254.1	5757591	5768867	chr17	18026897	18015621
5690	CP026254.1	5727867	5757213	chr17	18056621	18027275
5691	CP026254.1	5718278	5727668	chr17	18066210	18056820
5692	CP026254.1	5715940	5718078	chr17	18068548	18066410
5693	CP026254.1	5704895	5715506	chr17	18079593	18068982
5694	CP026254.1	5696103	5704713	chr17	18088385	18079775
5695	CP026254.1	5688642	5695940	chr17	18095846	18088548
5696	CP026254.1	5665105	5688472	chr17	18119383	18096016
5697	CP026254.1	5663285	5664939	chr17	18121203	18119549
5698	CP026254.1	5655448	5663181	chr17	18129040	18121307
5699	CP026254.1	5639581	5654949	chr17	18144907	18129539
5700	CP026254.1	5632668	5639306	chr17	18151820	18145182
5701	CP026254.1	5624531	5632202	chr17	18159957	18152286
5702	CP026254.1	5619372	5624232	chr17	18165116	18160256
5703	CP026254.1	5608057	5618237	chr17	18176431	18166251
5704	CP026254.1	5603722	5606302	chr17	18180766	18178186
5705	CP026254.1	5569782	5603612	chr17	18214706	18180876
5706	CP026254.1	5560512	5569585	chr17	18223976	18214903
5707	CP026254.1	5530199	5560136	chr17	18254289	18224352
5708	CP026254.1	5516476	5529794	chr17	18268012	18254694
5709	CP026254.1	5514187	5516315	chr17	18270301	18268173

5710	CP026254.1	5482767	5513431	chr17	18301721	18271057
5711	CP026254.1	5439118	5482652	chr17	18345370	18301836
5712	CP026254.1	5427161	5439000	chr17	18357327	18345488
5713	CP026254.1	5345630	5427042	chr17	18438858	18357446
5714	CP026254.1	5317648	5345431	chr17	18466840	18439057
5715	CP026254.1	5158147	5317305	chr17	18626341	18467183
5716	CP026254.1	5156297	5157766	chr17	18628191	18626722
5717	CP026254.1	5099031	5154400	chr17	18685457	18630088
5718	CP026254.1	5038915	5098712	chr17	18745573	18685776
5719	CP026254.1	5016306	5038702	chr17	18768182	18745786
5720	CP026254.1	4999429	5016215	chr17	18785059	18768273
5721	CP026254.1	4973549	4999126	chr17	18810939	18785362
5722	CP026254.1	4969777	4973392	chr17	18814711	18811096
5723	CP026254.1	4957215	4969288	chr17	18827273	18815200
5724	CP026254.1	4954255	4956909	chr17	18830233	18827579
5725	CP026254.1	4915677	4954137	chr17	18868811	18830351
5726	CP026254.1	4903352	4915354	chr17	18881136	18869134
5727	CP026254.1	4898616	4903256	chr17	18885872	18881232
5728	CP026254.1	4895613	4898457	chr17	18888875	18886031
5729	CP026254.1	4890110	4895372	chr17	18894378	18889116
5730	CP026254.1	4825097	4889369	chr17	18959391	18895119
5731	CP026254.1	4823722	4824970	chr17	18960766	18959518
5732	CP026254.1	4800887	4823449	chr17	18983601	18961039
5733	CP026254.1	4791531	4800795	chr17	18992957	18983693
5734	CP026254.1	4786752	4791309	chr17	18997736	18993179
5735	CP026254.1	4763008	4786128	chr17	19021480	18998360
5736	CP026254.1	4756710	4762765	chr17	19027778	19021723
5737	CP026254.1	4683041	4690486	chr17	19101447	19094002
5738	CP026254.1	4577284	4617664	chr17	19207204	19166824

5739	CP026254.1	4447678	4468850	chr17	19336810	19315638
5740	CP026254.1	1731869	1734152	chr17	22051381	22049098
5741	CP026254.1	1655401	1670685	chr17	22127849	22112565
5742	CP026254.1	1572268	1587874	chr17	22210982	22195376
5743	CP026254.1	1510279	1512400	chr17	22272971	22270850
5744	CP026254.1	1397008	1447361	chr17	22386242	22335889
5745	CP026254.1	1332961	1335473	chr17	22450289	22447777
5746	CP026254.1	1263244	1270434	chr17	22520006	22512816
5747	CP026254.1	1157992	1195852	chr17	22625258	22587398
5748	CP026254.1	1038581	1078101	chr17	22744669	22705149
5749	CP026254.1	954334	974791	chr17	22828916	22808459
5750	CP026254.1	880281	885676	chr17	22902969	22897574
5751	CP026254.1	800266	820043	chr17	22982984	22963207
5752	CP026254.1	730997	738683	chr17	23052253	23044567
5753	CP026254.1	658664	668954	chr17	23124586	23114296
5754	CP026254.1	595523	599343	chr17	23187727	23183907
5755	CP026254.1	496825	531263	chr17	23286425	23251987
5756	CP026254.1	415158	424335	chr17	23368092	23358915
5757	CP026254.1	343045	346135	chr17	23440205	23437115
5758	CP026254.1	280393	283254	chr17	23502857	23499996
5759	CP026254.1	276915	279991	chr17	23506335	23503259
5760	CP026254.1	271134	276641	chr17	23512116	23506609
5761	CP026254.1	268103	270206	chr17	23515147	23513044
5762	CP026254.1	265180	267489	chr17	23518070	23515761
5763	CP026254.1	259206	264573	chr17	23524044	23518677
5764	CP026254.1	257126	258386	chr17	23526124	23524864
5765	CP026254.1	253716	255569	chr17	23529534	23527681
5766	CP026254.1	248353	249885	chr17	23534897	23533365
5767	CP026254.1	246254	247694	chr17	23536996	23535556

5768	CP026254.1	240555	243338	chr17	23542695	23539912
5769	CP026254.1	237062	239741	chr17	23546188	23543509
5770	CP026254.1	234973	236494	chr17	23548277	23546756
5771	CP026246.1	217997	220612	chr18	30072786	30015148
5772	CP026246.1	222480	229980	chr18	30014944	29988562
5773	CP026246.1	230961	234440	chr18	29987714	29964015
5774	CP026246.1	238514	239834	chr18	29963777	29909641
5775	CP026246.1	243540	245379	chr18	29909524	29855342
5776	CP026246.1	246017	250028	chr18	29855241	29829748
5777	CP026246.1	250591	252595	chr18	29817158	29777143
5778	CP026246.1	256248	264033	chr18	29776268	29756081
5779	CP026246.1	267640	272024	chr18	29755397	29662598
5780	CP026246.1	274114	275237	chr18	29662489	29551675
5781	CP026246.1	278827	280566	chr18	29551381	29524565
5782	CP026246.1	280855	282996	chr18	29524425	29460895
5783	CP026246.1	286300	302141	chr18	29460771	29290119
5784	CP026246.1	302273	306888	chr18	29289895	29247061
5785	CP026246.1	307422	327347	chr18	29246850	29243515
5786	CP026246.1	327539	329151	chr18	29243302	29239686
5787	CP026246.1	329269	354075	chr18	29239565	29229688
5788	CP026246.1	354308	361126	chr18	29229323	29226200
5789	CP026246.1	361303	362781	chr18	29226066	29222201
5790	CP026246.1	362879	376041	chr18	29221605	29217964
5791	CP026246.1	377532	384902	chr18	29216993	29186924
5792	CP026246.1	385720	409637	chr18	29184486	29167966
5793	CP026246.1	413009	428252	chr18	29167783	29145530
5794	CP026246.1	428638	453992	chr18	29144989	29107388
5795	CP026246.1	454137	466467	chr18	29107302	29105494
5796	CP026246.1	466568	471112	chr18	29105407	29099432

5797	CP026246.1	501078	509882	chr18	29099324	29095534
5798	CP026246.1	513492	517828	chr18	29095362	29061087
5799	CP026246.1	517939	521202	chr18	29060844	29005934
5800	CP026246.1	522852	539515	chr18	29005685	29000187
5801	CP026246.1	542532	547616	chr18	29000056	28919521
5802	CP026246.1	551141	575269	chr18	28919425	28871028
5803	CP026246.1	576221	581388	chr18	28870523	28842073
5804	CP026246.1	581554	587688	chr18	28835901	28790168
5805	CP026246.1	587815	606926	chr18	28789955	28784472
5806	CP026246.1	607715	618835	chr18	28784332	28780670
5807	CP026246.1	619407	621939	chr18	28780494	28774295
5808	CP026246.1	623072	624320	chr18	28774181	28763304
5809	CP026246.1	624443	627039	chr18	28762672	28636264
5810	CP026246.1	627429	633683	chr18	28636158	28613415
5811	CP026246.1	633804	653531	chr18	28612594	28501726
5812	CP026246.1	653945	660678	chr18	28500788	28429226
5813	CP026246.1	661303	687386	chr18	28429088	28414989
5814	CP026246.1	687815	692706	chr18	28413887	28362631
5815	CP026246.1	694252	704887	chr18	28362515	28359308
5816	CP026246.1	706319	711218	chr18	28359042	28315669
5817	CP026246.1	711728	721930	chr18	28312486	28240299
5818	CP026246.1	722671	725024	chr18	28238693	28195460
5819	CP026246.1	727359	732800	chr18	28195302	28181737
5820	CP026246.1	733972	741641	chr18	28180949	28146065
5821	CP026246.1	744307	785353	chr18	28145895	28128193
5822	CP026246.1	785798	808315	chr18	28128106	28126165
5823	CP026246.1	808605	812400	chr18	28125906	28114753
5824	CP026246.1	812625	818526	chr18	28114597	28101197
5825	CP026246.1	818653	821486	chr18	28101105	28063147

5826	CP026246.1	821918	825794	chr18	28062941	28060121
5827	CP026246.1	825943	832739	chr18	28059969	28043073
5828	CP026246.1	833302	839433	chr18	28041543	28033548
5829	CP026246.1	839747	843836	chr18	28031716	28007584
5830	CP026246.1	844004	852307	chr18	28006990	27994103
5831	CP026246.1	852506	860889	chr18	27993593	27992058
5832	CP026246.1	861411	870632	chr18	27991906	27929614
5833	CP026246.1	876945	878571	chr18	27928153	27911301
5834	CP026246.1	878937	881662	chr18	27911074	27895664
5835	CP026246.1	883472	886298	chr18	27895507	27871813
5836	CP026246.1	887387	894393	chr18	27871704	27754496
5837	CP026246.1	896233	907238	chr18	27753931	27740996
5838	CP026246.1	907339	910406	chr18	27740827	27645885
5839	CP026246.1	937645	939073	chr18	27645767	27623145
5840	CP026246.1	940231	951261	chr18	27622351	27619191
5841	CP026246.1	951352	962947	chr18	27619000	27606793
5842	CP026246.1	963042	1007017	chr18	26771719	26906658
5843	CP026246.1	1008028	1009317	chr18	26455504	26768612
5844	CP026246.1	1010325	1014789	chr18	26391988	26455343
5845	CP026246.1	1015352	1019307	chr18	26335939	26389320
5846	CP026246.1	1019720	1111715	chr18	26151074	26332710
5847	CP026246.1	1115475	1136224	chr18	26130280	26150959
5848	CP026246.1	1136677	1138500	chr18	26097346	26129933
5849	CP026246.1	1139490	1144057	chr18	25388768	25449191
5850	CP026246.1	1144678	1146650	chr18	25291024	25388487
5851	CP026246.1	1147373	1163998	chr18	25238169	25289198
5852	CP026246.1	1164436	1175309	chr18	25238068	25235433
5853	CP026246.1	1175410	1217159	chr18	25200123	25156887
5854	CP026246.1	1219268	1229176	chr18	25156778	25016988

5855	CP026246.1	1230693	1232497	chr18	25016879	24925383
5856	CP026246.1	1234187	1235341	chr18	24860701	24925282
5857	CP026246.1	1236817	1240295	chr18	24860600	24857744
5858	CP026246.1	1241970	1244217	chr18	24842172	24836375
5859	CP026246.1	1244356	1245493	chr18	24835309	24833559
5860	CP026246.1	1245993	1252338	chr18	24830587	24820492
5861	CP026246.1	1253266	1255118	chr18	24820340	24798764
5862	CP026246.1	1255737	1256996	chr18	24790031	24714304
5863	CP026246.1	1258355	1260084	chr18	24711741	24562179
5864	CP026246.1	1260666	1262578	chr18	24553210	24522678
5865	CP026246.1	1263861	1267592	chr18	24511214	24522577
5866	CP026246.1	1268796	1286428	chr18	24506084	24510794
5867	CP026246.1	1290283	1292152	chr18	24474373	24505739
5868	CP026246.1	1294383	1296073	chr18	24336325	24474238
5869	CP026246.1	1296206	1310260	chr18	24304134	24335447
5870	CP026246.1	1310614	1313880	chr18	24294323	24301701
5871	CP026246.1	1314024	1332037	chr18	24270041	24293734
5872	CP026246.1	1336057	1343530	chr18	24267364	24269909
5873	CP026246.1	1348423	1374181	chr18	24257335	24265985
5874	CP026246.1	1374725	1375802	chr18	24253042	24256199
5875	CP026246.1	1375897	1380886	chr18	24244324	24252943
5876	CP026246.1	1381473	1383057	chr18	24231329	24244169
5877	CP026246.1	1383685	1392985	chr18	24207250	24231076
5878	CP026246.1	1393823	1395734	chr18	24177723	24206377
5879	CP026246.1	1396202	1398934	chr18	24157630	24177569
5880	CP026246.1	1400441	1405752	chr18	24124674	24157314
5881	CP026246.1	1409718	1415729	chr18	24103757	24124569
5882	CP026246.1	1418048	1433223	chr18	24101001	24102723
5883	CP026246.1	1434100	1438985	chr18	24009793	24100262

5884	CP026246.1	1439462	1441671	chr18	23519466	23516106
5885	CP026246.1	1442887	1444590	chr18	23302500	23307196
5886	CP026246.1	1445544	1450840	chr18	23293178	23302006
5887	CP026246.1	1451387	1461441	chr18	23254708	23292051
5888	CP026246.1	1463109	1472235	chr18	23179209	23253896
5889	CP026246.1	1472372	1496561	chr18	23098225	23179116
5890	CP026246.1	1496654	1501970	chr18	23089520	23098111
5891	CP026246.1	1502408	1513071	chr18	22917924	23088799
5892	CP026246.1	1514576	1579824	chr18	22887618	22917813
5893	CP026246.1	1580472	1644651	chr18	22883479	22886748
5894	CP026246.1	1646390	1678145	chr18	22776984	22882496
5895	CP026246.1	1678402	1694591	chr18	22765330	22776588
5896	CP026246.1	1694831	1707681	chr18	22762986	22764971
5897	CP026246.1	1707812	1713821	chr18	22759447	22761984
5898	CP026246.1	1714080	1725457	chr18	22734118	22759359
5899	CP026246.1	1725922	1731103	chr18	22720689	22733210
5900	CP026246.1	1731894	1744946	chr18	22698738	22720536
5901	CP026246.1	1745232	1827072	chr18	22668319	22698240
5902	CP026246.1	1827354	1836769	chr18	22651777	22667932
5903	CP026246.1	1837473	1847094	chr18	22616128	22651422
5904	CP026246.1	1847221	1855327	chr18	22610539	22615401
5905	CP026246.1	1855719	1884464	chr18	22588073	22610043
5906	CP026246.1	1884774	1901409	chr18	22573166	22587972
5907	CP026246.1	1901532	1916934	chr18	22509742	22573004
5908	CP026246.1	1917071	1925706	chr18	22483872	22509648
5909	CP026246.1	1925794	1927248	chr18	22475603	22483471
5910	CP026246.1	1927672	1954687	chr18	22463418	22475481
5911	CP026246.1	1955761	1959915	chr18	22419671	22463205
5912	CP026246.1	1960155	1965477	chr18	22277491	22416129

5913	CP026246.1	1965607	1970385	chr18	22244356	22276334
5914	CP026246.1	1970965	1978285	chr18	22135400	22244150
5915	CP026246.1	1978945	1997894	chr18	22108225	22135235
5916	CP026246.1	1999306	2000449	chr18	22028515	22108116
5917	CP026246.1	2000810	2025470	chr18	21937946	22028266
5918	CP026246.1	2025558	2026996	chr18	21922599	21937643
5919	CP026246.1	2028855	2042377	chr18	21859041	21921864
5920	CP026246.1	2042594	2052288	chr18	21821592	21858240
5921	CP026246.1	2052720	2082740	chr18	21799800	21821403
5922	CP026246.1	2085883	2088783	chr18	21788219	21798205
5923	CP026246.1	2089143	2091165	chr18	21743937	21787964
5924	CP026246.1	2091317	2093380	chr18	21714116	21740493
5925	CP026246.1	2093702	2114487	chr18	21702138	21714018
5926	CP026246.1	2115960	2123287	chr18	21697430	21701904
5927	CP026246.1	2123595	2125837	chr18	21682074	21697318
5928	CP026246.1	2128581	2132821	chr18	21643118	21681805
5929	CP026246.1	2133036	2144424	chr18	21556092	21642941
5930	CP026246.1	2145111	2151566	chr18	21354356	21555579
5931	CP026246.1	2151737	2154557	chr18	21243159	21353704
5932	CP026246.1	2154646	2168215	chr18	21221240	21242959
5933	CP026246.1	2168444	2187745	chr18	21160190	21221102
5934	CP026246.1	2187938	2190405	chr18	21154467	21156415
5935	CP026246.1	2190618	2207232	chr18	21112018	21150364
5936	CP026246.1	2207409	2263391	chr18	21069131	21110437
5937	CP026246.1	2263998	2282414	chr18	21063853	21069039
5938	CP026246.1	2282678	2301434	chr18	21044715	21063729
5939	CP026246.1	2301954	2303644	chr18	21037236	21044494
5940	CP026246.1	2303777	2312008	chr18	20991496	21036723
5941	CP026246.1	2313001	2334991	chr18	20975111	20990536

5942	CP026246.1	2336693	2386157	chr18	20961715	20974989
5943	CP026246.1	2386374	2411828	chr18	20915624	20961560
5944	CP026246.1	2413496	2426279	chr18	20817957	20915532
5945	CP026246.1	2426864	2431643	chr18	20562409	20817637
5946	CP026246.1	2431940	2442995	chr18	20533100	20562056
5947	CP026246.1	2443276	2450612	chr18	20479395	20532827
5948	CP026246.1	2451789	2475082	chr18	20427375	20477090
5949	CP026246.1	2475868	2477776	chr18	20389548	20426911
5950	CP026246.1	2477921	2513279	chr18	20330222	20388990
5951	CP026246.1	2514372	2526277	chr18	20197763	20329943
5952	CP026246.1	2526400	2528090	chr18	20119934	20197612
5953	CP026246.1	2528550	2531967	chr18	20060024	20119745
5954	CP026246.1	2532106	2552226	chr18	19968795	20059872
5955	CP026246.1	2552429	2557601	chr18	19951271	19968687
5956	CP026246.1	2557758	2559938	chr18	19935020	19951106
5957	CP026246.1	2561200	2569507	chr18	19925725	19934525
5958	CP026246.1	2569894	2572048	chr18	19898344	19925507
5959	CP026246.1	2572545	2582113	chr18	19880136	19895653
5960	CP026246.1	2583461	2623100	chr18	19826417	19880035
5961	CP026246.1	2623314	2632759	chr18	19786057	19826280
5962	CP026246.1	2632939	2657218	chr18	19740259	19785271
5963	CP026246.1	2657337	2665116	chr18	19684960	19739999
5964	CP026246.1	2666575	2668525	chr18	19663347	19684620
5965	CP026246.1	2669655	2721793	chr18	19619910	19662577
5966	CP026246.1	2721910	2752786	chr18	19586532	19619584
5967	CP026246.1	2752968	2785344	chr18	19569044	19586341
5968	CP026246.1	2785558	2803315	chr18	19536003	19568684
5969	CP026246.1	2804211	2810654	chr18	19527335	19535845
5970	CP026246.1	2811631	2815010	chr18	19517507	19527124

5971	CP026246.1	2816586	2823252	chr18	19461028	19517235
5972	CP026246.1	2823475	2842463	chr18	19429793	19460845
5973	CP026246.1	2842559	2851901	chr18	19421839	19428140
5974	CP026246.1	2852850	2868876	chr18	19413810	19421737
5975	CP026246.1	2869084	2900280	chr18	19379435	19413660
5976	CP026246.1	2900395	2920334	chr18	19368935	19379234
5977	CP026246.1	2920764	2930822	chr18	19287466	19368407
5978	CP026246.1	2932300	2945359	chr18	19176026	19287283
5979	CP026246.1	2946819	2963874	chr18	18943304	18941991
5980	CP026246.1	2964005	2985083	chr18	18932458	19175812
5981	CP026246.1	2985190	3001069	chr18	18904991	18932341
5982	CP026246.1	3002197	3020598	chr18	18788744	18904898
5983	CP026246.1	3020866	3030442	chr18	18758791	18788636
5984	CP026246.1	3030633	3032335	chr18	18749937	18758581
5985	CP026246.1	3032872	3034918	chr18	18561434	18749792
5986	CP026246.1	3035282	3070806	chr18	18559092	18561218
5987	CP026246.1	3071141	3101107	chr18	18541487	18558992
5988	CP026246.1	3101324	3165571	chr18	18522534	18541386
5989	CP026246.1	3166177	3224704	chr18	18252212	18493706
5990	CP026246.1	3224942	3227869	chr18	18053523	18247730
5991	CP026246.1	3228021	3235984	chr18	17839736	18052376
5992	CP026246.1	3236302	3256299	chr18	17792590	17839522
5993	CP026246.1	3256650	3259657	chr18	17756204	17790519
5994	CP026246.1	3260111	3262034	chr18	17738718	17756103
5995	CP026246.1	3263426	3268706	chr18	17664791	17733352
5996	CP026246.1	3269301	3281005	chr18	17613234	17664093
5997	CP026246.1	3281134	3322810	chr18	17508070	17612944
5998	CP026246.1	3322953	3325086	chr18	17359773	17507732
5999	CP026246.1	3325206	3333905	chr18	17299354	17359418

6000	CP026246.1	3365576	3369567	chr18	17224619	17299021
6001	CP026246.1	5420278	5453212	chr18	17206811	17224313
6002	CP026246.1	5454730	5474744	chr18	17142577	17206207
6003	CP026246.1	5475033	5494639	chr18	17012673	17140627
6004	CP026246.1	5495016	5573806	chr18	16807345	17012463
6005	CP026246.1	5575782	5586796	chr18	16770501	16807250
6006	CP026246.1	5586962	5635643	chr18	16744929	16770411
6007	CP026246.1	5635845	5639022	chr18	16739457	16744556
6008	CP026246.1	5640176	5687714	chr18	16726074	16739345
6009	CP026246.1	5688218	5732961	chr18	16643088	16725814
6010	CP026246.1	5733227	5736123	chr18	16534509	16642919
6011	CP026246.1	5736332	5771341	chr18	16451645	16534297
6012	CP026246.1	5771896	5784546	chr18	16396708	16450880
6013	CP026246.1	5784722	5864341	chr18	16361309	16396470
6014	CP026246.1	5865611	5921838	chr18	16149095	16360725
6015	CP026246.1	5921946	5942031	chr18	16124431	16148979
6016	CP026246.1	5942446	5980248	chr18	15917665	16123607
6017	CP026246.1	5980349	6044917	chr18	15736621	15917562
6018	CP026246.1	6045905	6244495	chr18	15621665	15735659
6019	CP026246.1	6244625	6290579	chr18	15621564	15542760
6020	CP026246.1	6290824	6296319	chr18	15522529	15494176
6021	CP026246.1	6296561	6303370	chr18	15492379	15476666
6022	CP026246.1	6303611	6324532	chr18	15431982	15476565
6023	CP026246.1	6324657	6342935	chr18	15400823	15431878
6024	CP026246.1	6343196	6352539	chr18	15396245	15400713
6025	CP026246.1	6352923	6383168	chr18	15382382	15389335
6026	CP026246.1	6383299	6393729	chr18	15317539	15382281
6027	CP026246.1	6394947	6431140	chr18	15260494	15317438
6028	CP026246.1	6432555	6461620	chr18	15147104	15260385

6029	CP026246.1	6461818	6473848	chr18	15091338	15146830
6030	CP026246.1	6473955	6525549	chr18	15090129	15091226
6031	CP026246.1	6526377	6536975	chr18	15057741	15090010
6032	CP026246.1	6537117	6661116	chr18	15036849	15056500
6033	CP026246.1	6662074	6672482	chr18	15035633	15036712
6034	CP026246.1	6672895	6689775	chr18	14995169	15035321
6035	CP026246.1	6689976	6711644	chr18	14966724	14994997
6036	CP026246.1	6711848	6804356	chr18	14908502	14966602
6037	CP026246.1	6805139	6847486	chr18	14623127	14908371
6038	CP026246.1	6847695	6854992	chr18	14580285	14622997
6039	CP026246.1	6855109	6960979	chr18	14529681	14580123
6040	CP026246.1	6961472	7001556	chr18	14517523	14529444
6041	CP026246.1	7001650	7082474	chr18	14463822	14517383
6042	CP026246.1	7082575	7105821	chr18	14423917	14462725
6043	CP026246.1	7106545	7120466	chr18	14328031	14423704
6044	CP026246.1	7121325	7123167	chr18	14273414	14327918
6045	CP026246.1	7123299	7169284	chr18	14196027	14273328
6046	CP026246.1	7169776	7207433	chr18	14142340	14195543
6047	CP026246.1	7207521	7236423	chr18	14127076	14142200
6048	CP026246.1	7236608	7266401	chr18	14087941	14126969
6049	CP026246.1	7266517	7275684	chr18	13978625	14087826
6050	CP026246.1	7275814	7283047	chr18	13812890	13978518
6051	CP026246.1	7283301	7286083	chr18	13740472	13812029
6052	CP026246.1	7286304	7294073	chr18	13650444	13739497
6053	CP026246.1	7294198	7334885	chr18	13645457	13650325
6054	CP026246.1	7334974	7441443	chr18	13578639	13645060
6055	CP026246.1	7441808	7503176	chr18	13577036	13578553
6056	CP026246.1	7503716	7545551	chr18	13567023	13576211
6057	CP026246.1	7546191	7657738	chr18	13478633	13565839

6058	CP026246.1	7658017	7659729	chr18	13394800	13478179
6059	CP026246.1	7659830	7688692	chr18	13363206	13394637
6060	CP026246.1	7688792	7700699	chr18	13330848	13362826
6061	CP026246.1	7700815	7702532	chr18	13270218	13330697
6062	CP026246.1	7702684	7718881	chr18	13258546	13270032
6063	CP026246.1	7719758	7761722	chr18	13071369	13258429
6064	CP026246.1	7761941	7882092	chr18	13022370	13070854
6065	CP026246.1	7882566	7940922	chr18	12874919	13022265
6066	CP026246.1	7941027	8081383	chr18	12871314	12874770
6067	CP026246.1	8081476	8090351	chr18	12739036	12871103
6068	CP026246.1	8090454	8155456	chr18	12720350	12738823
6069	CP026246.1	8155889	8204673	chr18	12495345	12720140
6070	CP026246.1	8204777	8208282	chr18	12438391	12495058
6071	CP026246.1	8209097	8485623	chr18	12417744	12438212
6072	CP026246.1	8485827	8540237	chr18	12413410	12417256
6073	CP026246.1	8540352	8578230	chr18	12371355	12413284
6074	CP026246.1	8578383	8638289	chr18	12275666	12371254
6075	CP026246.1	8638379	8676865	chr18	12223105	12275498
6076	CP026246.1	8676974	8722184	chr18	12203189	12222979
6077	CP026246.1	8722321	8762687	chr18	12056159	12202942
6078	CP026246.1	8762934	8765096	chr18	12031144	12054909
6079	CP026246.1	8765228	8797860	chr18	11947111	12029384
6080	CP026246.1	8797961	8819467	chr18	11926283	11946726
6081	CP026246.1	8819555	8824018	chr18	11798319	11925929
6082	CP026246.1	8824143	8850205	chr18	11759467	11797856
6083	CP026246.1	8850945	8949812	chr18	11729792	11759367
6084	CP026246.1	8949918	9137673	chr18	11690032	11729386
6085	CP026246.1	9137763	9139460	chr18	11644863	11689841
6086	CP026246.1	9140601	9174639	chr18	11638732	11644718

6087	CP026246.1	9174762	9208152	chr18	11550980	11638196
6088	CP026246.1	9208336	9342878	chr18	11327135	11550378
6089	CP026246.1	9342983	9375017	chr18	11317778	11327006
6090	CP026246.1	9375189	9432306	chr18	11288135	11316562
6091	CP026246.1	9432515	9435879	chr18	11247263	11287839
6092	CP026246.1	9436105	9514007	chr18	11189171	11247054
6093	CP026246.1	9514142	9585688	chr18	11183665	11189021
6094	CP026246.1	9585797	9725086	chr18	11180834	11182443
6095	CP026246.1	9725197	9766635	chr18	11151871	11180538
6096	CP026246.1	9770273	9815832	chr18	11065385	11150543
6097	CP026246.1	9815974	9882128	chr18	10970711	11064283
6098	CP026246.1	9882342	9904078	chr18	10871869	10969797
6099	CP026246.1	9904255	9957066	chr18	10718653	10871736
6100	CP026246.1	9957337	9966252	chr18	10637063	10718565
6101	CP026246.1	9966421	10004774	chr18	10464479	10636935
6102	CP026246.1	10004885	10009385	chr18	10446271	10463725
6103	CP026246.1	10009548	10046087	chr18	10370479	10446022
6104	CP026246.1	10046182	10234560	chr18	10345728	10370252
6105	CP026246.1	10234976	10274485	chr18	10211329	10345601
6106	CP026246.1	10274610	10355964	chr18	10124622	10209419
6107	CP026246.1	10356146	10432709	chr18	10116720	10124519
6108	CP026246.1	10433579	10634459	chr18	10081627	10116504
6109	CP026246.1	10634754	10717507	chr18	10079261	10080921
6110	CP026246.1	10717725	10767298	chr18	10034720	10079124
6111	CP026246.1	10767631	10918360	chr18	9896453	10034620
6112	CP026246.1	10918916	10980828	chr18	9829461	9896365
6113	CP026246.1	10984284	11140751	chr18	9767491	9828971
6114	CP026246.1	11141788	11182063	chr18	9751663	9767250
6115	CP026246.1	11182170	11313181	chr18	9743037	9750447

6116	CP026246.1	11313578	11449123	chr18	9607315	9742619
6117	CP026246.1	11449340	11478746	chr18	9599341	9606670
6118	CP026246.1	11478985	11548716	chr18	9526663	9598919
6119	CP026246.1	11548934	11567440	chr18	9507837	9526343
6120	CP026246.1	11567760	11640016	chr18	9437888	9507619
6121	CP026246.1	11640438	11647767	chr18	9408243	9437649
6122	CP026246.1	11648412	11783716	chr18	9272481	9408026
6123	CP026246.1	11784134	11791544	chr18	9141073	9272084
6124	CP026246.1	11792760	11808347	chr18	9100691	9140966
6125	CP026246.1	11808588	11870068	chr18	8943187	9099654
6126	CP026246.1	11870558	11937462	chr18	8877819	8939731
6127	CP026246.1	11937550	12075717	chr18	8726534	8877263
6128	CP026246.1	12075817	12120221	chr18	8676628	8726201
6129	CP026246.1	12120358	12122018	chr18	8593657	8676410
6130	CP026246.1	12122724	12157601	chr18	8392482	8593362
6131	CP026246.1	12157817	12165616	chr18	8315049	8391612
6132	CP026246.1	12165719	12250516	chr18	8233513	8314867
6133	CP026246.1	12252426	12386698	chr18	8193879	8233388
6134	CP026246.1	12386825	12411349	chr18	8005085	8193463
6135	CP026246.1	12411576	12487119	chr18	7968451	8004990
6136	CP026246.1	12487368	12504822	chr18	7963788	7968288
6137	CP026246.1	12505576	12678032	chr18	7925324	7963677
6138	CP026246.1	12678160	12759662	chr18	7916240	7925155
6139	CP026246.1	12759750	12912833	chr18	7863158	7915969
6140	CP026246.1	12912966	13010894	chr18	7841245	7862981
6141	CP026246.1	13011808	13105380	chr18	7774877	7841031
6142	CP026246.1	13106482	13191640	chr18	7729176	7774735
6143	CP026246.1	13192968	13221635	chr18	7684100	7725538
6144	CP026246.1	13221931	13223540	chr18	7544700	7683989

6145	CP026246.1	13224762	13230118	chr18	7473045	7544591
6146	CP026246.1	13230268	13288151	chr18	7395008	7472910
6147	CP026246.1	13288360	13328936	chr18	7391418	7394782
6148	CP026246.1	13329232	13357659	chr18	7334092	7391209
6149	CP026246.1	13358875	13368103	chr18	7301886	7333920
6150	CP026246.1	13368232	13591475	chr18	7167239	7301781
6151	CP026246.1	13592077	13679293	chr18	7133665	7167055
6152	CP026246.1	13679829	13685815	chr18	7099504	7133542
6153	CP026246.1	13685960	13730938	chr18	7096666	7098363
6154	CP026246.1	13731129	13770483	chr18	6908821	7096576
6155	CP026246.1	13770889	13800464	chr18	6809848	6908715
6156	CP026246.1	13800564	13838953	chr18	6783046	6809108
6157	CP026246.1	13839416	13967026	chr18	6778458	6782921
6158	CP026246.1	13967380	13987823	chr18	6756864	6778370
6159	CP026246.1	13988208	14070481	chr18	6724131	6756763
6160	CP026246.1	14072241	14096006	chr18	6721837	6723999
6161	CP026246.1	14097256	14244039	chr18	6681224	6721590
6162	CP026246.1	14244286	14264076	chr18	6635877	6681087
6163	CP026246.1	14264202	14316595	chr18	6597282	6635768
6164	CP026246.1	14316763	14412351	chr18	6537286	6597192
6165	CP026246.1	14412452	14454381	chr18	6499255	6537133
6166	CP026246.1	14454507	14458353	chr18	6444730	6499140
6167	CP026246.1	14458841	14479309	chr18	6168000	6444526
6168	CP026246.1	14479488	14536155	chr18	6163680	6167185
6169	CP026246.1	14536442	14761237	chr18	6114792	6163576
6170	CP026246.1	14761447	14779920	chr18	6049357	6114359
6171	CP026246.1	14780133	14912200	chr18	6040379	6049254
6172	CP026246.1	14912411	14915867	chr18	5899930	6040286
6173	CP026246.1	14916016	15063362	chr18	5841469	5899825

6174	CP026246.1	15063467	15111951	chr18	5720844	5840995
6175	CP026246.1	15112466	15299526	chr18	5678661	5720625
6176	CP026246.1	15299643	15311129	chr18	5661587	5677784
6177	CP026246.1	15311315	15371794	chr18	5659718	5661435
6178	CP026246.1	15371945	15403923	chr18	5647695	5659602
6179	CP026246.1	15404303	15435734	chr18	5618733	5647595
6180	CP026246.1	15435897	15519276	chr18	5616920	5618632
6181	CP026246.1	15519730	15606936	chr18	5505094	5616641
6182	CP026246.1	15608120	15617308	chr18	5462619	5504454
6183	CP026246.1	15618133	15619650	chr18	5400711	5462079
6184	CP026246.1	15619736	15686157	chr18	5293877	5400346
6185	CP026246.1	15686554	15691422	chr18	5253101	5293788
6186	CP026246.1	15691541	15780594	chr18	5245207	5252976
6187	CP026246.1	15781569	15853126	chr18	5242204	5244986
6188	CP026246.1	15853987	16019615	chr18	5234717	5241950
6189	CP026246.1	16019722	16128923	chr18	5225420	5234587
6190	CP026246.1	16129038	16168066	chr18	5195511	5225304
6191	CP026246.1	16168173	16183297	chr18	5166424	5195326
6192	CP026246.1	16183437	16236640	chr18	5128679	5166336
6193	CP026246.1	16237124	16314425	chr18	5082202	5128187
6194	CP026246.1	16314511	16369015	chr18	5080228	5082070
6195	CP026246.1	16369128	16464801	chr18	5065448	5079369
6196	CP026246.1	16465014	16503822	chr18	5041478	5064724
6197	CP026246.1	16504919	16558480	chr18	4960553	5041377
6198	CP026246.1	16558620	16570541	chr18	4920375	4960459
6199	CP026246.1	16570778	16621220	chr18	4814012	4919882
6200	CP026246.1	16621382	16664094	chr18	4806598	4813895
6201	CP026246.1	16664224	16949468	chr18	4764042	4806389
6202	CP026246.1	16949599	17007699	chr18	4670751	4763259

6203	CP026246.1	17007821	17036094	chr18	4648879	4670547
6204	CP026246.1	17036266	17076418	chr18	4631798	4648678
6205	CP026246.1	17076730	17077809	chr18	4620977	4631385
6206	CP026246.1	17077946	17097597	chr18	4496020	4620019
6207	CP026246.1	17098838	17131107	chr18	4485280	4495878
6208	CP026246.1	17131226	17132323	chr18	4432858	4484452
6209	CP026246.1	17132435	17187927	chr18	4420721	4432751
6210	CP026246.1	17188201	17301482	chr18	4391458	4420523
6211	CP026246.1	17301591	17358535	chr18	4353850	4390043
6212	CP026246.1	17358636	17423378	chr18	4342202	4352632
6213	CP026246.1	17447473	17454426	chr18	4311826	4342071
6214	CP026246.1	17461336	17465804	chr18	4302099	4311442
6215	CP026246.1	17465914	17496969	chr18	4283560	4301838
6216	CP026246.1	17497073	17541656	chr18	4262514	4283435
6217	CP026246.1	17623134	17701938	chr18	4255464	4262273
6218	CP026246.1	17722169	17750522	chr18	4249727	4255222
6219	CP026246.1	17752319	17768032	chr18	4203528	4249482
6220	CP026246.1	17768133	17882127	chr18	4004808	4203398
6221	CP026246.1	17883089	18064030	chr18	3939252	4003820
6222	CP026246.1	18064133	18270075	chr18	3901349	3939151
6223	CP026246.1	18270899	18295447	chr18	3880849	3900934
6224	CP026246.1	18295563	18507193	chr18	3824514	3880741
6225	CP026246.1	18507777	18542938	chr18	3743625	3823244
6226	CP026246.1	18543176	18597348	chr18	3730799	3743449
6227	CP026246.1	18598113	18680765	chr18	3695235	3730244
6228	CP026246.1	18680977	18789387	chr18	3692130	3695026
6229	CP026246.1	18789556	18872282	chr18	3647121	3691864
6230	CP026246.1	18872542	18885813	chr18	3599079	3646617
6231	CP026246.1	18885925	18891024	chr18	3594748	3597925

6232	CP026246.1	18891397	18916879	chr18	3545865	3594546
6233	CP026246.1	18916969	18953718	chr18	3534685	3545699
6234	CP026246.1	18953813	19158931	chr18	3453919	3532709
6235	CP026246.1	19159141	19287095	chr18	3433936	3453542
6236	CP026246.1	19289045	19352675	chr18	3413633	3433647
6237	CP026246.1	19353279	19370781	chr18	3379181	3412115
6238	CP026246.1	19371087	19445489	chr18	3142524	3146515
6239	CP026246.1	19445822	19505886	chr18	3102154	3110853
6240	CP026246.1	19506241	19654200	chr18	3099901	3102034
6241	CP026246.1	19654538	19759412	chr18	3058082	3099758
6242	CP026246.1	19759702	19810561	chr18	3046249	3057953
6243	CP026246.1	19811259	19879820	chr18	3040374	3045654
6244	CP026246.1	19885186	19902571	chr18	3037059	3038982
6245	CP026246.1	19902672	19936987	chr18	3033598	3036605
6246	CP026246.1	19939058	19985990	chr18	3013250	3033247
6247	CP026246.1	19986204	20198844	chr18	3004969	3012932
6248	CP026246.1	20199991	20394198	chr18	3001890	3004817
6249	CP026246.1	20398680	20640174	chr18	2943125	3001652
6250	CP026246.1	20669002	20687854	chr18	2878272	2942519
6251	CP026246.1	20687955	20705460	chr18	2848089	2878055
6252	CP026246.1	20705560	20707686	chr18	2812230	2847754
6253	CP026246.1	20707902	20896260	chr18	2809820	2811866
6254	CP026246.1	20896405	20905049	chr18	2807581	2809283
6255	CP026246.1	20905259	20935104	chr18	2797814	2807390
6256	CP026246.1	20935212	21051366	chr18	2779145	2797546
6257	CP026246.1	21051459	21078809	chr18	2762138	2778017
6258	CP026246.1	21078926	21322280	chr18	2740953	2762031
6259	CP026246.1	21088459	21089772	chr18	2723767	2740822
6260	CP026246.1	21269972	21273332	chr18	2709248	2722307

6261	CP026246.1	21322494	21433751	chr18	2697712	2707770
6262	CP026246.1	21433934	21514875	chr18	2677343	2697282
6263	CP026246.1	21515403	21525702	chr18	2646032	2677228
6264	CP026246.1	21525903	21560128	chr18	2629798	2645824
6265	CP026246.1	21560278	21568205	chr18	2619507	2628849
6266	CP026246.1	21568307	21574608	chr18	2600423	2619411
6267	CP026246.1	21576261	21607313	chr18	2593534	2600200
6268	CP026246.1	21607496	21663703	chr18	2588579	2591958
6269	CP026246.1	21663975	21673592	chr18	2581159	2587602
6270	CP026246.1	21673803	21682313	chr18	2562506	2580263
6271	CP026246.1	21682471	21715152	chr18	2529916	2562292
6272	CP026246.1	21715512	21732809	chr18	2498858	2529734
6273	CP026246.1	21733000	21766052	chr18	2446603	2498741
6274	CP026246.1	21766378	21809045	chr18	2443523	2445473
6275	CP026246.1	21809815	21831088	chr18	2434285	2442064
6276	CP026246.1	21831428	21886467	chr18	2409887	2434166
6277	CP026246.1	21886727	21931739	chr18	2400262	2409707
6278	CP026246.1	21932525	21972748	chr18	2360409	2400048
6279	CP026246.1	21972885	22026503	chr18	2349493	2359061
6280	CP026246.1	22026604	22042121	chr18	2346842	2348996
6281	CP026246.1	22044812	22071975	chr18	2338148	2346455
6282	CP026246.1	22072193	22080993	chr18	2334706	2336886
6283	CP026246.1	22081488	22097574	chr18	2329377	2334549
6284	CP026246.1	22097739	22115155	chr18	2309054	2329174
6285	CP026246.1	22115263	22206340	chr18	2305498	2308915
6286	CP026246.1	22206492	22266213	chr18	2303348	2305038
6287	CP026246.1	22266402	22344080	chr18	2291320	2303225
6288	CP026246.1	22344231	22476411	chr18	2254869	2290227
6289	CP026246.1	22476690	22535458	chr18	2252816	2254724

6290	CP026246.1	22536016	22573379	chr18	2228737	2252030
6291	CP026246.1	22573843	22623558	chr18	2220224	2227560
6292	CP026246.1	22625863	22679295	chr18	2208888	2219943
6293	CP026246.1	22679568	22708524	chr18	2203812	2208591
6294	CP026246.1	22708877	22964105	chr18	2190444	2203227
6295	CP026246.1	22964425	23062000	chr18	2163322	2188776
6296	CP026246.1	23062092	23108028	chr18	2113641	2163105
6297	CP026246.1	23108183	23121457	chr18	2089949	2111939
6298	CP026246.1	23121579	23137004	chr18	2080725	2088956
6299	CP026246.1	23137964	23183191	chr18	2078902	2080592
6300	CP026246.1	23183704	23190962	chr18	2059626	2078382
6301	CP026246.1	23191183	23210197	chr18	2040946	2059362
6302	CP026246.1	23210321	23215507	chr18	1984357	2040339
6303	CP026246.1	23215599	23256905	chr18	1967566	1984180
6304	CP026246.1	23258486	23296832	chr18	1964886	1967353
6305	CP026246.1	23300935	23302883	chr18	1945392	1964693
6306	CP026246.1	23306658	23367570	chr18	1931594	1945163
6307	CP026246.1	23367708	23389427	chr18	1928685	1931505
6308	CP026246.1	23389627	23500172	chr18	1922059	1928514
6309	CP026246.1	23500824	23702047	chr18	1909984	1921372
6310	CP026246.1	23702560	23789409	chr18	1905529	1909769
6311	CP026246.1	23789586	23828273	chr18	1900543	1902785
6312	CP026246.1	23828542	23843786	chr18	1892908	1900235
6313	CP026246.1	23843898	23848372	chr18	1870650	1891435
6314	CP026246.1	23848606	23860486	chr18	1868265	1870328
6315	CP026246.1	23860584	23886961	chr18	1866091	1868113
6316	CP026246.1	23890405	23934432	chr18	1862831	1865731
6317	CP026246.1	23934687	23944673	chr18	1829668	1859688
6318	CP026246.1	23946268	23967871	chr18	1819542	1829236

6319	CP026246.1	23968060	24004708	chr18	1805803	1819325
6320	CP026246.1	24005509	24068332	chr18	1802506	1803944
6321	CP026246.1	24069067	24084111	chr18	1777758	1802418
6322	CP026246.1	24084414	24174734	chr18	1776254	1777397
6323	CP026246.1	24174983	24254584	chr18	1755893	1774842
6324	CP026246.1	24254693	24281703	chr18	1747913	1755233
6325	CP026246.1	24281868	24390618	chr18	1742555	1747333
6326	CP026246.1	24390824	24422802	chr18	1737103	1742425
6327	CP026246.1	24423959	24562597	chr18	1732709	1736863
6328	CP026246.1	24566139	24609673	chr18	1704620	1731635
6329	CP026246.1	24609886	24621949	chr18	1702742	1704196
6330	CP026246.1	24622071	24629939	chr18	1694019	1702654
6331	CP026246.1	24630340	24656116	chr18	1678480	1693882
6332	CP026246.1	24656210	24719472	chr18	1661722	1678357
6333	CP026246.1	24719634	24734440	chr18	1632667	1661412
6334	CP026246.1	24734541	24792179	chr18	1624169	1632275
6335	CP026246.1	24792383	24818765	chr18	1614421	1624042
6336	CP026246.1	24819613	24843312	chr18	1604302	1613717
6337	CP026246.1	24843550	24897686	chr18	1522180	1604020
6338	CP026246.1	24897803	24951985	chr18	1508842	1521894
6339	CP026246.1	24952086	24984673	chr18	1502870	1508051
6340	CP026246.1	24985020	25005699	chr18	1491028	1502405
6341	CP026246.1	25005814	25187450	chr18	1484760	1490769
6342	CP026246.1	25190679	25244060	chr18	1471779	1484629
6343	CP026246.1	25246728	25310083	chr18	1455350	1471539
6344	CP026246.1	25310244	25623352	chr18	1423338	1455093
6345	CP026246.1	25626459	25761399	chr18	1357420	1421599
6346	CP026246.1	25761500	25812529	chr18	1291524	1356772
6347	CP026246.1	25814355	25911818	chr18	1279356	1290019

6348	CP026246.1	25912099	25972522	chr18	1273602	1278918
6349	CP026246.1	25972623	25975258	chr18	1249320	1273509
6350	CP026246.1	26010568	26053804	chr18	1240057	1249183
6351	CP026246.1	26053913	26193703	chr18	1239956	1236889
6352	CP026246.1	26193812	26285308	chr18	1209650	1208222
6353	CP026246.1	27046319	27071812	chr18	1207064	1196034
6354	CP026246.1	27084402	27124417	chr18	1195943	1184348
6355	CP026246.1	27125292	27145479	chr18	1184253	1140278
6356	CP026246.1	27146163	27238962	chr18	1139267	1137978
6357	CP026246.1	27239071	27349885	chr18	1136970	1132506
6358	CP026246.1	27350179	27376995	chr18	1131943	1127988
6359	CP026246.1	27377135	27440665	chr18	1127575	1035580
6360	CP026246.1	27440789	27611441	chr18	1031820	1011071
6361	CP026246.1	27611665	27654499	chr18	1010618	1008795
6362	CP026246.1	27654710	27658045	chr18	1007805	1003238
6363	CP026246.1	27658258	27661874	chr18	1002617	1000645
6364	CP026246.1	27661995	27671872	chr18	999922	983297
6365	CP026246.1	27672237	27675360	chr18	982859	971986
6366	CP026246.1	27675494	27679359	chr18	971885	970146
6367	CP026246.1	27679955	27683596	chr18	969857	967716
6368	CP026246.1	27684567	27714636	chr18	964412	948571
6369	CP026246.1	27717074	27733594	chr18	948439	943824
6370	CP026246.1	27733777	27756030	chr18	943290	923365
6371	CP026246.1	27756571	27794172	chr18	923173	921561
6372	CP026246.1	27794258	27796066	chr18	921443	896637
6373	CP026246.1	27796153	27802128	chr18	896404	889586
6374	CP026246.1	27802236	27806026	chr18	889409	887931
6375	CP026246.1	27806198	27840473	chr18	887833	874671
6376	CP026246.1	27840716	27895626	chr18	873180	865810

6377	CP026246.1	27895875	27901373	chr18	864992	841075
6378	CP026246.1	27901504	27982039	chr18	837703	822460
6379	CP026246.1	27982135	28030532	chr18	822074	796720
6380	CP026246.1	28031037	28059487	chr18	796575	784245
6381	CP026246.1	28065659	28111392	chr18	774090	784144
6382	CP026246.1	28111605	28117088	chr18	768247	773543
6383	CP026246.1	28117228	28120890	chr18	765590	767293
6384	CP026246.1	28121066	28127265	chr18	762165	764374
6385	CP026246.1	28127379	28138256	chr18	756803	761688
6386	CP026246.1	28138888	28265296	chr18	740751	755926
6387	CP026246.1	28265402	28288145	chr18	732421	738432
6388	CP026246.1	28288966	28399834	chr18	723144	728455
6389	CP026246.1	28400772	28472334	chr18	718905	721637
6390	CP026246.1	28472472	28486571	chr18	716526	718437
6391	CP026246.1	28487673	28538929	chr18	706388	715688
6392	CP026246.1	28539045	28542252	chr18	704176	705760
6393	CP026246.1	28542518	28585891	chr18	698600	703589
6394	CP026246.1	28589074	28661261	chr18	697428	698505
6395	CP026246.1	28662867	28706100	chr18	671126	696884
6396	CP026246.1	28706258	28719823	chr18	658760	666233
6397	CP026246.1	28720611	28755495	chr18	636727	654740
6398	CP026246.1	28755665	28773367	chr18	633317	636583
6399	CP026246.1	28773454	28775395	chr18	618909	632963
6400	CP026246.1	28775654	28786807	chr18	617086	618776
6401	CP026246.1	28786963	28800363	chr18	612986	614855
6402	CP026246.1	28800455	28838413	chr18	591499	609131
6403	CP026246.1	28838619	28841439	chr18	586564	590295
6404	CP026246.1	28841591	28858487	chr18	583369	585281
6405	CP026246.1	28860017	28868012	chr18	581058	582787

6406	CP026246.1	28869844	28893976	chr18	578440	579699
6407	CP026246.1	28894570	28907457	chr18	575969	577821
6408	CP026246.1	28907967	28909502	chr18	568696	575041
6409	CP026246.1	28909654	28971946	chr18	567059	568196
6410	CP026246.1	28973407	28990259	chr18	564673	566920
6411	CP026246.1	28990486	29005896	chr18	559520	562998
6412	CP026246.1	29006053	29029747	chr18	556890	558044
6413	CP026246.1	29029856	29147064	chr18	553396	555200
6414	CP026246.1	29147629	29160564	chr18	541971	551879
6415	CP026246.1	29160733	29255675	chr18	498113	539862
6416	CP026246.1	29255793	29278415	chr18	487007	498012
6417	CP026246.1	29279209	29282369	chr18	478161	485167
6418	CP026246.1	29282560	29294767	chr18	474246	477072
6419	CP026246.1	29294868	29297724	chr18	469711	472436
6420	CP026246.1	29313296	29319093	chr18	467719	469345
6421	CP026246.1	29320159	29321909	chr18	452185	461406
6422	CP026246.1	29324881	29334976	chr18	443280	451663
6423	CP026246.1	29335128	29356704	chr18	434778	443081
6424	CP026246.1	29365437	29441164	chr18	430521	434610
6425	CP026246.1	29443727	29593289	chr18	424076	430207
6426	CP026246.1	29602258	29632790	chr18	416717	423513
6427	CP026246.1	29632891	29654861	chr18	412692	416568
6428	CP026246.1	29655357	29660219	chr18	409427	412260
6429	CP026246.1	29660946	29696240	chr18	403399	409300
6430	CP026246.1	29696595	29712750	chr18	399379	403174
6431	CP026246.1	29713137	29743058	chr18	376572	399089
6432	CP026246.1	29743556	29765354	chr18	335081	376127
6433	CP026246.1	29765507	29778028	chr18	324746	332415
6434	CP026246.1	29778936	29804177	chr18	318133	323574

6435	CP026246.1	29804265	29806802	chr18	313445	315798
6436	CP026246.1	29807804	29809789	chr18	302502	312704
6437	CP026246.1	29810148	29821406	chr18	297093	301992
6438	CP026246.1	29821802	29927314	chr18	285026	295661
6439	CP026246.1	29928297	29931566	chr18	278589	283480
6440	CP026246.1	29932436	29962631	chr18	252077	278160
6441	CP026246.1	29962742	30133617	chr18	244719	251452
6442	CP026246.1	30134338	30142929	chr18	224578	244305
6443	CP026246.1	30143043	30223934	chr18	218203	224457
6444	CP026246.1	30224027	30298714	chr18	215217	217813
6445	CP026246.1	30299526	30336869	chr18	213846	215094
6446	CP026246.1	30337996	30346824	chr18	210181	212713
6447	CP026246.1	30347318	30352014	chr18	198489	209609
6448	CP026246.1	30356679	30447148	chr18	178589	197700
6449	CP026246.1	30447887	30449609	chr18	172328	178462
6450	CP026246.1	30450643	30471455	chr18	166995	172162
6451	CP026246.1	30471560	30504200	chr18	141915	166043
6452	CP026246.1	30504516	30524455	chr18	133306	138390
6453	CP026246.1	30524609	30553263	chr18	113626	130289
6454	CP026246.1	30554136	30577962	chr18	108713	111976
6455	CP026246.1	30578215	30591055	chr18	104266	108602
6456	CP026246.1	30591210	30599829	chr18	91852	100656
6457	CP026246.1	30599928	30603085	chr18	57342	61886
6458	CP026246.1	30604221	30612871	chr18	56118	57241
6459	CP026246.1	30614250	30616795	chr18	49644	54028
6460	CP026246.1	30616927	30640620	chr18	38252	46037
6461	CP026246.1	30641209	30648587	chr18	32595	34599
6462	CP026246.1	30651020	30682333	chr18	28021	32032
6463	CP026246.1	30683211	30821124	chr18	25544	27383

6464	CP026246.1	30821259	30852625	chr18	20518	21838
6465	CP026246.1	30852970	30857680	chr18	12965	16444
6466	CP026246.1	30858100	30869463	chr18	4484	11984
6467	CP026246.1	30869564	30934145	chr18	1	2616
6468	CP026258.1	1	1875	chr19	21650661	21551841
6469	CP026258.1	3212	5135	chr19	21551743	21511061
6470	CP026258.1	6824	9468	chr19	21510934	21481550
6471	CP026258.1	11016	15337	chr19	21481453	21352800
6472	CP026258.1	17872	19622	chr19	21352581	21336008
6473	CP026258.1	21055	25096	chr19	21335897	21275548
6474	CP026258.1	26104	27513	chr19	21275331	21254888
6475	CP026258.1	27779	33722	chr19	21254632	21242895
6476	CP026258.1	35513	38621	chr19	21241172	21235977
6477	CP026258.1	40222	42646	chr19	21235857	21230101
6478	CP026258.1	46042	50359	chr19	21229881	21087970
6479	CP026258.1	50557	59825	chr19	21087695	21015719
6480	CP026258.1	61881	63833	chr19	21015171	20992254
6481	CP026258.1	64859	66258	chr19	20992060	20984905
6482	CP026258.1	66436	78068	chr19	20983821	20894092
6483	CP026258.1	78241	82815	chr19	20893990	20709954
6484	CP026258.1	83326	93532	chr19	20709376	20618829
6485	CP026258.1	96266	102508	chr19	20618699	20578033
6486	CP026258.1	102597	106385	chr19	20577094	20519422
6487	CP026258.1	106643	114490	chr19	20519160	20501936
6488	CP026258.1	115030	121054	chr19	20501759	20348993
6489	CP026258.1	124368	131579	chr19	20345337	20152625
6490	CP026258.1	131923	156829	chr19	20152258	20068174
6491	CP026258.1	156918	182845	chr19	20067408	19813133
6492	CP026258.1	183456	186387	chr19	19804646	19813032

6493	CP026258.1	190599	192609	chr19	19794278	19801323
6494	CP026258.1	192751	205529	chr19	19787728	19793833
6495	CP026258.1	205772	220571	chr19	19775680	19784092
6496	CP026258.1	220683	223540	chr19	19773297	19775240
6497	CP026258.1	224185	249440	chr19	19750066	19772356
6498	CP026258.1	253209	254355	chr19	19704990	19749965
6499	CP026258.1	256051	257692	chr19	19631178	19703887
6500	CP026258.1	259756	266734	chr19	19569312	19607688
6501	CP026258.1	268638	274665	chr19	19569211	19499907
6502	CP026258.1	275444	280822	chr19	19497898	18925781
6503	CP026258.1	281225	285629	chr19	18925191	18780890
6504	CP026258.1	286563	292838	chr19	18780764	18547185
6505	CP026258.1	293184	297152	chr19	18544859	18453124
6506	CP026258.1	301621	303649	chr19	18452925	18451772
6507	CP026258.1	307062	312225	chr19	18451673	18418306
6508	CP026258.1	312474	313832	chr19	18418052	18272513
6509	CP026258.1	315841	317460	chr19	18272357	18210957
6510	CP026258.1	318025	319577	chr19	18210757	18170009
6511	CP026258.1	320629	322449	chr19	18169680	18095886
6512	CP026258.1	323278	328458	chr19	18095147	17989886
6513	CP026258.1	329290	330832	chr19	17986672	17982530
6514	CP026258.1	331686	342339	chr19	17982320	17937398
6515	CP026258.1	342997	350394	chr19	17936939	17930202
6516	CP026258.1	351684	359115	chr19	17929428	17924522
6517	CP026258.1	361061	369255	chr19	17923373	17917309
6518	CP026258.1	370373	374334	chr19	17915564	17892377
6519	CP026258.1	374993	379825	chr19	17890853	17840783
6520	CP026258.1	380625	382734	chr19	17840688	17751119
6521	CP026258.1	386479	388677	chr19	17750496	17737890

6522	CP026258.1	388962	395494	chr19	17737785	17666142
6523	CP026258.1	396812	398054	chr19	17665866	17653185
6524	CP026258.1	398587	400574	chr19	17652859	17636433
6525	CP026258.1	401048	410050	chr19	17636185	17627491
6526	CP026258.1	410930	420381	chr19	17627038	17576482
6527	CP026258.1	420565	426640	chr19	17576284	17547723
6528	CP026258.1	427035	429760	chr19	17547229	17545757
6529	CP026258.1	431183	434894	chr19	17545291	17537734
6530	CP026258.1	434996	513103	chr19	17534380	17516806
6531	CP026258.1	513321	516528	chr19	17515655	17508927
6532	CP026258.1	516629	520304	chr19	17507855	17498737
6533	CP026258.1	520649	522532	chr19	17494844	17487930
6534	CP026258.1	524069	525379	chr19	17486735	17477775
6535	CP026258.1	527124	534053	chr19	17477635	17476093
6536	CP026258.1	534438	549932	chr19	17475645	17474245
6537	CP026258.1	550048	555187	chr19	17472719	17444732
6538	CP026258.1	556766	661864	chr19	17442156	17435386
6539	CP026258.1	661988	682787	chr19	17434327	17426796
6540	CP026258.1	682952	692518	chr19	17424762	17419876
6541	CP026258.1	693701	695769	chr19	17418656	17410652
6542	CP026258.1	696133	698043	chr19	17402848	17410551
6543	CP026258.1	698821	703400	chr19	17394712	17401996
6544	CP026258.1	703739	706093	chr19	17387233	17394256
6545	CP026258.1	707497	725577	chr19	17376371	17385700
6546	CP026258.1	726119	759188	chr19	17365162	17374283
6547	CP026258.1	760675	763194	chr19	17355360	17364170
6548	CP026258.1	763962	804691	chr19	17342029	17351788
6549	CP026258.1	806855	810733	chr19	17336609	17341462
6550	CP026258.1	811693	815813	chr19	17332467	17335858

6551	CP026258.1	816121	830254	chr19	17311132	17330804
6552	CP026258.1	832189	834933	chr19	17308843	17310951
6553	CP026258.1	835169	836641	chr19	17301933	17305306
6554	CP026258.1	837414	841385	chr19	17296963	17300415
6555	CP026258.1	842765	851115	chr19	17283113	17296454
6556	CP026258.1	851519	860594	chr19	17280607	17282790
6557	CP026258.1	860744	864137	chr19	17277123	17279886
6558	CP026258.1	864266	882812	chr19	17273704	17275307
6559	CP026258.1	883539	888245	chr19	17260660	17273446
6560	CP026258.1	888992	945349	chr19	17247458	17259938
6561	CP026258.1	947350	953160	chr19	17243807	17246997
6562	CP026258.1	953446	976376	chr19	17238855	17242379
6563	CP026258.1	972032	973169	chr19	17233799	17238526
6564	CP026258.1	974865	976002	chr19	17222413	17233483
6565	CP026258.1	976635	980649	chr19	17204965	17219608
6566	CP026258.1	981604	985039	chr19	17203026	17204864
6567	CP026258.1	985307	993932	chr19	17196039	17202203
6568	CP026258.1	994461	997852	chr19	17190954	17192581
6569	CP026258.1	998008	999962	chr19	17182359	17189759
6570	CP026258.1	1000945	1005179	chr19	17179695	17180748
6571	CP026258.1	1005470	1009125	chr19	17174517	17179333
6572	CP026258.1	1009450	1019298	chr19	17169903	17174421
6573	CP026258.1	1020508	1028017	chr19	17161404	17169490
6574	CP026258.1	1028318	1034525	chr19	17152494	17154165
6575	CP026258.1	1037456	1040417	chr19	17147803	17150677
6576	CP026258.1	1040842	1050247	chr19	17145237	17147541
6577	CP026258.1	1050685	1066000	chr19	17136823	17144059
6578	CP026258.1	1066895	1068525	chr19	17111116	17117209
6579	CP026258.1	1068698	1071237	chr19	17095232	17111012

6580	CP026258.1	1071377	1085052	chr19	17081131	17094558
6581	CP026258.1	1085163	1090990	chr19	17077187	17080454
6582	CP026258.1	1091278	1098383	chr19	17069131	17076060
6583	CP026258.1	1098631	1106233	chr19	17053223	17066978
6584	CP026258.1	1107768	1127768	chr19	17049007	17052894
6585	CP026258.1	1129996	1132869	chr19	17044786	17046554
6586	CP026258.1	1134740	1136005	chr19	17040150	17042430
6587	CP026258.1	1136429	1139449	chr19	17034831	17038965
6588	CP026258.1	1140193	1142926	chr19	17024581	17034362
6589	CP026258.1	1143135	1146619	chr19	17011768	17024480
6590	CP026258.1	1147475	1189051	chr19	16975860	17006068
6591	CP026258.1	1189275	1212673	chr19	16938881	16975759
6592	CP026258.1	1213590	1219886	chr19	16911069	16937748
6593	CP026258.1	1220002	1223335	chr19	16906019	16909834
6594	CP026258.1	1223441	1226655	chr19	16728459	16905179
6595	CP026258.1	1226991	1245754	chr19	16715399	16727474
6596	CP026258.1	1245967	1249159	chr19	16702135	16711971
6597	CP026258.1	1249463	1280793	chr19	16689159	16702027
6598	CP026258.1	1280884	1291948	chr19	16669261	16689001
6599	CP026258.1	1292433	1302965	chr19	16627406	16669111
6600	CP026258.1	1303325	1325861	chr19	16529470	16625957
6601	CP026258.1	1326222	1337502	chr19	16525586	16529044
6602	CP026258.1	1338899	1346292	chr19	16520260	16525384
6603	CP026258.1	1346416	1356721	chr19	16490682	16519743
6604	CP026258.1	1360659	1366843	chr19	16407293	16490546
6605	CP026258.1	1368748	1374155	chr19	16296047	16407157
6606	CP026258.1	1377764	1380936	chr19	16290159	16295844
6607	CP026258.1	1381047	1383248	chr19	16190604	16289944
6608	CP026258.1	1384609	1386886	chr19	16056435	16190474

6609	CP026258.1	1389475	1396121	chr19	16036181	16056289
6610	CP026258.1	1396235	1421981	chr19	15818241	16035592
6611	CP026258.1	1423458	1428654	chr19	15798600	15817325
6612	CP026258.1	1428868	1449017	chr19	15746001	15798423
6613	CP026258.1	1449205	1454811	chr19	15721516	15745908
6614	CP026258.1	1456369	1520784	chr19	15678028	15721191
6615	CP026258.1	1521073	1558606	chr19	15630200	15677936
6616	CP026258.1	1558814	1563154	chr19	15593057	15629631
6617	CP026258.1	1563827	1567793	chr19	15586529	15592945
6618	CP026258.1	1568520	1621206	chr19	15455188	15586252
6619	CP026258.1	1622418	1639362	chr19	15274606	15454788
6620	CP026258.1	1640986	1643757	chr19	15065076	15274503
6621	CP026258.1	1643886	1645885	chr19	15045130	15064332
6622	CP026258.1	1646882	1651888	chr19	15038948	15044703
6623	CP026258.1	1652537	1660522	chr19	14996741	15037716
6624	CP026258.1	1661713	1689853	chr19	14981912	14996542
6625	CP026258.1	1691075	1698566	chr19	14826906	14981517
6626	CP026258.1	1699065	1725252	chr19	14590475	14826190
6627	CP026258.1	1725957	1731876	chr19	14544437	14590363
6628	CP026258.1	1731988	1741288	chr19	14501171	14544326
6629	CP026258.1	1741920	1744042	chr19	14488285	14501072
6630	CP026258.1	1744359	1760198	chr19	14389917	14487903
6631	CP026258.1	1762277	1781101	chr19	14370504	14389779
6632	CP026258.1	1781245	1786617	chr19	14354744	14370083
6633	CP026258.1	1786705	1806358	chr19	14234020	14354562
6634	CP026258.1	1806572	1840361	chr19	14169261	14233917
6635	CP026258.1	1840533	1888921	chr19	14115558	14169131
6636	CP026258.1	1890042	1906925	chr19	14102234	14115408
6637	CP026258.1	1907059	1984335	chr19	14042519	14101798

6638	CP026258.1	1984792	1986733	chr19	13984882	14042414
6639	CP026258.1	1987106	1996070	chr19	13982510	13984604
6640	CP026258.1	1996967	2008836	chr19	13943442	13982401
6641	CP026258.1	2008937	2022943	chr19	13813691	13943281
6642	CP026258.1	2023044	2027358	chr19	13782035	13811359
6643	CP026258.1	2027631	2033857	chr19	13658705	13780892
6644	CP026258.1	2035532	2037332	chr19	13654416	13658289
6645	CP026258.1	2038568	2050747	chr19	13619213	13654189
6646	CP026258.1	2051050	2054463	chr19	13588975	13617767
6647	CP026258.1	2054572	2065852	chr19	13566767	13588468
6648	CP026258.1	2069612	2071781	chr19	13475252	13566608
6649	CP026258.1	2075649	2076964	chr19	13443992	13471224
6650	CP026258.1	2081188	2086124	chr19	13437895	13443586
6651	CP026258.1	2087630	2094834	chr19	13323111	13437808
6652	CP026258.1	2095307	2096904	chr19	13266947	13322939
6653	CP026258.1	2097993	2101813	chr19	13155704	13266726
6654	CP026258.1	2102142	2114365	chr19	13144439	13155510
6655	CP026258.1	2114491	2116062	chr19	13077885	13143956
6656	CP026258.1	2116282	2121766	chr19	12965609	13076579
6657	CP026258.1	2122083	2123378	chr19	12951049	12965463
6658	CP026258.1	2124238	2157816	chr19	12922714	12950842
6659	CP026258.1	2158309	2163885	chr19	12870375	12922077
6660	CP026258.1	2164250	2176029	chr19	12628388	12870031
6661	CP026258.1	2176256	2195828	chr19	12370650	12627503
6662	CP026258.1	2195949	2223354	chr19	12326996	12370520
6663	CP026258.1	2223516	2286334	chr19	12275217	12326906
6664	CP026258.1	2286532	2314584	chr19	12234756	12275012
6665	CP026258.1	2315485	2317235	chr19	12190835	12234633
6666	CP026258.1	2318563	2375175	chr19	12097693	12190655

6667	CP026258.1	2375588	2379351	chr19	11988398	12097171
6668	CP026258.1	2379546	2477573	chr19	11972350	11988175
6669	CP026258.1	2477689	2478917	chr19	11961925	11972245
6670	CP026258.1	2479112	2491175	chr19	11935464	11961818
6671	CP026258.1	2491792	2505536	chr19	11764784	11935346
6672	CP026258.1	2505759	2507392	chr19	11708026	11764682
6673	CP026258.1	2507763	2516902	chr19	11577731	11706248
6674	CP026258.1	2516998	2527857	chr19	11445509	11577632
6675	CP026258.1	2528364	2532933	chr19	11424907	11444731
6676	CP026258.1	2533303	2536859	chr19	11307389	11424694
6677	CP026258.1	2537350	2592123	chr19	11296662	11306303
6678	CP026258.1	2592298	2623586	chr19	11274137	11296470
6679	CP026258.1	2623752	2639089	chr19	11234649	11273956
6680	CP026258.1	2640782	2729536	chr19	11159250	11232738
6681	CP026258.1	2730133	2756471	chr19	11078380	11158851
6682	CP026258.1	2756699	2899450	chr19	10803267	11078184
6683	CP026258.1	2900199	2934712	chr19	10801885	10803166
6684	CP026258.1	2935767	2958668	chr19	10617108	10801729
6685	CP026258.1	2959318	2964764	chr19	10611839	10616855
6686	CP026258.1	2965331	2966570	chr19	10600987	10611733
6687	CP026258.1	2967070	2977712	chr19	10532579	10600296
6688	CP026258.1	2978028	2989252	chr19	10358526	10532434
6689	CP026258.1	2989728	3027192	chr19	10342591	10358326
6690	CP026258.1	3029332	3037282	chr19	10286984	10342450
6691	CP026258.1	3037395	3051839	chr19	10247358	10286855
6692	CP026258.1	3052025	3104637	chr19	10135541	10247195
6693	CP026258.1	3104889	3133155	chr19	10071472	10135318
6694	CP026258.1	3133277	3144497	chr19	10067488	10071354
6695	CP026258.1	3144743	3165093	chr19	10052199	10067400

6696	CP026258.1	3165471	3189740	chr19	10034459	10052106
6697	CP026258.1	3189932	3215550	chr19	9896405	10034314
6698	CP026258.1	3216108	3235277	chr19	9870089	9896177
6699	CP026258.1	3235367	3238040	chr19	9855836	9869828
6700	CP026258.1	3238171	3279922	chr19	9705595	9855647
6701	CP026258.1	3280053	3282947	chr19	9685346	9705430
6702	CP026258.1	3283649	3291415	chr19	9677373	9685141
6703	CP026258.1	3293224	3314597	chr19	9673961	9677267
6704	CP026258.1	3315719	3330886	chr19	9664690	9673605
6705	CP026258.1	3332166	3363801	chr19	9615649	9664508
6706	CP026258.1	3364093	3401706	chr19	9517652	9614541
6707	CP026258.1	3401822	3406488	chr19	9416462	9516828
6708	CP026258.1	3406830	3411139	chr19	9204069	9416329
6709	CP026258.1	3412260	3421659	chr19	9158263	9203930
6710	CP026258.1	3422006	3430740	chr19	9067884	9158137
6711	CP026258.1	3433059	3437640	chr19	8876600	9067022
6712	CP026258.1	3437932	3444391	chr19	8722789	8876343
6713	CP026258.1	3444584	3453616	chr19	8630089	8722279
6714	CP026258.1	3457894	3463081	chr19	8590503	8628838
6715	CP026258.1	3468446	3476512	chr19	8560633	8590394
6716	CP026258.1	3476955	3484833	chr19	8534524	8560487
6717	CP026258.1	3484931	3495474	chr19	8522923	8533698
6718	CP026258.1	3495748	3497028	chr19	8362887	8522641
6719	CP026258.1	3497886	3501311	chr19	8348076	8362726
6720	CP026258.1	3502991	3504521	chr19	8290065	8347916
6721	CP026258.1	3505270	3525414	chr19	8235398	8289398
6722	CP026258.1	3525586	3535405	chr19	8229246	8234552
6723	CP026258.1	3536315	3558745	chr19	8194150	8229144
6724	CP026258.1	3558951	3589161	chr19	8174175	8194050

6725	CP026258.1	3589523	3693572	chr19	8170590	8174066
6726	CP026258.1	3693714	3710404	chr19	7984018	8170292
6727	CP026258.1	3710557	3736875	chr19	7980792	7983866
6728	CP026258.1	3737112	3904887	chr19	7977324	7980422
6729	CP026258.1	3905498	3939577	chr19	7949569	7977233
6730	CP026258.1	3939766	3945342	chr19	7833251	7949389
6731	CP026258.1	3945958	4011131	chr19	7740035	7833157
6732	CP026258.1	4011418	4013343	chr19	7723438	7739916
6733	CP026258.1	4013715	4026983	chr19	7713222	7721907
6734	CP026258.1	4027620	4033610	chr19	7687061	7712760
6735	CP026258.1	4034363	4044828	chr19	7684485	7685685
6736	CP026258.1	4044933	4194609	chr19	7647301	7684323
6737	CP026258.1	4194738	4299419	chr19	7643709	7646699
6738	CP026258.1	4299534	4305722	chr19	7636004	7643313
6739	CP026258.1	4305903	4317180	chr19	7564094	7635489
6740	CP026258.1	4317962	4343299	chr19	7500350	7562759
6741	CP026258.1	4343412	4367934	chr19	7488157	7500194
6742	CP026258.1	4368061	4403960	chr19	7404250	7486985
6743	CP026258.1	4404278	4467590	chr19	7318555	7403950
6744	CP026258.1	4467800	4503549	chr19	7314410	7317868
6745	CP026258.1	4503978	4519517	chr19	6827984	6876955
6746	CP026258.1	4519615	4526129	chr19	6776210	6826825
6747	CP026258.1	4527048	4565406	chr19	6758597	6776018
6748	CP026258.1	4566058	4579861	chr19	6742429	6757148
6749	CP026258.1	4580037	4589747	chr19	6587889	6742127
6750	CP026258.1	4590578	4594385	chr19	6496855	6587638
6751	CP026258.1	4594541	4604673	chr19	6434356	6496710
6752	CP026258.1	4604861	4615580	chr19	6417428	6434252
6753	CP026258.1	4615934	4631668	chr19	6311983	6417093

6754	CP026258.1	4631882	4633394	chr19	6300187	6311864
6755	CP026258.1	4633635	4641310	chr19	6286075	6300101
6756	CP026258.1	4642271	4651804	chr19	6275838	6285984
6757	CP026258.1	4651933	4658567	chr19	6257694	6275737
6758	CP026258.1	4658780	4664550	chr19	6156496	6256581
6759	CP026258.1	4665770	4710022	chr19	6109769	6156284
6760	CP026258.1	4710237	4720982	chr19	6060301	6109662
6761	CP026258.1	4721103	4744577	chr19	5995988	6060115
6762	CP026258.1	4745351	4771062	chr19	5987347	5995606
6763	CP026258.1	4772076	4774240	chr19	5979015	5987225
6764	CP026258.1	4774336	4810075	chr19	5967914	5978499
6765	CP026258.1	4810274	4820615	chr19	5964884	5967368
6766	CP026258.1	4820932	4859932	chr19	5936685	5964796
6767	CP026258.1	4860327	4883174	chr19	5907417	5936215
6768	CP026258.1	4883484	4892072	chr19	5802732	5907290
6769	CP026258.1	4892442	4959170	chr19	5717079	5802624
6770	CP026258.1	4959330	4991247	chr19	5672760	5716955
6771	CP026258.1	4992392	5016614	chr19	5665527	5672605
6772	CP026258.1	5016962	5026265	chr19	5649002	5665184
6773	CP026258.1	5026361	5030711	chr19	5605961	5648892
6774	CP026258.1	5030828	5059376	chr19	5527828	5605403
6775	CP026258.1	5059915	5135661	chr19	5475218	5527571
6776	CP026258.1	5136105	5154300	chr19	5397394	5475113
6777	CP026258.1	5154397	5166797	chr19	5392829	5397297
6778	CP026258.1	5166949	5199703	chr19	5355100	5392507
6779	CP026258.1	5200390	5225962	chr19	5337358	5354814
6780	CP026258.1	5226090	5243722	chr19	5333687	5337083
6781	CP026258.1	5243947	5268305	chr19	5330326	5333599
6782	CP026258.1	5268420	5312425	chr19	5326613	5330019

6783	CP026258.1	5312522	5326780	chr19	5311973	5326231
6784	CP026258.1	5327162	5330568	chr19	5267871	5311876
6785	CP026258.1	5330875	5334148	chr19	5243398	5267756
6786	CP026258.1	5334236	5337632	chr19	5225541	5243173
6787	CP026258.1	5337907	5355363	chr19	5199841	5225413
6788	CP026258.1	5355649	5393056	chr19	5166400	5199154
6789	CP026258.1	5393378	5397846	chr19	5153848	5166248
6790	CP026258.1	5397943	5475662	chr19	5135556	5153751
6791	CP026258.1	5475767	5528120	chr19	5059366	5135112
6792	CP026258.1	5528377	5605952	chr19	5030279	5058827
6793	CP026258.1	5606510	5649441	chr19	5025812	5030162
6794	CP026258.1	5649551	5665733	chr19	5016413	5025716
6795	CP026258.1	5666076	5673154	chr19	4991843	5016065
6796	CP026258.1	5673309	5717504	chr19	4958781	4990698
6797	CP026258.1	5717628	5803173	chr19	4891893	4958621
6798	CP026258.1	5803281	5907839	chr19	4882935	4891523
6799	CP026258.1	5907966	5936764	chr19	4859778	4882625
6800	CP026258.1	5937234	5965345	chr19	4820383	4859383
6801	CP026258.1	5965433	5967917	chr19	4809725	4820066
6802	CP026258.1	5968463	5979048	chr19	4773787	4809526
6803	CP026258.1	5979564	5987774	chr19	4771527	4773691
6804	CP026258.1	5987896	5996155	chr19	4744802	4770513
6805	CP026258.1	5996537	6060664	chr19	4720554	4744028
6806	CP026258.1	6060850	6110211	chr19	4709688	4720433
6807	CP026258.1	6110318	6156833	chr19	4665221	4709473
6808	CP026258.1	6157045	6257130	chr19	4658231	4664001
6809	CP026258.1	6258243	6276286	chr19	4651384	4658018
6810	CP026258.1	6276387	6286533	chr19	4641722	4651255
6811	CP026258.1	6286624	6300650	chr19	4633086	4640761

6812	CP026258.1	6300736	6312413	chr19	4631333	4632845
6813	CP026258.1	6312532	6417642	chr19	4615385	4631119
6814	CP026258.1	6417977	6434801	chr19	4604312	4615031
6815	CP026258.1	6434905	6497259	chr19	4593992	4604124
6816	CP026258.1	6497404	6588187	chr19	4590029	4593836
6817	CP026258.1	6588438	6742676	chr19	4579488	4589198
6818	CP026258.1	6742978	6757697	chr19	4565509	4579312
6819	CP026258.1	6759146	6776567	chr19	4526499	4564857
6820	CP026258.1	6776759	6827374	chr19	4519066	4525580
6821	CP026258.1	6828533	6877504	chr19	4503429	4518968
6822	CP026258.1	6877605	6881063	chr19	4467251	4503000
6823	CP026258.1	6881750	6967145	chr19	4403729	4467041
6824	CP026258.1	6967445	7050180	chr19	4367512	4403411
6825	CP026258.1	7051352	7063389	chr19	4342863	4367385
6826	CP026258.1	7063545	7125954	chr19	4317413	4342750
6827	CP026258.1	7127289	7198684	chr19	4305354	4316631
6828	CP026258.1	7199199	7206508	chr19	4298985	4305173
6829	CP026258.1	7206904	7209894	chr19	4194189	4298870
6830	CP026258.1	7210496	7247518	chr19	4044384	4194060
6831	CP026258.1	7247680	7248880	chr19	4033814	4044279
6832	CP026258.1	7250256	7275955	chr19	4027071	4033061
6833	CP026258.1	7276417	7285102	chr19	4013166	4026434
6834	CP026258.1	7286633	7303111	chr19	4010869	4012794
6835	CP026258.1	7303230	7396352	chr19	3945409	4010582
6836	CP026258.1	7396446	7512584	chr19	3939217	3944793
6837	CP026258.1	7512764	7540428	chr19	3904949	3939028
6838	CP026258.1	7540519	7543617	chr19	3736563	3904338
6839	CP026258.1	7543987	7547061	chr19	3710008	3736326
6840	CP026258.1	7547213	7733487	chr19	3693165	3709855

6841	CP026258.1	7733785	7737261	chr19	3588974	3693023
6842	CP026258.1	7737370	7757245	chr19	3558402	3588612
6843	CP026258.1	7757345	7792339	chr19	3535766	3558196
6844	CP026258.1	7792441	7797747	chr19	3525037	3534856
6845	CP026258.1	7798593	7852593	chr19	3504721	3524865
6846	CP026258.1	7853260	7911111	chr19	3502442	3503972
6847	CP026258.1	7911271	7925921	chr19	3497337	3500762
6848	CP026258.1	7926082	8085836	chr19	3495199	3496479
6849	CP026258.1	8086118	8096893	chr19	3484382	3494925
6850	CP026258.1	8097719	8123682	chr19	3476406	3484284
6851	CP026258.1	8123828	8153589	chr19	3467897	3475963
6852	CP026258.1	8153698	8192033	chr19	3457345	3462532
6853	CP026258.1	8193284	8285474	chr19	3444035	3453067
6854	CP026258.1	8285984	8439538	chr19	3437383	3443842
6855	CP026258.1	8439795	8630217	chr19	3432510	3437091
6856	CP026258.1	8631079	8721332	chr19	3421457	3430191
6857	CP026258.1	8721458	8767125	chr19	3411711	3421110
6858	CP026258.1	8767264	8979524	chr19	3406281	3410590
6859	CP026258.1	8979657	9080023	chr19	3401273	3405939
6860	CP026258.1	9080847	9177736	chr19	3363544	3401157
6861	CP026258.1	9178844	9227703	chr19	3331617	3363252
6862	CP026258.1	9227885	9236800	chr19	3315170	3330337
6863	CP026258.1	9237156	9240462	chr19	3292675	3314048
6864	CP026258.1	9240568	9248336	chr19	3283100	3290866
6865	CP026258.1	9248541	9268625	chr19	3279504	3282398
6866	CP026258.1	9268790	9418842	chr19	3237622	3279373
6867	CP026258.1	9419031	9433023	chr19	3234818	3237491
6868	CP026258.1	9433284	9459372	chr19	3215559	3234728
6869	CP026258.1	9459600	9597509	chr19	3189383	3215001

6870	CP026258.1	9597654	9615301	chr19	3164922	3189191
6871	CP026258.1	9615394	9630595	chr19	3144194	3164544
6872	CP026258.1	9630683	9634549	chr19	3132728	3143948
6873	CP026258.1	9634667	9698513	chr19	3104340	3132606
6874	CP026258.1	9698736	9810390	chr19	3051476	3104088
6875	CP026258.1	9810553	9850050	chr19	3036846	3051290
6876	CP026258.1	9850179	9905645	chr19	3028783	3036733
6877	CP026258.1	9905786	9921521	chr19	2989179	3026643
6878	CP026258.1	9921721	10095629	chr19	2977479	2988703
6879	CP026258.1	10095774	10163491	chr19	2966521	2977163
6880	CP026258.1	10164182	10174928	chr19	2964782	2966021
6881	CP026258.1	10175034	10180050	chr19	2958769	2964215
6882	CP026258.1	10180303	10364924	chr19	2935218	2958119
6883	CP026258.1	10365080	10366361	chr19	2899650	2934163
6884	CP026258.1	10366462	10641379	chr19	2756150	2898901
6885	CP026258.1	10641575	10722046	chr19	2729584	2755922
6886	CP026258.1	10722445	10795933	chr19	2640233	2728987
6887	CP026258.1	10797844	10837151	chr19	2623203	2638540
6888	CP026258.1	10837332	10859665	chr19	2591749	2623037
6889	CP026258.1	10859857	10869498	chr19	2536801	2591574
6890	CP026258.1	10870584	10987889	chr19	2532754	2536310
6891	CP026258.1	10988102	11007926	chr19	2527815	2532384
6892	CP026258.1	11008704	11140827	chr19	2516449	2527308
6893	CP026258.1	11140926	11269443	chr19	2507214	2516353
6894	CP026258.1	11271221	11327877	chr19	2505210	2506843
6895	CP026258.1	11327979	11498541	chr19	2491243	2504987
6896	CP026258.1	11498659	11525013	chr19	2478563	2490626
6897	CP026258.1	11525120	11535440	chr19	2477140	2478368
6898	CP026258.1	11535545	11551370	chr19	2378997	2477024

6899	CP026258.1	11551593	11660366	chr19	2375039	2378802
6900	CP026258.1	11660888	11753850	chr19	2318014	2374626
6901	CP026258.1	11754030	11797828	chr19	2314936	2316686
6902	CP026258.1	11797951	11838207	chr19	2285983	2314035
6903	CP026258.1	11838412	11890101	chr19	2222967	2285785
6904	CP026258.1	11890191	11933715	chr19	2195400	2222805
6905	CP026258.1	11933845	12190698	chr19	2175707	2195279
6906	CP026258.1	12191583	12433226	chr19	2163701	2175480
6907	CP026258.1	12433570	12485272	chr19	2157760	2163336
6908	CP026258.1	12485909	12514037	chr19	2123689	2157267
6909	CP026258.1	12514244	12528658	chr19	2121534	2122829
6910	CP026258.1	12528804	12639774	chr19	2115733	2121217
6911	CP026258.1	12641080	12707151	chr19	2113942	2115513
6912	CP026258.1	12707634	12718705	chr19	2101593	2113816
6913	CP026258.1	12718899	12829921	chr19	2097444	2101264
6914	CP026258.1	12830142	12886134	chr19	2094758	2096355
6915	CP026258.1	12886306	13001003	chr19	2087081	2094285
6916	CP026258.1	13001090	13006781	chr19	2080639	2085575
6917	CP026258.1	13007187	13034419	chr19	2075100	2076415
6918	CP026258.1	13038447	13129803	chr19	2069063	2071232
6919	CP026258.1	13129962	13151663	chr19	2054023	2065303
6920	CP026258.1	13152170	13180962	chr19	2050501	2053914
6921	CP026258.1	13182408	13217384	chr19	2038019	2050198
6922	CP026258.1	13217611	13221484	chr19	2034983	2036783
6923	CP026258.1	13221900	13344087	chr19	2027082	2033308
6924	CP026258.1	13345230	13374554	chr19	2022495	2026809
6925	CP026258.1	13376886	13506476	chr19	2008388	2022394
6926	CP026258.1	13506637	13545596	chr19	1996418	2008287
6927	CP026258.1	13545705	13547799	chr19	1986557	1995521

6928	CP026258.1	13548077	13605609	chr19	1984243	1986184
6929	CP026258.1	13605714	13664993	chr19	1906510	1983786
6930	CP026258.1	13665429	13678603	chr19	1889493	1906376
6931	CP026258.1	13678753	13732326	chr19	1839984	1888372
6932	CP026258.1	13732456	13797112	chr19	1806023	1839812
6933	CP026258.1	13797215	13917757	chr19	1786156	1805809
6934	CP026258.1	13917939	13933278	chr19	1780696	1786068
6935	CP026258.1	13933699	13952974	chr19	1761728	1780552
6936	CP026258.1	13953112	14051098	chr19	1743810	1759649
6937	CP026258.1	14051480	14064267	chr19	1741371	1743493
6938	CP026258.1	14064366	14107521	chr19	1731439	1740739
6939	CP026258.1	14107632	14153558	chr19	1725408	1731327
6940	CP026258.1	14153670	14389385	chr19	1698516	1724703
6941	CP026258.1	14390101	14544712	chr19	1690526	1698017
6942	CP026258.1	14545107	14559737	chr19	1661164	1689304
6943	CP026258.1	14559936	14600911	chr19	1651988	1659973
6944	CP026258.1	14602143	14607898	chr19	1646333	1651339
6945	CP026258.1	14608325	14627527	chr19	1643337	1645336
6946	CP026258.1	14628271	14837698	chr19	1640437	1643208
6947	CP026258.1	14837801	15017983	chr19	1621869	1638813
6948	CP026258.1	15018383	15149447	chr19	1567971	1620657
6949	CP026258.1	15149724	15156140	chr19	1563278	1567244
6950	CP026258.1	15156252	15192826	chr19	1558265	1562605
6951	CP026258.1	15193395	15241131	chr19	1520524	1558057
6952	CP026258.1	15241223	15284386	chr19	1455820	1520235
6953	CP026258.1	15284711	15309103	chr19	1448656	1454262
6954	CP026258.1	15309196	15361618	chr19	1428319	1448468
6955	CP026258.1	15361795	15380520	chr19	1422909	1428105
6956	CP026258.1	15381436	15598787	chr19	1395686	1421432

6957	CP026258.1	15599376	15619484	chr19	1388926	1395572
6958	CP026258.1	15619630	15753669	chr19	1384060	1386337
6959	CP026258.1	15753799	15853139	chr19	1380498	1382699
6960	CP026258.1	15853354	15859039	chr19	1377215	1380387
6961	CP026258.1	15859242	15970352	chr19	1368199	1373606
6962	CP026258.1	15970488	16053741	chr19	1360110	1366294
6963	CP026258.1	16053877	16082938	chr19	1345867	1356172
6964	CP026258.1	16083455	16088579	chr19	1338350	1345743
6965	CP026258.1	16088781	16092239	chr19	1325673	1336953
6966	CP026258.1	16092665	16189152	chr19	1302776	1325312
6967	CP026258.1	16190601	16232306	chr19	1291884	1302416
6968	CP026258.1	16232456	16252196	chr19	1280335	1291399
6969	CP026258.1	16252354	16265222	chr19	1248914	1280244
6970	CP026258.1	16265330	16275166	chr19	1245418	1248610
6971	CP026258.1	16278594	16290669	chr19	1226442	1245205
6972	CP026258.1	16291654	16468374	chr19	1222892	1226106
6973	CP026258.1	16469214	16473029	chr19	1219453	1222786
6974	CP026258.1	16474264	16500943	chr19	1213041	1219337
6975	CP026258.1	16502076	16538954	chr19	1188726	1212124
6976	CP026258.1	16610488	16709308	chr19	1146926	1188502
6977	CP026258.1	16709406	16750088	chr19	1142586	1146070
6978	CP026258.1	16750215	16779599	chr19	1139644	1142377
6979	CP026258.1	16779696	16908349	chr19	1135880	1138900
6980	CP026258.1	16908568	16925141	chr19	1134191	1135456
6981	CP026258.1	16925252	16985601	chr19	1129447	1132320
6982	CP026258.1	16985818	17006261	chr19	1107219	1127219
6983	CP026258.1	17006517	17018254	chr19	1098082	1105684
6984	CP026258.1	17019977	17025172	chr19	1090729	1097834
6985	CP026258.1	17025292	17031048	chr19	1084614	1090441

6986	CP026258.1	17031268	17173179	chr19	1070828	1084503
6987	CP026258.1	17173454	17245430	chr19	1068149	1070688
6988	CP026258.1	17245978	17268895	chr19	1066346	1067976
6989	CP026258.1	17269089	17276244	chr19	1050136	1065451
6990	CP026258.1	17277328	17367057	chr19	1040293	1049698
6991	CP026258.1	17367159	17551195	chr19	1036907	1039868
6992	CP026258.1	17551773	17642320	chr19	1027769	1033976
6993	CP026258.1	17642450	17683116	chr19	1019959	1027468
6994	CP026258.1	17684055	17741727	chr19	1008901	1018749
6995	CP026258.1	17741989	17759213	chr19	1004921	1008576
6996	CP026258.1	17759390	17912156	chr19	1000396	1004630
6997	CP026258.1	17915812	18108524	chr19	997459	999413
6998	CP026258.1	18108891	18192975	chr19	993912	997303
6999	CP026258.1	18193741	18448016	chr19	984758	993383
7000	CP026258.1	18448117	18478325	chr19	981055	984490
7001	CP026258.1	18484025	18496737	chr19	976086	980100
7002	CP026258.1	18587562	18625938	chr19	975453	974316
7003	CP026258.1	18649428	18722137	chr19	972620	971483
7004	CP026258.1	18723240	18768215	chr19	952897	975827
7005	CP026258.1	18769173	18838477	chr19	946801	952611
7006	CP026258.1	18840486	19412603	chr19	888443	944800
7007	CP026258.1	19413193	19557494	chr19	882990	887696
7008	CP026258.1	19557620	19791199	chr19	863717	882263
7009	CP026258.1	19793525	19885260	chr19	860195	863588
7010	CP026258.1	19885459	19886612	chr19	850970	860045
7011	CP026258.1	19886711	19920078	chr19	842216	850566
7012	CP026258.1	19920332	20065871	chr19	836865	840836
7013	CP026258.1	20066027	20127427	chr19	834620	836092
7014	CP026258.1	20127627	20168375	chr19	831640	834384

7015	CP026258.1	20168704	20242498	chr19	815572	829705
7016	CP026258.1	20243237	20348498	chr19	811144	815264
7017	CP026258.1	20351712	20355854	chr19	806306	810184
7018	CP026258.1	20356064	20400986	chr19	763413	804142
7019	CP026258.1	20401445	20408182	chr19	760126	762645
7020	CP026258.1	20408956	20413862	chr19	725570	758639
7021	CP026258.1	20415011	20421075	chr19	706948	725028
7022	CP026258.1	20422820	20446007	chr19	703190	705544
7023	CP026258.1	20447531	20497601	chr19	698272	702851
7024	CP026258.1	20497696	20587265	chr19	695584	697494
7025	CP026258.1	20587888	20600494	chr19	693152	695220
7026	CP026258.1	20600599	20672242	chr19	682403	691969
7027	CP026258.1	20672518	20685199	chr19	661439	682238
7028	CP026258.1	20685525	20701951	chr19	556217	661315
7029	CP026258.1	20702199	20710893	chr19	549499	554638
7030	CP026258.1	20711346	20761902	chr19	533889	549383
7031	CP026258.1	20762100	20790661	chr19	526575	533504
7032	CP026258.1	20791155	20792627	chr19	523520	524830
7033	CP026258.1	20793093	20800650	chr19	520100	521983
7034	CP026258.1	20804004	20821578	chr19	516080	519755
7035	CP026258.1	20822729	20829457	chr19	512772	515979
7036	CP026258.1	20830529	20839647	chr19	434447	512554
7037	CP026258.1	20843540	20850454	chr19	430634	434345
7038	CP026258.1	20851649	20860609	chr19	426486	429211
7039	CP026258.1	20860749	20862291	chr19	420016	426091
7040	CP026258.1	20862739	20864139	chr19	410381	419832
7041	CP026258.1	20865665	20893652	chr19	400499	409501
7042	CP026258.1	20896228	20902998	chr19	398038	400025
7043	CP026258.1	20904057	20911588	chr19	396263	397505

7044	CP026258.1	20913622	20918508	chr19	388413	394945
7045	CP026258.1	20919728	20927732	chr19	385930	388128
7046	CP026258.1	21003468	21018111	chr19	380076	382185
7047	CP026258.1	21020916	21031986	chr19	374444	379276
7048	CP026258.1	21032302	21037029	chr19	369824	373785
7049	CP026258.1	21037358	21040882	chr19	360512	368706
7050	CP026258.1	21042310	21045500	chr19	351135	358566
7051	CP026258.1	21045961	21058441	chr19	342448	349845
7052	CP026258.1	21059163	21071949	chr19	331137	341790
7053	CP026258.1	21072207	21073810	chr19	328741	330283
7054	CP026258.1	21075626	21078389	chr19	322729	327909
7055	CP026258.1	21079110	21081293	chr19	320080	321900
7056	CP026258.1	21081616	21094957	chr19	317476	319028
7057	CP026258.1	21095466	21098918	chr19	315292	316911
7058	CP026258.1	21100436	21103809	chr19	311925	313283
7059	CP026258.1	21107346	21109454	chr19	306513	311676
7060	CP026258.1	21109635	21129307	chr19	301072	303100
7061	CP026258.1	21130970	21134361	chr19	292635	296603
7062	CP026258.1	21135112	21139965	chr19	286014	292289
7063	CP026258.1	21140532	21150291	chr19	280676	285080
7064	CP026258.1	21153863	21162673	chr19	274895	280273
7065	CP026258.1	21163665	21172786	chr19	268089	274116
7066	CP026258.1	21174874	21184203	chr19	259207	266185
7067	CP026258.1	21185736	21192759	chr19	255502	257143
7068	CP026258.1	21193215	21200499	chr19	252660	253806
7069	CP026258.1	21201351	21209054	chr19	252559	250685
7070	CP026258.1	21209155	21231445	chr19	249348	247425
7071	CP026258.1	21232386	21234329	chr19	245736	243092
7072	CP026258.1	21234769	21243181	chr19	241544	237223

7073	CP026258.1	21246817	21252922	chr19	234688	232938
7074	CP026258.1	21253367	21260412	chr19	231505	227464
7075	CP026258.1	21263735	21272121	chr19	226456	225047
7076	CP026258.1	21272222	21282003	chr19	224781	218838
7077	CP026258.1	21282472	21286606	chr19	217047	213939
7078	CP026258.1	21287791	21290071	chr19	212338	209914
7079	CP026258.1	21292427	21294195	chr19	206518	202201
7080	CP026258.1	21296648	21300535	chr19	202003	192735
7081	CP026258.1	21300864	21314619	chr19	190679	188727
7082	CP026258.1	21316772	21323701	chr19	187701	186302
7083	CP026258.1	21324828	21328095	chr19	186124	174492
7084	CP026258.1	21328772	21342199	chr19	174319	169745
7085	CP026258.1	21342873	21358653	chr19	169234	159028
7086	CP026258.1	21358757	21364850	chr19	156294	150052
7087	CP026258.1	21384464	21391700	chr19	149963	146175
7088	CP026258.1	21392878	21395182	chr19	145917	138070
7089	CP026258.1	21395444	21398318	chr19	137530	131506
7090	CP026258.1	21400135	21401806	chr19	128192	120981
7091	CP026258.1	21409045	21417131	chr19	120637	95731
7092	CP026258.1	21417544	21422062	chr19	95642	69715
7093	CP026258.1	21422158	21426974	chr19	69104	66173
7094	CP026258.1	21427336	21428389	chr19	61961	59951
7095	CP026258.1	21430000	21437400	chr19	59809	47031
7096	CP026258.1	21438595	21440222	chr19	46788	31989
7097	CP026258.1	21443680	21449844	chr19	31877	29020
7098	CP026258.1	21450667	21452505	chr19	28375	3120
7099	CP026259.1	13086635	13188120	chr20	101486	1
7100	CP026259.1	13063999	13072217	chr20	124122	115904
7101	CP026259.1	12894427	13009498	chr20	293694	178623

7102	CP026259.1	12784155	12804906	chr20	403966	383215
7103	CP026259.1	12672877	12748228	chr20	515244	439893
7104	CP026259.1	12506800	12639710	chr20	681321	548411
7105	CP026259.1	12174741	12180558	chr20	1013380	1007563
7106	CP026259.1	12096236	12138444	chr20	1091885	1049677
7107	CP026259.1	11983346	12032943	chr20	1204775	1155178
7108	CP026259.1	11889744	11948555	chr20	1298377	1239566
7109	CP026259.1	11719319	11752281	chr20	1468802	1435840
7110	CP026259.1	11622200	11634292	chr20	1565921	1553829
7111	CP026259.1	11409932	11484207	chr20	1778189	1703914
7112	CP026259.1	11280213	11363401	chr20	1907908	1824720
7113	CP026259.1	10885941	11124332	chr20	2302180	2063789
7114	CP026259.1	10807324	10815567	chr20	2380797	2372554
7115	CP026259.1	10687608	10734450	chr20	2500513	2453671
7116	CP026259.1	10464917	10549525	chr20	2723204	2638596
7117	CP026259.1	10302471	10332106	chr20	2885650	2856015
7118	CP026259.1	10244924	10251492	chr20	2943197	2936629
7119	CP026259.1	10206356	10241556	chr20	2981765	2946565
7120	CP026259.1	10039908	10138313	chr20	3148213	3049808
7121	CP026259.1	9877750	9903676	chr20	3310371	3284445
7122	CP026259.1	9819314	9820633	chr20	3368807	3367488
7123	CP026259.1	9393902	9708492	chr20	3794219	3479629
7124	CP026259.1	9081518	9249202	chr20	4106603	3938919
7125	CP026259.1	9008014	9012657	chr20	4180107	4175464
7126	CP026259.1	8915721	8992381	chr20	4272400	4195740
7127	CP026259.1	8727549	8852907	chr20	4460572	4335214
7128	CP026259.1	8623778	8657401	chr20	4564343	4530720
7129	CP026259.1	8449475	8580319	chr20	4738646	4607802
7130	CP026259.1	8328158	8404170	chr20	4859963	4783951

7131	CP026259.1	8280374	8291408	chr20	4907747	4896713
7132	CP026259.1	8235396	8250436	chr20	4952725	4937685
7133	CP026259.1	8206145	8211328	chr20	4981976	4976793
7134	CP026259.1	8004027	8029918	chr20	5184094	5158203
7135	CP026259.1	7975893	7977532	chr20	5212228	5210589
7136	CP026259.1	7949437	7975792	chr20	5238684	5212329
7137	CP026259.1	7705439	7812170	chr20	5482682	5375951
7138	CP026259.1	7508157	7520561	chr20	5679964	5667560
7139	CP026259.1	7212865	7470524	chr20	5975256	5717597
7140	CP026259.1	7032534	7063333	chr20	6155587	6124788
7141	CP026259.1	6952066	6974642	chr20	6236055	6213479
7142	CP026259.1	6768828	6839091	chr20	6419293	6349030
7143	CP026259.1	6748760	6768741	chr20	6439361	6419380
7144	CP026259.1	6695316	6705854	chr20	6492805	6482267
7145	CP026259.1	6466243	6489985	chr20	6721878	6698136
7146	CP026259.1	6363751	6417357	chr20	6824370	6770764
7147	CP026259.1	6310945	6318390	chr20	6877176	6869731
7148	CP026259.1	6264423	6272285	chr20	6923698	6915836
7149	CP026259.1	6195794	6239547	chr20	6992327	6948574
7150	CP026259.1	6154517	6160726	chr20	7033604	7027395
7151	CP026259.1	6148785	6151235	chr20	7039336	7036886
7152	CP026259.1	6122901	6144343	chr20	7065220	7043778
7153	CP026259.1	6073678	6076102	chr20	7114443	7112019
7154	CP026259.1	6062357	6073589	chr20	7125764	7114532
7155	CP026259.1	6046703	6057270	chr20	7141418	7130851
7156	CP026259.1	5985962	6011212	chr20	7202159	7176909
7157	CP026259.1	5916781	5923230	chr20	7271340	7264891
7158	CP026259.1	5850624	5869142	chr20	7337497	7318979
7159	CP026259.1	5808169	5811726	chr20	7379952	7376395

7160	CP026259.1	5791671	5793335	chr20	7396450	7394786
7161	CP026259.1	5738221	5748425	chr20	7449900	7439696
7162	CP026259.1	5729843	5732414	chr20	7458278	7455707
7163	CP026259.1	5721403	5722234	chr20	7466718	7465887
7164	CP026259.1	5679133	5684715	chr20	7508988	7503406
7165	CP026259.1	5621384	5627851	chr20	7566737	7560270
7166	CP026259.1	5559164	5569661	chr20	7628957	7618460
7167	CP026259.1	5485168	5493080	chr20	7702953	7695041
7168	CP026259.1	5415858	5425779	chr20	7772263	7762342
7169	CP026259.1	5403118	5407318	chr20	7785003	7780803
7170	CP026259.1	5359920	5367886	chr20	7828201	7820235
7171	CP026259.1	5347626	5349350	chr20	7840495	7838771
7172	CP026259.1	5321130	5322655	chr20	7866991	7865466
7173	CP026259.1	5288786	5304916	chr20	7899335	7883205
7174	CP026259.1	5221268	5288693	chr20	7966853	7899428
7175	CP026259.1	5105179	5127240	chr20	8082942	8060881
7176	CP026259.1	5100474	5104834	chr20	8087647	8083287
7177	CP026259.1	5082148	5100007	chr20	8105607	8087748
7178	CP026259.1	5081368	5082019	chr20	8106387	8105736
7179	CP026259.1	5066352	5081211	chr20	8121403	8106544
7180	CP026259.1	4978478	5065048	chr20	8209277	8122707
7181	CP026259.1	4960842	4978293	chr20	8226913	8209462
7182	CP026259.1	4620101	4960733	chr20	8567654	8227022
7183	CP026259.1	4593234	4619904	chr20	8594521	8567851
7184	CP026259.1	4586918	4593019	chr20	8600837	8594736
7185	CP026259.1	4518164	4586728	chr20	8669591	8601027
7186	CP026259.1	4513201	4517770	chr20	8674554	8669985
7187	CP026259.1	4486804	4513002	chr20	8700951	8674753
7188	CP026259.1	4481873	4486685	chr20	8705882	8701070

7189	CP026259.1	4471099	4480705	chr20	8716656	8707050
7190	CP026259.1	4467543	4470818	chr20	8720212	8716937
7191	CP026259.1	4436090	4467232	chr20	8751665	8720523
7192	CP026259.1	4424838	4435974	chr20	8762917	8751781
7193	CP026259.1	4342742	4424690	chr20	8845013	8763065
7194	CP026259.1	4336172	4342378	chr20	8851583	8845377
7195	CP026259.1	4331554	4335750	chr20	8856201	8852005
7196	CP026259.1	4295736	4331446	chr20	8892019	8856309
7197	CP026259.1	4291168	4295405	chr20	8896587	8892350
7198	CP026259.1	4281951	4290282	chr20	8905804	8897473
7199	CP026259.1	4280074	4281783	chr20	8907681	8905972
7200	CP026259.1	4255638	4261727	chr20	8932117	8926028
7201	CP026259.1	4236676	4242037	chr20	8951079	8945718
7202	CP026259.1	4216205	4219386	chr20	8971550	8968369
7203	CP026259.1	4185264	4187312	chr20	9002491	9000443
7204	CP026259.1	4182885	4185167	chr20	9004870	9002588
7205	CP026259.1	4140060	4167974	chr20	9047695	9019781
7206	CP026259.1	4120690	4125563	chr20	9067065	9062192
7207	CP026259.1	4058468	4091714	chr20	9129287	9096041
7208	CP026259.1	4045811	4050824	chr20	9141944	9136931
7209	CP026259.1	4016243	4019407	chr20	9171512	9168348
7210	CP026259.1	3970300	3974640	chr20	9217455	9213115
7211	CP026259.1	3939029	3942965	chr20	9248726	9244790
7212	CP026259.1	3855673	3918146	chr20	9332082	9269609
7213	CP026259.1	3807801	3816411	chr20	9379954	9371344
7214	CP026259.1	3749798	3783588	chr20	9437957	9404167
7215	CP026259.1	3678636	3742997	chr20	9509119	9444758
7216	CP026259.1	3645000	3662746	chr20	9542755	9525009
7217	CP026259.1	3621713	3644899	chr20	9566042	9542856

7218	CP026259.1	3563070	3602001	chr20	9624685	9585754
7219	CP026259.1	3529508	3557059	chr20	9658247	9630696
7220	CP026259.1	3496266	3508149	chr20	9691489	9679606
7221	CP026259.1	3423461	3483289	chr20	9764294	9704466
7222	CP026259.1	3401534	3417092	chr20	9786221	9770663
7223	CP026259.1	3386906	3395796	chr20	9800849	9791959
7224	CP026259.1	3360044	3362679	chr20	9827711	9825076
7225	CP026259.1	3218189	3262632	chr20	9969566	9925123
7226	CP026259.1	3163751	3195752	chr20	10024004	9992003
7227	CP026259.1	3033576	3064102	chr20	10154179	10123653
7228	CP026259.1	3023424	3028579	chr20	10164331	10159176
7229	CP026259.1	2986366	3018568	chr20	10201389	10169187
7230	CP026259.1	2962071	2974617	chr20	10225684	10213138
7231	CP026259.1	2914229	2953677	chr20	10273526	10234078
7232	CP026259.1	2893869	2907071	chr20	10293886	10280684
7233	CP026259.1	2847921	2888213	chr20	10339834	10299542
7234	CP026259.1	2777445	2801740	chr20	10410310	10386015
7235	CP026259.1	2534877	2698910	chr20	10652878	10488845
7236	CP026259.1	2445679	2494281	chr20	10742076	10693474
7237	CP026259.1	2386325	2389652	chr20	10801430	10798103
7238	CP026259.1	2303381	2317264	chr20	10884374	10870491
7239	CP026259.1	2206644	2213583	chr20	10981111	10974172
7240	CP026259.1	2137915	2156427	chr20	11049840	11031328
7241	CP026259.1	2125036	2128506	chr20	11062719	11059249
7242	CP026259.1	2054691	2088001	chr20	11133064	11099754
7243	CP026259.1	2045996	2049402	chr20	11141759	11138353
7244	CP026259.1	2026616	2032079	chr20	11161139	11155676
7245	CP026259.1	1955248	1964374	chr20	11232507	11223381
7246	CP026259.1	1933542	1935163	chr20	11254213	11252592

7247	CP026259.1	1898727	1907917	chr20	11289028	11279838
7248	CP026259.1	1895749	1898627	chr20	11292006	11289128
7249	CP026259.1	1873452	1890457	chr20	11314303	11297298
7250	CP026259.1	1862690	1868458	chr20	11325065	11319297
7251	CP026259.1	1847486	1852911	chr20	11340269	11334844
7252	CP026259.1	1839113	1839557	chr20	11348642	11348198
7253	CP026259.1	1821034	1824617	chr20	11366721	11363138
7254	CP026259.1	1796663	1809389	chr20	11391092	11378366
7255	CP026259.1	1773218	1774612	chr20	11414537	11413143
7256	CP026259.1	1755559	1759394	chr20	11432196	11428361
7257	CP026259.1	1748138	1749921	chr20	11439617	11437834
7258	CP026259.1	1697901	1714166	chr20	11489854	11473589
7259	CP026259.1	1647553	1680139	chr20	11540202	11507616
7260	CP026259.1	1626400	1638175	chr20	11561355	11549580
7261	CP026259.1	1576688	1579373	chr20	11611067	11608382
7262	CP026259.1	1550374	1557064	chr20	11637381	11630691
7263	CP026259.1	1494269	1525753	chr20	11693486	11662002
7264	CP026259.1	1390254	1455993	chr20	11797501	11731762
7265	CP026259.1	1351528	1353720	chr20	11836227	11834035
7266	CP026259.1	1327173	1337097	chr20	11860582	11850658
7267	CP026259.1	1300412	1314614	chr20	11887343	11873141
7268	CP026259.1	1279311	1286565	chr20	11908444	11901190
7269	CP026259.1	1263264	1268683	chr20	11924491	11919072
7270	CP026259.1	1251814	1257133	chr20	11935941	11930622
7271	CP026259.1	1241663	1245847	chr20	11946092	11941908
7272	CP026259.1	1229057	1230926	chr20	11958698	11956829
7273	CP026259.1	1210897	1213764	chr20	11976858	11973991
7274	CP026259.1	1145871	1177871	chr20	12041884	12009884
7275	CP026259.1	1114788	1140186	chr20	12072967	12047569

7276	CP026259.1	1095791	1103814	chr20	12091964	12083941
7277	CP026259.1	1087634	1090131	chr20	12100121	12097624
7278	CP026259.1	1078165	1079603	chr20	12109590	12108152
7279	CP026259.1	1047281	1056052	chr20	12140474	12131703
7280	CP026259.1	1022035	1027097	chr20	12165720	12160658
7281	CP026259.1	984346	1010949	chr20	12203409	12176806
7282	CP026259.1	932884	935043	chr20	12254871	12252712
7283	CP026259.1	912493	932783	chr20	12275262	12254972
7284	CP026259.1	901742	902168	chr20	12286013	12285587
7285	CP026259.1	868517	875869	chr20	12319238	12311886
7286	CP026259.1	858625	861945	chr20	12329130	12325810
7287	CP026259.1	848565	852308	chr20	12339190	12335447
7288	CP026259.1	845446	847317	chr20	12342309	12340438
7289	CP026259.1	821632	824981	chr20	12345759	12342410
7290	CP026259.1	818191	820992	chr20	12349200	12346399
7291	CP026259.1	813245	817883	chr20	12354146	12349508
7292	CP026259.1	786485	812483	chr20	12380906	12354908
7293	CP026259.1	784153	785996	chr20	12383238	12381395
7294	CP026259.1	774145	782112	chr20	12393246	12385279
7295	CP026259.1	771870	773140	chr20	12395521	12394251
7296	CP026259.1	764716	768393	chr20	12402675	12398998
7297	CP026259.1	758984	762978	chr20	12408407	12404413
7298	CP026259.1	754521	758584	chr20	12412870	12408807
7299	CP026259.1	749749	754390	chr20	12417642	12413001
7300	CP026259.1	746736	748611	chr20	12420655	12418780
7301	CP026259.1	743144	745185	chr20	12424247	12422206
7302	CP026259.1	741385	742892	chr20	12426006	12424499
7303	CP026259.1	738395	740422	chr20	12428996	12426969
7304	CP026259.1	733889	737270	chr20	12433502	12430121

7305	CP026259.1	729078	733456	chr20	12438313	12433935
7306	CP026259.1	723041	728264	chr20	12444350	12439127
7307	CP026259.1	720020	722529	chr20	12447371	12444862
7308	CP026259.1	717459	719109	chr20	12449932	12448282
7309	CP026259.1	706935	717303	chr20	12460456	12450088
7310	CP026259.1	697234	706846	chr20	12470157	12460545
7311	CP026259.1	645275	645555	chr20	12494727	12494447
7312	CP026259.1	644542	644822	chr20	12495460	12495180
7313	CP026243.1	31900011	31901587	chr21	1577	1
7314	CP026243.1	31897271	31898492	chr21	4317	3096
7315	CP026243.1	31892160	31895217	chr21	9428	6371
7316	CP026243.1	31886098	31890348	chr21	15490	11240
7317	CP026243.1	31869859	31885529	chr21	31729	16059
7318	CP026243.1	31866309	31869088	chr21	35279	32500
7319	CP026243.1	31864871	31865788	chr21	36717	35800
7320	CP026243.1	31856224	31864773	chr21	45364	36815
7321	CP026243.1	31853872	31855310	chr21	47716	46278
7322	CP026243.1	31848361	31850882	chr21	53227	50706
7323	CP026243.1	31806556	31848243	chr21	95032	53345
7324	CP026243.1	31794719	31805065	chr21	106869	96523
7325	CP026243.1	31792076	31794472	chr21	109512	107116
7326	CP026243.1	31783979	31791931	chr21	117609	109657
7327	CP026243.1	31773244	31783682	chr21	128344	117906
7328	CP026243.1	31769742	31772679	chr21	131846	128909
7329	CP026243.1	31766449	31767592	chr21	135139	133996
7330	CP026243.1	31752377	31765681	chr21	149211	135907
7331	CP026243.1	31711880	31750941	chr21	189708	150647
7332	CP026243.1	31697747	31710592	chr21	203841	190996
7333	CP026243.1	31656358	31697632	chr21	245230	203956

7334	CP026243.1	31647324	31655773	chr21	254264	245815
7335	CP026243.1	31636337	31646882	chr21	265251	254706
7336	CP026243.1	31619589	31635198	chr21	281999	266390
7337	CP026243.1	31615355	31619089	chr21	286233	282499
7338	CP026243.1	31606862	31614368	chr21	294726	287220
7339	CP026243.1	31390410	31606763	chr21	511178	294825
7340	CP026243.1	31364161	31390313	chr21	537427	511275
7341	CP026243.1	31309180	31363392	chr21	592408	538196
7342	CP026243.1	31307150	31309001	chr21	594438	592587
7343	CP026243.1	31305572	31306927	chr21	596016	594661
7344	CP026243.1	31290585	31305077	chr21	611003	596511
7345	CP026243.1	31280532	31290446	chr21	621056	611142
7346	CP026243.1	31274172	31280443	chr21	627416	621145
7347	CP026243.1	31268909	31273503	chr21	632679	628085
7348	CP026243.1	31264414	31267698	chr21	637174	633890
7349	CP026243.1	31260906	31264161	chr21	640682	637427
7350	CP026243.1	31245272	31260570	chr21	656316	641018
7351	CP026243.1	31189407	31244963	chr21	712181	656625
7352	CP026243.1	31181377	31189194	chr21	720211	712394
7353	CP026243.1	31145880	31181117	chr21	755708	720471
7354	CP026243.1	31143736	31145738	chr21	757852	755850
7355	CP026243.1	31141279	31143628	chr21	760309	757960
7356	CP026243.1	31139261	31140781	chr21	762327	760807
7357	CP026243.1	31132257	31138032	chr21	769331	763556
7358	CP026243.1	31123989	31132124	chr21	777599	769464
7359	CP026243.1	31108837	31123608	chr21	792751	777980
7360	CP026243.1	31068762	31108531	chr21	832826	793057
7361	CP026243.1	31047385	31068617	chr21	854203	832971
7362	CP026243.1	31042235	31046415	chr21	859353	855173

7363	CP026243.1	31036184	31040159	chr21	865404	861429
7364	CP026243.1	31030979	31035064	chr21	870609	866524
7365	CP026243.1	31024228	31030474	chr21	877360	871114
7366	CP026243.1	31020160	31022207	chr21	881428	879381
7367	CP026243.1	30999024	31020070	chr21	902564	881518
7368	CP026243.1	30992464	30998551	chr21	909124	903037
7369	CP026243.1	30986593	30990902	chr21	914995	910686
7370	CP026243.1	30974860	30986177	chr21	926728	915411
7371	CP026243.1	30972367	30974398	chr21	929221	927190
7372	CP026243.1	30938061	30972119	chr21	963527	929469
7373	CP026243.1	30924674	30937222	chr21	976914	964366
7374	CP026243.1	30912108	30924456	chr21	989480	977132
7375	CP026243.1	30884162	30911944	chr21	1017426	989644
7376	CP026243.1	30867698	30883799	chr21	1033890	1017789
7377	CP026243.1	30856985	30867559	chr21	1044603	1034029
7378	CP026243.1	30851457	30856840	chr21	1050131	1044748
7379	CP026243.1	30833118	30847695	chr21	1068470	1053893
7380	CP026243.1	30826049	30832822	chr21	1075539	1068766
7381	CP026243.1	30817893	30824740	chr21	1083695	1076848
7382	CP026243.1	30812384	30817431	chr21	1089204	1084157
7383	CP026243.1	30788027	30812179	chr21	1113561	1089409
7384	CP026243.1	30758085	30787930	chr21	1143503	1113658
7385	CP026243.1	30679151	30757813	chr21	1222437	1143775
7386	CP026243.1	30677166	30678172	chr21	1224422	1223416
7387	CP026243.1	30669810	30677079	chr21	1231778	1224509
7388	CP026243.1	30642819	30668963	chr21	1258769	1232625
7389	CP026243.1	30633065	30642271	chr21	1268523	1259317
7390	CP026243.1	30630533	30631619	chr21	1271055	1269969
7391	CP026243.1	30624148	30628716	chr21	1277440	1272872

7392	CP026243.1	30621233	30623451	chr21	1280355	1278137
7393	CP026243.1	30612129	30620772	chr21	1289459	1280816
7394	CP026243.1	30606146	30610864	chr21	1295442	1290724
7395	CP026243.1	30595871	30605516	chr21	1305717	1296072
7396	CP026243.1	30593395	30595532	chr21	1308193	1306056
7397	CP026243.1	30586329	30592872	chr21	1315259	1308716
7398	CP026243.1	30583647	30585831	chr21	1317941	1315757
7399	CP026243.1	30578626	30583462	chr21	1322962	1318126
7400	CP026243.1	30574426	30577863	chr21	1327162	1323725
7401	CP026243.1	30571994	30574164	chr21	1329594	1327424
7402	CP026243.1	30569837	30571288	chr21	1331751	1330300
7403	CP026243.1	30566533	30567906	chr21	1335055	1333682
7404	CP026243.1	30550588	30559581	chr21	1351000	1342007
7405	CP026243.1	30547598	30550417	chr21	1353990	1351171
7406	CP026243.1	30541226	30547106	chr21	1360362	1354482
7407	CP026243.1	30534769	30539412	chr21	1366819	1362176
7408	CP026243.1	30512426	30534605	chr21	1389162	1366983
7409	CP026243.1	30483110	30511787	chr21	1418478	1389801
7410	CP026243.1	30482632	30482936	chr21	1418956	1418652
7411	CP026243.1	30450220	30482385	chr21	1451368	1419203
7412	CP026243.1	30447540	30449221	chr21	1454048	1452367
7413	CP026243.1	30430519	30445924	chr21	1471069	1455664
7414	CP026243.1	30424758	30426490	chr21	1476830	1475098
7415	CP026243.1	30410328	30423864	chr21	1491260	1477724
7416	CP026243.1	30382041	30408833	chr21	1519547	1492755
7417	CP026243.1	30378695	30381698	chr21	1522893	1519890
7418	CP026243.1	30370166	30373369	chr21	1531422	1528219
7419	CP026243.1	30360676	30369984	chr21	1540912	1531604
7420	CP026243.1	30359528	30360421	chr21	1542060	1541167

7421	CP026243.1	30356735	30358939	chr21	1544853	1542649
7422	CP026243.1	30350612	30353943	chr21	1550976	1547645
7423	CP026243.1	30343999	30348353	chr21	1557589	1553235
7424	CP026243.1	30322245	30343775	chr21	1579343	1557813
7425	CP026243.1	30317616	30319617	chr21	1583972	1581971
7426	CP026243.1	30309751	30317407	chr21	1591837	1584181
7427	CP026243.1	30305184	30309325	chr21	1596404	1592263
7428	CP026243.1	30302948	30305089	chr21	1598640	1596499
7429	CP026243.1	30271116	30302840	chr21	1630472	1598748
7430	CP026243.1	30262271	30270808	chr21	1639317	1630780
7431	CP026243.1	30205476	30260996	chr21	1696112	1640592
7432	CP026243.1	30078116	30184993	chr21	1823472	1716595
7433	CP026243.1	30074719	30076881	chr21	1826869	1824707
7434	CP026243.1	30063514	30072804	chr21	1838074	1828784
7435	CP026243.1	29401922	29402590	chr21	2497734	2497066
7436	CP026243.1	29386574	29401815	chr21	2513082	2497841
7437	CP026243.1	29347790	29386338	chr21	2551866	2513318
7438	CP026243.1	29221475	29347682	chr21	2678181	2551974
7439	CP026243.1	29211553	29219843	chr21	2688103	2679813
7440	CP026243.1	29210433	29211424	chr21	2689223	2688232
7441	CP026243.1	29207055	29210330	chr21	2692601	2689326
7442	CP026243.1	29187706	29190647	chr21	2711950	2709009
7443	CP026243.1	29107151	29187541	chr21	2792505	2712115
7444	CP026243.1	29075542	29106568	chr21	2824114	2793088
7445	CP026243.1	29071875	29075260	chr21	2827781	2824396
7446	CP026243.1	29053085	29071573	chr21	2846571	2828083
7447	CP026243.1	29050465	29051871	chr21	2849191	2847785
7448	CP026243.1	29038309	29050290	chr21	2861347	2849366
7449	CP026243.1	29032944	29038214	chr21	2866712	2861442

7450	CP026243.1	28993592	29032832	chr21	2906064	2866824
7451	CP026243.1	28976309	28980290	chr21	2923347	2919366
7452	CP026243.1	28967468	28976109	chr21	2932188	2923547
7453	CP026243.1	28958155	28967155	chr21	2941501	2932501
7454	CP026243.1	28923895	28957920	chr21	2975761	2941736
7455	CP026243.1	28900606	28910582	chr21	2999050	2989074
7456	CP026243.1	28896147	28900499	chr21	3003509	2999157
7457	CP026243.1	28884997	28895857	chr21	3014659	3003799
7458	CP026243.1	28842728	28884852	chr21	3056928	3014804
7459	CP026243.1	28839641	28842639	chr21	3060015	3057017
7460	CP026243.1	28803318	28839496	chr21	3096338	3060160
7461	CP026243.1	28780963	28799702	chr21	3118693	3099954
7462	CP026243.1	28769411	28778540	chr21	3130245	3121116
7463	CP026243.1	28761203	28768381	chr21	3138453	3131275
7464	CP026243.1	28756038	28758300	chr21	3143618	3141356
7465	CP026243.1	28740223	28755779	chr21	3159433	3143877
7466	CP026243.1	28730345	28739501	chr21	3169311	3160155
7467	CP026243.1	28721621	28730207	chr21	3178035	3169449
7468	CP026243.1	28706306	28718608	chr21	3193350	3181048
7469	CP026243.1	28697157	28706119	chr21	3202499	3193537
7470	CP026243.1	28693809	28697014	chr21	3205847	3202642
7471	CP026243.1	28692340	28692827	chr21	3207316	3206829
7472	CP026243.1	28633775	28645460	chr21	3265881	3254196
7473	CP026243.1	28618712	28630062	chr21	3280944	3269594
7474	CP026243.1	28593656	28618540	chr21	3306000	3281116
7475	CP026243.1	28591058	28593544	chr21	3308598	3306112
7476	CP026243.1	28585462	28590919	chr21	3314194	3308737
7477	CP026243.1	28581020	28585376	chr21	3318636	3314280
7478	CP026243.1	28579076	28580922	chr21	3320580	3318734

7479	CP026243.1	28572310	28578921	chr21	3327346	3320735
7480	CP026243.1	28563770	28572079	chr21	3335886	3327577
7481	CP026243.1	28539949	28562563	chr21	3359707	3337093
7482	CP026243.1	28533130	28536741	chr21	3366526	3362915
7483	CP026243.1	28508561	28532107	chr21	3391095	3367549
7484	CP026243.1	28503167	28508397	chr21	3396489	3391259
7485	CP026243.1	28485817	28494311	chr21	3413839	3405345
7486	CP026243.1	28453728	28463144	chr21	3445928	3436512
7487	CP026243.1	28435909	28451420	chr21	3463747	3448236
7488	CP026243.1	28430860	28435607	chr21	3468796	3464049
7489	CP026243.1	28413423	28430734	chr21	3486233	3468922
7490	CP026243.1	28412095	28413311	chr21	3487561	3486345
7491	CP026243.1	28407187	28411853	chr21	3492469	3487803
7492	CP026243.1	28389316	28395070	chr21	3510340	3504586
7493	CP026243.1	28321290	28389159	chr21	3578366	3510497
7494	CP026243.1	28289464	28321134	chr21	3610192	3578522
7495	CP026243.1	28288222	28289340	chr21	3611434	3610316
7496	CP026243.1	28286418	28288120	chr21	3613238	3611536
7497	CP026243.1	28281533	28284246	chr21	3618123	3615410
7498	CP026243.1	28240173	28259876	chr21	3659483	3639780
7499	CP026243.1	28200524	28239952	chr21	3699132	3659704
7500	CP026243.1	28179570	28195269	chr21	3720086	3704387
7501	CP026243.1	28173401	28178422	chr21	3726255	3721234
7502	CP026243.1	28154797	28173090	chr21	3744859	3726566
7503	CP026243.1	28150926	28153122	chr21	3748730	3746534
7504	CP026243.1	28119838	28147915	chr21	3779818	3751741
7505	CP026243.1	28003243	28036566	chr21	3896413	3863090
7506	CP026243.1	27877406	27898198	chr21	4022250	4001458
7507	CP026243.1	27824175	27866515	chr21	4075481	4033141

7508	CP026243.1	27805878	27824018	chr21	4093778	4075638
7509	CP026243.1	27708689	27802577	chr21	4190967	4097079
7510	CP026243.1	27702227	27708499	chr21	4197429	4191157
7511	CP026243.1	27658391	27702113	chr21	4241265	4197543
7512	CP026243.1	27594892	27643065	chr21	4304764	4256591
7513	CP026243.1	27593427	27594781	chr21	4306229	4304875
7514	CP026243.1	27586519	27587483	chr21	4307294	4306330
7515	CP026243.1	27570712	27575396	chr21	4323101	4318417
7516	CP026243.1	27551778	27570588	chr21	4342035	4323225
7517	CP026243.1	27549476	27551690	chr21	4344337	4342123
7518	CP026243.1	27430805	27549188	chr21	4463008	4344625
7519	CP026243.1	27429014	27430502	chr21	4464799	4463311
7520	CP026243.1	27426448	27428389	chr21	4467365	4465424
7521	CP026243.1	27403448	27426279	chr21	4490365	4467534
7522	CP026243.1	27400692	27402756	chr21	4493121	4491057
7523	CP026243.1	27395216	27400313	chr21	4498597	4493500
7524	CP026243.1	27389120	27392650	chr21	4504693	4501163
7525	CP026243.1	27336238	27388929	chr21	4557575	4504884
7526	CP026243.1	27304393	27336004	chr21	4589420	4557809
7527	CP026243.1	27302174	27303967	chr21	4591639	4589846
7528	CP026243.1	27240284	27301797	chr21	4653529	4592016
7529	CP026243.1	27227044	27240119	chr21	4666769	4653694
7530	CP026243.1	27224874	27226954	chr21	4668939	4666859
7531	CP026243.1	27223194	27224772	chr21	4670619	4669041
7532	CP026243.1	27196170	27223062	chr21	4697643	4670751
7533	CP026243.1	27187329	27195963	chr21	4706484	4697850
7534	CP026243.1	27172353	27185312	chr21	4721460	4708501
7535	CP026243.1	27145015	27172032	chr21	4748798	4721781
7536	CP026243.1	27127576	27144863	chr21	4766237	4748950

7537	CP026243.1	27119730	27127350	chr21	4774083	4766463
7538	CP026243.1	27114834	27119314	chr21	4778979	4774499
7539	CP026243.1	27099336	27114244	chr21	4794477	4779569
7540	CP026243.1	27080371	27099240	chr21	4813442	4794573
7541	CP026243.1	27076742	27079863	chr21	4817071	4813950
7542	CP026243.1	27057170	27073917	chr21	4836643	4819896
7543	CP026243.1	27002835	27056001	chr21	4890978	4837812
7544	CP026243.1	26993776	27002734	chr21	4900037	4891079
7545	CP026243.1	26992272	26993382	chr21	4901541	4900431
7546	CP026243.1	26987240	26991389	chr21	4906573	4902424
7547	CP026243.1	26922618	26987073	chr21	4971195	4906740
7548	CP026243.1	26919391	26921720	chr21	4974422	4972093
7549	CP026243.1	26903524	26919163	chr21	4990289	4974650
7550	CP026243.1	26899382	26903278	chr21	4994431	4990535
7551	CP026243.1	26898948	26899267	chr21	4994865	4994546
7552	CP026243.1	26895705	26898818	chr21	4998108	4994995
7553	CP026243.1	26869816	26895475	chr21	5023997	4998338
7554	CP026243.1	26828100	26869554	chr21	5065713	5024259
7555	CP026243.1	26801181	26827910	chr21	5092632	5065903
7556	CP026243.1	26791897	26800954	chr21	5101916	5092859
7557	CP026243.1	26740268	26791799	chr21	5153545	5102014
7558	CP026243.1	26736513	26737920	chr21	5157300	5155893
7559	CP026243.1	26733921	26735602	chr21	5159892	5158211
7560	CP026243.1	26729334	26730614	chr21	5164479	5163199
7561	CP026243.1	26728251	26729223	chr21	5165562	5164590
7562	CP026243.1	26722326	26728070	chr21	5171487	5165743
7563	CP026243.1	26713437	26720257	chr21	5180376	5173556
7564	CP026243.1	26707155	26713256	chr21	5186658	5180557
7565	CP026243.1	23546859	23547592	chr21	8345520	8344787

7566	CP026243.1	23541333	23546770	chr21	8351046	8345609
7567	CP026243.1	23518935	23540668	chr21	8373444	8351711
7568	CP026243.1	23511952	23518782	chr21	8380427	8373597
7569	CP026243.1	23507937	23511822	chr21	8384442	8380557
7570	CP026243.1	23500844	23507833	chr21	8391535	8384546
7571	CP026243.1	23495117	23500728	chr21	8397262	8391651
7572	CP026243.1	23439606	23494698	chr21	8452773	8397681
7573	CP026243.1	23422222	23439413	chr21	8470157	8452966
7574	CP026243.1	23413976	23422075	chr21	8478403	8470304
7575	CP026243.1	23325747	23413157	chr21	8566632	8479222
7576	CP026243.1	23289397	23325614	chr21	8602982	8566765
7577	CP026243.1	23286183	23289245	chr21	8606196	8603134
7578	CP026243.1	23119027	23286032	chr21	8773352	8606347
7579	CP026243.1	22831614	23118687	chr21	9060765	8773692
7580	CP026243.1	22822508	22829688	chr21	9069871	9062691
7581	CP026243.1	22818814	22822386	chr21	9073565	9069993
7582	CP026243.1	22654814	22818693	chr21	9237565	9073686
7583	CP026243.1	22646693	22654656	chr21	9245686	9237723
7584	CP026243.1	22631630	22646589	chr21	9260749	9245790
7585	CP026243.1	22570432	22630170	chr21	9321947	9262209
7586	CP026243.1	22536662	22570329	chr21	9355717	9322050
7587	CP026243.1	22176415	22535250	chr21	9715964	9357129
7588	CP026243.1	22094221	22173862	chr21	9798158	9718517
7589	CP026243.1	21945895	22094120	chr21	9946484	9798259
7590	CP026243.1	21902482	21945729	chr21	9989897	9946650
7591	CP026243.1	21899652	21902389	chr21	9992727	9989990
7592	CP026243.1	21865290	21899390	chr21	10027089	9992989
7593	CP026243.1	21747424	21864937	chr21	10144955	10027442
7594	CP026243.1	21665961	21747253	chr21	10226418	10145126

7595	CP026243.1	21372549	21665327	chr21	10519830	10227052
7596	CP026243.1	21360031	21372455	chr21	10532348	10519924
7597	CP026243.1	21092179	21359847	chr21	10800200	10532532
7598	CP026243.1	20969464	21092051	chr21	10922915	10800328
7599	CP026243.1	20894323	20969341	chr21	10998056	10923038
7600	CP026243.1	20671148	20891068	chr21	11221231	11001311
7601	CP026243.1	20654150	20671021	chr21	11238229	11221358
7602	CP026243.1	20601782	20653846	chr21	11290597	11238533
7603	CP026243.1	20594080	20601558	chr21	11298299	11290821
7604	CP026243.1	20573465	20593964	chr21	11318914	11298415
7605	CP026243.1	20560254	20573353	chr21	11332125	11319026
7606	CP026243.1	20518314	20560032	chr21	11374065	11332347
7607	CP026243.1	20515742	20517603	chr21	11376637	11374776
7608	CP026243.1	20398745	20515531	chr21	11493634	11376848
7609	CP026243.1	20355749	20398578	chr21	11536630	11493801
7610	CP026243.1	20350430	20355547	chr21	11541949	11536832
7611	CP026243.1	20297590	20350297	chr21	11594789	11542082
7612	CP026243.1	20261612	20297469	chr21	11630767	11594910
7613	CP026243.1	20208543	20260358	chr21	11683836	11632021
7614	CP026243.1	20176699	20208431	chr21	11715680	11683948
7615	CP026243.1	20144870	20176442	chr21	11747509	11715937
7616	CP026243.1	20120507	20144492	chr21	11771872	11747887
7617	CP026243.1	20119064	20120241	chr21	11773315	11772138
7618	CP026243.1	19948970	20118863	chr21	11943409	11773516
7619	CP026243.1	19856689	19948679	chr21	12035690	11943700
7620	CP026243.1	19705855	19856588	chr21	12186524	12035791
7621	CP026243.1	19599392	19705589	chr21	12292987	12186790
7622	CP026243.1	19576996	19599281	chr21	12315383	12293098
7623	CP026243.1	19464716	19576904	chr21	12427663	12315475

7624	CP026243.1	19380500	19463730	chr21	12511879	12428649
7625	CP026243.1	19379216	19380405	chr21	12513163	12511974
7626	CP026243.1	19368799	19379078	chr21	12523580	12513301
7627	CP026243.1	19329049	19368695	chr21	12563330	12523684
7628	CP026243.1	19319747	19328789	chr21	12572632	12563590
7629	CP026243.1	19204424	19319589	chr21	12687955	12572790
7630	CP026243.1	19195554	19201168	chr21	12696825	12691211
7631	CP026243.1	18961818	19195327	chr21	12930561	12697052
7632	CP026243.1	18952846	18961468	chr21	12939533	12930911
7633	CP026243.1	18803481	18952606	chr21	13088898	12939773
7634	CP026243.1	18705503	18803350	chr21	13186876	13089029
7635	CP026243.1	18564732	18705116	chr21	13327647	13187263
7636	CP026243.1	18518754	18564363	chr21	13373625	13328016
7637	CP026243.1	18513540	18518199	chr21	13378839	13374180
7638	CP026243.1	18450404	18513248	chr21	13441975	13379131
7639	CP026243.1	18231205	18450218	chr21	13661174	13442161
7640	CP026243.1	18220278	18231065	chr21	13672101	13661314
7641	CP026243.1	18054846	18219647	chr21	13837533	13672732
7642	CP026243.1	18012491	18054640	chr21	13879888	13837739
7643	CP026243.1	17888890	18012355	chr21	14003489	13880024
7644	CP026243.1	17662569	17888562	chr21	14229810	14003817
7645	CP026243.1	17479600	17661784	chr21	14412779	14230595
7646	CP026243.1	17461361	17479425	chr21	14431018	14412954
7647	CP026243.1	17438932	17461252	chr21	14453447	14431127
7648	CP026243.1	17424770	17438755	chr21	14467609	14453624
7649	CP026243.1	17394378	17424637	chr21	14498001	14467742
7650	CP026243.1	17258458	17394151	chr21	14633921	14498228
7651	CP026243.1	17221244	17258325	chr21	14671135	14634054
7652	CP026243.1	17157777	17220970	chr21	14734602	14671409

7653	CP026243.1	17149737	17157122	chr21	14742642	14735257
7654	CP026243.1	17147094	17149347	chr21	14745285	14743032
7655	CP026243.1	17139085	17146390	chr21	14753294	14745989
7656	CP026243.1	17128541	17138184	chr21	14763838	14754195
7657	CP026243.1	17068074	17128436	chr21	14824305	14763943
7658	CP026243.1	16737779	17067969	chr21	15154600	14824410
7659	CP026243.1	16735715	16737626	chr21	15156664	15154753
7660	CP026243.1	16728548	16735497	chr21	15163831	15156882
7661	CP026243.1	16715092	16728361	chr21	15177287	15164018
7662	CP026243.1	16694918	16714391	chr21	15197461	15177988
7663	CP026243.1	16680940	16693974	chr21	15211439	15198405
7664	CP026243.1	16675459	16680613	chr21	15216920	15211766
7665	CP026243.1	16661067	16675036	chr21	15231312	15217343
7666	CP026243.1	16647493	16659931	chr21	15244886	15232448
7667	CP026243.1	16574851	16647294	chr21	15317528	15245085
7668	CP026243.1	16548450	16574662	chr21	15343929	15317717
7669	CP026243.1	16536237	16545161	chr21	15356142	15347218
7670	CP026243.1	16369321	16534516	chr21	15523058	15357863
7671	CP026243.1	16355115	16369069	chr21	15537264	15523310
7672	CP026243.1	16211248	16354972	chr21	15681131	15537407
7673	CP026243.1	16091433	16210703	chr21	15800946	15681676
7674	CP026243.1	16011713	16091214	chr21	15880666	15801165
7675	CP026243.1	15936795	16011623	chr21	15955584	15880756
7676	CP026243.1	15751616	15936635	chr21	16140763	15955744
7677	CP026243.1	15738433	15751507	chr21	16153946	16140872
7678	CP026243.1	15537642	15738168	chr21	16354737	16154211
7679	CP026243.1	13894430	13895008	chr21	18001386	18000796
7680	CP026243.1	13894154	13894429	chr21	18001969	18001699
7681	CP026243.1	13675873	13675938	chr21	18265003	18264938

7682	CP026249.1	17085987	17112132	chr22	31403059	31402604
7683	CP026249.1	17065483	17085700	chr22	29149656	29146047
7684	CP026249.1	17063817	17065391	chr22	29145647	29143648
7685	CP026249.1	16875805	17063692	chr22	29143350	29139677
7686	CP026249.1	16873477	16875608	chr22	29136960	29128501
7687	CP026249.1	16779084	16869871	chr22	29127784	29125084
7688	CP026249.1	16748080	16778892	chr22	29124835	29120851
7689	CP026249.1	16715032	16747846	chr22	29120470	29102352
7690	CP026249.1	16712060	16714713	chr22	29099038	29090641
7691	CP026249.1	16682597	16711886	chr22	29090521	29066023
7692	CP026249.1	16670035	16682454	chr22	29061629	29024488
7693	CP026249.1	16664026	16669264	chr22	29023036	28976543
7694	CP026249.1	16479347	16663920	chr22	28975599	28832156
7695	CP026249.1	16474161	16479237	chr22	28831308	28811232
7696	CP026249.1	16134050	16473734	chr22	28810533	28787447
7697	CP026249.1	15906702	16132834	chr22	28787137	28647254
7698	CP026249.1	15888968	15905596	chr22	28646264	28636877
7699	CP026249.1	15767568	15888767	chr22	28636548	28633828
7700	CP026249.1	15700408	15766484	chr22	28633148	28592855
7701	CP026249.1	15654975	15699811	chr22	28590543	28572115
7702	CP026249.1	15616472	15654885	chr22	28571732	28546567
7703	CP026249.1	15585506	15616344	chr22	28546320	28538509
7704	CP026249.1	15515578	15585142	chr22	28538345	28511701
7705	CP026249.1	15340399	15515314	chr22	28511284	28477747
7706	CP026249.1	15284498	15339957	chr22	28477607	28450766
7707	CP026249.1	15220069	15284393	chr22	28450635	28417763
7708	CP026249.1	15212369	15214908	chr22	28417551	28383797
7709	CP026249.1	15210845	15212270	chr22	28381847	28378992
7710	CP026249.1	15140493	15210673	chr22	28378782	28284466

7711	CP026249.1	14933459	15140363	chr22	28284339	28270474
7712	CP026249.1	14876964	14933322	chr22	28270307	28260486
7713	CP026249.1	14732374	14876800	chr22	28260339	28200429
7714	CP026249.1	14679598	14730961	chr22	28197611	28186910
7715	CP026249.1	14676392	14679491	chr22	28185800	28170637
7716	CP026249.1	14628739	14676271	chr22	28169441	28163479
7717	CP026249.1	14506619	14628627	chr22	28163379	28140090
7718	CP026249.1	14500200	14506324	chr22	28139822	28123159
7719	CP026249.1	14410912	14499645	chr22	28122812	28081456
7720	CP026249.1	14401790	14410636	chr22	28080904	28066787
7721	CP026249.1	14209738	14401220	chr22	28066677	28066046
7722	CP026249.1	14196870	14209476	chr22	28065862	28050579
7723	CP026249.1	14180458	14196663	chr22	28050452	28012931
7724	CP026249.1	14099241	14180105	chr22	28012769	28009533
7725	CP026249.1	13720883	14098998	chr22	28008477	27978955
7726	CP026249.1	13704011	13719653	chr22	27978438	27914289
7727	CP026249.1	13652011	13703777	chr22	27914053	27910538
7728	CP026249.1	13649416	13651819	chr22	27910353	27907042
7729	CP026249.1	13450991	13649200	chr22	27906549	27853572
7730	CP026249.1	13380848	13450783	chr22	27853311	27807221
7731	CP026249.1	13373381	13380233	chr22	27807110	27793903
7732	CP026249.1	13229428	13372568	chr22	27793678	27745198
7733	CP026249.1	13124650	13227930	chr22	27744990	27719595
7734	CP026249.1	13093248	13123835	chr22	27719319	27717235
7735	CP026249.1	13048028	13093003	chr22	27716379	27702337
7736	CP026249.1	12967032	13047643	chr22	27698356	27687103
7737	CP026249.1	12940826	12966818	chr22	27686826	27644140
7738	CP026249.1	12863515	12940708	chr22	27644018	27638604
7739	CP026249.1	12845563	12863401	chr22	27638474	27630982

7740	CP026249.1	12674482	12845145	chr22	27629801	27623508
7741	CP026249.1	12670023	12674326	chr22	27623365	27612340
7742	CP026249.1	12480911	12669846	chr22	27612250	27586425
7743	CP026249.1	12460608	12480129	chr22	27586139	27580936
7744	CP026249.1	12432050	12460474	chr22	27580844	27539721
7745	CP026249.1	12408560	12431946	chr22	27539255	27535113
7746	CP026249.1	12205940	12408434	chr22	27534853	27485138
7747	CP026249.1	11799922	12204137	chr22	27484921	27451052
7748	CP026249.1	11756721	11798842	chr22	27450834	27441029
7749	CP026249.1	11730225	11751028	chr22	27440937	27429296
7750	CP026249.1	11705352	11729942	chr22	27428932	27374521
7751	CP026249.1	11538140	11705192	chr22	27374312	27369634
7752	CP026249.1	11507269	11537644	chr22	27369475	27346196
7753	CP026249.1	11436560	11506320	chr22	27345680	27290563
7754	CP026249.1	11426127	11436441	chr22	27290418	27243427
7755	CP026249.1	11409264	11425879	chr22	27243299	27228899
7756	CP026249.1	11383344	11408346	chr22	27228247	27159984
7757	CP026249.1	11381023	11383061	chr22	27159754	26951793
7758	CP026249.1	11346745	11380754	chr22	26951651	26899627
7759	CP026249.1	11342949	11346582	chr22	26899258	26894172
7760	CP026249.1	11336468	11342811	chr22	26894012	26865769
7761	CP026249.1	11228387	11335451	chr22	26865414	26841480
7762	CP026249.1	11122750	11225701	chr22	26841311	26790029
7763	CP026249.1	11065222	11122432	chr22	26789937	26717524
7764	CP026249.1	10937897	11065066	chr22	26716991	26396670
7765	CP026249.1	10929245	10937752	chr22	26396037	26351507
7766	CP026249.1	10853369	10929103	chr22	26351417	26343864
7767	CP026249.1	10823467	10853254	chr22	26343371	26194939
7768	CP026249.1	10535976	10823225	chr22	26194838	26132073

7769	CP026249.1	10461965	10535672	chr22	26129476	25775303
7770	CP026249.1	10427794	10461845	chr22	25775163	25680219
7771	CP026249.1	10420711	10427674	chr22	25680103	25669584
7772	CP026249.1	10399515	10420507	chr22	25669336	25622600
7773	CP026249.1	10282363	10398625	chr22	25622357	25560026
7774	CP026249.1	9889643	10282250	chr22	25559149	25548315
7775	CP026249.1	9725820	9888918	chr22	25547903	25435537
7776	CP026249.1	9698266	9725724	chr22	25435351	25420086
7777	CP026249.1	9644631	9698174	chr22	25418902	25348262
7778	CP026249.1	9587433	9643809	chr22	25348129	25176544
7779	CP026249.1	9517609	9587246	chr22	25176390	25021430
7780	CP026249.1	9505997	9517434	chr22	25021279	24970834
7781	CP026249.1	9379140	9505897	chr22	24970282	24962956
7782	CP026249.1	9376093	9378841	chr22	24962823	24955814
7783	CP026249.1	9233417	9375966	chr22	24955656	24920746
7784	CP026249.1	9146653	9233282	chr22	24920629	24903653
7785	CP026249.1	9027281	9146567	chr22	24903512	24894224
7786	CP026249.1	9009974	9027078	chr22	24894029	24828922
7787	CP026249.1	8932866	9009830	chr22	24828780	24816872
7788	CP026249.1	8915127	8931743	chr22	24816673	24650083
7789	CP026249.1	8878846	8914794	chr22	24649481	24578505
7790	CP026249.1	8861908	8878711	chr22	24576637	24556260
7791	CP026249.1	8858155	8861290	chr22	24556044	24363604
7792	CP026249.1	8831165	8858062	chr22	24363398	24358417
7793	CP026249.1	8794041	8831079	chr22	24358321	24189033
7794	CP026249.1	8780357	8793951	chr22	24188830	24152261
7795	CP026249.1	8754661	8778776	chr22	24152096	24120538
7796	CP026249.1	8731108	8754054	chr22	24119205	24044685
7797	CP026249.1	8682754	8730997	chr22	24044448	24005952

7798	CP026249.1	8671375	8682577	chr22	24005852	23966405
7799	CP026249.1	8538732	8670671	chr22	23966312	23864211
7800	CP026249.1	8479071	8538577	chr22	23863894	23845276
7801	CP026249.1	8475231	8478862	chr22	23845141	23581499
7802	CP026249.1	8348650	8474894	chr22	23580444	23512135
7803	CP026249.1	8280232	8348541	chr22	23512026	23385782
7804	CP026249.1	8015535	8279177	chr22	23385445	23381814
7805	CP026249.1	7996782	8015400	chr22	23381605	23322099
7806	CP026249.1	7894364	7996465	chr22	23321944	23190005
7807	CP026249.1	7854824	7894271	chr22	23189301	23178099
7808	CP026249.1	7816228	7854724	chr22	23177922	23129679
7809	CP026249.1	7741471	7815991	chr22	23129568	23106622
7810	CP026249.1	7708580	7740138	chr22	23106015	23081900
7811	CP026249.1	7671846	7708415	chr22	23080319	23066725
7812	CP026249.1	7502355	7671643	chr22	23066635	23029597
7813	CP026249.1	7497278	7502259	chr22	23029511	23002614
7814	CP026249.1	7304632	7497072	chr22	23002521	22999386
7815	CP026249.1	7284039	7304416	chr22	22998768	22981965
7816	CP026249.1	7211195	7282171	chr22	22981830	22945882
7817	CP026249.1	7044003	7210593	chr22	22945549	22928933
7818	CP026249.1	7031896	7043804	chr22	22927810	22850846
7819	CP026249.1	6966647	7031754	chr22	22850702	22833598
7820	CP026249.1	6957164	6966452	chr22	22833395	22714109
7821	CP026249.1	6940047	6957023	chr22	22714023	22627394
7822	CP026249.1	6905020	6939930	chr22	22627259	22484710
7823	CP026249.1	6897853	6904862	chr22	22484583	22481835
7824	CP026249.1	6890394	6897720	chr22	22481536	22354779
7825	CP026249.1	6839397	6889842	chr22	22354679	22343242
7826	CP026249.1	6684286	6839246	chr22	22343067	22273430

7827	CP026249.1	6512547	6684132	chr22	22273243	22216867
7828	CP026249.1	6441774	6512414	chr22	22216045	22162502
7829	CP026249.1	6425325	6440590	chr22	22162410	22134952
7830	CP026249.1	6312773	6425139	chr22	22134856	21971758
7831	CP026249.1	6301527	6312361	chr22	21971033	21578426
7832	CP026249.1	6238319	6300650	chr22	21578313	21462051
7833	CP026249.1	6191340	6238076	chr22	21461161	21440169
7834	CP026249.1	6180573	6191092	chr22	21439965	21433002
7835	CP026249.1	6085513	6180457	chr22	21432882	21398831
7836	CP026249.1	5731200	6085373	chr22	21398711	21325004
7837	CP026249.1	5665838	5728603	chr22	21324700	21037451
7838	CP026249.1	5517305	5665737	chr22	21037209	21007422
7839	CP026249.1	5509259	5516812	chr22	21007307	20931573
7840	CP026249.1	5464639	5509169	chr22	20931431	20922924
7841	CP026249.1	5143685	5464006	chr22	20922779	20795610
7842	CP026249.1	5070739	5143152	chr22	20795454	20738244
7843	CP026249.1	5019365	5070647	chr22	20737926	20634975
7844	CP026249.1	4995262	5019196	chr22	20632289	20525225
7845	CP026249.1	4966664	4994907	chr22	20524208	20517865
7846	CP026249.1	4961418	4966504	chr22	20517727	20514094
7847	CP026249.1	4909025	4961049	chr22	20513931	20479922
7848	CP026249.1	4700922	4908883	chr22	20479653	20477615
7849	CP026249.1	4632429	4700692	chr22	20477332	20452330
7850	CP026249.1	4617377	4631777	chr22	20451412	20434797
7851	CP026249.1	4570258	4617249	chr22	20434549	20424235
7852	CP026249.1	4514996	4570113	chr22	20424116	20354356
7853	CP026249.1	4491201	4514480	chr22	20353407	20323032
7854	CP026249.1	4486364	4491042	chr22	20322536	20155484
7855	CP026249.1	4431744	4486155	chr22	20155324	20130734

7856	CP026249.1	4419739	4431380	chr22	20130451	20109648
7857	CP026249.1	4409842	4419647	chr22	20103955	20061834
7858	CP026249.1	4375755	4409624	chr22	20060754	19656539
7859	CP026249.1	4325823	4375538	chr22	19654736	19452242
7860	CP026249.1	4321421	4325563	chr22	19452116	19428730
7861	CP026249.1	4279832	4320955	chr22	19428626	19400202
7862	CP026249.1	4274537	4279740	chr22	19400068	19380547
7863	CP026249.1	4248426	4274251	chr22	19379765	19190830
7864	CP026249.1	4237311	4248336	chr22	19190653	19186350
7865	CP026249.1	4230875	4237168	chr22	19186194	19015531
7866	CP026249.1	4222202	4229694	chr22	19015113	18997275
7867	CP026249.1	4216658	4222072	chr22	18997161	18919968
7868	CP026249.1	4173850	4216536	chr22	18919850	18893858
7869	CP026249.1	4162320	4173573	chr22	18893644	18813033
7870	CP026249.1	4144297	4158339	chr22	18812648	18767673
7871	CP026249.1	4141357	4143441	chr22	18767428	18736841
7872	CP026249.1	4115686	4141081	chr22	18736026	18632746
7873	CP026249.1	4066998	4115478	chr22	18631248	18488108
7874	CP026249.1	4053566	4066773	chr22	18487295	18480443
7875	CP026249.1	4007365	4053455	chr22	18479828	18409893
7876	CP026249.1	3954127	4007104	chr22	18409685	18211476
7877	CP026249.1	3950323	3953634	chr22	18211260	18208857
7878	CP026249.1	3946623	3950138	chr22	18208665	18156899
7879	CP026249.1	3882238	3946387	chr22	18156665	18141023
7880	CP026249.1	3852199	3881721	chr22	18139793	17761678
7881	CP026249.1	3847907	3851143	chr22	17761435	17680571
7882	CP026249.1	3810224	3847745	chr22	17680218	17664013
7883	CP026249.1	3794814	3810097	chr22	17663806	17651200
7884	CP026249.1	3793999	3794630	chr22	17650938	17459456

7885	CP026249.1	3779772	3793889	chr22	17458886	17450040
7886	CP026249.1	3737864	3779220	chr22	17449764	17361031
7887	CP026249.1	3720854	3737517	chr22	17360476	17354352
7888	CP026249.1	3697297	3720586	chr22	17354057	17232049
7889	CP026249.1	3691235	3697197	chr22	17231937	17184405
7890	CP026249.1	3674876	3690039	chr22	17184284	17181185
7891	CP026249.1	3663065	3673766	chr22	17181078	17129715
7892	CP026249.1	3600337	3660247	chr22	17128302	16983876
7893	CP026249.1	3590369	3600190	chr22	16983712	16927354
7894	CP026249.1	3576337	3590202	chr22	16927217	16720313
7895	CP026249.1	3481894	3576210	chr22	16720183	16650003
7896	CP026249.1	3478829	3481684	chr22	16649831	16648406
7897	CP026249.1	3443125	3476879	chr22	16648307	16645768
7898	CP026249.1	3410041	3442913	chr22	16640607	16576283
7899	CP026249.1	3383069	3409910	chr22	16576178	16520719
7900	CP026249.1	3349392	3382929	chr22	16520277	16345362
7901	CP026249.1	3322331	3348975	chr22	16345098	16275534
7902	CP026249.1	3314356	3322167	chr22	16275170	16244332
7903	CP026249.1	3288944	3314109	chr22	16244204	16205791
7904	CP026249.1	3270133	3288561	chr22	16205701	16160865
7905	CP026249.1	3227528	3267821	chr22	16160268	16094192
7906	CP026249.1	3224128	3226848	chr22	16093108	15971909
7907	CP026249.1	3214412	3223799	chr22	15971708	15955080
7908	CP026249.1	3073539	3213422	chr22	15953974	15727842
7909	CP026249.1	3050143	3073229	chr22	15726626	15386942
7910	CP026249.1	3029368	3049444	chr22	15386515	15381439
7911	CP026249.1	2885077	3028520	chr22	15381329	15196756
7912	CP026249.1	2837640	2884133	chr22	15196650	15191412
7913	CP026249.1	2799047	2836188	chr22	15190641	15178222

7914	CP026249.1	2770155	2794653	chr22	15178079	15148790
7915	CP026249.1	2761638	2770035	chr22	15148616	15145963
7916	CP026249.1	2740206	2758324	chr22	15145644	15112830
7917	CP026249.1	2735841	2739825	chr22	15112596	15081784
7918	CP026249.1	2732892	2735592	chr22	15081592	14990805
7919	CP026249.1	2723716	2732175	chr22	14987199	14985068
7920	CP026249.1	2717326	2720999	chr22	14984871	14796984
7921	CP026249.1	2715029	2717028	chr22	14796859	14795285
7922	CP026249.1	2711020	2714629	chr22	14795193	14774976
7923	CP026249.1	467543	467998	chr22	14774689	14748544

Table S5 Detailed information of some SNP loci

Linkage group	Scafford	Physical position	Flanking Marker	primer（5’→3’）	Phenotypic variance explained %	SNP genotype	sig.
LG16	sm5_s00066	176583	Marker177834	F:GCAGTCTAATTCTAATGACGGAT<br>R:GCGTTATTAAAGGGCGTGT	23.8	A/G	0.056
LG16	sm5_s00015	6837146	Marker79521	F:TACAACTCCCTTGCTCGT<br>R:TGGAGTCTTTGTGAGGCT	23.8	C/T	0.42
LG16	sm5_s00015	6539397	Marker79433*	F:ATGTCACAAGAACTGCT<br>R:ACAAAGAAAACAGATGC	23.7	A/C	0.009
LG16	sm5_s00015	6304164	Marker79369	F:CAGGGAGCTGTGGCAAAG<br>R:GAAAAGGACAGTTGGGGAAAT	25.4	G/T	0.155
LG16	sm5_s00015	1010292	Marker77078*	F:CAGGCAGAGGGATGATAGTG<br>R:CTGTGGCGATGAAAAGGTC	26.1	C/T	0.031
LG16	sm5_s00015	7960262	Marker79971**	F:GGCATAATTGTACCTGATG<br>R:GTCGCTAAACTAAACAACAG	31	G/A	0.003
LG16	sm5_s00066	181612	Marker177839*	F:ATTGCTATCAGAATTATGG<br>R:TAGTTCCTCTTTCCCTCT	23.8	G/A	0.024
LG16	sm5_s00066	383251	Marker177940	F:TGAGATGGAGAGGATGCT<br>R:TCGTAGATTGAGTCTCGTT	23.8	G/C	0.143
LG16	sm5_s00015	2755849	Marker77912	F:TCCATACCAACATTTTCAT<br>R:GGAATCTTTTGTTATTTATACC	24.7	T/A	0.089
LG16	sm5_s00015	1909677	Marker77501	F:TTGAAAGCGACTAAGTGTC<br>R:TGAAATGAGCAATGGAAT	24.7	C/T	0.12
LG16	sm5_s00066	508309	Marker178020	F:CATTACTTCATTACCAACATCT<br>R:ATATTTGCAGGGTGCG	23.8	A/T	0.23
LG16	sm5_s00066	445145	Marker177982	F:TAACCGACAATGACACGAC<br>R:CAAAGACAAATAAGCCCAC	23.8	C/T	0.058
LG22	sm5_s00182	45328	Marker228232*	F:GTTGGAGGGTTGAAAG<br>R:ACTTTGACCTAAACTTCTC	26.2	A/T	0.013
LG16	sm5_s00015	5164167	Marker78928**	F:GTATTTGTTTCTGTAGATGCTC<br>R:AGCCACAACATGTAAATT	23.5	G/C	0.001
LG16	sm5_s00015	492876	Marker76817*	F:TAATCTAAGGATGATGACTGC<br>R:TGTGTCTATCGATTTGTTTGT	26.1	G/A	0.044
LG16	sm5_s00015	4861772	Marker78790**	F:CCAAACGACCTGTGGCAAG<br>R:TGTGAGCAGCAGCGATGAG	26.1	C/A	0.005

Note: * indicates significant association; ** indicates very significant association.

Table S6 Results of haplotype analysis

ID	Haplotypes	Haplotype frequency
1	AACCGAA	0.01
2	AATCGCA	0.002
3	AATCGCC	0.023
4	AACTGCC*	0.077
5	ATTCAAA	0.028
6	ATCCAAC	0.004
7	ATTCGAC	0.018
8	ATCCGCC*	0.039
9	ATCTGCC*	0.136
10	GATTACC	0.019
11	GACCGCC*	0.052
12	GTCCACC*	0.184
13	GTCTAAC	0.005
14	GTTTACC*	0.11
15	GTCCGCA*	0.035
16	GTCTGAC	0.022
17	GTCCGCC*	0.268

Note: * indicate the haplotype frequency>0.03 (LFT>0.03); A, T, G, C is adenine, thymine, guanine and cytosine respectively.

Table S7 Candidate genes of UTT-QTL markers identified by compartive genomics

Genetic_Info				Physical_Info				annotation	
Linkage group	initiate-postive	finally-postive	Distance(cM)	Scafford	initiate-postive	finally-postive	Flanking markers	KEGG_annotation	Description
13	19.426	19.426	0	sm5 s00261	13494	13494	Marker206141	--	peptidase M20 domain-containing protein 2-like
13	26.478	28.232	1.754	sm5 s00020	399562	1481717	Marker93558	--	putative ras and Rab interactor-like protein
13	0.87	0.87	0	sm5 s00177	3531	83551	Marker227711	K06083|0|mze:101469375|K06083 WAS protein family, member 3 | (RefSeq) wiskott-Aldrich syndrome protein family member 3	wiskott-Aldrich syndrome protein family member 3-like isoform X1
13	19.426	20.657	1.231	sm5 s00107	100434	758786	Marker206523	K08446|0|ola:101162663|K08446 CD97 antigen | (RefSeq) adgre5; adhesion G protein-coupled receptor E5	adhesion G protein-coupled receptor E5
							Marker206485	--	hypothetical protein F2P81_003464
16	83.846	83.846	0	sm5 s00015	8943217	8943217	Marker177795	--	cat eye syndrome critical region protein 2 homolog isoform X2 [Scophthalmus maximus]

16	103.136	107.981	4.845	sm5 s00015	172474	4495469	Marker77268	K18743|0|mze:101479582|K18743 caprin-1 | (RefSeq) caprin1; cell cycle associated protein 1	caprin-1 isoform X1 [Seriola dumerili]
							Marker76893	K08146|0|ola:101166043|K08146 MFS transporter, SP family, solute carrier family 2 (facilitated glucose transporter), member 9 | (RefSeq) slc2a9; solute carrier family 2 member 9	putative solute carrier family 2 facilitated glucose transporter member 9 [Scophthalmus maximus]
							Marker76958	K04319|0|tru:101078384|K04319 G protein-coupled receptor 22 | (RefSeq) gpr22; G protein-coupled receptor 22	G-protein coupled receptor 22-like[Scophthalmus maximus]
							Marker76883	K04437|0|mze:101468348|K04437 filamin | (RefSeq) filamin-C-like	filamin-C-like isoform X2 [Scophthalmus maximus]
							Marker77021	K17452|6.30793e-129|mze:101464281|K17452 zinc finger protein AEBP2 | (RefSeq) zinc finger protein aebp2	zinc finger protein aebp2-like isoform X1 [Scophthalmus maximus]
							Marker76902	--	5'-nucleotidase domain-containing protein 3 [Scophthalmus maximus]

							Marker76781	K05853|0|mze:101483402|K05853 Ca2+ transporting ATPase, sarcoplasmic/endoplasmic reticulum [EC:3.6.3.8] | (RefSeq) atp2a3; ATPase sarcoplasmic/endoplasmic reticulum Ca2+ transporting 3	Calcium-transporting ATPase [Scophthalmus maximus]
16	64.559	70.465	5.906	sm5 s00015	5164167	7495944	Marker79524	K01251|0|ola:101173464|K01251 adenosylhomocysteinase [EC:3.3.1.1] | (RefSeq) putative adenosylhomocysteinase 3	S-adenosylhomocysteine hydrolase-like protein 1 isoform X2 [Xiphias gladius]
							Marker79369	--	hypothetical protein SMAX5B_009794 [Scophthalmus maximus]
							Marker79103	K16882|0|mze:101476438|K16882 protein piccolo | (RefSeq) protein piccolo-like	putative protein piccolo isoform 2 [Scophthalmus maximus]
16	89.346	101.171	11.825	sm5 s00015	338267	8013154	Marker79251	K18078|0|mze:101477653|K18078 protein tyrosine phosphatase domain-containing protein 1 [EC:3.1.3.-] | (RefSeq) protein tyrosine phosphatase domain-containing protein 1-like	putative protein tyrosine phosphatase domain-containing protein 1-like [Scophthalmus maximus]

Marker79433	--	putative genetic suppressor element 1-like [Scophthalmus maximus]
Marker79453	K10873|0|ola:101162524|K10873 DNA repair and recombination protein RAD52 | (RefSeq) rad52; RAD52 homolog, DNA repair protein	A repair protein RAD52 -like [Scophthalmu
Marker77349	K16728|1.09602e-61|mze:101474819|K16728 centrosomal protein CEP152 | (RefSeq) cep152; centrosomal protein 152	centrosomal protein of 152 kDa isoform X5 [Scophthalmus maximus]
Marker77608	K16779|0|ola:101167615|K16779 Rab-3A-interacting protein | (RefSeq) rab3il1; RAB3A interacting protein like 1	guanine nucleotide exchange factor for Rab-3A isoform X5 [Scophthalmus maximus]
Marker78790	K05087|0|mze:101479543|K05087 insulin-like growth factor 1 receptor [EC:2.7.10.1] | (RefSeq) igf1r; insulin-like growth factor 1 receptor	insulin-like growth factor 1 receptor [Scophthalmus maximus]

							Marker77599	K20191|5.23854e-11|mze:101466045|K20191 Hermansky-Pudlak syndrome 5 protein | (RefSeq) hps5; HPS5, biogenesis of lysosomal organelles complex 2 subunit 2	putative Hermansky-Pudlak syndrome 5 protein [Scophthalmus maximus]
16	59.265	61.047	1.782	sm5 s00015	5134604	6417388	Marker79135	K05460|0|mze:101470516|K05460 hepatocyte growth factor | (RefSeq) hgf; hepatocyte growth factor	hepatocyte growth factor [Scophthalmus maximus]
22	168.668	168.668	0	sm5 s00118	232801	478554	Marker211914	K10396|0|ola:101155960|K10396 kinesin family member 5 | (RefSeq) kinesin-1 heavy chain	Kinesin-like protein [Scophthalmus maximus]

Table S8 TOP 30 of KEGG pathways enrichment in UTT-QTL

NO.	Pathway involved	gene Num in UTT-QTL	Adjusted *p* value
1	Nucleotide excision repair	18	0.001231
2	Drug metabolism - other enzymes	10	0.001943
3	Fatty acid metabolism	22	0.003192
4	Gap junction	22	0.003839
5	Ubiquitin mediated proteolysis	35	0.00429
6	Peroxisome	18	0.005261
7	mTOR signaling pathway	25	0.00583
8	Apoptosis	19	0.00628
9	GnRH signaling pathway	17	0.008059
10	FoxO signaling pathway	24	0.008478
11	Jak-STAT signaling pathway	21	0.01073
12	Calcium signaling pathway	28	0.018616
13	Adherens junction	27	0.01983
14	MAPK signaling pathway	33	0.01995
15	Fatty acid degradation	27	0.02312
16	Focal adhesion	20	0.0294
17	p53 signaling pathway	14	0.0311
18	Adipocytokine signaling pathway	15	0.03157
19	Adrenergic signaling in cardiomyocytes	27	0.0355
20	Vascular smooth muscle contraction	13	0.03802
21	PPAR signaling pathway	11	0.04134
22	Cytokine-cytokine receptor interaction	31	0.04417
23	Progesterone-mediated oocyte maturation	10	0.04742
24	Endocytosis	28	0.04908
25	Purine metabolism	15	0.05371
26	Insulin signaling pathway	24	0.05978
27	Cell adhesion molecules (CAMs)	21	0.0739
28	Regulation of actin cytoskeleton	21	0.08328
29	Lysosome	15	0.0977
30	Phosphatidylinositol signaling system	9	0.1232

Note: using p-adjust values cut-offs (0.05/0.01) for significances.

Table S9 Candidate genes of UTT identified by comparative genomics

QTL name	LG	Gene Symbol	Description	Pathway involved
qUTT16-1	16	MLYCD	malonyl-CoA decarboxylase, mitochondrial	ko04146//Peroxisome.
qUTT16-2	16	HRas	GTPase HRas-like	ko04010//MAPK signaling pathway; ko04530//Tight junction; ko04068//FoxO signaling pathway; ko04910//Insulin signaling pathway; ko04012//ErbB signaling pathwayko04912//GnRH
qUTT16-4	16	UBE2H	ubiquitin-conjugating enzyme E2 H	ko04120//Ubiquitin mediated proteolysis.
qUTT16-4	16	DHPR	Voltage-dependent calcium channel	ko04010//MAPK signaling pathway; ko04020//Calcium signaling pathway; ko04261//Adrenergic signaling in cardiomyocytes.
qUTT16-4	16	CDH	B-cadherin-like	ko04514//Cell adhesion molecules (CAMs); ko04520//Adherens
qUTT16-4	16	MEK1/3	Ras-like protein 1	ko04068//FoxO signaling pathway.
qUTT16-4	16	IGF1R	Insulin-like peptide receptor beta chain	ko04144//Endocytosis; ko04510//Focal adhesion; ko04068//FoxO signaling pathway; ko04520//Adherens junction; ko04114//Oocyte meiosis; ko04914//Progesterone-mediated oocyte maturation.
qUTT16-5	16	TJP	tight junction protein ZO-1	ko04530//Tight junction;ko04520//Adherens junction;ko04540//Gap junction
qUTT16-5	16	Smad3	mothers against decapentaplegic homolog 3	ko04144//Endocytosis; ko04068//FoxO signaling pathway; ko04520//Adherens junction; ko04110//Cell cycle; ko04350//TGF-beta signaling pathway.
qUTT16-5	16	JIP	JNK-interacting protein 1	ko04068//FoxO signaling pathway; ko04010//MAPK signaling
qUTT16-6	16	IDH	Isocitrate dehydrogenase [NADP], mitochondrial (Precursor)	ko01200//Carbon metabolism; ko04146//Peroxisome; ko01230//Biosynthesis of amino acids; ko00480//Glutathione metabolism; ko00020//Citrate cycle (TCA cycle); ko01210//2-Oxocarboxylic acid metabolism.
qUTT16-6	16	CD82	CD82 antigen-like	ko04115//p53 signaling pathway.
qUTT16-6	16	p48	DNA damage-binding protein 2-like	ko04120//Ubiquitin mediated proteolysis; ko04115//p53 signaling pathway; ko03420//Nucleotide excision repair.
qUTT16-6	16	PIDD	P53-induced protein with a death domain-like	ko04115//p53 signaling pathway.

qUTT16-6	16	PKA	cAMP-dependent protein kinase type II regulatory subunit	ko04010//MAPK signaling pathway; ko04020//Calcium signaling pathway; ko04261//Adrenergic signaling in cardiomyocytes; ko04310//Wnt signaling pathway;ko04910//Insulin signaling pathway; ko04270//Vascular smooth muscle contraction; ko04916//Melanogenesis; ko04114//Oocyte meiosis; ko04912//GnRH signaling pathway; ko04914//Progesterone-mediated oocyte maturation; ko04540//Gap junction;
qUTT16-6	16	VLACS	very long-chain acyl-CoA	ko04146//Peroxisome.
qUTT16-6	16	PIAS	E3 SUMO-protein ligase PIAS1	ko04120//Ubiquitin mediated proteolysis; ko04630//Jak-STAT signaling pathway.
qUTT16-6	16	FLNB	filamin-B-like	ko04010//MAPK signaling pathway; ko04510//Focal adhesion.
qUTT16-6	16	PEX11	peroxisomal membrane protein 11A	ko04146//Peroxisome.
qUTT16-6	16	UBE2Q	ubiquitin-conjugating enzyme E2 Q	ko04120//Ubiquitin mediated proteolysis.
qUTT16-6	16	DLG1	Disks large 1 tumor suppressor	ko04115//p53 signaling pathway.
qUTT16-6	16	Smad6	mothers against decapentaplegic	ko04350//TGF-beta signaling pathway.
qUTT16-6	16	TPM	tropomyosin alpha-1 chain-like	ko04261//Adrenergic signaling in cardiomyocytes; ko04260//Cardiac muscle contraction.
qUTT16-6	16	HERC2	Probable E3 ubiquitin-protein ligase	ko04120//Ubiquitin mediated proteolysis.
qUTT16-6	16	HSP90b	heat shock protein 90kDa beta	--
qUTT16-6	16	HSP40	heat shock protein family (Hsp40)	--
qUTT22-1	22	CED9	Apoptosis regulator ced-9	ko04210//Apoptosis.
qUTT22-1	22	CBL	E3 ubiquitin-protein ligase cblA	ko04144//Endocytosis; ko04910//Insulin signaling pathway; ko04120//Ubiquitin mediated proteolysis; ko04012//ErbB signaling pathway.
qUTT22-1	22	UBE2V	Probable ubiquitin-conjugating enzyme E2 variant	ko04120//Ubiquitin mediated proteolysis.
qUTT22-1	22	ADCY	Ca(2+)/calmodulin-responsive adenylate cyclase	ko04010//MAPK signaling pathway; ko04020//Calcium signaling pathway; ko04261//Adrenergic signaling in cardiomyocytes.
qUTT22-1	22	--	Voltage-dependent calcium channel type A subunit alpha-1	ko04020//Calcium signaling pathway; ko04261//Adrenergic signaling in cardiomyocytes.
qUTT22-1	22	stk11	Serine/threonine-protein kinase stk11 homolog	ko04068//FoxO signaling pathway; ko04920//Adipocytokine signaling pathway; ko04150//mTOR signaling pathway.
qUTT22-1	22	plk2	Serine/threonine-protein kinase	ko04068//FoxO signaling pathway.
qUTT22-1	22	FACS	Fatty acyl-CoA synthetase B	ko00071//fatty acid metabolism.

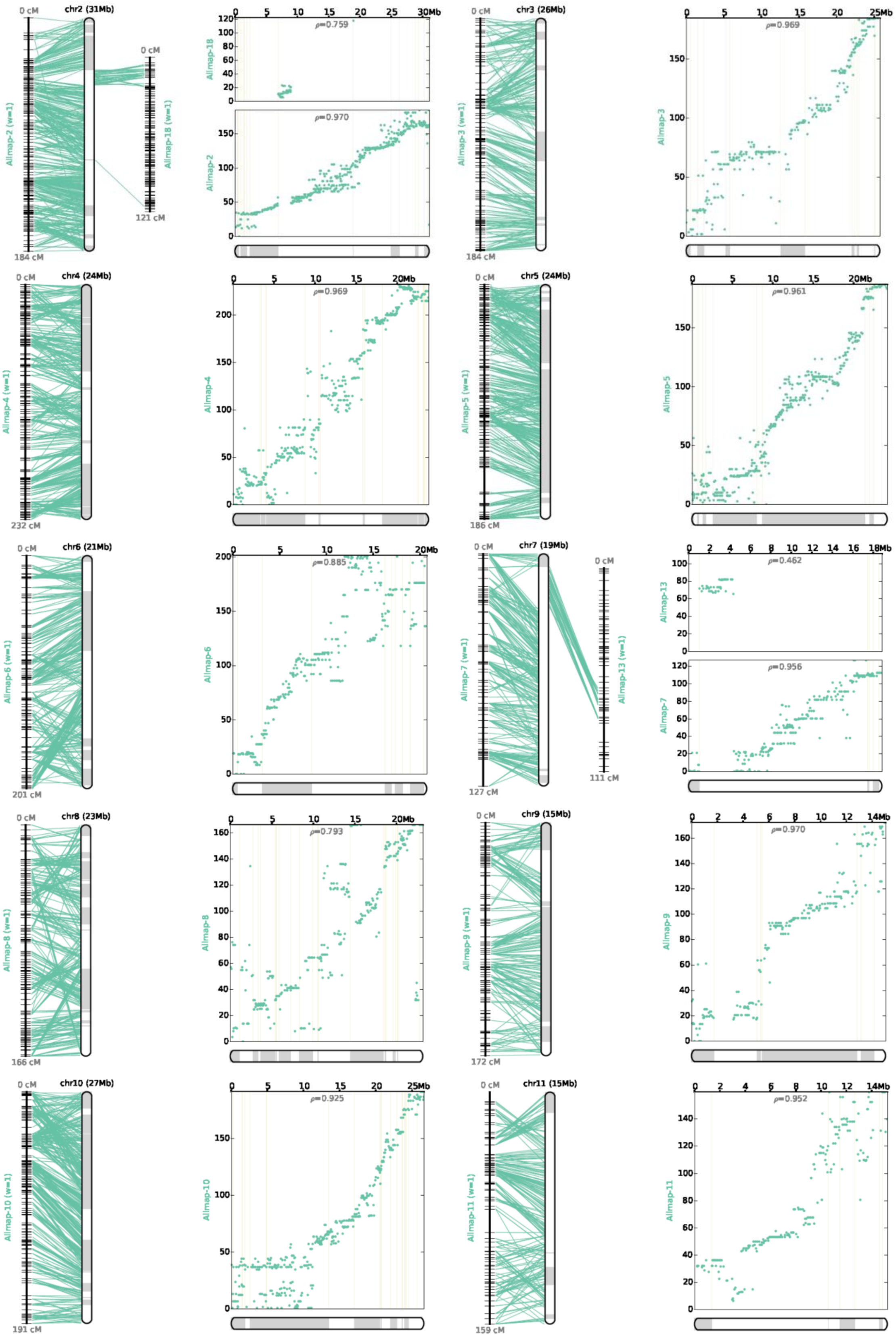

chr2 (31Mb)
Allmap-2 (w=1)
Allmap-18 (w=1)
ρ=0.759
ρ=0.970
chr3 (26Mb)
Allmap-3 (w=1)
ρ=0.969
chr4 (24Mb)
Allmap-4 (w=1)
ρ=0.969
chr5 (24Mb)
Allmap-5 (w=1)
ρ=0.961
chr6 (21Mb)
Allmap-6 (w=1)
ρ=0.885
chr7 (19Mb)
Allmap-7 (w=1)
Allmap-13 (w=1)
ρ=0.462
ρ=0.956
chr8 (23Mb)
Allmap-8 (w=1)
ρ=0.793
chr9 (15Mb)
Allmap-9 (w=1)
ρ=0.970
chr10 (27Mb)
Allmap-10 (w=1)
ρ=0.925
chr11 (15Mb)
Allmap-11 (w=1)
ρ=0.952

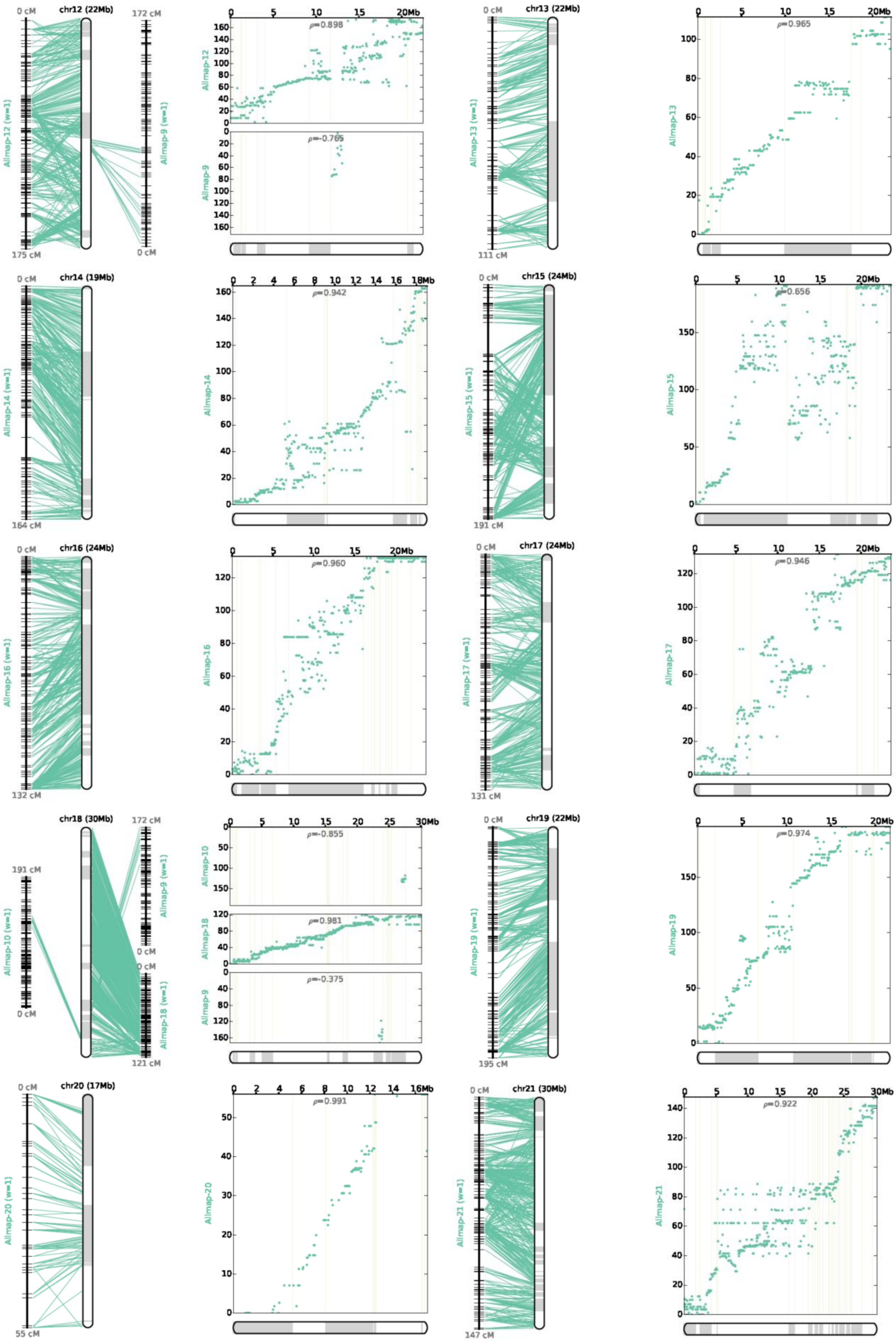

chr12 (22Mb)
Allmap-12 (w=1)
Allmap-9 (w=1)
ρ=0.898
ρ=-0.765
chr13 (22Mb)
Allmap-13 (w=1)
ρ=0.965
chr14 (19Mb)
Allmap-14 (w=1)
ρ=0.942
chr15 (24Mb)
Allmap-15 (w=1)
ρ=0.656
chr16 (24Mb)
Allmap-16 (w=1)
ρ=0.960
chr17 (24Mb)
Allmap-17 (w=1)
ρ=0.946
chr18 (30Mb)
Allmap-10 (w=1)
Allmap-18 (w=1)
ρ=-0.855
ρ=0.981
ρ=-0.375
chr19 (22Mb)
Allmap-19 (w=1)
ρ=0.974
chr20 (17Mb)
Allmap-20 (w=1)
ρ=0.991
chr21 (30Mb)
Allmap-21 (w=1)
ρ=0.922

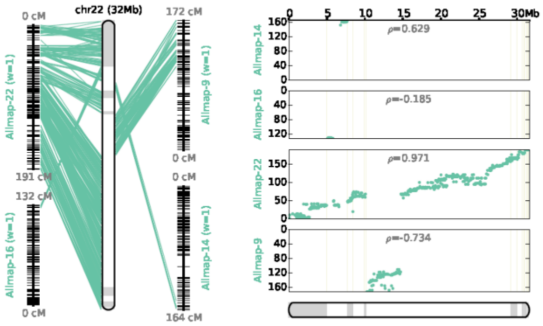

Fig S1. The co-linearity of chromosome 2-22 between the SLAF-based high-density genetic map and the corresponding chromosome assembly by ALLMAP.

LG8

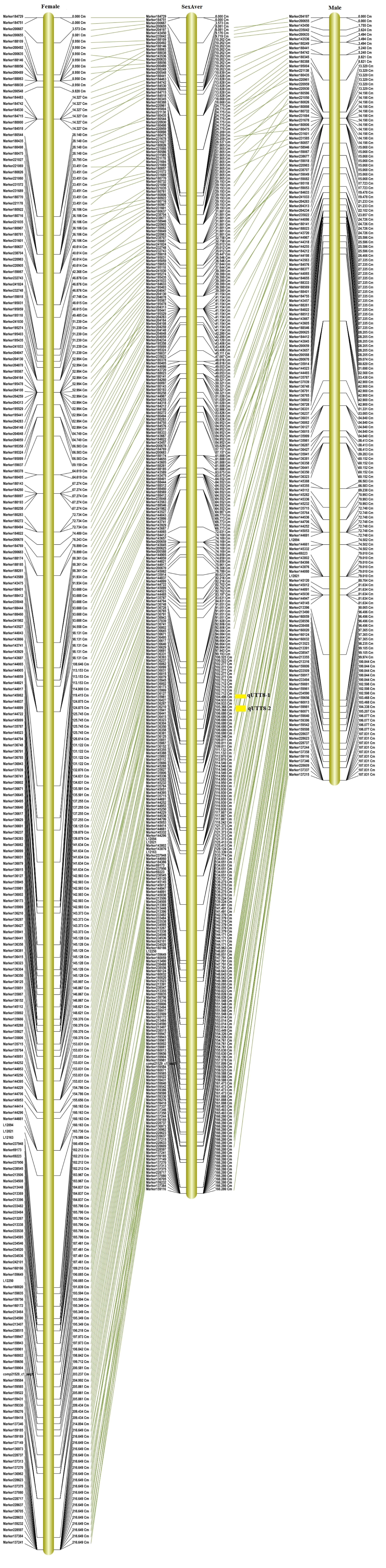

LG10

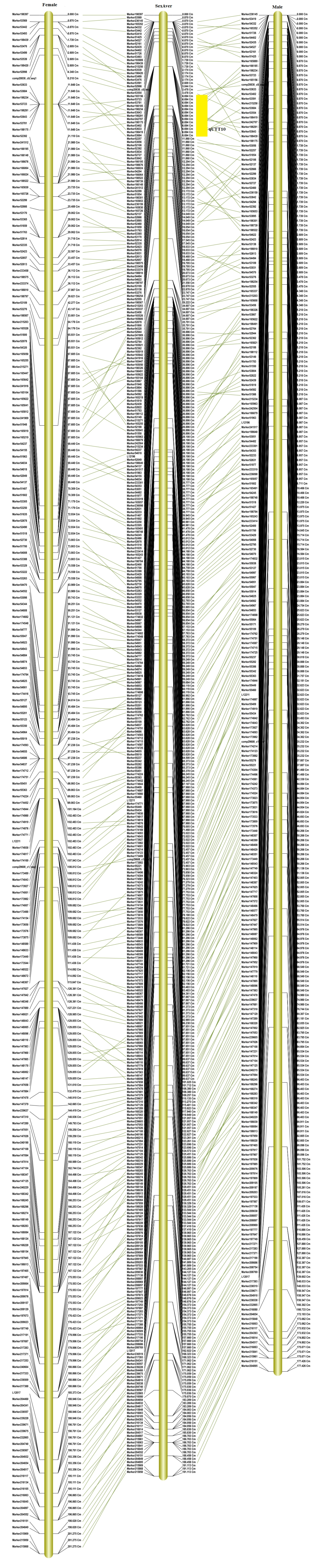

LG13

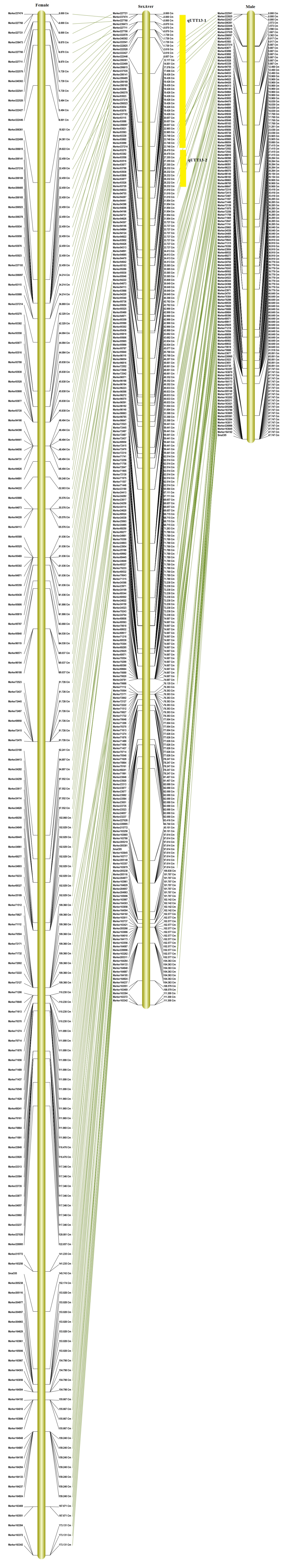

LG16

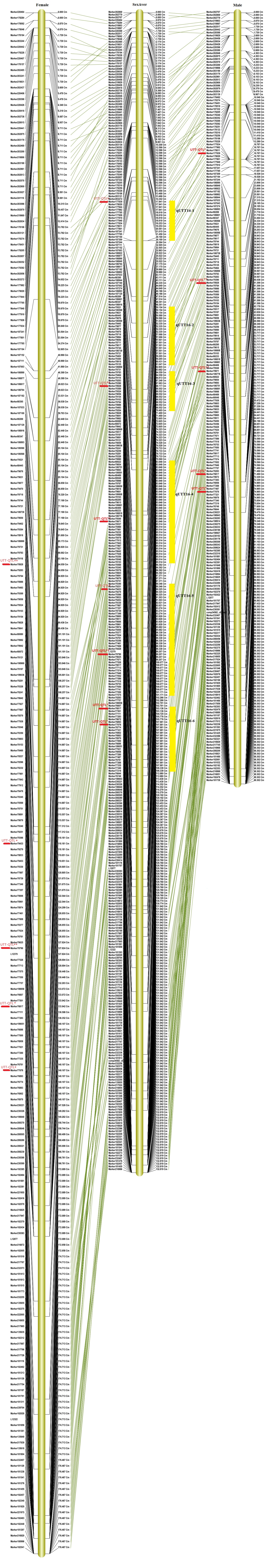
Female
SexAver
Male
qUTT16-1
qUTT16-2
qUTT16-3
qUTT16-4
qUTT16-5
qUTT16-6

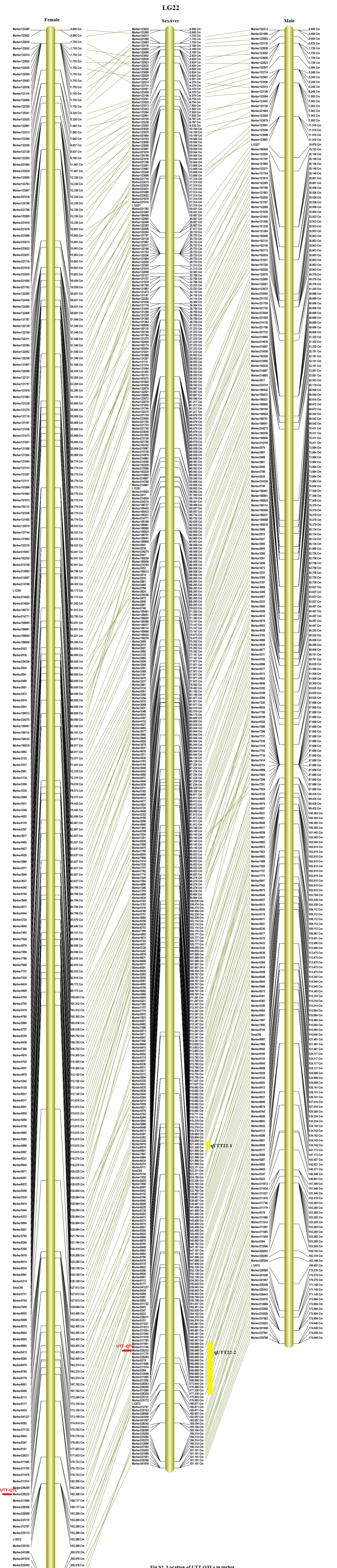

Fig S2 Location of UTT QTLs in turbot
Note: UTT-QTL** showed that SNP loci were highly significantly correlated with UTT ($p < 0.01$); UTT-QTL** showed that SNP loci were significantly correlated with UTT ($p < 0.05$).

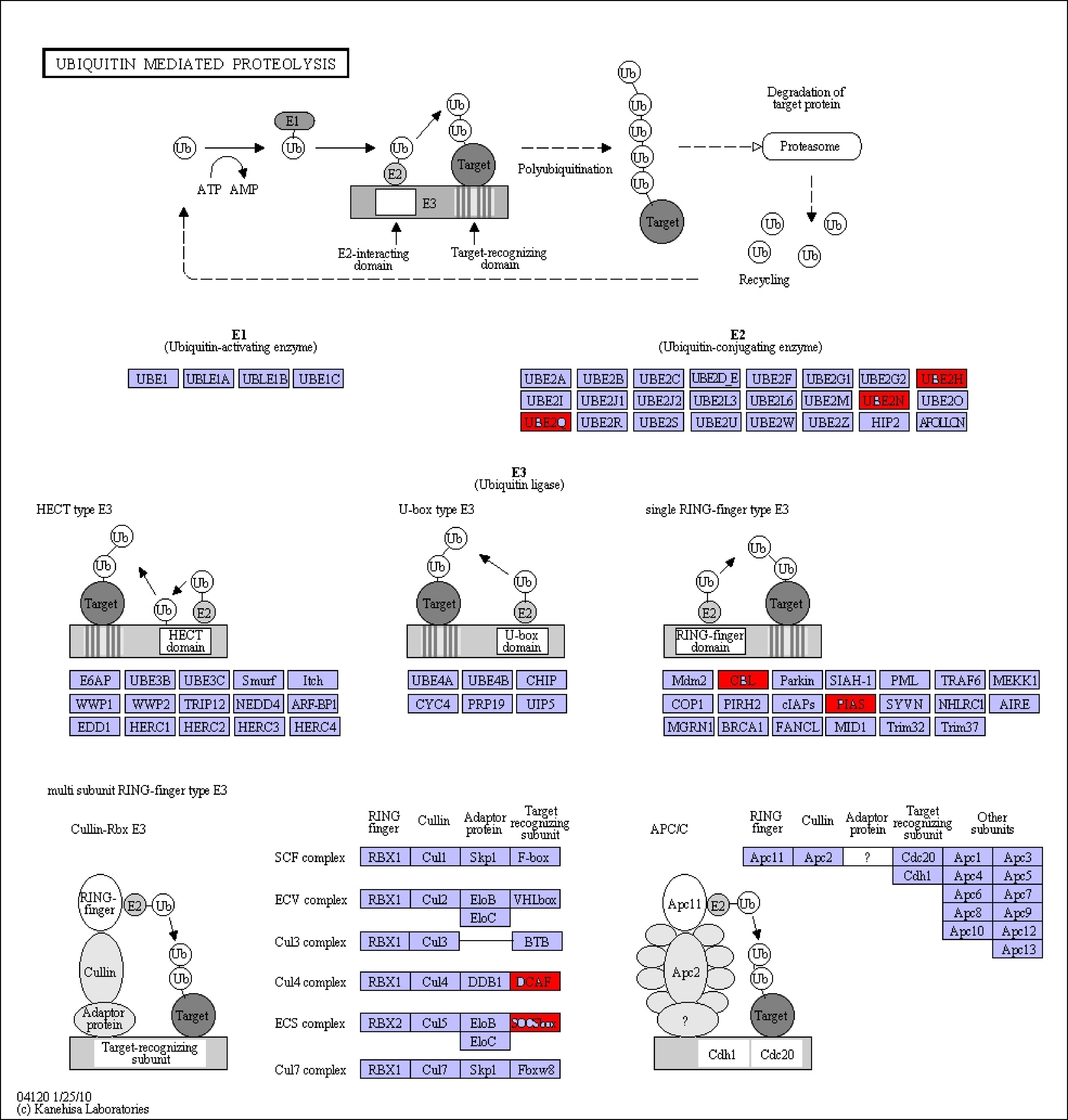
UBIQUITIN MEDIATED PROTEOLYSIS
Ub
ATP AMP
E1
E2
E3
Target
Polyubiquitination
Degradation of target protein
Proteasome
Recycling
E2-interacting domain
Target-recognizing domain
E1 (Ubiquitin-activating enzyme)
UBE1 UBLE1A UBLE1B UBE1C
E2 (Ubiquitin-conjugating enzyme)
UBE2A UBE2B UBE2C UBE2D_E UBE2F UBE2G1 UBE2G2 UBE2H
UBE2I UBE2J1 UBE2J2 UBE2L3 UBE2L6 UBE2M UBE2N UBE2O
UBE2Q UBE2R UBE2S UBE2U UBE2W UBE2Z HIP2 APOLLON
E3 (Ubiquitin ligase)
HECT type E3
HECT domain
E6AP UBE3B UBE3C Smurf Itch
WWP1 WWP2 TRIP12 NEDD4 ARF-BP1
EDD1 HERC1 HERC2 HERC3 HERC4
U-box type E3
U-box domain
UBE4A UBE4B CHIP
CYC4 PRP19 UIP5
single RING-finger type E3
RING-finger domain
Mdm2 CBL Parkin SIAH-1 PML TRAF6 MEKK1
COP1 PIRH2 cIAPs PIAS SYVN NHLRC1 AIRE
MGRN1 BRCA1 FANCL MID1 Trim32 Trim37
multi subunit RING-finger type E3
Cullin-Rbx E3
RING-finger
Cullin
Adaptor protein
Target-recognizing subunit
RING finger
Cullin
Adaptor protein
Target recognizing subunit
SCF complex RBX1 Cul1 Skp1 F-box
ECV complex RBX1 Cul2 EloB VHLbox EloC
Cul3 complex RBX1 Cul3 BTB
Cul4 complex RBX1 Cul4 DDB1 DCAF
ECS complex RBX2 Cul5 EloB SOCSbox EloC
Cul7 complex RBX1 Cul7 Skp1 Fbxw8
APC/C
RING finger
Cullin
Adaptor protein
Target recognizing subunit
Other subunits
Apc11 Apc2 ? Cdc20 Cdh1
Apc1 Apc3 Apc4 Apc5 Apc6 Apc7 Apc8 Apc9 Apc10 Apc12 Apc13
Apc11
Apc2
?
Cdh1 Cdc20
04120 1/25/10
(c) Kanehisa Laboratories

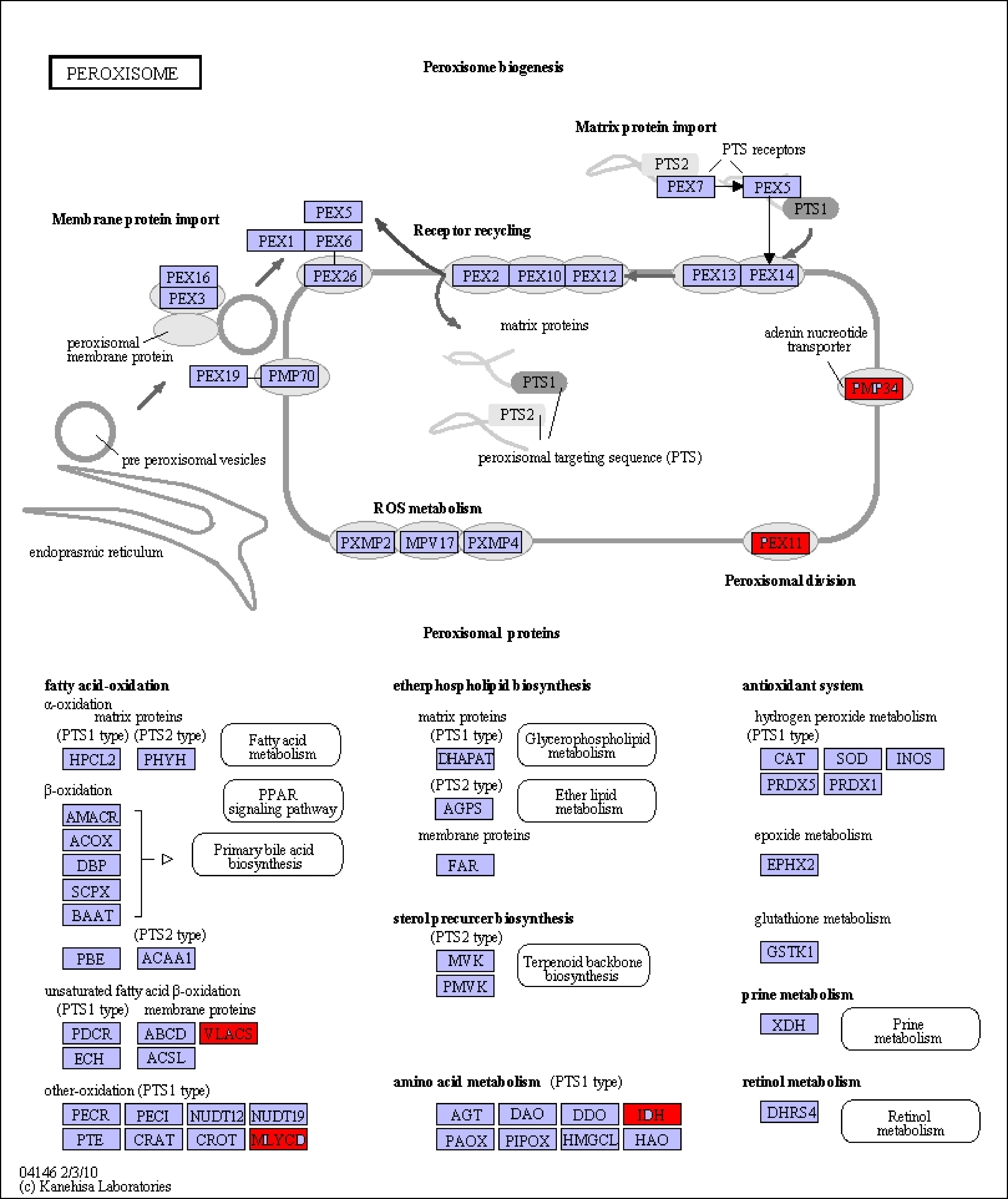
PEROXISOME
Peroxisome biogenesis
Matrix protein import
PTS2
PTS receptors
PEX7
PEX5
PTS1
Membrane protein import
PEX5
PEX1
PEX6
Receptor recycling
PEX16
PEX3
PEX26
PEX2
PEX10
PEX12
PEX13
PEX14
matrix proteins
peroxisomal membrane protein
adenin nucreotide transporter
PEX19
PMP70
PMP34
PTS1
PTS2
pre peroxisomal vesicles
peroxisomal targeting sequence (PTS)
ROS metabolism
PXMP2
MPV17
PXMP4
PEX11
endoprasmic reticulum
Peroxisomal division
Peroxisomal proteins
fatty acid-oxidation
α-oxidation
matrix proteins
(PTS1 type) (PTS2 type)
HPCL2
PHYH
Fatty acid metabolism
β-oxidation
PPAR signaling pathway
AMACR
ACOX
DBP
SCPX
BAAT
Primary bile acid biosynthesis
(PTS2 type)
PBE
ACAA1
unsaturated fatty acid β-oxidation
(PTS1 type) membrane proteins
PDCR
ABCD
VLACS
ECH
ACSL
other-oxidation (PTS1 type)
PECR
PECI
NUDT12
NUDT19
PTE
CRAT
CROT
MLYCD
etherphospholipid biosynthesis
matrix proteins
(PTS1 type)
DHAPAT
Glycerophospholipid metabolism
(PTS2 type)
AGPS
Ether lipid metabolism
membrane proteins
FAR
sterol precurcer biosynthesis
(PTS2 type)
MVK
PMVK
Terpenoid backbone biosynthesis
amino acid metabolism (PTS1 type)
AGT
DAO
DDO
IDH
PAOX
PIPOX
HMGCL
HAO
antioxidant system
hydrogen peroxide metabolism
(PTS1 type)
CAT
SOD
INOS
PRDX5
PRDX1
epoxide metabolism
EPHX2
glutathione metabolism
GSTK1
prine metabolism
XDH
Prine metabolism
retinol metabolism
DHRS4
Retinol metabolism
04146 2/3/10
(c) Kanehisa Laboratories

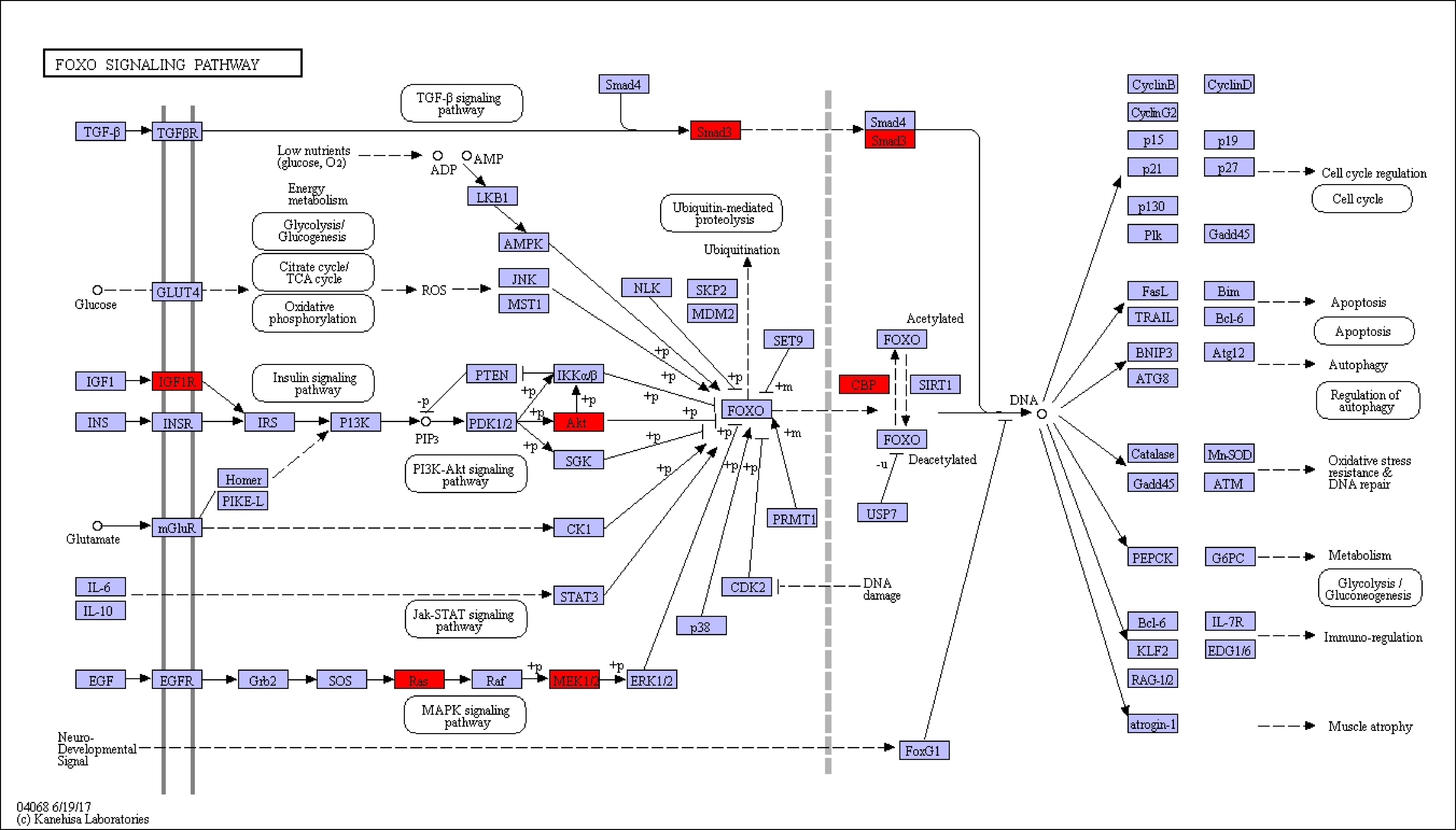
FOXO SIGNALING PATHWAY
TGF-β
TGFβR
TGF-β signaling pathway
Smad4
Smad3
Smad4
Smad3
Low nutrients (glucose, O2)
ADP
AMP
LKB1
AMPK
Energy metabolism
Glycolysis/ Glucogenesis
Citrate cycle/ TCA cycle
Oxidative phosphorylation
Glucose
GLUT4
ROS
JNK
MST1
Ubiquitin-mediated proteolysis
Ubiquitination
NLK
SKP2
MDM2
SET9
IGF1
IGF1R
Insulin signaling pathway
PTEN
IKKα/β
INS
INSR
IRS
PI3K
PIP3
PDK1/2
Akt
SGK
FOXO
PI3K-Akt signaling pathway
Homer
PIKE-L
Glutamate
mGluR
CK1
PRMT1
IL-6
IL-10
STAT3
Jak-STAT signaling pathway
CDK2
DNA damage
p38
EGF
EGFR
Grb2
SOS
Ras
Raf
MEK1/2
ERK1/2
MAPK signaling pathway
Neuro-Developmental Signal
FoxG1
Acetylated
FOXO
CBP
SIRT1
FOXO
Deacetylated
USP7
DNA
CyclinB
CyclinD
CyclinG2
p15
p19
p21
p27
Cell cycle regulation
Cell cycle
p130
Plk
Gadd45
FasL
Bim
TRAIL
Bcl-6
Apoptosis
Apoptosis
BNIP3
Atg12
ATG8
Autophagy
Regulation of autophagy
Catalase
MnSOD
Gadd45
ATM
Oxidative stress resistance & DNA repair
PEPCK
G6PC
Metabolism
Glycolysis / Gluconeogenesis
Bcl-6
IL-7R
KLF2
EDG1/6
Immuno-regulation
RAG-1/2
atrogin-1
Muscle atrophy
+p
+m
-p
-u
04068 6/19/17
(c) Kanehisa Laboratories

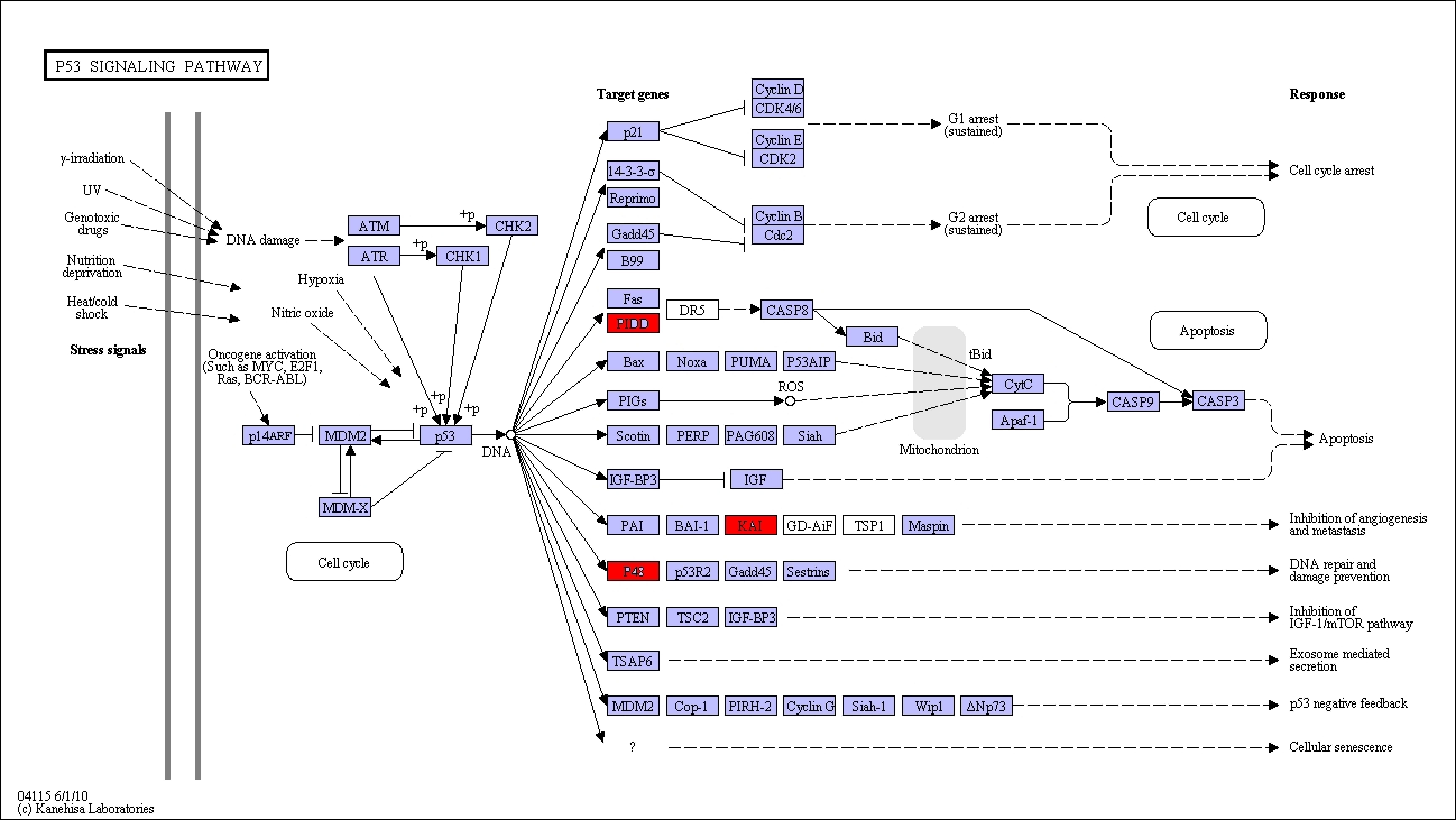
P53 SIGNALING PATHWAY
Stress signals
γ-irradiation
UV
Genotoxic drugs
Nutrition deprivation
Heat/cold shock
DNA damage
Hypoxia
Nitric oxide
Oncogene activation (Such as MYC, E2F1, Ras, BCR-ABL)
ATM
ATR
CHK2
CHK1
+p
p14ARF
MDM2
p53
MDM-X
DNA
Cell cycle
Target genes
p21
14-3-3-σ
Reprimo
Gadd45
B99
Fas
PIDD
DR5
CASP8
Bid
tBid
Bax
Noxa
PUMA
P53AIP
PIGs
ROS
Scotin
PERP
PAG608
Siah
Mitochondrion
CytC
Apaf-1
CASP9
CASP3
IGF-BP3
IGF
PAI
BAI-1
KAI
GD-AiF
TSP1
Maspin
P48
p53R2
Gadd45
Sestrins
PTEN
TSC2
IGF-BP3
TSAP6
MDM2
Cop-1
PIRH-2
Cyclin G
Siah-1
Wip1
ΔNp73
?
Cyclin D
CDK4/6
Cyclin E
CDK2
Cyclin B
Cdc2
G1 arrest (sustained)
G2 arrest (sustained)
Response
Cell cycle arrest
Cell cycle
Apoptosis
Apoptosis
Inhibition of angiogenesis and metastasis
DNA repair and damage prevention
Inhibition of IGF-1/mTOR pathway
Exosome mediated secretion
p53 negative feedback
Cellular senescence
04115 6/1/10
(c) Kanehisa Laboratories

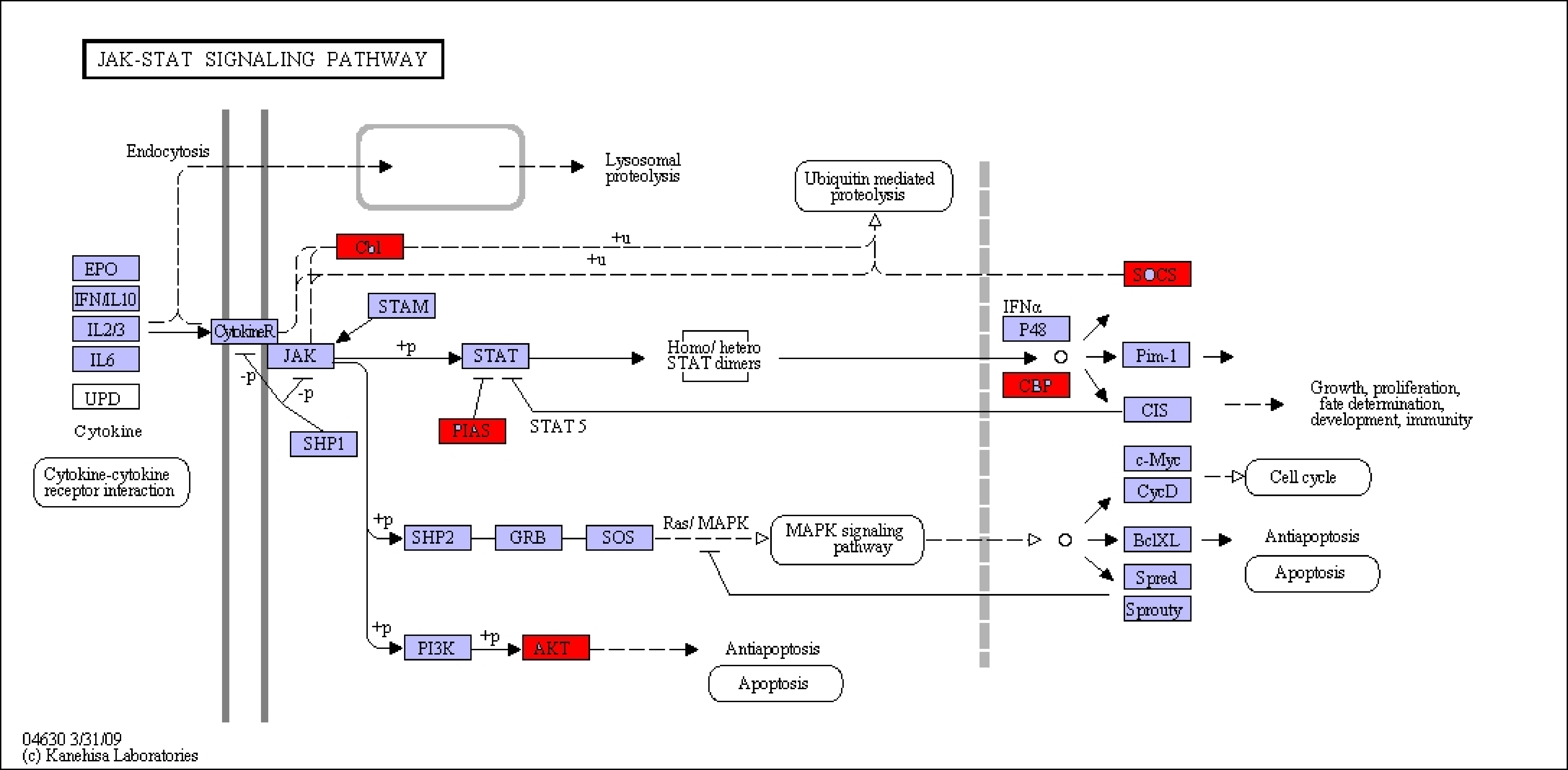

JAK-STAT SIGNALING PATHWAY
Endocytosis
Lysosomal proteolysis
Ubiquitin mediated proteolysis
Cbl
+u
+u
SOCS
EPO
IFN/IL10
IL2/3
IL6
UPD
Cytokine
CytokineR
STAM
JAK
+p
STAT
Homo/ hetero STAT dimers
IFNα
P48
CBP
Pim-1
CIS
Growth, proliferation, fate determination, development, immunity
-p
-p
SHP1
PIAS
STAT 5
Cytokine-cytokine receptor interaction
c-Myc
CycD
Cell cycle
+p
SHP2
GRB
SOS
Ras/ MAPK
MAPK signaling pathway
BclXL
Antiapoptosis
Apoptosis
Spred
Sprouty
+p
PI3K
+p
AKT
Antiapoptosis
Apoptosis
04630 3/31/09
(c) Kanehisa Laboratories

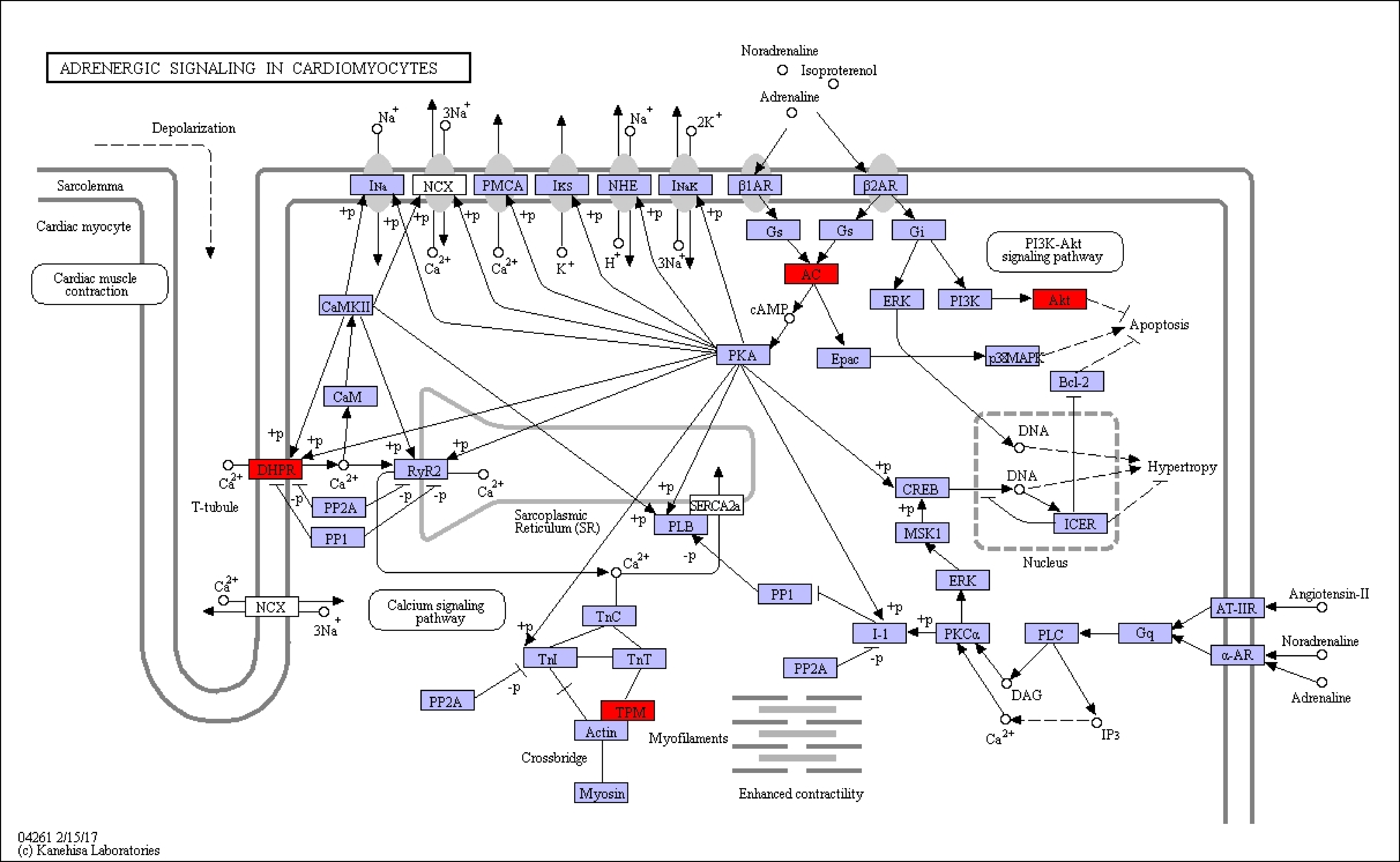
ADRENERGIC SIGNALING IN CARDIOMYOCYTES
Noradrenaline
Isoproterenol
Adrenaline
Depolarization
Sarcolemma
Cardiac myocyte
Cardiac muscle contraction
INa
NCX
PMCA
IKs
NHE
INaK
β1AR
β2AR
Gs
Gs
Gi
AC
PI3K-Akt signaling pathway
ERK
PI3K
Akt
Apoptosis
cAMP
PKA
Epac
p38 MAPK
Bcl-2
CaMKII
CaM
DHPR
RyR2
PP2A
PP1
T-tubule
Sarcoplasmic Reticulum (SR)
SERCA2a
PLB
DNA
Hypertropy
CREB
MSK1
ICER
Nucleus
ERK
PP1
NCX
3Na+
Calcium signaling pathway
TnC
TnI
TnT
PP2A
TPM
Actin
Myosin
Crossbridge
Myofilaments
Enhanced contractility
I-1
PP2A
PKCα
PLC
Gq
AT-IIR
α-AR
Angiotensin-II
Noradrenaline
Adrenaline
DAG
IP3
04261 2/15/17
(c) Kanehisa Laboratories

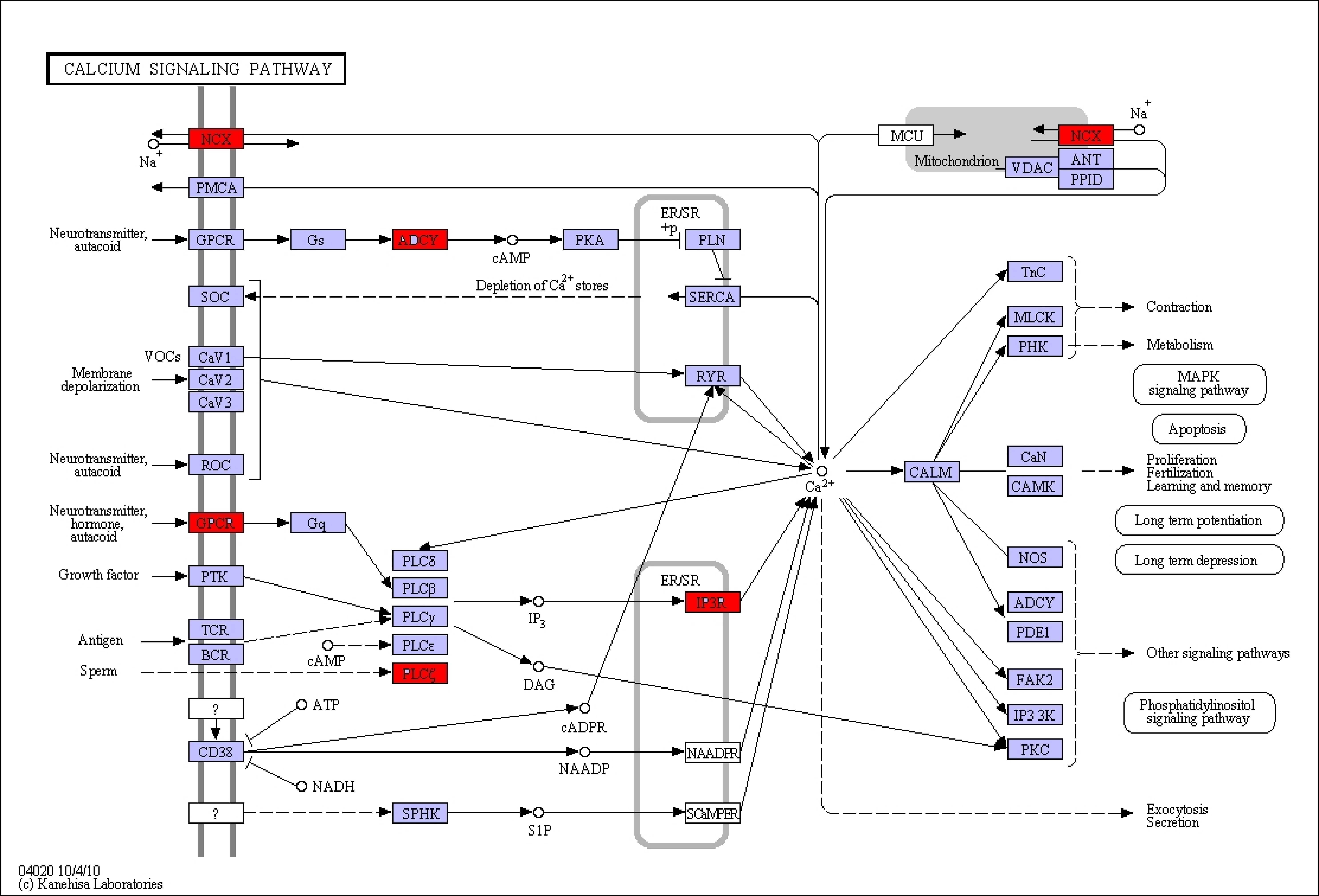

CALCIUM SIGNALING PATHWAY
NCX
Na+
PMCA
Neurotransmitter, autacoid
GPCR
Gs
ADCY
cAMP
PKA
ER/SR
+p
PLN
SERCA
SOC
Depletion of Ca2+ stores
VOCs
CaV1
CaV2
CaV3
Membrane depolarization
RYR
ROC
Neurotransmitter, autacoid
Neurotransmitter, hormone, autacoid
GPCR
Gq
PLCδ
PLCβ
Growth factor
PTK
PLCγ
TCR
BCR
Antigen
PLCε
cAMP
Sperm
PLCζ
IP3
DAG
ER/SR
IP3R
?
ATP
CD38
cADPR
NAADP
NAADPR
NADH
?
SPHK
S1P
SCaMPER
MCU
NCX
Na+
Mitochondrion
VDAC
ANT
PPID
TnC
MLCK
PHK
Contraction
Metabolism
MAPK signaling pathway
Apoptosis
Ca2+
CALM
CaN
CAMK
Proliferation
Fertilization
Learning and memory
Long term potentiation
Long term depression
NOS
ADCY
PDE1
Other signaling pathways
FAK2
IP3 3K
PKC
Phosphatidylinositol signaling pathway
Exocytosis
Secretion
04020 10/4/10
(c) Kanehisa Laboratories

FATTY ACID METABOLISM

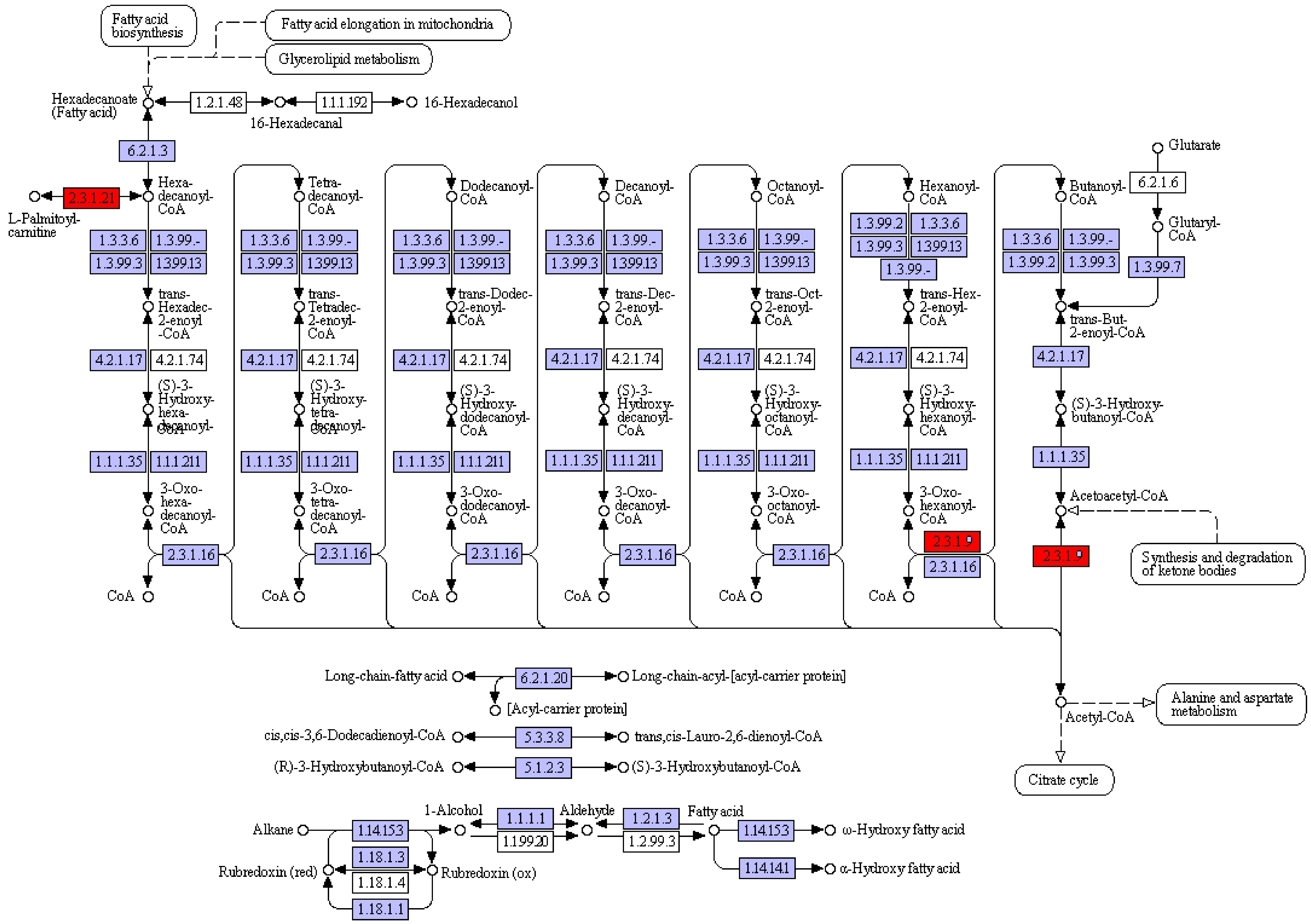

00071 2/24/11
(c) Kanehisa Laboratories

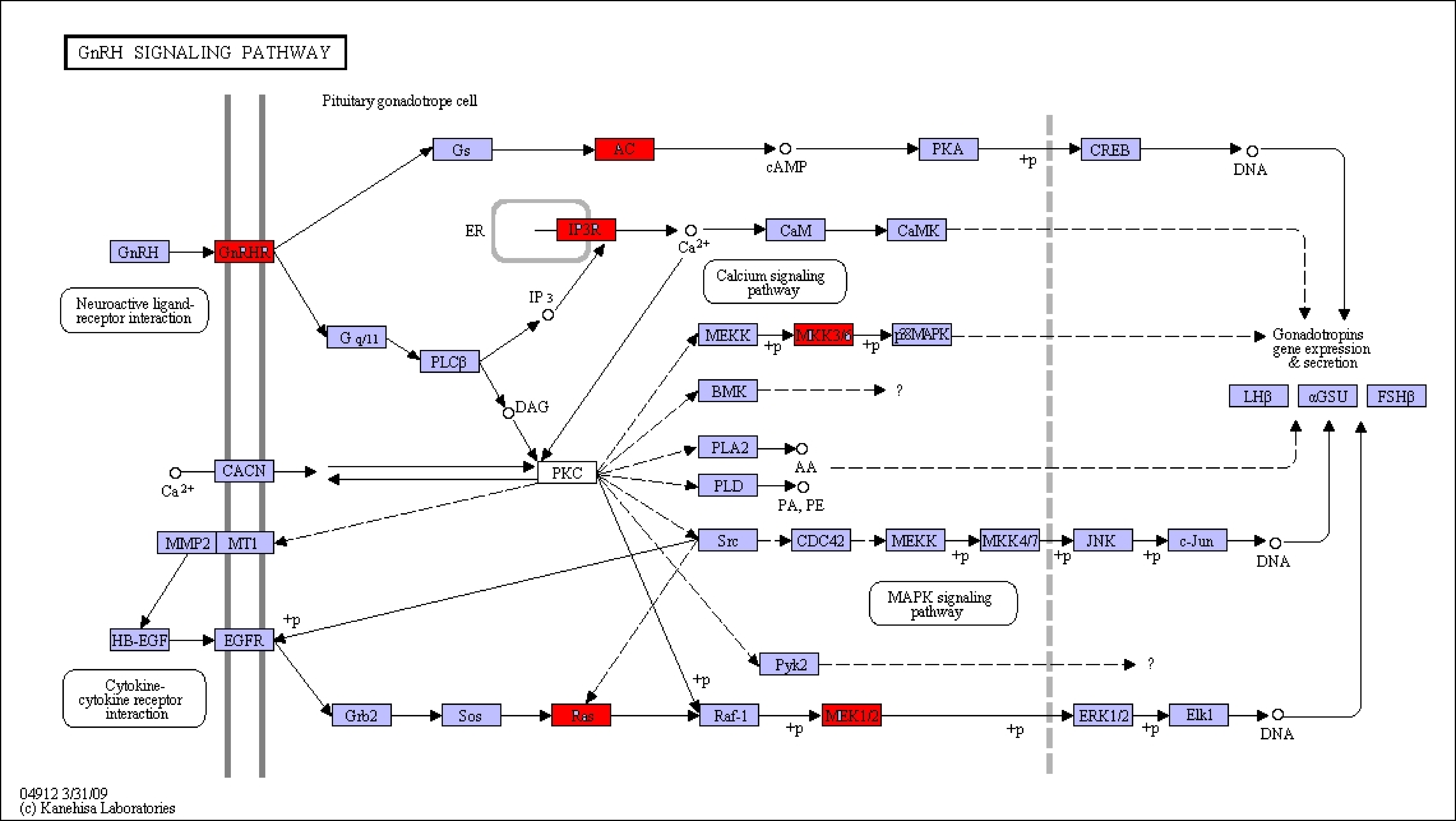
GnRH SIGNALING PATHWAY
Pituitary gonadotrope cell
GnRH
GnRHR
Neuroactive ligand-receptor interaction
Gs
AC
cAMP
PKA
+p
CREB
DNA
ER
IP3R
Ca2+
CaM
CaMK
Calcium signaling pathway
IP 3
G q/11
PLCβ
DAG
MEKK
+p
MKK3/6
+p
p38MAPK
Gonadotropins gene expression & secretion
LHβ
αGSU
FSHβ
BMK
?
PLA2
AA
PLD
PA, PE
Ca 2+
CACN
PKC
MMP2
MT1
Src
CDC42
MEKK
+p
MKK4/7
+p
JNK
+p
c-Jun
DNA
MAPK signaling pathway
+p
HB-EGF
EGFR
Pyk2
?
Cytokine-cytokine receptor interaction
+p
Grb2
Sos
Ras
Raf-1
+p
MEK1/2
+p
ERK1/2
+p
Elk1
DNA
04912 3/31/09
(c) Kanehisa Laboratories

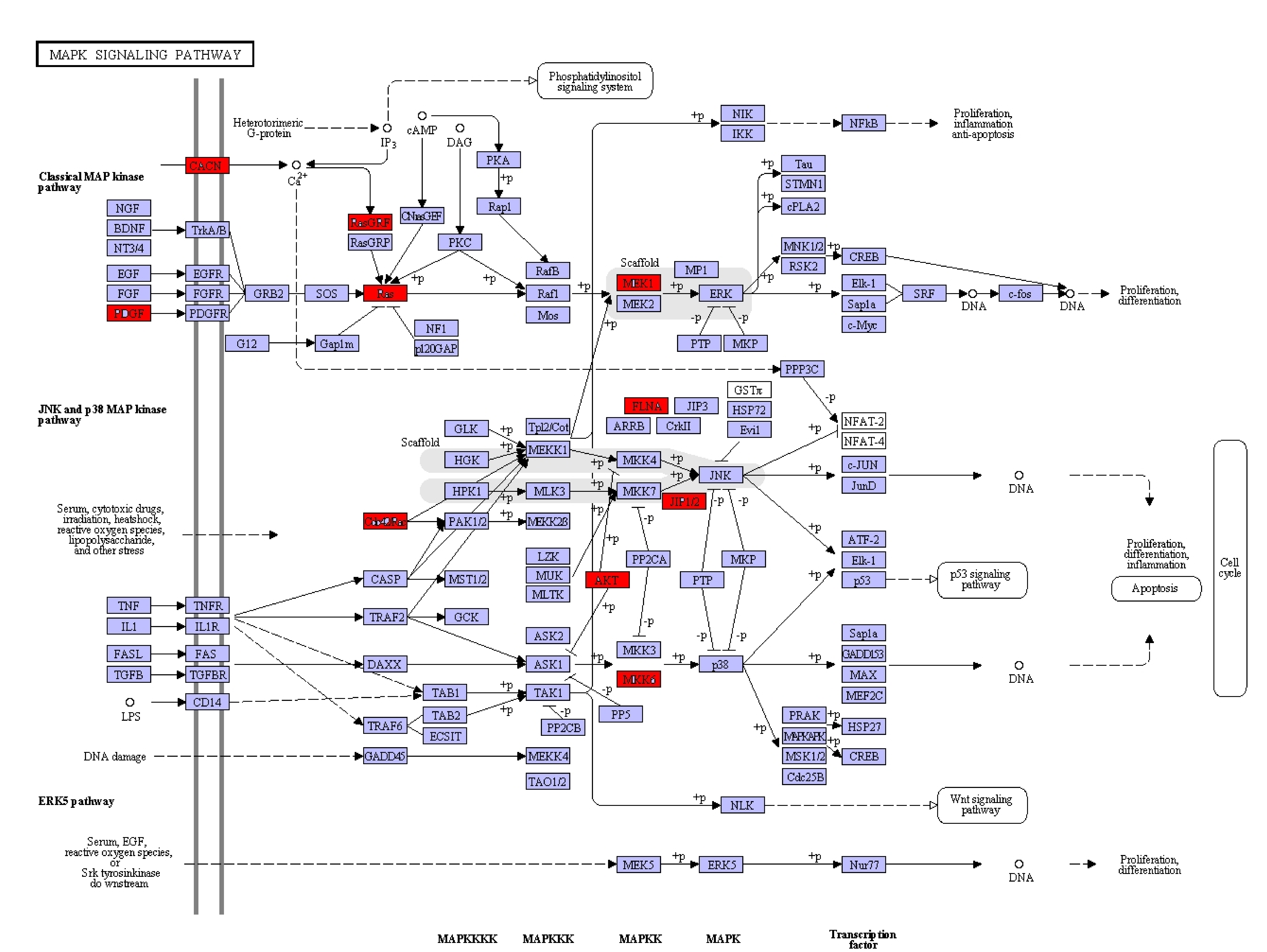

Fig. S3 UTT-associated QTL regions were significantly enriched pathways
Note: The red markers indicate the identify candidate genes linked to UTT, which in the enrich signal pathways.
